# Global Analysis of Post-Translational Side-Chain Arginylation Using Pan-Arginylation Antibodies

**DOI:** 10.1016/j.mcpro.2023.100664

**Published:** 2023-10-12

**Authors:** Brittany MacTaggart, Marie Shimogawa, Marshall Lougee, Hsin-Yao Tang, E.J. Petersson, Anna Kashina

**Affiliations:** 1 School of Veterinary Medicine, University of Pennsylvania, Philadelphia, Pennsylvania, USA; 2 Department of Chemistry, School of Arts and Sciences, University of Pennsylvania, Philadelphia, Pennsylvania, USA; 3 Proteomics and Metabolomics Facility, Wistar Institute, Philadelphia, Pennsylvania, USA

**Keywords:** arginylation, arginyltransferase, side-chain arginylation, global identification, proteomics

## Abstract

Arginylation is a post-translational modification mediated by the arginyltransferase 1 (ATE1), which transfers the amino acid arginine to a protein or peptide substrate from a tRNA molecule. Initially, arginylation was thought to occur only on *N-*terminally exposed acidic residues, and its function was thought to be limited to targeting proteins for degradation. However, more recent data have shown that ATE1 can arginylate side chains of internal acidic residues in a protein without necessarily affecting metabolic stability. This greatly expands the potential targets and functions of arginylation, but tools for studying this process have remained limited. Here, we report the first global screen specifically for side-chain arginylation. We generate and validate “pan-arginylation” antibodies, which are designed to detect side-chain arginylation in any amino acid sequence context. We use these antibodies for immunoaffinity enrichment of side-chain arginylated proteins from wildtype and *Ate1* knockout cell lysates. In this way, we identify a limited set of proteins that likely undergo ATE1-dependent side-chain arginylation and that are enriched in specific cellular roles, including translation, splicing, and the cytoskeleton.

## Highlights

• Pan-arginylation antibodies recognize side-chain arginylation in biological samples.
• Pan-arginylation antibodies facilitate identification of side-chain arginylation.
• Side-chain arginylation is enriched in specific biological pathways.

## Graphical Abstract

**Figure:**
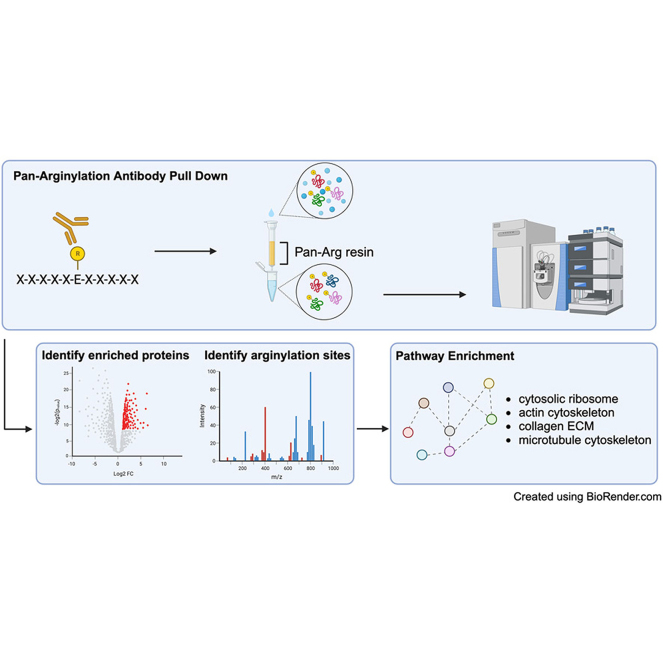

## In Brief

We used immunoaffinity chromatography and mass spectrometry to identify a set of proteins that likely undergo ATE1-dependent side-chain arginylation. These proteins are enriched in specific cellular roles, including translation, splicing, and the cytoskeleton.

Arginylation is a post-translational modification, in which the amino acid arginine (Arg, R) is transferred from a tRNA molecule onto a protein by the enzyme arginyltransferase 1 (ATE1) (reviewed in Ref. (1)). ATE1 is the only known arginyltransferase and is conserved in all eukaryotes (2, 3, 4). In mice, *Ate1* deletion results in embryonic lethality starting at E12.5 because of various developmental defects, including impaired angiogenesis and cardiac morphogenesis (5). Further studies using tissue-specific knockouts of *Ate1* have shown that it plays a role in neural crest cell migration and craniofacial morphogenesis (6), cardiac myofibril formation and contractility (7), germ cell development (8), cancer metastases (9), and neurite outgrowth and brain development (10). Despite the broad biological consequences of depleting ATE1, the protein targets of arginylation that mediate these effects have still not been well defined.

Initially, it was believed that arginylation could only occur on *N-*terminally exposed glutamate (Glu, E), aspartate (Asp, D), or oxidized cysteine (Cys, C) residues and that these arginylated proteins are marked for proteasomal degradation *via* the N*-*end rule pathway (3, 4, 5, 11). However, global arginylation screens have since shown that the majority of arginylated proteins are metabolically stable (12). This opened the door to studying arginylation as a post-translational modification that can functionally alter a protein, rather than just targeting it for degradation. For example, *N-*terminal arginylation of β-actin has been shown to occur at the leading edge of cells to promote actin polymerization and cell migration (13, 14, 15, 16).

More recently, arginylation on the side chains of Glu and Asp residues of intact proteins has been reported. Specifically, arginylation on a midchain glutamate residue in the regulatory peptide neurotensin was identified, with the linkage occurring *via* an isopeptide bond between the carboxyl group in the glutamate side chain and the amino group of arginine*.* This modification was identified *in vivo* and was shown to slightly increase the affinity of neurotensin for its receptor (17). Further studies have confirmed that ATE1 can directly modify midchain Glu and Asp residues of both peptide and protein substrates *in vitro*. These studies also showed that ATE1 does not recognize a specific consensus sequence and might instead rely on the general physiochemical properties of the surrounding residues or on specific secondary structural features of the protein (18, 19).

The discovery that arginylation can occur on the side chains of acidic residues within a protein, rather than being restricted to those exposed at the *N* terminus through prior proteolytic processing, significantly broadened the scope of potential biological regulation by arginylation. Indeed, a reanalysis of mass spectrometry (MS) data from previous studies without limiting the search to N-terminal acidic residues identified many additional arginylation sites on midchain Glu and Asp residues (18). However, the identification of arginylation in complex protein samples is often hindered by the relatively low abundance of this modification on proteins. For example, less than 1% of cellular β-actin is estimated to have N-terminal arginylation (20). This leaves many questions about which proteins are targets of midchain arginylation by ATE1.

One method to improve detection of a post-translational modification in complex protein samples is to enrich for the modification prior to performing MS. A previous study successfully used antibodies generated against N*-*terminally arginylated Glu and Asp on peptides with otherwise low immunogenicity to enrich for arginylated proteins in wildtype cell and mouse embryo extracts (12). Many of the arginylation sites identified in this study were on midchain residues, but no global screen specifically enriching for side-chain arginylation has been performed to date.

In this study, we develop and validate antibodies against peptides containing side-chain arginylation on glutamate, termed “pan-arginylation” antibodies. We use these antibodies for immunoaffinity enrichment of arginylated proteins from cell lysates. Using *Ate1* knockout cells as a negative control, we identify a limited set of proteins that undergo side-chain arginylation.

## Experimental Procedures

### Generation of Peptides and Antibodies

Synthesis of the arginylated peptide library XXXXXE^Arg^XXXXXC (X: mixture of 19 noncysteine natural amino acids, methionine is replaced with norleucine to prevent oxidation issues) was performed on the 100 μmol scale for generation of antibodies recognizing arginylation regardless of the surrounding sequence. This method is expected to generate 6.13 × 1012 different peptides, covering all the theoretically possible sequence combinations. A step-by-step procedure for this synthesis has been described elsewhere (21). A nonarginylated peptide library XXXXXEXXXXXC was synthesized in parallel for the generation of negative control antibodies. The syntheses utilized an Fmoc-based solid phase peptide synthesis strategy and an isokinetic mixture reported previously (22, 23). A previously described method was used for the synthesis of Fmoc-Glu(Arg(Pbf)-OtBu)-OH (24). The general structure of the peptide library and characterization of the products by MALDI can be found in Supplemental Fig. S1.

2-Chlorotrityl resin was swollen in v1:1 dichloromethane/dimethylformamide (DMF) for 30 min. The first amino acid Cys was coupled using 2 eq Fmoc-Cys(Trt)-OH at a concentration of 0.15 M in DMF and 4 eq of *N,N-*diisopropylethylamine, reacted for 15 min at room temperature. Unreacted sites were capped with methanol. Fmoc deprotection was performed after stirring in 20 v % piperidine/DMF for 5 min, repeated once, and the resin was adjusted to 100 μmol.

For coupling of X residues, the isokinetic mixture was prepared by combining a mixture of 19 natural amino acids (240 eq total per residue) at the ratio determined previously with N-hydroxybenzotriazole (240 eq per residue). Coupling was carried out for an hour at room temperature, following activation of the amino acids by *N*,*N*′-diisopropylcarbodiimide (240 eq, 3.716 ml). Fmoc deprotection was performed as described previously. Coupling of Fmoc-Glu(Arg(Pbf)OtBu)-OH (2 eq) was carried out at 37 °C for 1 to 2 h with shaking at 250 rpm, using 2 eq of the amino acid in DMF with 1.9 eq 2-(1H-benzotriazol-1-yl)-N,N,N’,N’-tetramethyluronium hexafluorophosphate and 4 eq *N,N-*diisopropylethylamine.

Peptide cleavage from the resin was performed using 30 μl of Reagent K (82.5% v/v TFA, 5% w/v phenol, 5% v/v water, 5% v/v thioanisole, and 2.5% v/v 1.2-ethanedithiol) per mg of resin and agitating for 1.5 h. Cocktail containing cleaved peptide was collected, and peptide was precipitated using at least 10-fold volume of diethyl ether. The crude pellet was dissolved in acetonitrile–water mixture and lyophilized.

Three rabbit polyclonal antibodies were generated against this peptide mixture (GenScript) using one primary immunization (day 0) and three boosts at days 14, 28, and 42, followed by the final production bleed at day 49. Antibodies were purified from the crude serum on Protein A agarose and stored at −80 °C prior to use.

### Validation of Antibodies

The arginylated and unmodified peptide mixtures were dissolved to 1.6 mg/ml in dimethyl sulfoxide and serially diluted to 0.8, 0.4, 0.2, 0.1, and 0.05 mg/ml in Milli-Q water. Synuclein samples were diluted to 20 μM in Milli-Q water. Dot blots were performed by dotting 1 to 2 μl of each sample onto an 0.45 μm nitrocellulose membrane. After drying, total protein and antibody staining were performed as described for Western blots.

### Western Blotting

Protein samples were run on 12% SDS-PAGE gels at 100 V for 90 min and transferred to 0.45 μm nitrocellulose membranes at 100 V for 60 min. Blots were stained with REVERT Total Protein Stain (LI-COR) and imaged before blocking. Blots were blocked in 3% bovine serum albumin in PBS with Tween-20 for 1 h at room temperature and then incubated with primary antibodies at 4 °C overnight. The other antibodies used in this study were mouse anti-α-tubulin (catalog no.: MABT205; Sigma) and rabbit anti-β-tubulin (catalog no.: 2146; Cell Signaling). The blots were washed and incubated with secondary antibodies (1:10,000 dilution) conjugated to IRDye800 or IRDye680 (LI-COR) for 1 h at room temperature. Images were acquired and quantified using the Odyssey Imaging System (LI-COR).

### Cell Culture

*Ate1*^*+/+*^ and *Ate1*^*−/−*^ mouse embryonic fibroblasts (MEFs) were isolated from littermate E12.5 embryos and immortalized by continuous passaging in culture as previously described (5). The cells were grown in Dulbecco’s modified Eagle’s medium with GlutaMAX (Gibco) supplemented with 10% fetal bovine serum and 1% penicillin–streptomycin at 37 °C and 5% CO_2_.

### Immunoaffinity Enrichment

For each pan-arginylation antibody, 2 to 3 mg of antibody was coupled to 600 μl of agarose resin (AminoLink Plus Immobilization Kit; ThermoFisher). Coupling efficiency was measured by spectrophotometry and found to be >85%.

Ten 10 cm dishes of each wildtype and *Ate1* knockout cells were grown to ∼80% confluency. Cells were washed once in PBS and collected by scraping and centrifugation. Cells were lysed by resuspension in 5 μl per mg of cell pellet of lysis buffer (50 mM Tris–HCl [pH 7.5], 150 mM NaCl, 0.1% Triton X-100, and 1:50 protease inhibitor cocktail [Sigma] and sonicated on ice [level 5, 20 cycles of 3 s on/3 s off]). Cell lysates were clarified by centrifugation at 16,000*g* for 15 min at 4 °C. Protein concentration was measured using the Pierce BCA Protein Assay Kit (ThermoFisher) and adjusted to 5 mg/ml.

Affinity purification was carried out as described in the AminoLink kit. In short, the resin was equilibrated with wash buffer (50 mM Tris–HCl [pH 7.5] and 150 mM NaCl). Each antibody column was split into two (300 μl) and incubated with 1.5 ml of wildtype or *Ate1* knockout cell lysate at 4 °C overnight with rocking. The flow through and four wash fractions were collected. The columns were eluted into eight fractions using 500 μl of 0.1 M glycine (pH 2.7) into Eppendorf tubes containing 50 μl of 1 M Tris–HCl (pH 8.5). The input, flow through, washes, and eluted fractions were all monitored by dot blot using REVERT Total Protein Stain.

### MS

The input and peak elution fractions for *Ate1*^*+/+*^ (F2) and *Ate1*^*−/−*^ (F1) cell lysates were precipitated using trichloroacetic acid, resuspended in sample buffer, and run into a 4 to 12% Bis–Tris protein gel (ThermoFisher) for MS. The entire protein*-*containing lanes were excised, reduced with Tris(2-carboxyethyl)phosphine, alkylated with iodoacetamide, and digested with trypsin. Tryptic digests were analyzed using a 1.5 h LC–MS/MS run on a Thermo Q Exactive Plus mass spectrometer. Tryptic digests were loaded onto a 180 μm × 20 mm nanoACQUITY UPLC Symmetry C18 trap column with 5 μm particle size and 100 Å pore size (Waters) with 0.1% formic acid in Milli-Q water (solvent A). Analytical separation was performed on a 1.7 μm × 250 mm nanoACQUITY UPLC Peptide BEH C18 column with 1.7 μm particle size and 130 Å pore size (Waters) using a 90 min gradient with 0.1% formic acid in acetonitrile (solvent B) as follows: 5 to 30% B over 75 min, 30 to 80% B over 5 min, and constant 80% B over 10 min. Full MS spectra were recorded at a resolution of 70,000 with a scan range of 400 to 2000 *m/z* in profile mode. Full MS automatic gain control target and maximum injection time were set to 3e6 and 50 ms, respectively. MS2 spectra were recorded at 17,500 resolution with MS2 automatic gain control target and maximum injection time set to 5e4 and 50 ms, respectively. Data-dependent analysis was performed on the 20 most abundant ions using an isolation width of 1.5 *m/z* and an underfill of 1%, corresponding to a minimum threshold of 1e4. Peptide match was set to preferred, and unassigned, singly, and >6 charged ions were rejected. Dynamic exclusion was set to 30 s.

MS data were searched with full tryptic specificity (trypsin/P) with a maximum of two missed cleavages against the Swiss-Prot mouse proteome database (25,374 entries, downloaded on July 19, 2021) and a common contaminant database (353 entries; consisting of keratins, bovine, and mycoplasma proteins commonly detected in cell culture, and proteases used in sample digestion) using MaxQuant (Max Planck Institute of Biochemistry), version 1.6.17.0 (25). Precursor mass tolerance was set to 4.5 ppm in the main search, and fragment mass tolerance was set to 20 ppm. Variable modifications considered in the search were Met oxidation and Glu/Asp/peptide N-terminal arginylation, methyl-arginylation, and dimethyl-arginylation. Carbamidomethyl (Cys) was set as a fixed modification. A minimum peptide length of seven residues was required for identification, and up to five modifications per peptide were allowed. No Andromeda score threshold was set for unmodified peptides. A minimum Andromeda score of 40 was required for modified peptides. Protein, peptide, and site false discovery rates were set to 1% based off a target-decoy reverse database.

### Protein Enrichment and Arginylation Site Analysis

Protein enrichment analysis was performed as described (26). Only proteins with Max Razor + unique peptides >1 in *Ate1*^*+/+*^ or *Ate1*^*−/−*^ were analyzed. Quantification was based on intensity, which is the sum of the peptide MS peak areas for the protein. First, the data were log-transformed. Then, the data were normalized across samples by scaling each intensity value against the average intensity of all the proteins in the sample. Missing values, which likely represent proteins present below the detection limit, were imputed by generating a random number between 0 and 0.3 and subtracting 2.5-fold the standard deviation to allow for statistical analysis. Significantly enriched proteins were identified using the following criteria: (1) average fold change >1.5 after normalizing to input, (2) *p* value ≤0.05, and (3) protein was identified in at least two *Ate1*^*+/+*^ samples. For proteins that met the quality control criteria previously described, high-confidence arginylation sites were defined as those that were identified exclusively in *Ate1*^*+/+*^ samples with localization probability >0.75 and posterior error probability (PEP) ≤0.01. Unless otherwise noted, arginylation sites that occurred on the first residue of a peptide directly preceded by Arg in the protein sequence were removed manually.

### Pathway Enrichment Analysis

Network analysis and clustering were performed using STRING (a Core Data Resource by STRING consortium) (27). Interactions were determined using query proteins only with a minimum required interaction score of 0.40 (medium). Clusters were generated using Markov clustering with the inflation parameter set to 1.7.

### Experimental Design and Statistical Rationale

Samples for MS analysis represent one biological replicate analyzed on three independent polyclonal pan-arginylation antibody columns. In each case, *Ate1* knockout cells were used as a negative control for nonspecific enrichment. Differences between the two cell lines were measured using a paired *t* test with a significance level of ⍺ = 0.05. Analyses were performed using Microsoft Excel (version 16.54; Microsoft) and Prism GraphPad (version 9.3.0; GraphPad Software, Inc).

## Results

### Generation and Validation of Pan-Arginylation Antibodies

**Fig. 1:**
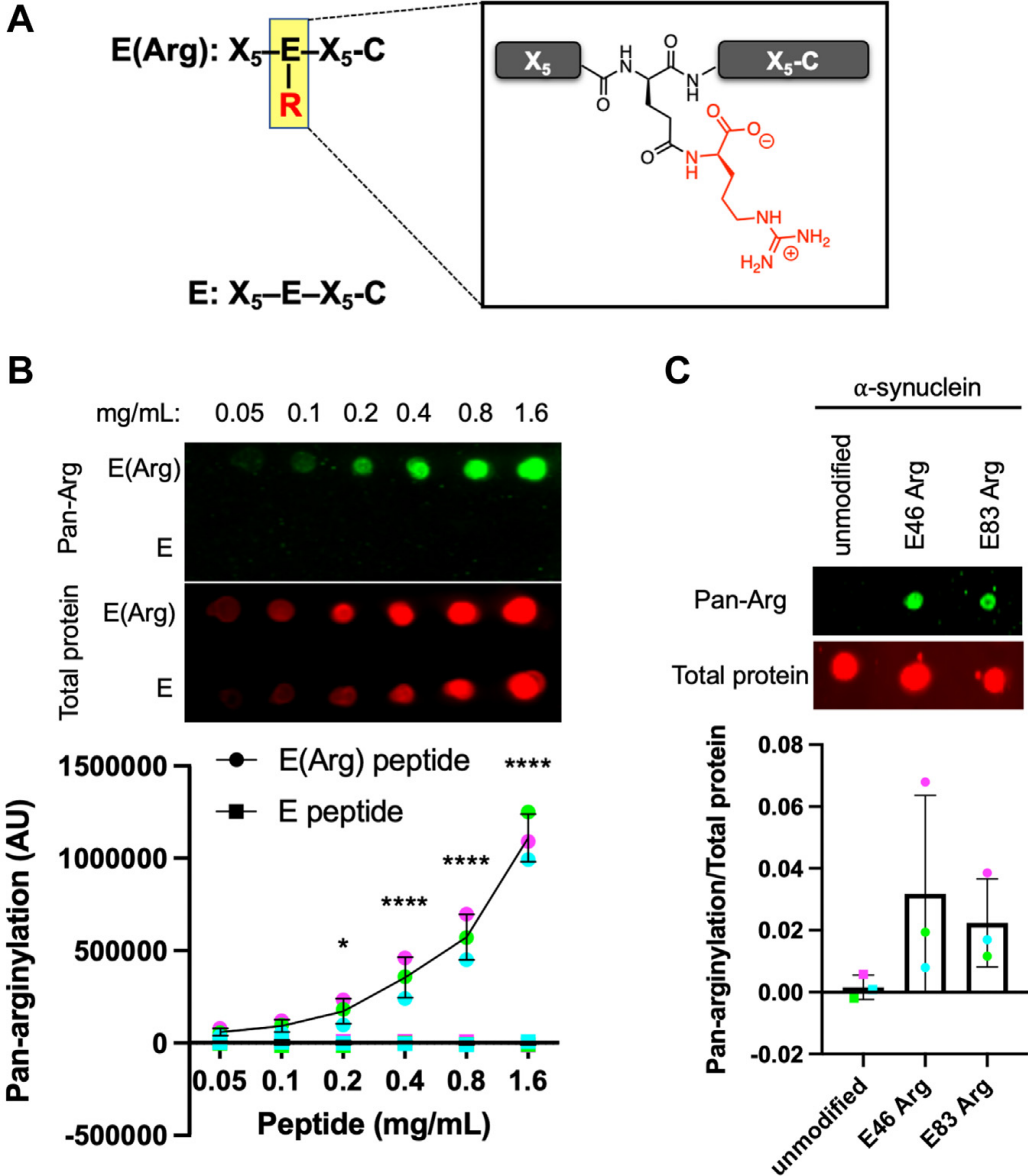
**Generation and validation of pan-arginylation antibodies.***A*, peptide mixtures used for antibody generation and validation. X_5_ indicates a mixture of 19 amino acids (no cysteine). *B*, *top*, dot blot of arginylated and unmodified peptide mixtures using a pan-arginylation antibody (R02988, *green*) and total protein stain (*red*). For all replicates, see Supplemental Fig. S2. *Bottom*, quantification of the pan-arginylation antibody signal (R02988 in *red*, R02989 in *green*, and R02990 in *blue*). Error bars represent SD, n = 3, ∗∗∗*p* = 0.007, repeated-measures two-way ANOVA corrected for multiple comparisons. *C*, *top*, dot blot of arginylated (E46, E83) and unmodified ⍺-synuclein using a pan-arginylation antibody (R02988, *green*) and total protein stain (*red*). For all replicates, see Supplemental Fig. S2. *Bottom*, quantification of the pan-arginylation antibody signal normalized to the total protein stain signal (R02988 in *red*, R02989 in *green*, and R02990 in *blue*). Error bars represent SD, n = 3, ns, repeated-measures one-way ANOVA corrected for multiple comparisons.

To isolate proteins that have undergone side-chain arginylation, three polyclonal antibodies were generated against a mixture of peptides, containing a central arginylated Glu residue flanked by five random amino acids on each side (Fig. 1*A* and Supplemental Fig. S1). Such antibodies should in principle bind to side-chain arginylated glutamates regardless of the surrounding protein sequence, serving as pan-arginylation antibodies. A similar strategy has been successful in identifying proteins that undergo lysine methylation (28).

All three of the resulting pan-arginylation antibodies showed a high specificity for the mixture of arginylated peptides, as compared with a mixture of unmodified peptides (Fig. 1*B* and Supplemental Fig. S2*A*). To further test these antibodies in the context of a full protein, the antibodies were used in dot blots against ⍺-synuclein, a protein previously shown to undergo side-chain arginylation at multiple residues (24, 29, 30). When tested against ⍺-synuclein chemically synthesized to contain arginylation at E46 or E83 (30), the pan-arginylation antibodies reacted with arginylated ⍺-synuclein but not with the unmodified protein (Fig. 1*C* and Supplemental Fig. S2*B*). These results confirm that the pan-arginylation antibodies can reliably detect side-chain arginylation.

### Immunoaffinity Enrichment of Arginylated Proteins

**Fig. 2:**
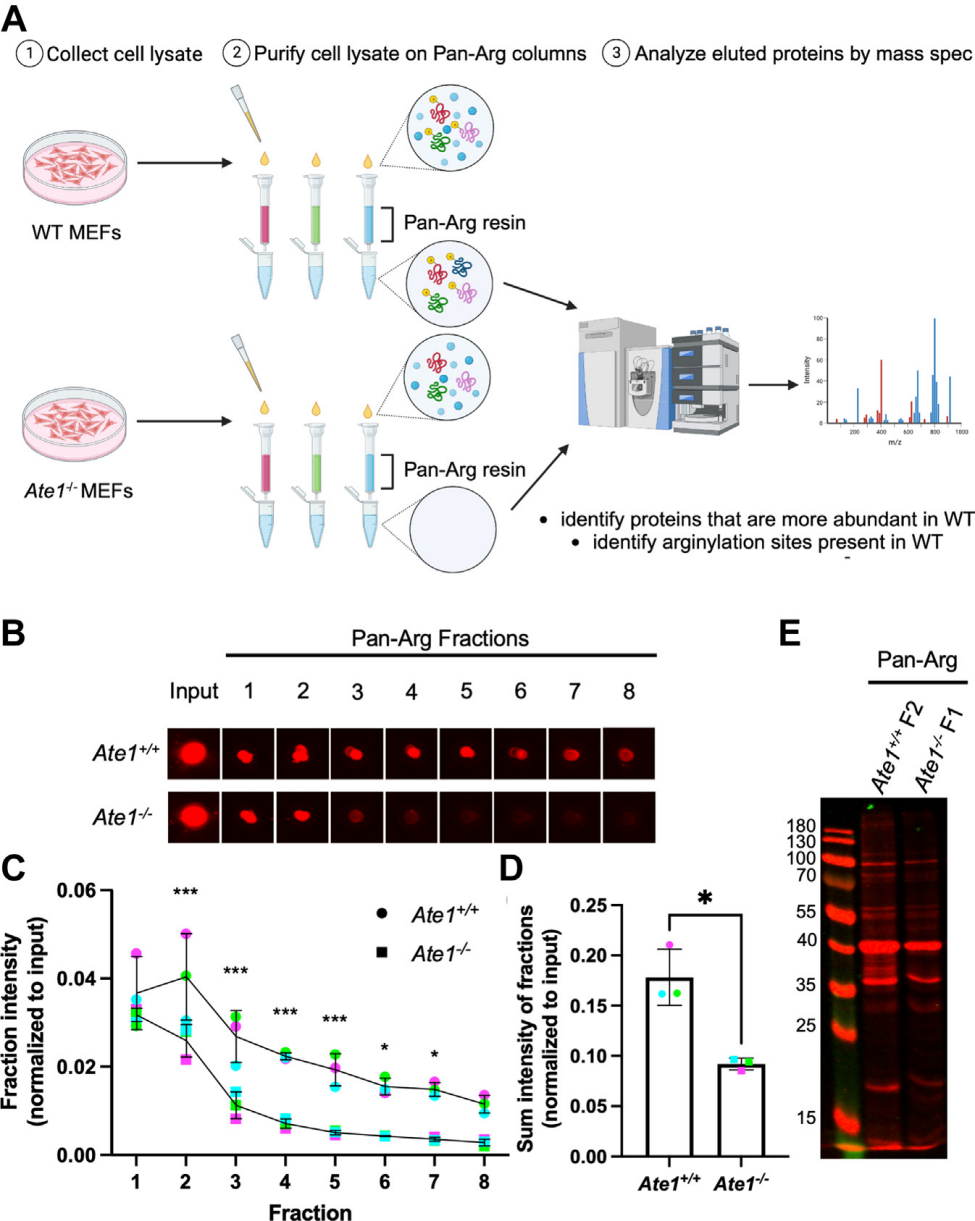
**Pan*-*arginylation antibodies pull down more protein from *Ate1***^***+/+***^**cells than *Ate1***^***−/−***^**cells.***A*, schematic showing the workflow. Wildtype (*Ate1*^*+/+*^) and Ate1 knockout (*Ate1*^*−/−*^) cell lysates were collected and purified on columns coupled to three different pan-arginylation antibodies (R02988 in *red*, R02989 in *green*, and R02990 in *blue*). The peak fractions were subjected to mass spectrometry. Created with BioRender.com. *B*, dot blot showing total protein stain of the input and eluted fractions for *Ate1*^*+/+*^ and *Ate1*^*−/−*^ cell lysates pulled down with a pan-arginylation antibody (R02988). Images were cropped to align the dots, but all scaling and quantification were done on the full image. For all replicates, see Supplemental Fig. S4. *C*, quantification of the average total protein intensity (normalized to input) in each eluted fraction from *Ate1*^*+/+*^ and *Ate1*^*−/−*^ lysates pulled down with the pan-arginylation antibodies (R02988 in *red*, R02989 in *green*, and R02990 in *blue*). Error bars represent SD, n = 3, ∗∗*p* = 0.003, repeated-measures two-way ANOVA corrected for multiple comparisons. *D*, sum of total protein stain intensity (normalized to input) from all eluted fractions from *Ate1*^*+/+*^ and *Ate1*^*−/−*^ lysates pulled down with the pan-arginylation antibodies (R02988 in *red*, R02989 in *green*, and R02990 in *blue*). Error bars represent SD, n = 3, ∗*p* = 0.047, paired *t* test. *E*, Western blot of wildtype fraction 2 (*Ate1*^*+/+*^ F2) and *Ate1* knockout fraction 1 (*Ate1*^*−/−*^ F1) using total protein stain (*red*). For all replicates, see Supplemental Fig. S4.

Our previous studies with N-terminal pan-arginylation antibodies showed that such antibodies do not reliably distinguish between wildtype and *Ate1* knockout cell extracts by Western blot but show promising results in immunoaffinity pulldowns (12). Consistent with this, our side-chain pan-arginylation antibodies were unable to detect robust differences in wildtype (*Ate1*^*+/+*^) and *Ate1* knockout (*Ate1*^*−/−*^) MEF cell lysates by Western blot (Supplemental Fig. S3). We therefore turned to immunoaffinity chromatography to enrich for side-chain arginylated proteins in cell extracts (Fig. 2*A*). For this experiment, we used independently derived immortalized MEF cultures from wildtype (arginylation positive, *Ate1*^+/+^) and Ate1 knockout (arginylation negative, *Ate1*^−/−^) E12.5 embryos. We also used wildtype mouse brain lysates without a negative control.

All three pan-arginylation antibodies were covalently coupled to agarose resin to prevent crosscontamination of the antibodies in the pulldowns. Equal amounts of total lysates from *Ate1*^*+/+*^ and *Ate1*^*−/−*^ MEFs were loaded onto equal volumes of each pan-arginylation antibody column and incubated overnight to allow for binding. The columns were washed four times, and the bound proteins were eluted into eight fractions. The overall protein content of the input, flow through, wash fractions, and elution fractions was monitored by dot blot (Supplemental Fig. S4, *A* and *C*). In this experiment, we reasoned that since the columns are specifically pulling down arginylated proteins, the column eluates from wildtype lysates should contain much more protein than those from *Ate1* knockout lysates, which should constitute only the background signal (Fig. 2*A*).

All three pan-arginylation antibody column eluates showed a much higher protein yield from *Ate1*^*+/+*^ cells than *Ate1*^*−/−*^ cells, suggesting that the pan-arginylation antibodies were binding specifically to arginylated proteins and could potentially enrich them from complex protein mixtures (Fig. 2, *C* and *D* and Supplemental Fig. S4, *A* and *C*). The peak fractions for both *Ate1*^*+/+*^ and *Ate1*^*−/−*^ cells were also analyzed by Western blot using total protein stain to detect the overall protein composition. This analysis showed several bands in wildtype samples that were not present in *Ate1* knockout (Figs 2*E* and Supplemental Fig. S4*B*). However, the pan-arginylation antibodies did not react strongly with any of the bands in eluted *Ate1*^*+/+*^ or *Ate1*^*−/−*^ fractions, confirming that these antibodies are not suitable for use in Western blot (Supplemental Fig. S5).

Thus, side-chain pan-arginylation antibodies can yield enriched preparations of proteins from wildtype arginylation*-*positive samples but not *Ate1* knockout arginylation*-*negative samples. These enriched preparations likely represent the majority of ATE1-dependent side-chain arginylation targets in cells.

To identify arginylation in side-chain pan-arginylation pulldowns, the peak *Ate1*^*+/+*^ and *Ate1*^*−/−*^ fractions were analyzed by MS. In this analysis, we looked for proteins that were considerably more abundant in wildtype *versus* knockout eluates and/or contained arginylation sites specifically in wildtype samples (Fig. 2*A*). We also performed column purification of wildtype mouse brain lysates without matching *Ate1* knockout samples.

### Identification of Proteins Enriched by Pan-Arginylation Antibodies in *Ate1*^+/+^ Cells

**Fig. 3:**
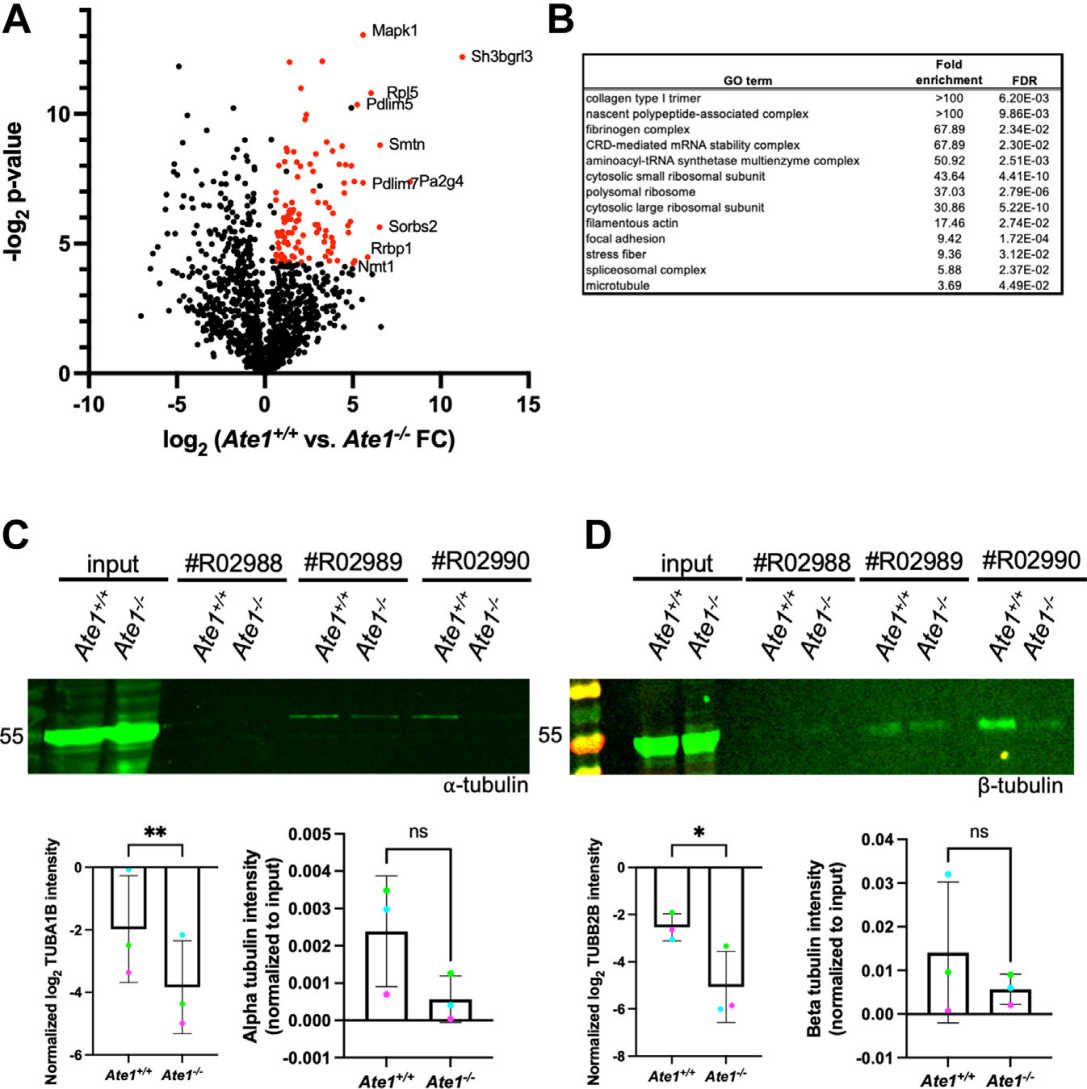
**A subset of proteins is enriched in pan-arginylation pull down samples from *Ate1***^***+/+***^**cells compared with *Ate1***^***−/−***^**cells.***A*, volcano plot of −log_2_(*p* value) *versus* log_2_(*Ate1*^+/+^*versus Ate1*^−/−^ fold change) for proteins identified in the screen. *Red dots* indicate proteins that met the criteria of FC >1.5, *p* value <0.05, and were present in at least two wildtype samples. The top ten most enriched proteins are labeled. *B*, Gene Ontology analysis of proteins enriched in *Ate1*^+/+^ samples. *C*, Western blot confirmation of increased ⍺-tubulin enrichment in *Ate1*^+/+^ samples (*top*) and quantification of TUBA1B from mass spectrometry (*bottom left*) and ⍺-tubulin from Western blot (*bottom right*) with R02988 in *red*, R02989 in *green*, and R02990 in *blue*. *D*, Western blot confirmation of increased β-tubulin enrichment in *Ate1*^+/+^ samples (*top*) and quantification of TUBB2B from mass spectrometry (*bottom left*) and β-tubulin from Western blot (*bottom right*) with R02988 in *red*, R02989 in *green*, and R02990 in *blue*. For uncropped blots, see Supplemental Fig. S6.

To identify the proteins more abundant in wildtype samples, *Ate1*^*+/+*^ *versus Ate1*^*−/−*^ fold change for each protein was calculated for each pan-arginylation column. After filtering for quality control and normalizing to input, the results were filtered for proteins that (1) had an average *Ate1*^*+/+*^ *versus Ate1*^*−/−*^ fold change of at least 1.5 compared with input, (2) had a *p* value ≤0.05, and (3) were identified in *Ate1*^*+/+*^ samples on at least two of the three pan-arginylation columns. Filtering from 1193 total identified proteins, represented by 18,599 identified peptides, this resulted in a final list of 108 enriched proteins (Fig. 3*A* and Table 1).

**Table 1:** Proteins significantly enriched in wildtype samples on all three pan-arginylation antibody columns

Protein names	Gene names	Fold change	*p*
SH3 domain–binding glutamic acid–rich-like protein 3	Sh3bgrl3	2206.8	2.4E-04
Proliferation-associated protein 2G4	Pa2g4	275.7	6.3E-03
Smoothelin	Smtn	99.6	1.1E-03
Sorbin and SH3 domain–containing protein 2	Sorbs2	87.4	1.9E-02
60S ribosomal protein L5	Rpl5	63.7	1.2E-03
Ribosome-binding protein 1	Rrbp1	57.2	4.7E-02
PDZ and LIM domain protein 7	Pdlim7	51.1	5.3E-03
Mitogen-activated protein kinase 1	Mapk1	45.2	9.8E-04
PDZ and LIM domain protein 5	Pdlim5	37.7	9.9E-04
Testin	Tes	35.6	4.1E-03
Glycylpeptide *N*-tetradecanoyltransferase 1	Nmt1	35.5	5.3E-02
Collagen alpha-2(I) chain	Col1a2	32.6	5.2E-02
Nestin	Nes	30.9	2.7E-03
Procollagen C-endopeptidase enhancer 1	Pcolce	30.2	2.1E-02
ATP-dependent Clp protease ATP-binding subunit clpX-like, mitochondrial	Clpx	27.0	4.1E-03
Peptidyl-prolyl *cis*–*trans* isomerase B	Ppib	27.0	2.3E-02
2,4-dienoyl-CoA reductase, mitochondrial	Decr1	26.3	1.8E-02
NADH-cytochrome b5 reductase 3; NADH-cytochrome b5 reductase 3 membrane-bound form; NADH-cytochrome b5 reductase 3 soluble form	Cyb5r3	23.9	6.4E-03
Structural maintenance of chromosomes protein 2	Smc2	22.0	8.0E-03
Tubulin beta-3 chain	Tubb3	21.1	2.3E-03
Prolyl 4-hydroxylase subunit alpha-1	P4ha1	18.3	3.4E-03
Dedicator of cytokinesis protein 7	Dock7	17.1	5.0E-02
Filamin A-interacting protein 1-like	Filip1l	16.3	1.4E-02
Transcription factor BTF3	Btf3	15.5	3.3E-02
Pleckstrin homology-like domain family B member 1	Phldb1	14.9	2.6E-02
Epiplakin	Eppk1	14.3	3.8E-02
Guanine nucleotide-binding protein G(s) subunit alpha isoforms short; guanine nucleotide-binding protein G(s) subunit alpha isoforms XLas	Gnas	13.9	4.7E-03
Leucine–tRNA ligase, cytoplasmic	Lars	13.7	5.1E-02
Dolichyl-diphosphooligosaccharide--protein glycosyltransferase 48 kDa subunit	Ddost	13.6	4.1E-02
Actin-binding protein anillin	Anln	12.6	1.7E-02
Leucine zipper protein 1	Luzp1	12.1	3.2E-02
Microtubule-associated protein 2	Map2	11.6	1.1E-03
Sulfide:quinone oxidoreductase, mitochondrial	Sqrdl	11.6	4.4E-02
NADH dehydrogenase (ubiquinone) 1 alpha subcomplex subunit 8	Ndufa8	11.5	2.6E-02
Myb-binding protein 1A	Mybbp1a	10.9	1.1E-02
Fibulin-2	Fbln2	10.4	2.0E-02
Annexin A6	Anxa6	10.4	3.0E-02
Leiomodin-1	Lmod1	8.9	6.4E-04
Protein-lysine 6-oxidase	Lox	8.5	1.2E-02
60S ribosomal protein L4	Rpl4	8.4	2.1E-02
ATP-binding cassette subfamily F member 1	Abcf1	7.8	1.9E-02
Collagen alpha-1(I) chain	Col1a1	7.7	2.3E-02
Filamin-C	Flnc	7.5	2.8E-03
Ras GTPase-activating-like protein IQGAP1	Iqgap1	7.3	1.9E-02
Tubulointerstitial nephritis antigen-like	Tinagl1	6.8	5.5E-03
Aspartyl/asparaginyl beta-hydroxylase	Asph	6.6	1.2E-02
Collagen alpha-1(XII) chain	Col12a1	6.0	3.7E-03
Tubulin beta-2B chain	Tubb2b	5.8	4.6E-02
Nascent polypeptide-associated complex subunit alpha, muscle-specific form; nascent polypeptide-associated complex subunit alpha	Naca	5.1	1.0E-03
Paired mesoderm homeobox protein 2; paired mesoderm homeobox protein 1	Prrx2; Prrx1	4.9	1.1E-03
60S ribosomal protein L34	Rpl34	4.5	2.5E-02
Reticulon-4	Rtn4	4.2	5.2E-02
Vigilin	Hdlbp	4.2	4.0E-02
Fibronectin; anastellin	Fn1	4.2	4.9E-04
Ras-related protein Rab-2A; Ras-related protein Rab-2B	Rab2a; Rab2b	4.1	3.8E-02
Protein-glutamine gamma-glutamyltransferase 2	Tgm2	3.9	1.2E-02
40S ribosomal protein S16	Rps16	3.8	1.4E-02
40S ribosomal protein S28	Rps28	3.7	3.0E-02
Thrombospondin-1	Thbs1	3.7	2.9E-02
Tubulin alpha-1A chain; tubulin alpha-3 chain	Tuba1a; Tuba3a	3.6	5.3E-03
40S ribosomal protein S23	Rps23	3.5	3.6E-03
Serine/threonine-protein phosphatase 2A 55 kDa regulatory subunit B alpha isoform; serine/threonine-protein phosphatase 2A 55 kDa regulatory subunit B delta isoform	Ppp2r2a; Ppp2r2d	3.5	1.7E-02
ADP-ribosylation factor-like protein 8A; ADP-ribosylation factor-like protein 8B	Arl8a; Arl8b	3.4	3.5E-02
A-kinase anchor protein 2	Akap2	3.2	2.1E-02
Bystin	Bysl	3.2	4.5E-02
40S ribosomal protein S2	Rps2	3.1	2.7E-03
Developmentally regulated GTP-binding protein 1	Drg1	3.1	1.3E-02
60S ribosomal protein L18a	Rpl18a	2.9	4.4E-02
60S ribosomal protein L13	Rpl13	2.9	1.0E-02
60S ribosomal protein L26	Rpl26	2.9	2.4E-02
Calponin-2	Cnn2	2.8	1.6E-02
GMP synthase (glutamine-hydrolyzing)	Gmps	2.8	1.3E-02
Serine/arginine-rich splicing factor 2	Srsf2	2.7	5.1E-02
RNA-binding protein 3	Rbm3	2.6	2.4E-04
40S ribosomal protein S5; 40S ribosomal protein S5, N-terminally processed	Rps5	2.5	2.3E-02
LIM and SH3 domain protein 1	Lasp1	2.5	1.2E-02
Allograft inflammatory factor 1-like	Aif1l	2.5	8.0E-03
60S ribosomal protein L38	Rpl38	2.5	5.2E-02
Succinyl-CoA ligase (ADP/GDP-forming) subunit alpha, mitochondrial	Suclg1	2.4	3.0E-02
Serine/arginine-rich splicing factor 3	Srsf3	2.4	2.7E-03
60S ribosomal protein L27	Rpl27	2.4	1.6E-02
Alpha-crystallin B chain	Cryab	2.3	2.5E-02
Serine/threonine-protein kinase DCLK1	Dclk1	2.3	2.4E-03
Ubiquitin-associated protein 2-like	Ubap2l	2.2	1.1E-02
40S ribosomal protein S3a	Rps3a	2.2	3.5E-03
40S ribosomal protein S14	Rps14	2.2	2.6E-02
Aminoacyl tRNA synthase complex-interacting multifunctional protein 2	Aimp2	2.2	1.8E-02
60S ribosomal protein L27a	Rpl27a	2.2	5.2E-02
F-actin-capping protein subunit alpha-1	Capza1	2.1	3.0E-02
Heterogeneous nuclear ribonucleoprotein F; heterogeneous nuclear ribonucleoprotein F, N-terminally processed	Hnrnpf	2.1	1.6E-02
Uridine 5-monophosphate synthase; orotate phosphoribosyltransferase; orotidine 5-phosphate decarboxylase	Umps	2.0	4.7E-02
ATP-dependent RNA helicase DDX3X; putative ATP-dependent RNA helicase Pl10	Ddx3x; D1Pas1	2.0	5.2E-02
Protein hook homolog 3	Hook3	2.0	3.8E-02
ATP synthase subunit O, mitochondrial	Atp5o	1.9	2.5E-02
Heterogeneous nuclear ribonucleoprotein U	Hnrnpu	1.9	4.2E-02
Eukaryotic initiation factor 4A-I	Eif4a1	1.9	5.2E-02
Filamin-B	Flnb	1.8	2.4E-02
Heterogeneous nuclear ribonucleoprotein H; heterogeneous nuclear ribonucleoprotein H, N-terminally processed	Hnrnph1	1.7	3.5E-02
Ataxin-2-like protein	Atxn2l	1.7	3.9E-03
Polypyrimidine tract-binding protein 1	Ptbp1	1.7	2.5E-02
40S ribosomal protein S27; 40S ribosomal protein S27-like	Rps27; Rps27l	1.7	4.3E-02
Protein S100-A4	S100a4	1.7	3.1E-02
ELAV-like protein 1	Elavl1	1.6	2.3E-02
Actin, alpha cardiac muscle 1; actin, alpha skeletal muscle	Actc1; Acta1	1.6	4.4E-02
Poly(rC)-binding protein 2	Pcbp2	1.6	4.2E-02
CTP synthase 1	Ctps1	1.6	9.8E-03
60S ribosomal protein L9	Rpl9	1.5	8.0E-03
Nuclease-sensitive element-binding protein 1	Ybx1	1.5	5.0E-02

Western blot analysis confirmed the increased abundance of some of these proteins in wildtype fractions compared with *Ate1* knockout fractions (Fig. 3, C and *D* and Supplemental Fig. S6). Importantly, since each polyclonal pan-arginylation antibody will recognize a slightly different set of arginylation sites, this list likely represents an underestimation of the total number of proteins enriched. However, the proteins that showed increased abundance in wildtype fractions on all three pan-arginylation columns are strong candidates for targets of arginylation. Gene Ontology analysis showed that the proteins enriched in pan-arginylation fractions from wildtype cells are preferentially involved in the actin cytoskeleton, microtubule cytoskeleton, and translation (Fig. 3*B*) (31, 32). These pathways have previously been identified as altered in *Ate1* knockout cells (12).

### Identification of Arginylation Sites in *Ate1*^+/+^ Cells

We next analyzed the proteins identified in the column eluates to identify specific arginylated sites. We searched for arginylation on any Asp residue or Glu residue with semitryptic specificity to account for *in vivo* protein processing that may generate truncated N-terminal fragments or functional protein fragments. Importantly, control experiments showed that trypsin does not affect side-chain arginylation, since >99.9% of ⍺-synuclein synthesized with arginylation at E46 remained arginylated following trypsin digestion (Supplemental Fig. S7). We used the *Ate1* knockout as a negative control, reasoning that any arginylation seen in these samples is likely nonenzymatic and definitely not ATE1 dependent. Since this analysis is subject to many ambiguities and requires stringent manual validation, we divided these results into high-, medium-, and low-confidence hits as specified below.

For proteins that met the quality control criteria previously described, high-confidence arginylation sites were defined as those that were identified exclusively in *Ate1*^*+/+*^ samples with a protein localization probability >0.75 and PEP ≤0.01. Based on these criteria, 17 high-confidence arginylation sites on 15 proteins were identified (Table 2). Most of these sites are in regions predicted to be exposed on the surface of the protein and/or in regions of the protein where other post-translational modifications have been reported (33). In addition, 25 non–high-confidence arginylation sites on 22 proteins were identified exclusively in *Ate1*^*+/+*^ samples but on sites with a localization probability <0.75 and/or PEP >0.01 (Supplemental Table S2). Finally, 38 additional arginylation sites were identified in pan-arginylation fractions from wildtype brain samples that did not have a matching *Ate1* knockout negative control. These included 21 high-confidence arginylation sites on 20 proteins and 17 non–high-confidence sites on 17 proteins (Supplemental Tables S3 and S4). It is likely that many of these sites also represent *bona fide* arginylation targets, but more support is needed.

**Table 2:** High-confidence arginylation sites identified exclusively in wildtype samples

Protein names	Gene names	Arginyl (R) site positions	Methyl arginyl (R-me) site positions	Dimethyl arginyl (R-me2) site positions	Peptide	Localization probability	PEP
26S proteasome non-ATPase regulatory subunit 2	Psmd2	E76			DTSLYRPALE(R)ELR	0.95191	0.00496884^a^
Acidic leucine-rich nuclear phosphoprotein 32 family member B	Anp32b	D119, E124			SLD(R)LFGCE(R)VTNRSDYR	0.99997	0.00584857
Actin, alpha cardiac muscle 1	Actc1		D294		D(R-me)LYANNVLSGGTTMYPGIADR	1	2.79152E-11^b^
Actin, alpha cardiac muscle 1; actin, alpha skeletal muscle; actin, cytoplasmic 1; actin, cytoplasmic 2	Actc1; Acta1; Actb; Actg1	D58; D58; D56; D56			DSYVGD(R)EAQSK	0.904826	0.000375832
Anthrax toxin receptor 1	Antxr1	D115			VLPGGD(R)TYMHEGFER	0.996989	0.00619278
CAAX prenyl protease 1 homolog	Zmpste24		E311	E307,D309	NEGE(R-me2)GD(R-me2)SE(R-me)EVK	0.890795	0.00069882
Eukaryotic translation initiation factor 3 subunit G	Eif3g	D5, D7, E21, and D22			PTGD(R)FD(R)SKPSWADQVEEEGE(R)D(R)DK	0.779874	0.00363262
F-actin-capping protein subunit beta	Capzb	E264			SKQEALKNDLVE(R)ALK	0.999565	0.00581404
Myosin-10	Myh10	E1887			QLE(R)EAEEEATR	0.866771	0.00166674
Myosin-9	Myh9	E1385			KKMEDGVGCLETAEE(R)AK	0.83621	0.00066077
Myotubularin-related protein 5	Sbf1	D1341			PD(R)PLQQWELVPIEVFEAR	0.999425	0.000338381
Neurogenic locus notch homolog protein 1	Notch1	E1655, D1657			RE(R)LD(R)PMDIR	0.998716	0.00855934
Palladin	Palld	D220			LLGAD(R)SANVFNIQEPEETAANQEYK	1	9.39096E-17
Plectin	Plec	D685; D537			ID(R)SAEWGVDLPSVEAQLGSHR	0.974335	0.00000241981
Plectin	Plec	E2069; E1921			QVE(R)EEIMALK	0.811853	0.0080895
Ribosome-binding protein 1	Rrbp1	E1081			ESEE(R)ALQK	0.930368	0.0146506
Unconventional myosin-Ic	Myo1c	E55			VGVQDFVLLE(R)NFTSEAAFIENLR	0.983825	2.0807E-12

a E77 identified in KO.

b Arginylation of D292 of Actb/Actg1 identified in KO.

### Pathway Enrichment Analysis of Proteins Enriched and/or Arginylated in *Ate1*^+/+^ Samples

**Fig. 4:**
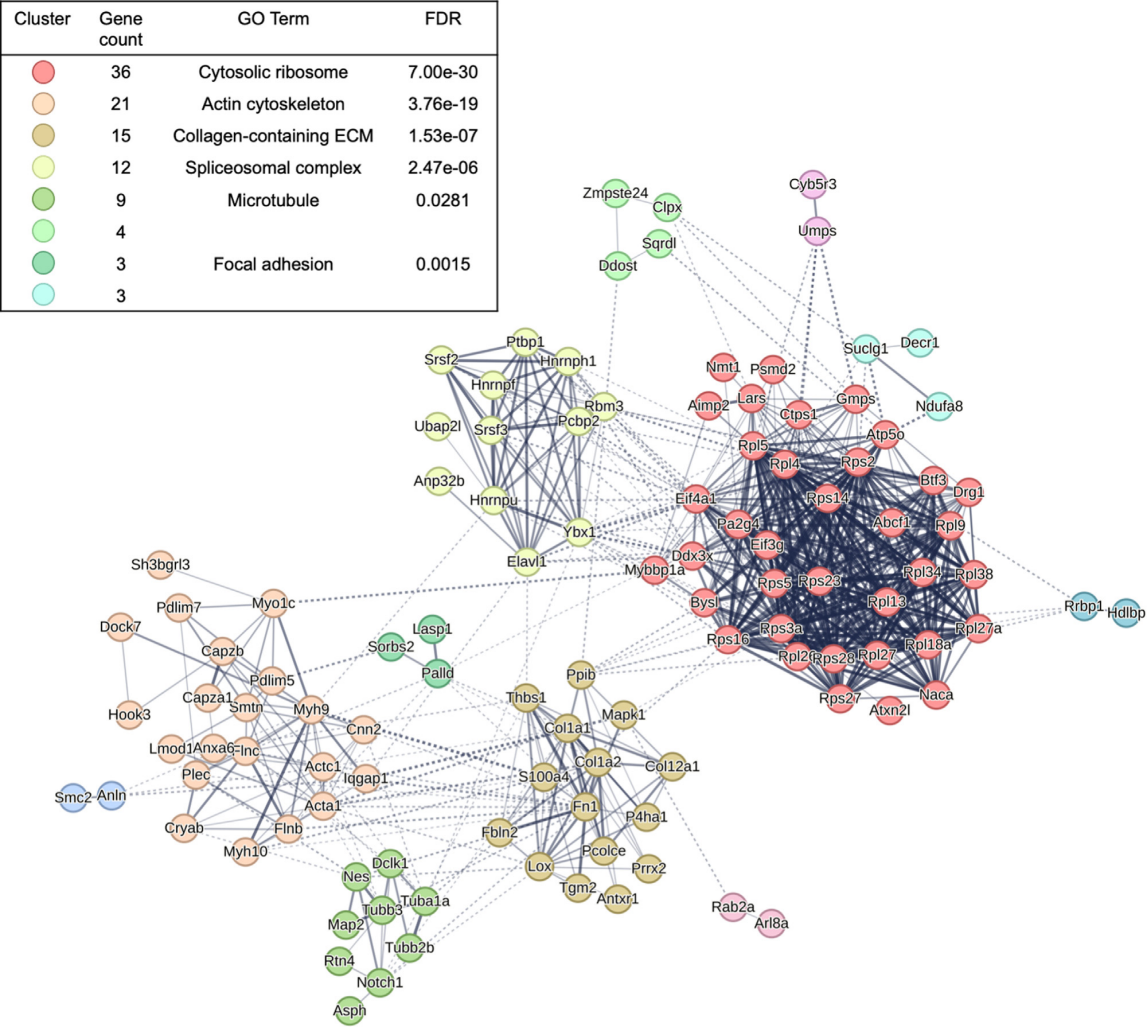
**Pathway analysis of proteins enriched and/or arginylated in *Ate1***^***+/+***^**samples.** Network analysis and clustering were performed using STRING. Interactions were determined using query proteins only with a minimum required interaction score of 0.40 (medium). Clusters were generated using Markov clustering with the inflation parameter set to 1.7. *Solid lines* indicate interactions within clusters. *Dotted lines* indicate interactions between clusters. The thickness of all *lines* indicates the confidence of the interaction.

Proteins that were significantly enriched in and/or had a high-confidence arginylation site identified in *Ate1*^*+/+*^ samples were subjected to network analysis using STRING (Fig. 4) (27). The protein*–*protein interaction *p* value for the network was less than 1.0e-16, suggesting that the proteins identified in this screen are functionally connected. Further clustering analysis organized these proteins into 12 distinct functional clusters. These clusters include some pathways that have previously been shown to be altered in *Ate1*^*−/−*^ cells, including actin cytoskeleton, cortical cytoskeleton, focal adhesions, and microtubule cytoskeleton. Other pathways, such as the cytosolic ribosome and the spliceosomal complex, represent potential areas for future study.

## Discussion

In this study, we performed the first global arginylation screen specifically enriching for side-chain arginylation. We generated antibodies against mixed peptides containing a central arginylated glutamate residue and used these antibodies for immunoaffinity enrichment of arginylated proteins from wildtype and *Ate1* knockout cell lysates. In this way, we identified a set of approximately 100 proteins that likely undergo ATE1-dependent side-chain arginylation. These proteins are enriched in a limited set of cellular locations, including the ribosome, the spliceosome, and the actin and microtubule cytoskeletons. These proteins represent new potential areas of study for understanding the functional consequences of arginylation. In addition, these newly developed pan-arginylation antibodies can now be used to perform similar screens in specific tissues or in cell lines under specific treatments or conditions of interest.

Previous studies from our laboratory performed a similar analysis of N-terminally arginylated proteins (12), providing the first comprehensive list of likely arginylated protein targets that are not subject to arginylation*-*dependent degradation. The current study expands this list to a large number of proteins that likely undergo side-chain arginylation and provides a proof of concept for side-chain arginylation as a global mechanism of protein regulation *in vivo*.

Identification of arginylated sites on these proteins is generally more challenging because of a number of factors, including low abundance, contaminants, limitations of some MS instruments, and numerous mass ambiguities that preclude reliable validation (34). It is also possible that the appended Arg are hydrolyzed during lysis, degrading the arginylation sites. While the method we chose involves the least possible amount of sample handling, as opposed to more expansive methods of identification of post-translational modifications on peptides in preproteolyzed samples, it is still subject to these uncertainties. In many cases, the MS/MS spectra contain sufficient fragment ion information to confidently localize the arginylation site to a specific amino acid residue. In addition, it is important to acknowledge that there are instances where the exact modification site is ambiguous because of the absence of informative fragment ions. Furthermore, the identification of peptides with multiple arginylation sites should be considered tentative despite surviving the 1% false discovery rate cutoff. These considerations should be kept in mind when approaching the results of this and other analyses of the arginylated sites.

Because of all these limitations, specific arginylation sites could be identified on only a small fraction of the proteins found to be more abundant in wildtype cells. Specifically, of the 108 proteins found to be significantly enriched in eluates from wildtype samples *versus Ate1* knockout cells, only six had arginylation sites that could be identified and validated by viewing the mass spectra (Table 2 and Supplemental Table S2). An additional seven proteins had arginylation sites identified in pan-arginylation fractions from wildtype mouse brain samples (Supplemental Tables S3 and S4). It is most likely because only a small fraction of the protein was modified or because the modification was not stable; however, it is also possible that some of these proteins are enriched in wildtype samples because they interact with another protein in an arginylation*-*dependent manner. For example, the ribosome-binding protein Rrbp1 showed over a 50-fold enrichment in *Ate1*^*+/+*^ samples and had an arginylation site identified at E1080, which occurs in a coiled-coil region of the protein shown to be important for polysome formation on the endoplasmic reticulum (35). Several other ribosomal proteins (Rpl5, Rpl4, Rpl34, Rps16, Rps28, Rps23, Rps2, Rpl18a, Rpl13, Rpl26, Rps5, Rpl27, Rps3a, Rps14, Rps27, and Rpl9) did not have any arginylation sites identified but still showed significant enrichment in *Ate1*^*+/+*^ samples. Many additional ribosomal proteins were also enriched in *Ate1*^*+/+*^ samples but did not reach significance.

Proteins identified during our current analysis belong to several distinct functional categories, including components of the actin cytoskeleton and cell adhesion complex, consistent with previous observations of a critical role of arginylation in cell migration (13, 14, 15, 16). Surprisingly, additional distinct functional categories have been identified. Some of them have been previously discussed in potential connection with arginylation, including tubulin (12), mitochondria (36, 37, 38), and ribosomal complex (39). Others, such as spliceosomal proteins, are new and point to additional previously unknown roles of ATE1. Overall, the use of pan-arginylation antibodies, as well as similar antibodies raised against specific arginylated sites, represents a potentially promising tool for dissecting these functions *in vivo*.

Arginylation was first discovered 60 years ago, and in that time, the understanding of its functional consequences has gradually evolved. It was initially thought to be a modification restricted to exposed N-terminal acidic residues that targeted a protein for degradation, but it is now understood that arginylation can occur on the side chains of any available acidic residue in a protein without necessarily impacting its metabolic stability. This vastly expanded the potential targets and functions of arginylation, but the tools for studying these have remained limited. Our study, and other work using new tools for dissecting arginylation, will develop our understanding of this enigmatic but highly essential biological process.

## Data Availability

The MS proteomics data have been deposited to the ProteomeXchange Consortium (http://www.proteomexchange.org/) *via* the MassIVE partner repository (https://massive.ucsd.edu/ProteoSAFe/static/massive.jsp) with the dataset identifier PXD040534 and MSV000091388, respectively.

## Supplemental Data

This article contains supplemental data.

## Conflict of interest

The authors declare no competing interests.
