# Supplementary material for: Global Analysis of Post-Translational Side-Chain Arginylation Using Pan-Arginylation Antibodies
Source: Mol Cell Proteomics. 2023 Oct 12;22(11):100664. doi: 10.1016/j.mcpro.2023.100664 (PMC10656225; doi:10.1016/j.mcpro.2023.100664)
Supplement: Supplemental File 1 [file mmc1.pdf]

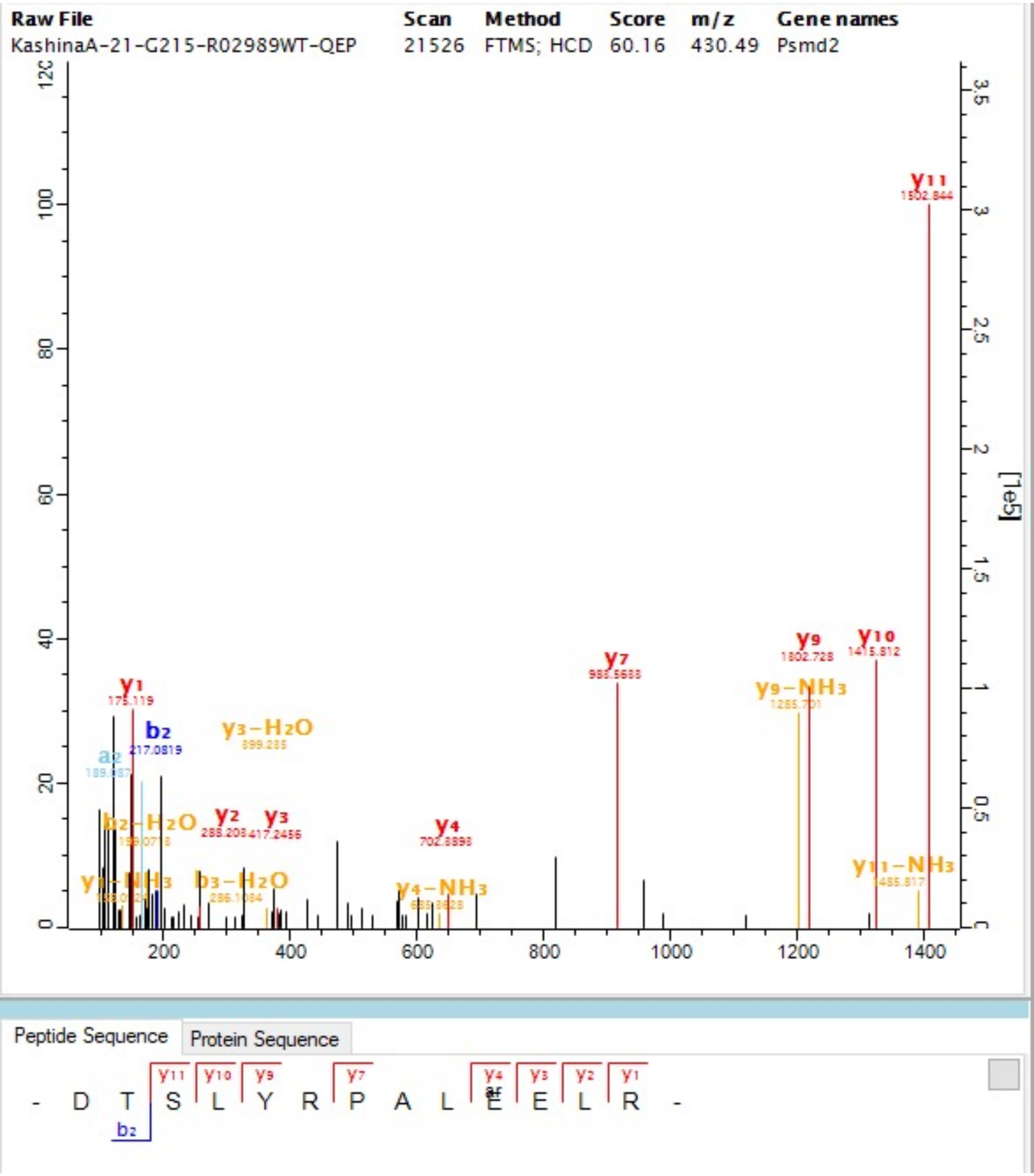

Raw File
KashinaA-21-G215-R02989WT-QEP
Scan
21526
Method
FTMS; HCD
Score
60.16
m/z
430.49
Gene names
Psmd2
y11
y10
1415.812
y9
1302.728
y7
y9-NH3
1285.701
y1
175.119
b2
217.0819
y3-H2O
a2
b2-H2O
y2
y3
417.2456
y4
b3-H2O
y4-NH3
y11-NH3
[1e5]
Peptide Sequence
Protein Sequence
- D T S L Y R P A L E E L R -

Raw File	Scan	Method	Score	m/z	Gene names
KashinaA-21-G215-R02990WT-QEP	21823	FTMS; HCD	60.16	430.74	Psmd2

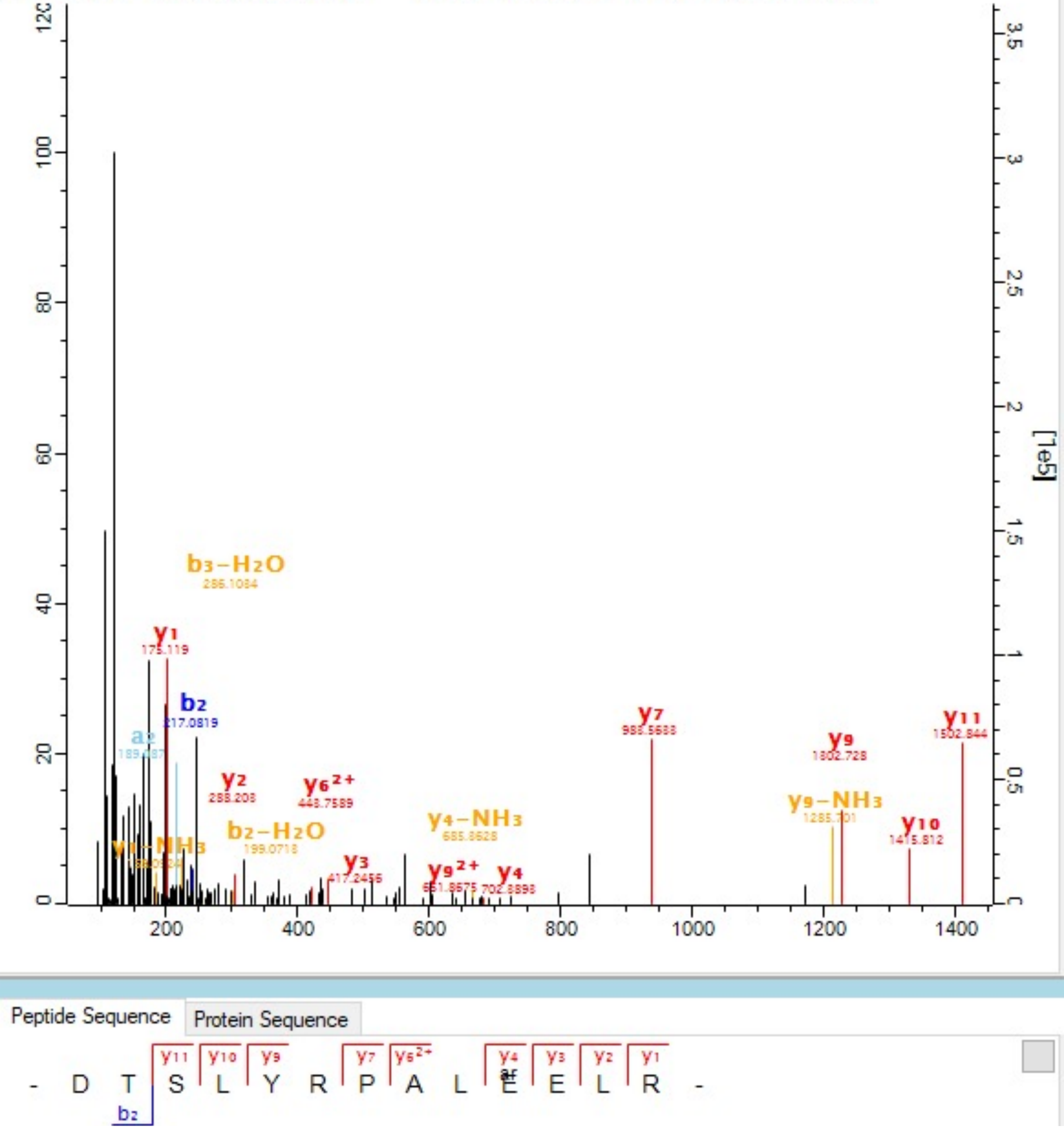

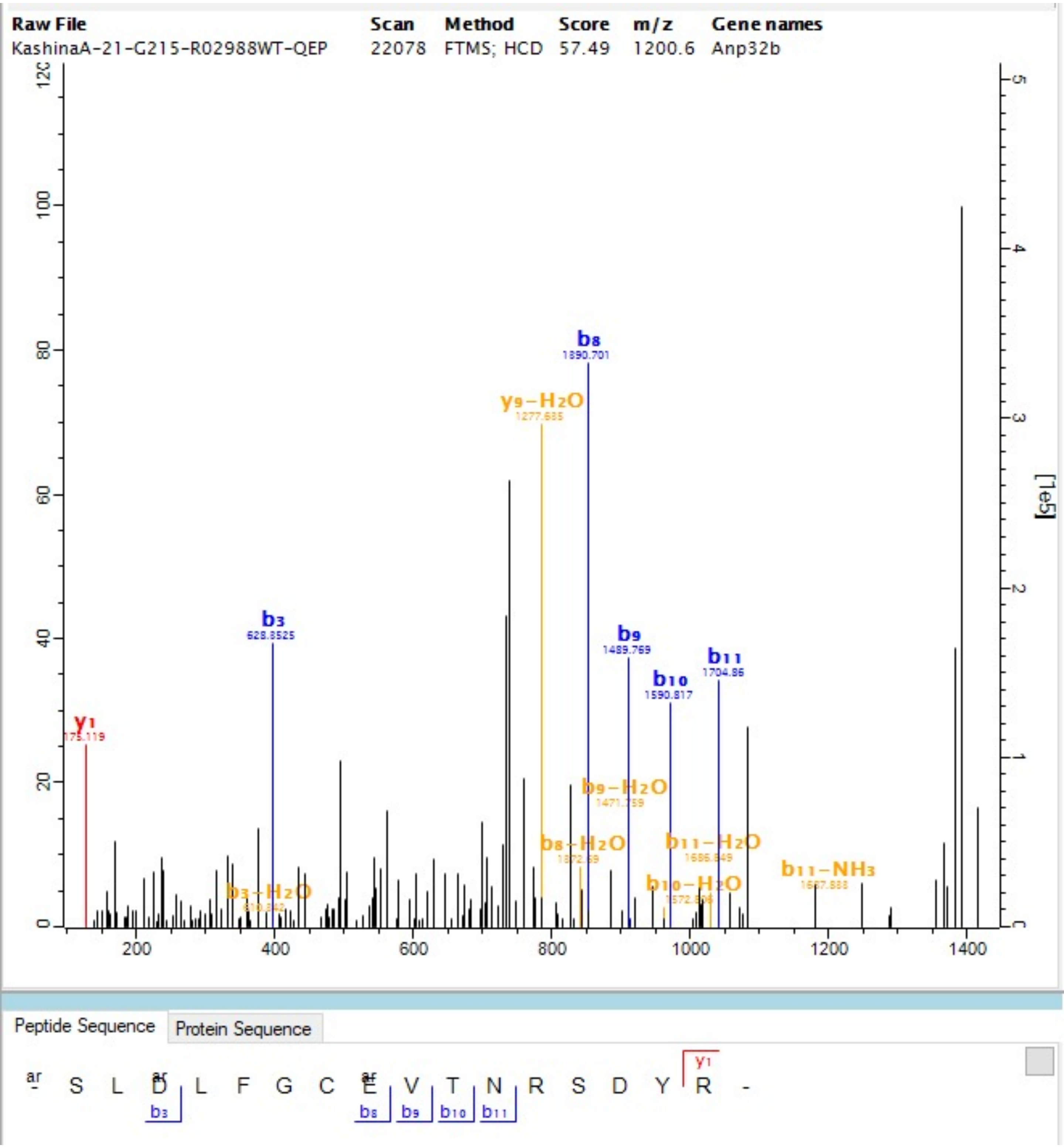
Raw File
KashinaA-21-G215-R02988WT-QEP
Scan
22078
Method
FTMS; HCD
Score
57.49
m/z
1200.6
Gene names
Anp32b
y1
175.119
b3
628.3525
b3-H2O
b8
1890.701
y9-H2O
1277.685
b8-H2O
b9
1489.769
b9-H2O
1471.759
b10
1590.817
b10-H2O
b11
1704.86
b11-H2O
b11-NH3
[1e5]
Peptide Sequence
Protein Sequence
ar
- S L D L F G C E V T N R S D Y R -
b3
b8
b9
b10
b11
y1

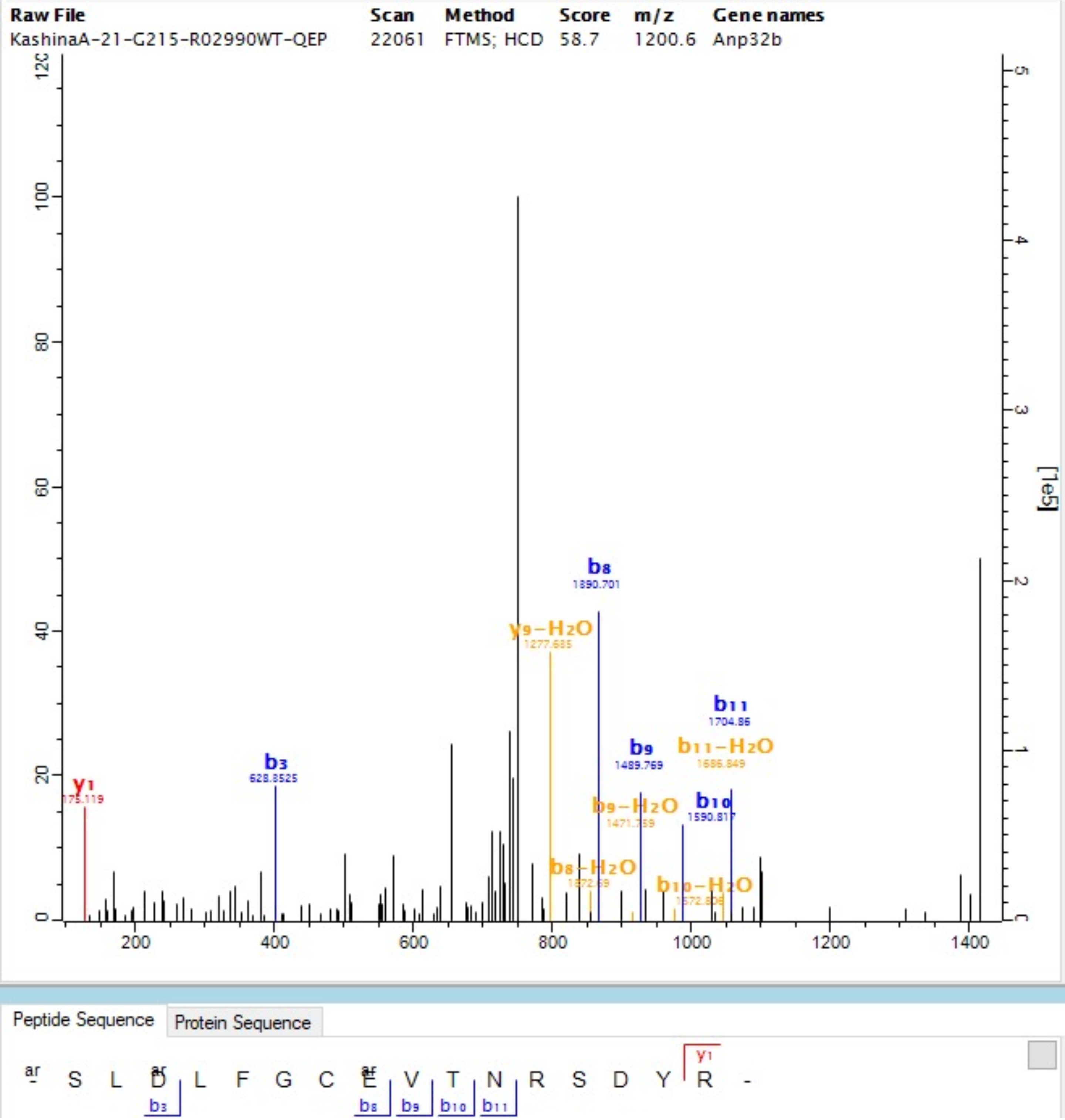

Raw File
Scan
Method
Score
m/z
Gene names
KashinaA-21-G215-R02990WT-QEP
22061
FTMS; HCD
58.7
1200.6
Anp32b
[1e5]
y1
175.119
b3
628.8525
y9-H2O
1277.685
b8
1890.701
b9
1489.769
b9-H2O
1471.759
b8-H2O
b10
1590.817
b10-H2O
b11
1704.86
b11-H2O
1686.849
Peptide Sequence
Protein Sequence
- S L D L F G C E V T N R S D Y R -
b3
b8
b9
b10
b11
y1

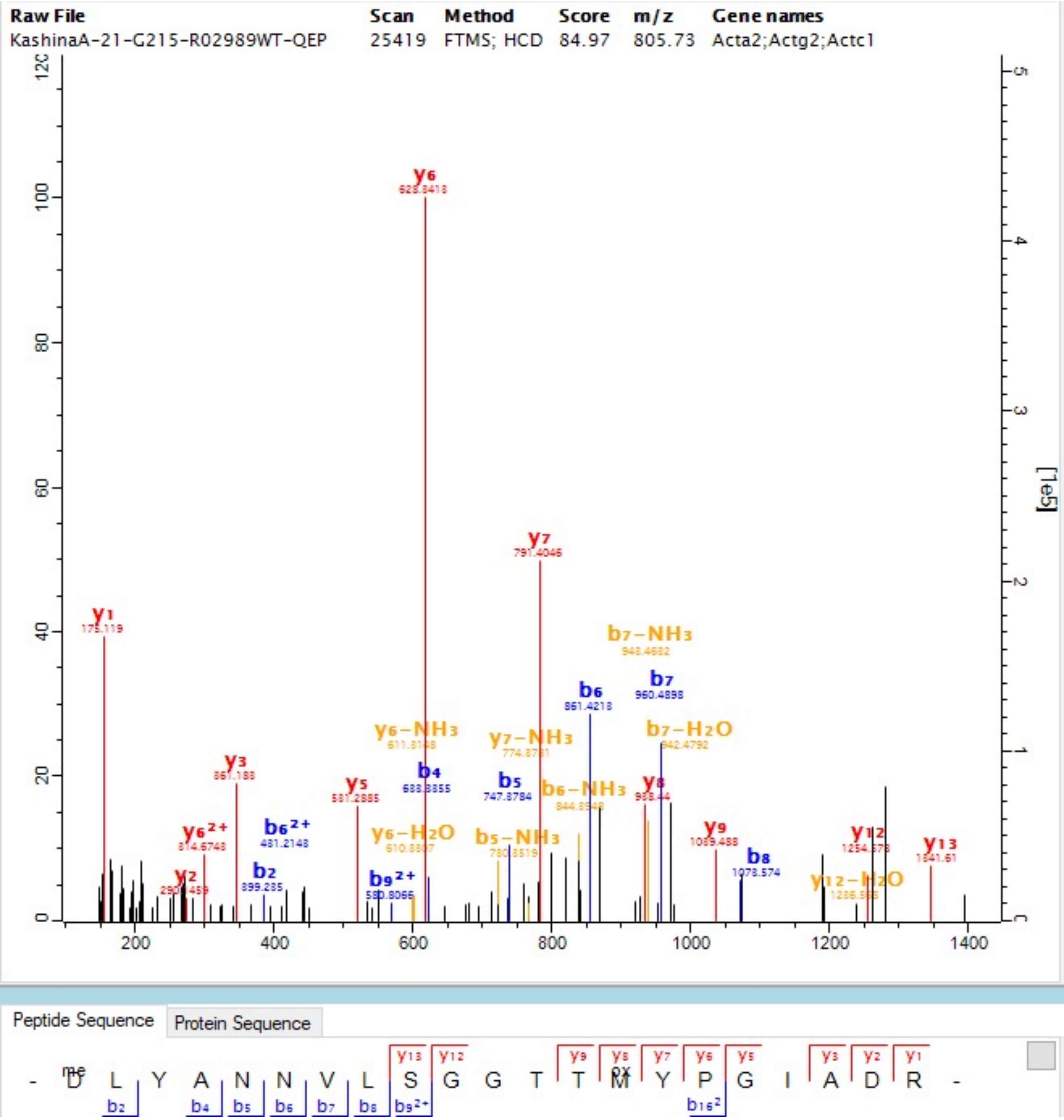

Raw File
KashinaA-21-G215-R02989WT-QEP
Scan
25419
Method
FTMS; HCD
Score
84.97
m/z
805.73
Gene names
Acta2;Actg2;Actc1
Peptide Sequence
Protein Sequence
- D L Y A N N V L S G G T T M Y P G I A D R -

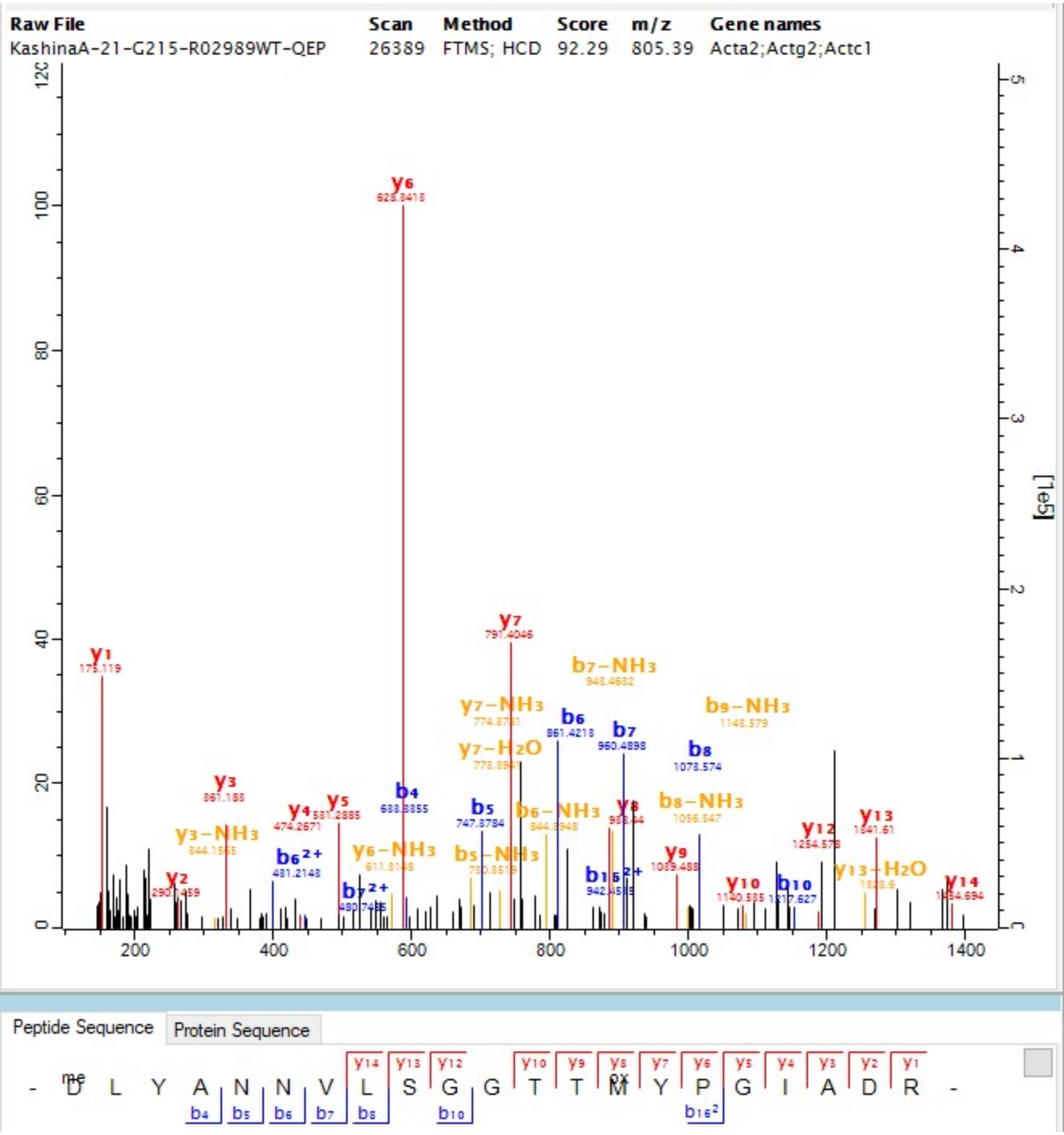

Raw File
KashinaA-21-G215-R02989WT-QEP
Scan
26389
Method
FTMS; HCD
Score
92.29
m/z
805.39
Gene names
Acta2;Actg2;Actc1
y6
y7
y1
y3
y4
y5
b4
b5
b6
b7
b8
y8
y9
y10
b10
y12
y13
y14
y2
b6²⁺
b7²⁺
b16²⁺
y3−NH3
y6−NH3
y7−NH3
y7−H2O
b5−NH3
b6−NH3
b7−NH3
b8−NH3
b9−NH3
y13−H2O
[1e5]
Peptide Sequence
Protein Sequence
- D L Y A N N V L S G G T T M Y P G I A D R -

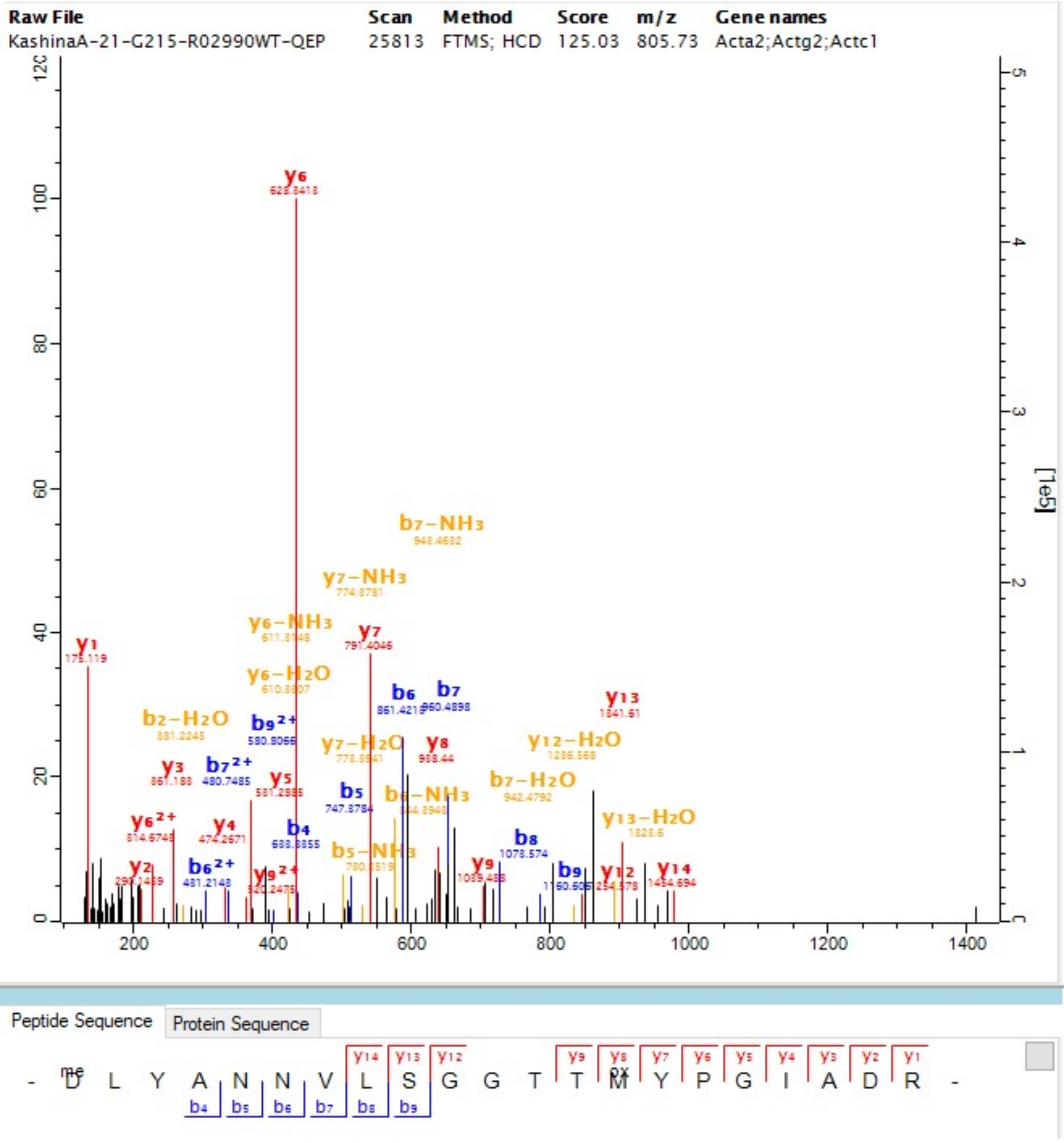
Raw File
KashinaA-21-G215-R02990WT-QEP
Scan
25813
Method
FTMS; HCD
Score
125.03
m/z
805.73
Gene names
Acta2;Actg2;Actc1
[1e5]
Peptide Sequence
Protein Sequence
- D L Y A N N V L S G G T T M Y P G I A D R -
me
ox

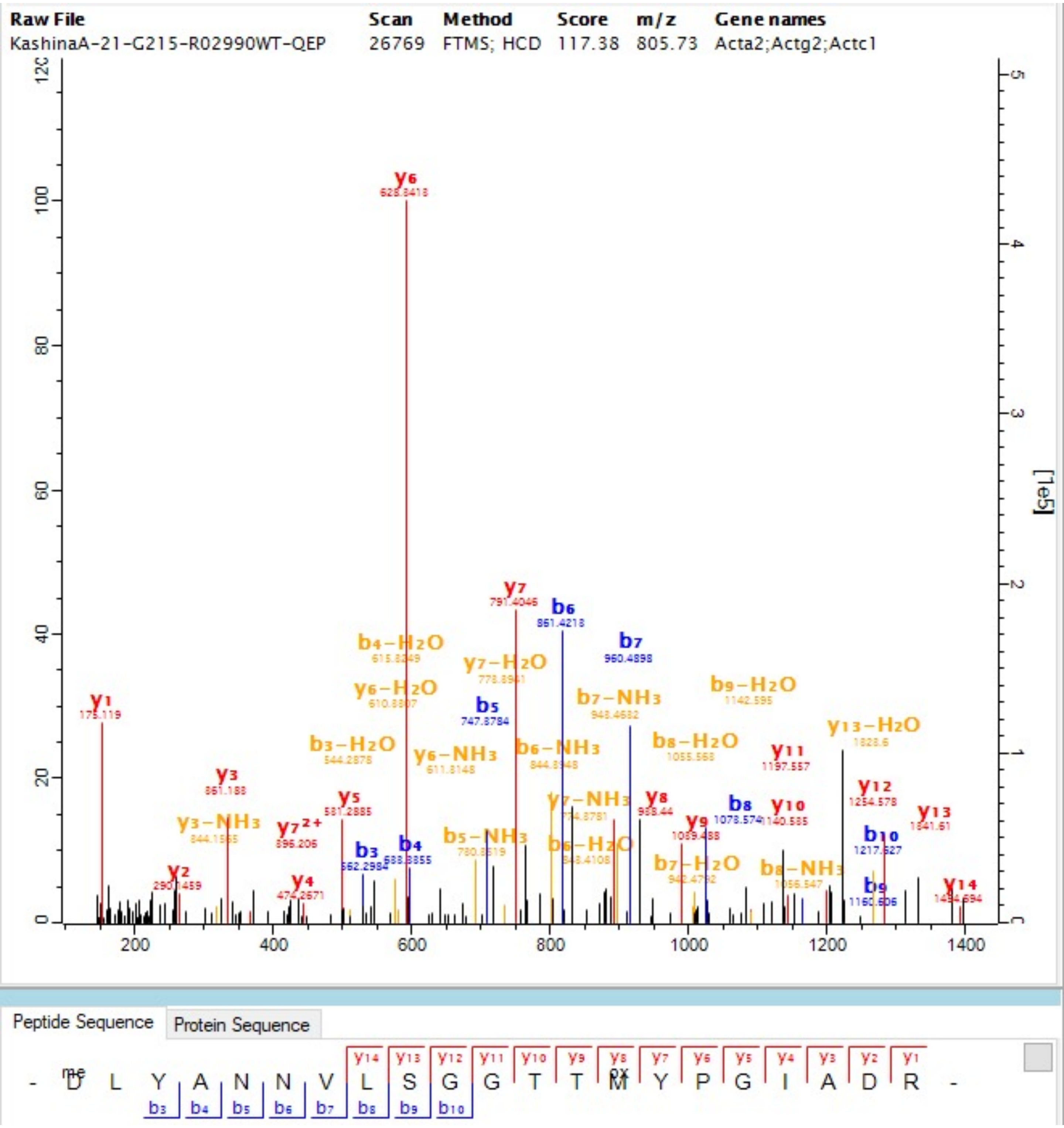

Raw File
KashinaA-21-G215-R02990WT-QEP
Scan
26769
Method
FTMS; HCD
Score
117.38
m/z
805.73
Gene names
Acta2;Actg2;Actc1
[1e5]
y6
y7
b6
b7
b4-H2O
y7-H2O
y6-H2O
b5
b9-H2O
y1
y13-H2O
b7-NH3
b3-H2O
y6-NH3
b6-NH3
b8-H2O
y11
y3
y12
y7-NH3
y5
y8
b8
y10
y3-NH3
y7 2+
y9
y13
b3
b4
b5-NH3
b10
b6-H2O
b7-H2O
y2
b8-NH3
y4
b9
y14
200
400
600
800
1000
1200
1400
Peptide Sequence
Protein Sequence
- D L Y A N N V L S G G T T M Y P G I A D R -
me
ox
y14 y13 y12 y11 y10 y9 y8 y7 y6 y5 y4 y3 y2 y1
b3 b4 b5 b6 b7 b8 b9 b10

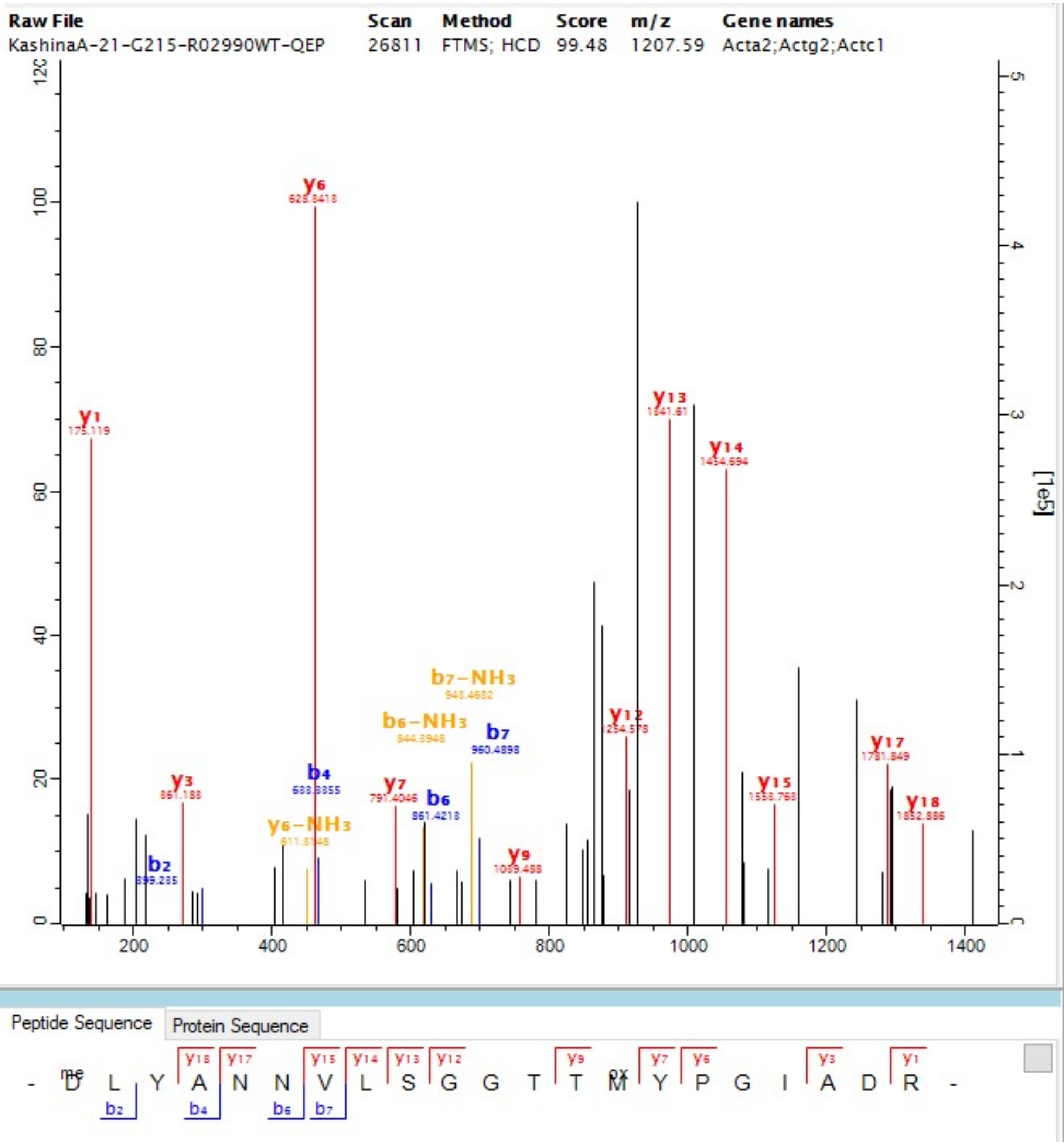
Raw File
KashinaA-21-G215-R02990WT-QEP
Scan
26811
Method
FTMS; HCD
Score
99.48
m/z
1207.59
Gene names
Acta2;Actg2;Actc1
[1e5]
y1
y3
b2
b4
y6
y6-NH3
y7
b6
b6-NH3
b7-NH3
b7
960.4898
y9
y12
y13
y14
y15
y17
y18
Peptide Sequence
Protein Sequence
- D L Y A N N V L S G G T T M Y P G I A D R -
me
ox

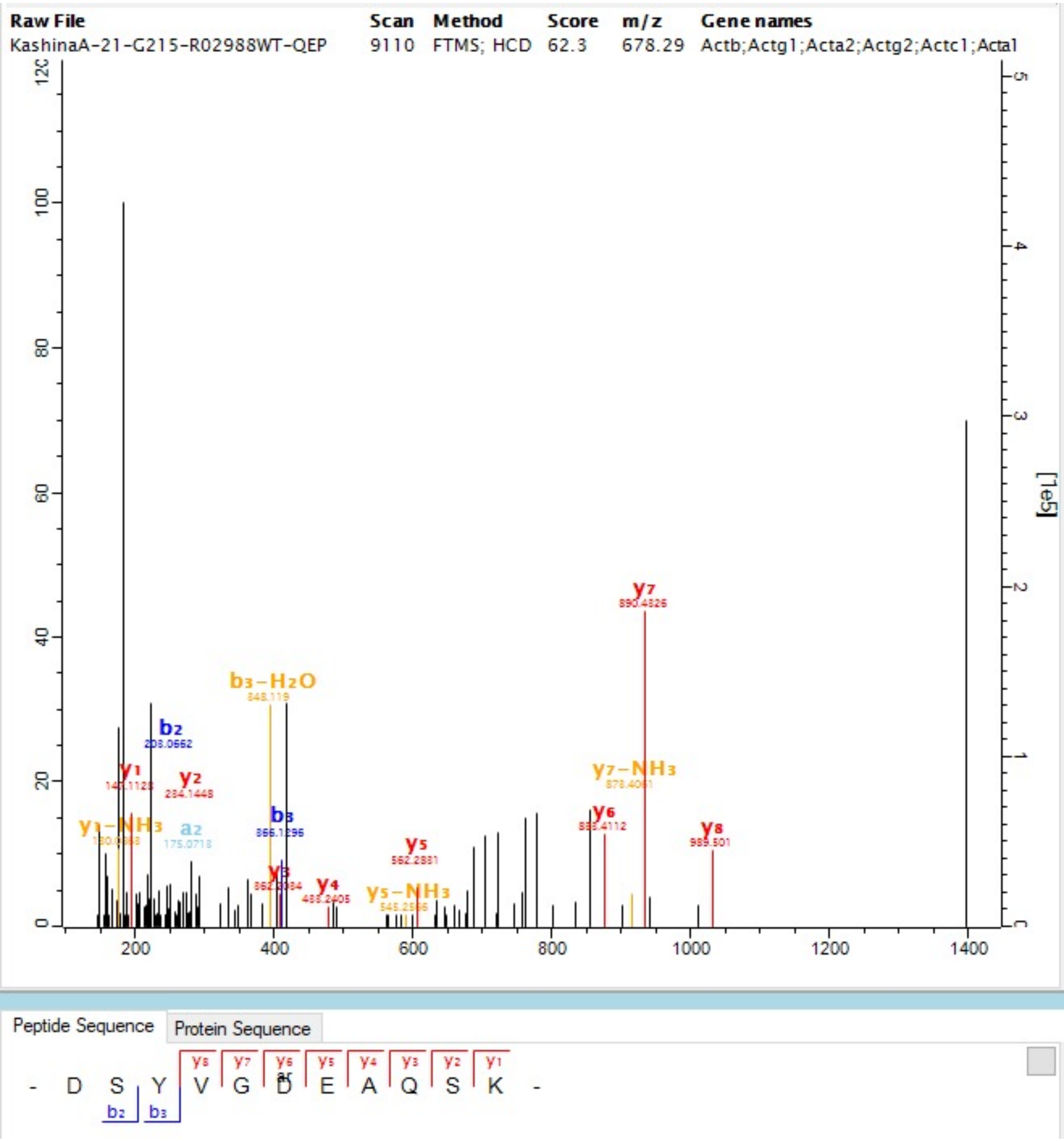

Raw File
KashinaA-21-G215-R02988WT-QEP
Scan
9110
Method
FTMS; HCD
Score
62.3
m/z
678.29
Gene names
Actb;Actg1;Acta2;Actg2;Actc1;Acta1
[1e5]
y7
890.4326
b3-H2O
b2
y1
y2
284.1448
y7-NH3
y6
y1-NH3
a2
175.0718
b3
y8
989.501
y5
562.2881
y3
y4
y5-NH3
Peptide Sequence
Protein Sequence
- D S Y V G D E A Q S K -

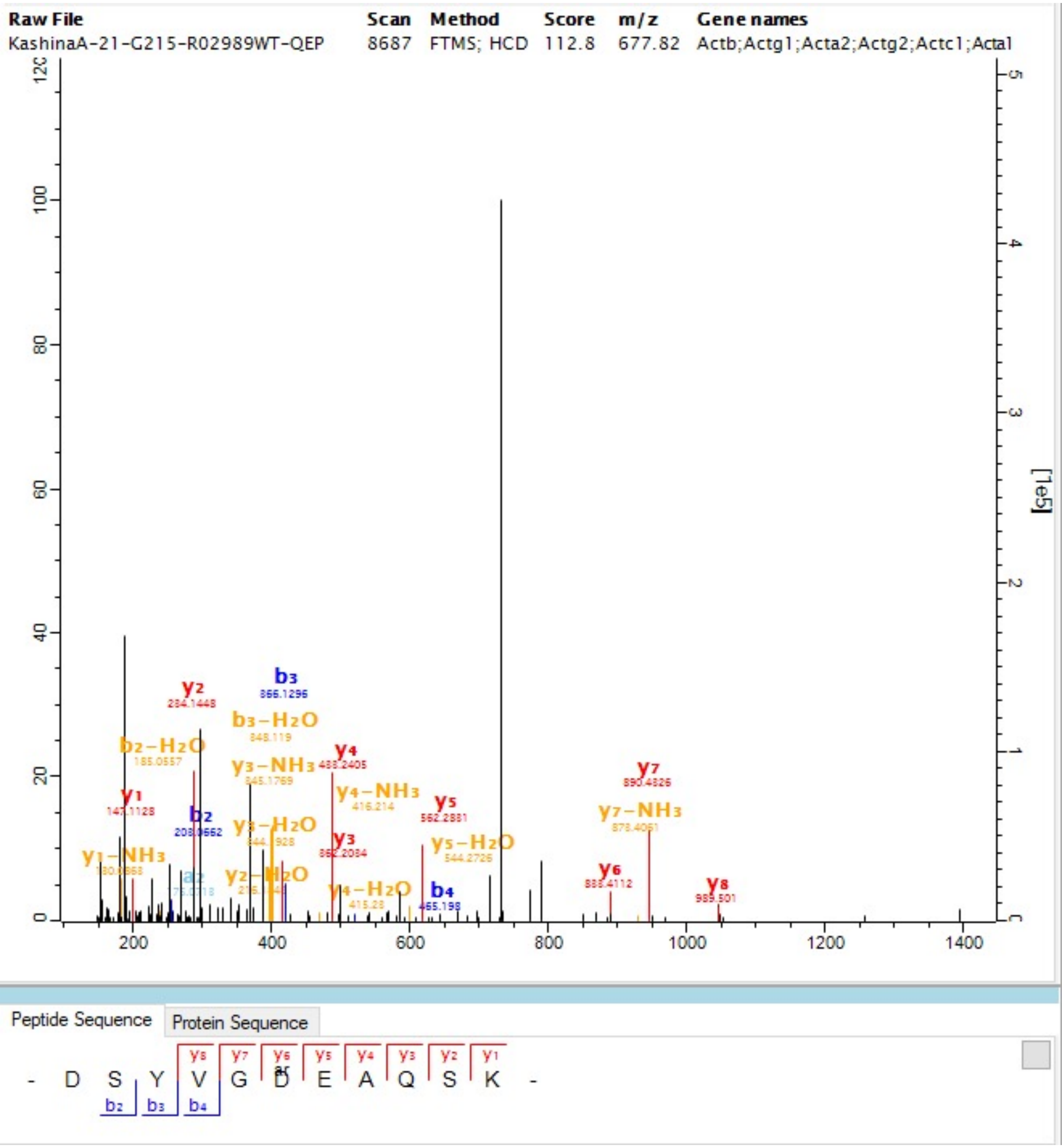

Raw File
KashinaA-21-G215-R02989WT-QEP
Scan
8687
Method
FTMS; HCD
Score
112.8
m/z
677.82
Gene names
Actb;Actg1;Acta2;Actg2;Actc1;Acta1
[1e5]
y1
y2
b2
b3
b4
y3
y4
y5
y6
y7
y8
b2−H2O
b3−H2O
y1−NH3
y3−NH3
y4−NH3
y7−NH3
y3−H2O
y2−H2O
y4−H2O
y5−H2O
a2
Peptide Sequence
Protein Sequence
- D S Y V G D E A Q S K -

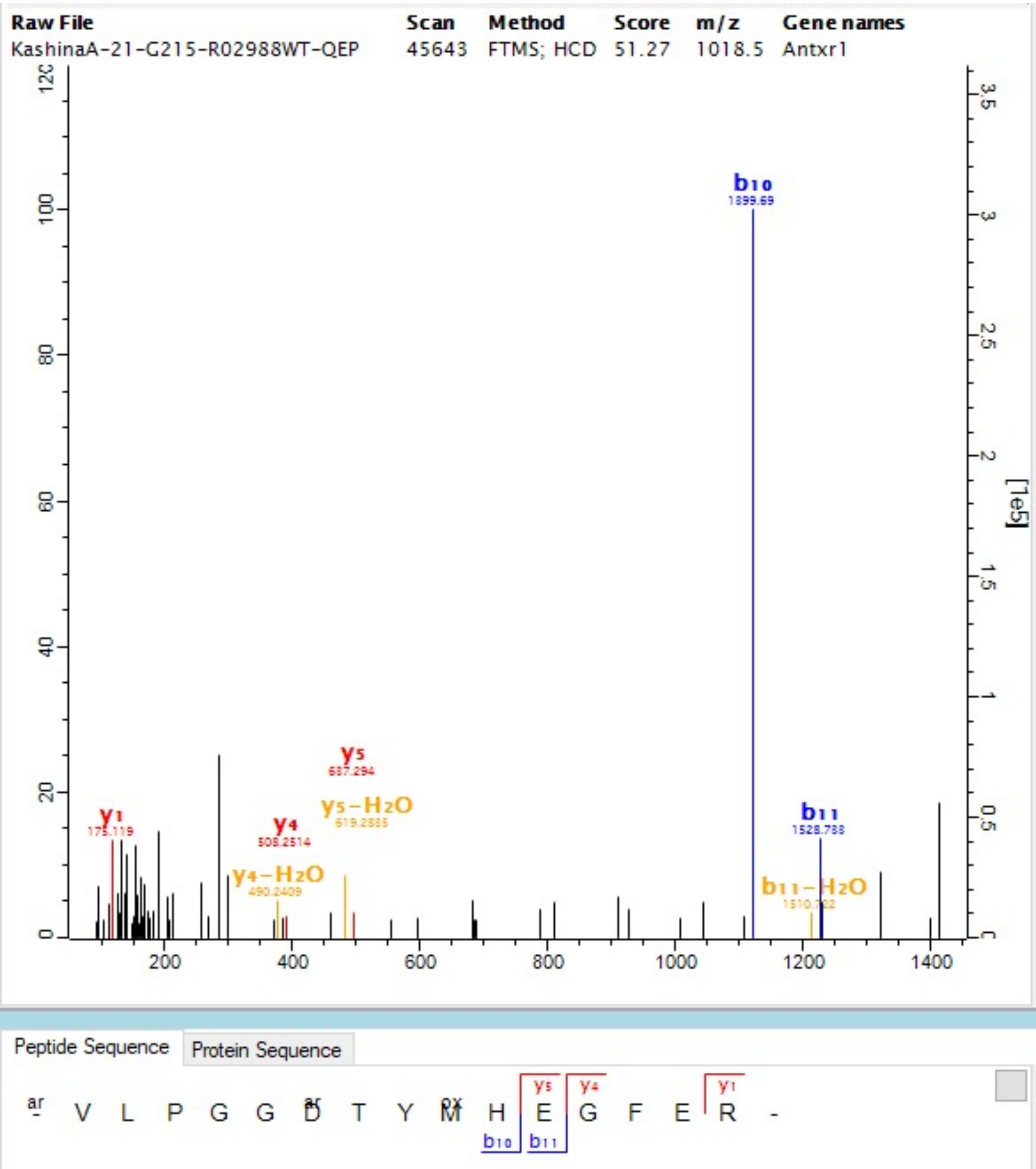

Raw File
KashinaA-21-G215-R02988WT-QEP
Scan
45643
Method
FTMS; HCD
Score
51.27
m/z
1018.5
Gene names
Antxr1
b10
1399.69
b11
1528.788
b11-H2O
y1
175.119
y4
508.2514
y4-H2O
490.2409
y5
637.294
y5-H2O
619.2835
[1e5]
Peptide Sequence
Protein Sequence
- V L P G G D T Y M H E G F E R -

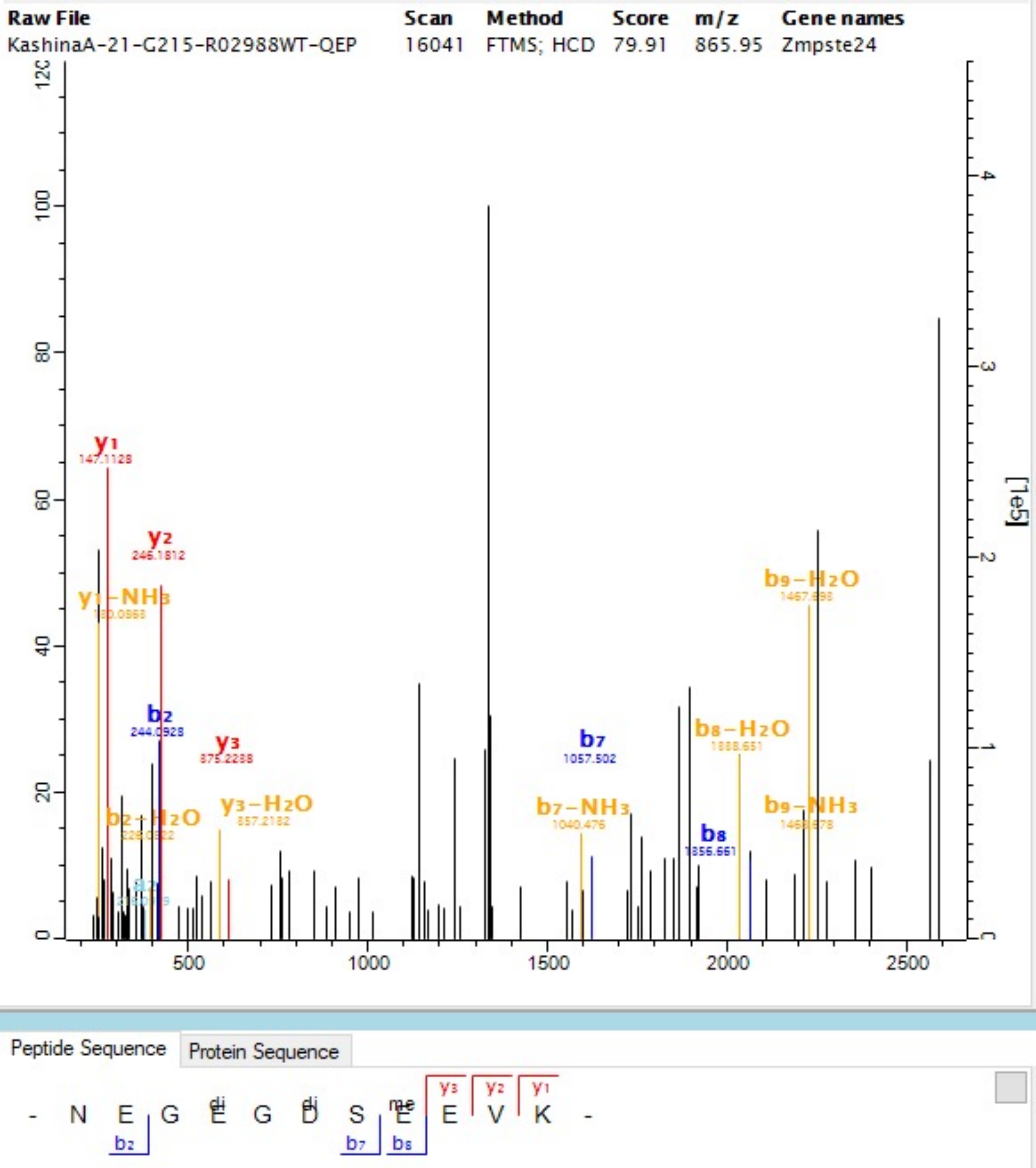
Raw File
KashinaA-21-G215-R02988WT-QEP
Scan
16041
Method
FTMS; HCD
Score
79.91
m/z
865.95
Gene names
Zmpste24
y1
147.1128
y2
246.1812
y1-NH3
b2
244.0928
y3
875.2288
b2-H2O
y3-H2O
857.2182
b7
1057.502
b7-NH3
1040.476
b8
1856.661
b8-H2O
1888.651
b9-H2O
b9-NH3
[1e5]
Peptide Sequence
Protein Sequence
- N E G E G D S E E V K -
di
di
me
b2
b7
b8
y3
y2
y1

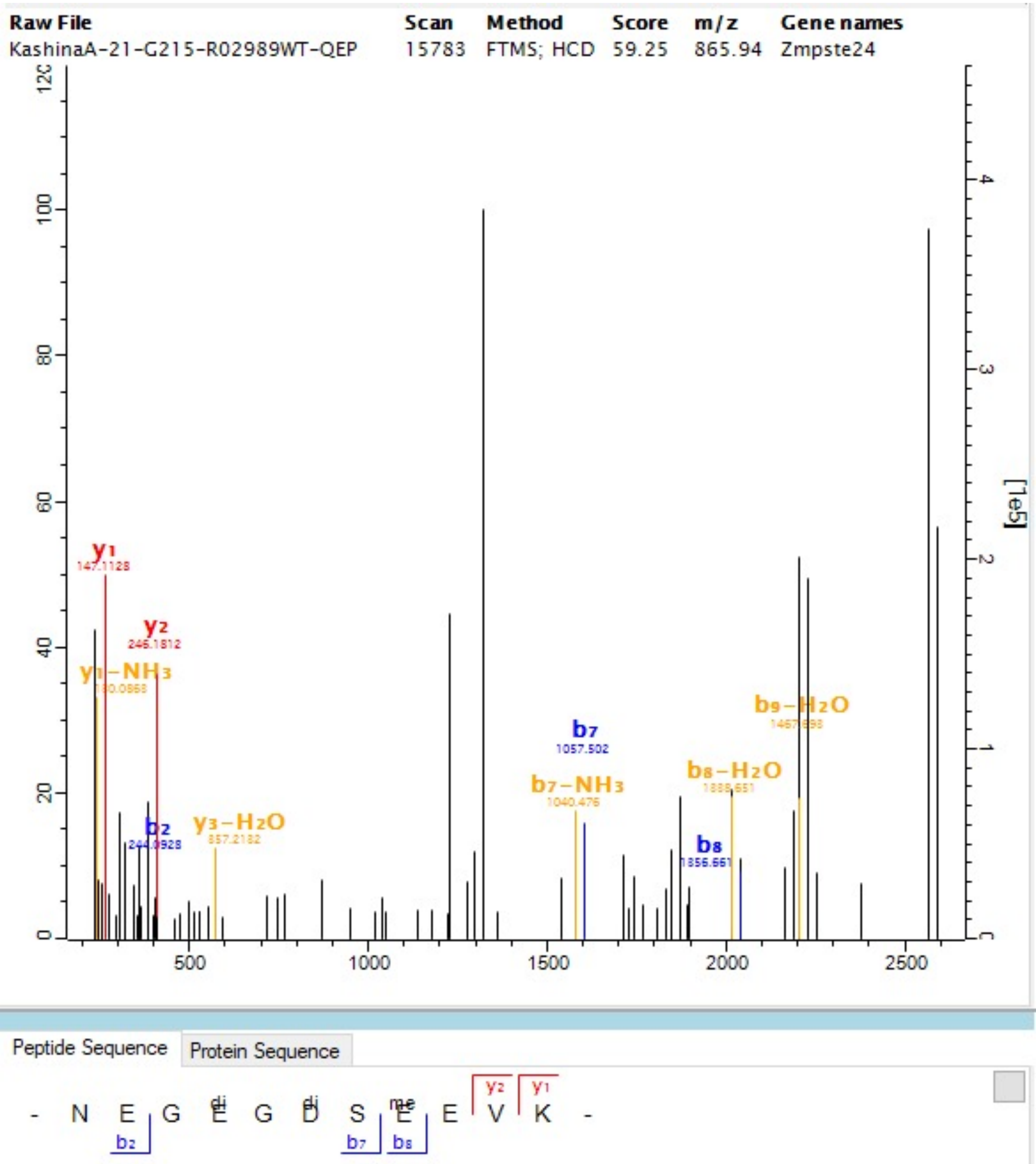
Raw File
KashinaA-21-G215-R02989WT-QEP
Scan
15783
Method
FTMS; HCD
Score
59.25
m/z
865.94
Gene names
Zmpste24
[1e5]
Peptide Sequence
Protein Sequence
- N E G E G D S E E V K -

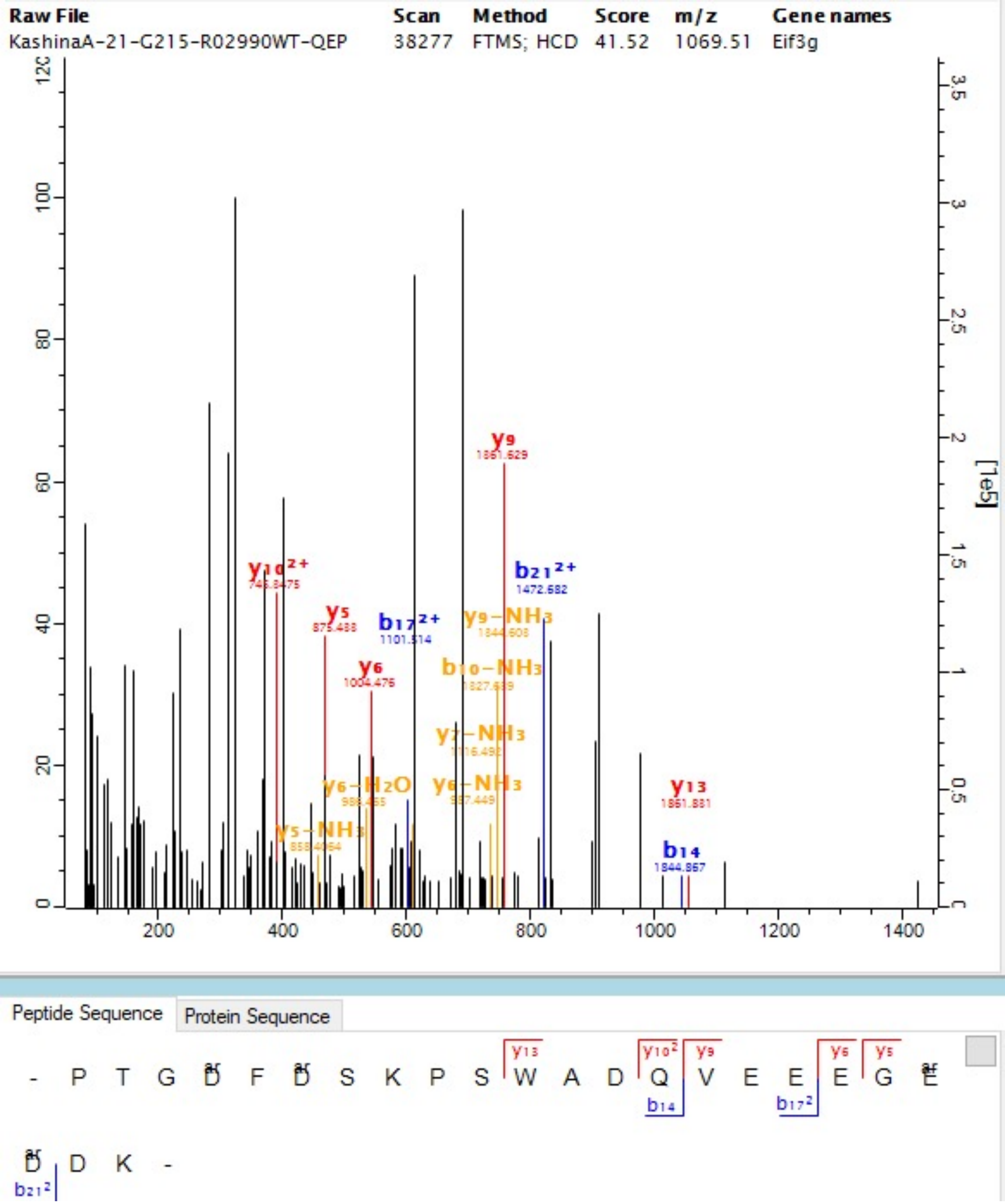
Raw File
KashinaA-21-G215-R02990WT-QEP
Scan
38277
Method
FTMS; HCD
Score
41.52
m/z
1069.51
Gene names
Eif3g
y9
y10 2+
y5
875.488
b17 2+
1101.514
y9-NH3
1844.608
b21 2+
1472.682
y6
1004.476
b10-NH3
y7-NH3
y6-H2O
y6-NH3
y5-NH3
y13
b14
1844.857
[1e5]
Peptide Sequence
Protein Sequence
- P T G D F D S K P S W A D Q V E E E G E
D D K -

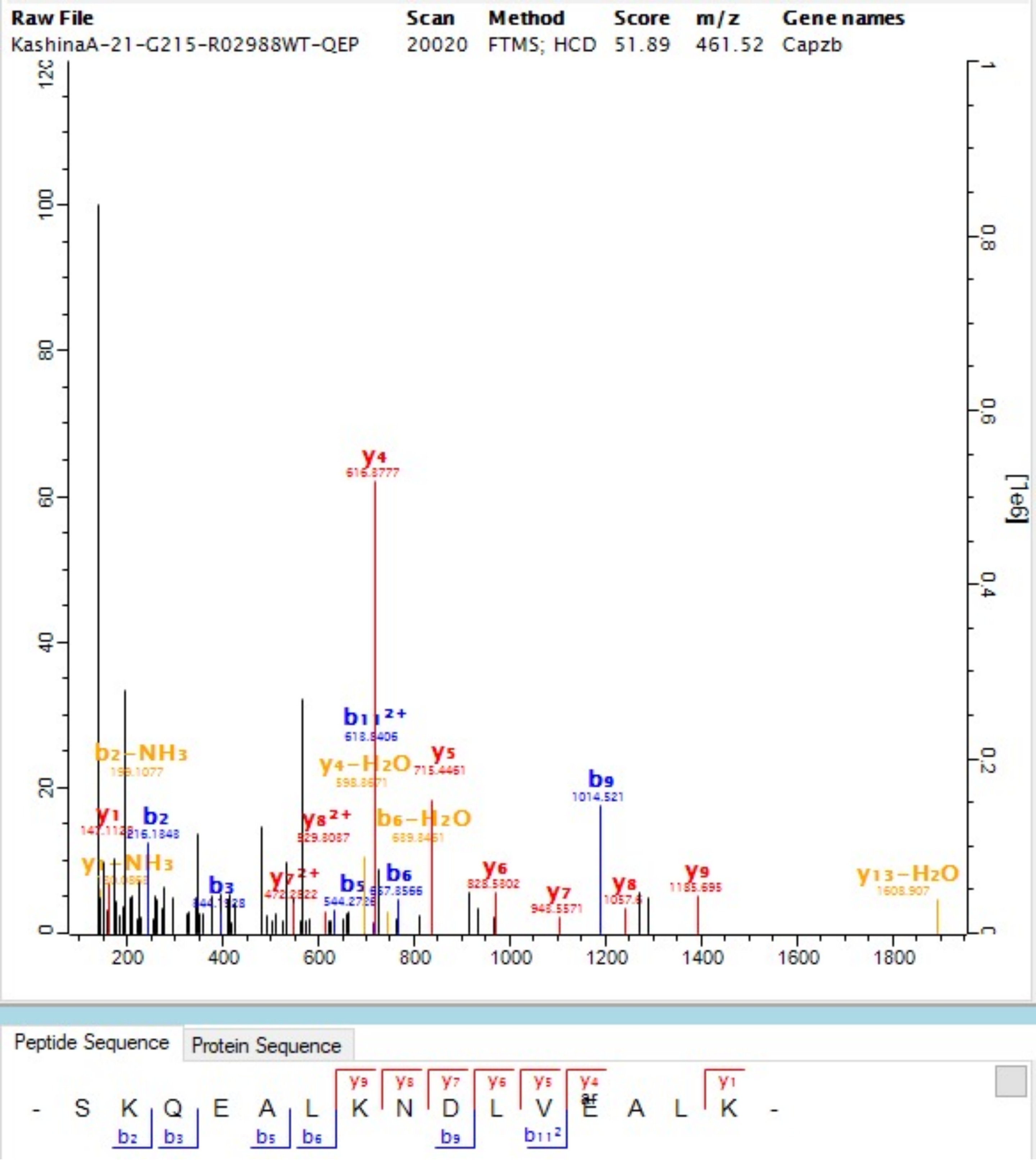

Raw File
KashinaA-21-G215-R02988WT-QEP
Scan
20020
Method
FTMS; HCD
Score
51.89
m/z
461.52
Gene names
Capzb
[1e6]
y4
b11 2+
b2-NH3
y4-H2O
y5
715.4461
b9
1014.521
y1
b2
216.1848
y8 2+
529.8087
b6-H2O
y1-NH3
b3
y7 2+
b5
b6
y6
828.5302
y7
943.5571
y8
y9
1185.695
y13-H2O
1608.907
Peptide Sequence
Protein Sequence
- S K Q E A L K N D L V E A L K -
y9 y8 y7 y6 y5 y4 y1
b2 b3 b5 b6 b9 b11 2

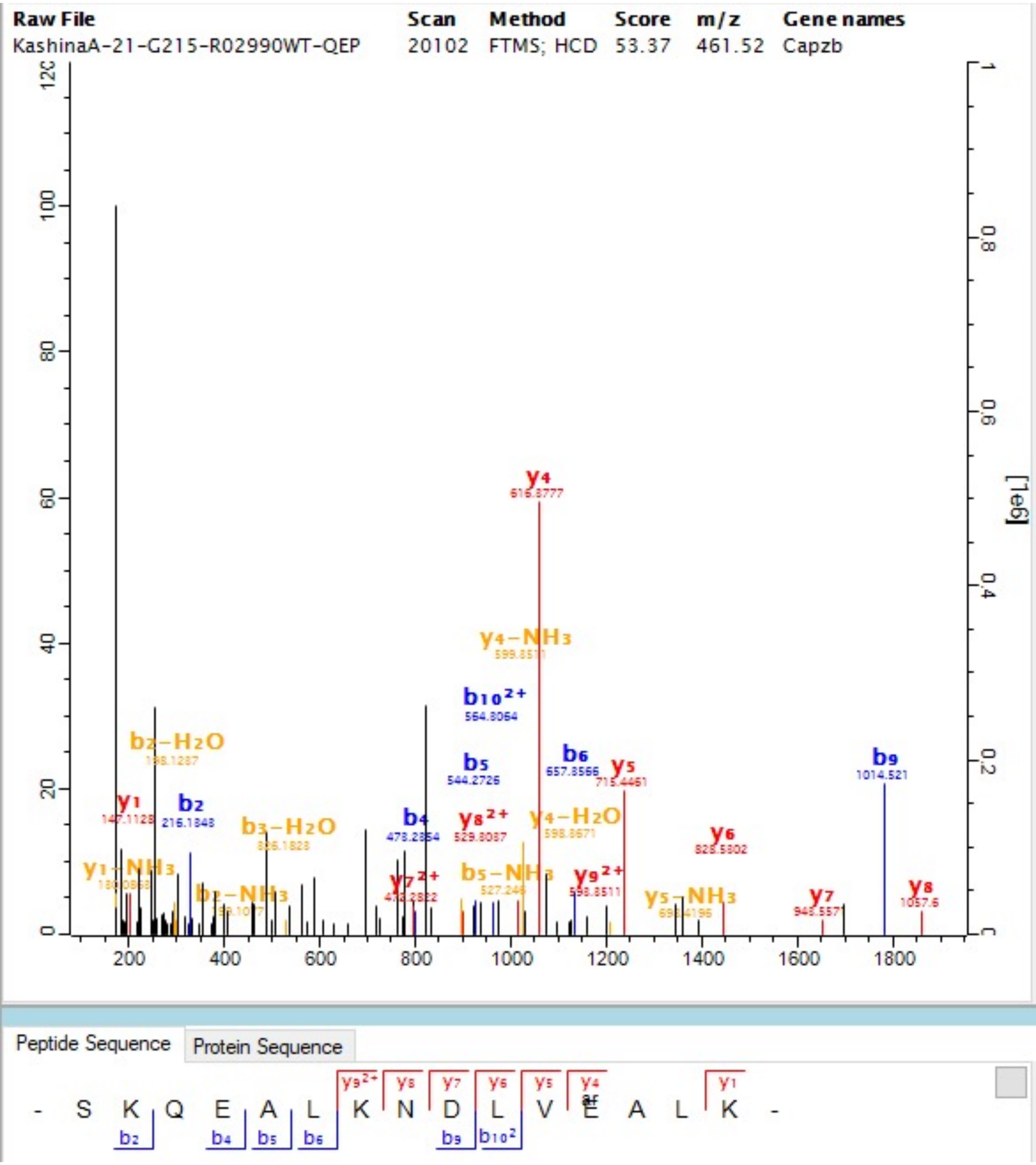
Raw File
KashinaA-21-G215-R02990WT-QEP
Scan
20102
Method
FTMS; HCD
Score
53.37
m/z
461.52
Gene names
Capzb
y4
y4-NH3
b10 2+
b2-H2O
b5
b6
y5
b9
y1
b2
b3-H2O
b4
y8 2+
y4-H2O
y6
y1-NH3
b5-NH3
y7 2+
y9 2+
b2-NH3
y5-NH3
y7
y8
[1e6]
Peptide Sequence
Protein Sequence
- S K Q E A L K N D L V E A L K -

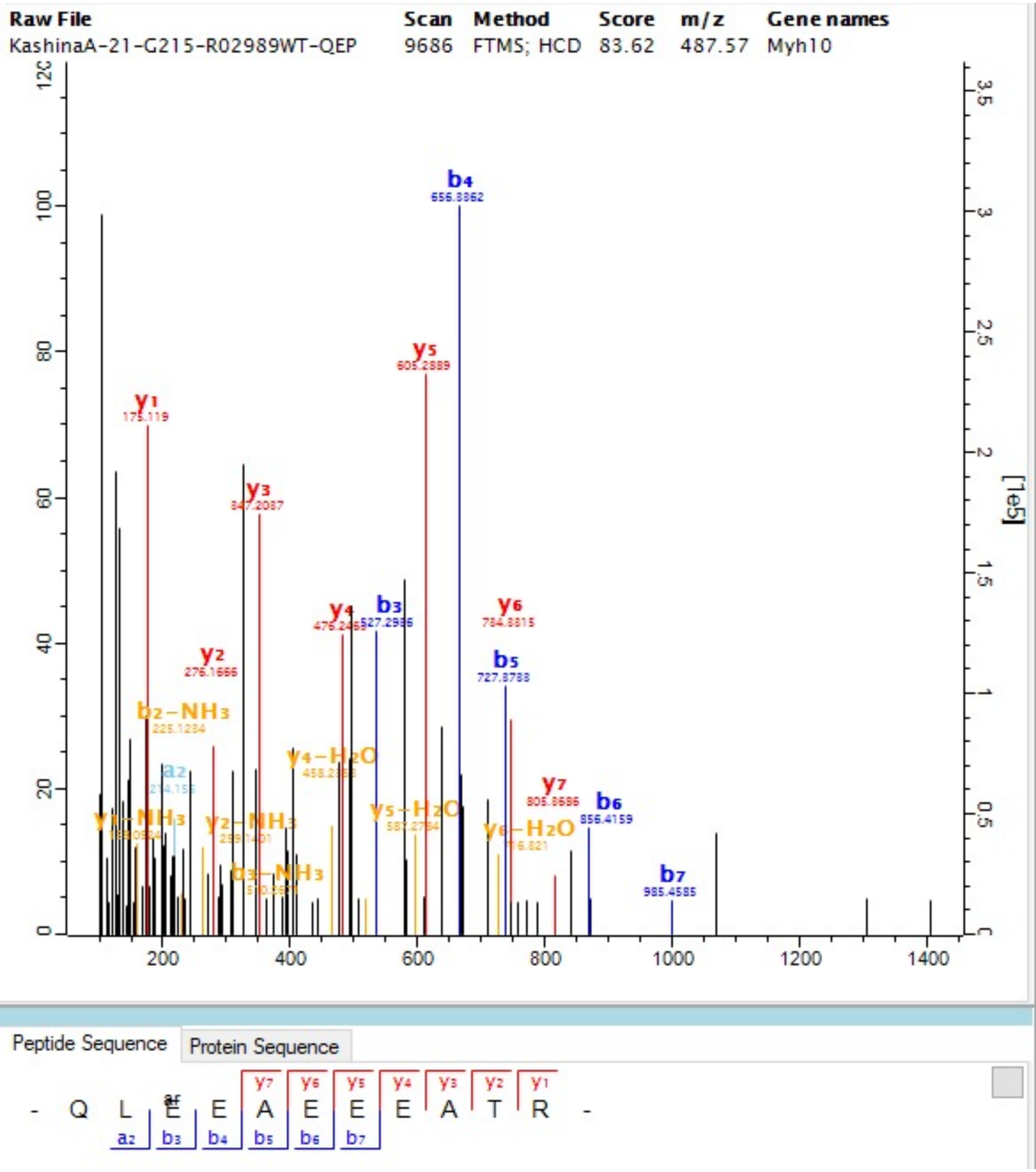
Raw File
KashinaA-21-G215-R02989WT-QEP
Scan
9686
Method
FTMS; HCD
Score
83.62
m/z
487.57
Gene names
Myh10
b4
656.3362
y5
605.2889
y1
175.119
y3
347.2087
y4
b3
527.2986
y6
784.3815
y2
276.1666
b5
727.3788
b2-NH3
225.1284
a2
214.155
y4-H2O
y7
805.3686
b6
856.4159
y5-H2O
y6-H2O
b3-NH3
b7
985.4585
[1e5]
Peptide Sequence
Protein Sequence
- Q L E E A E E E A T R -

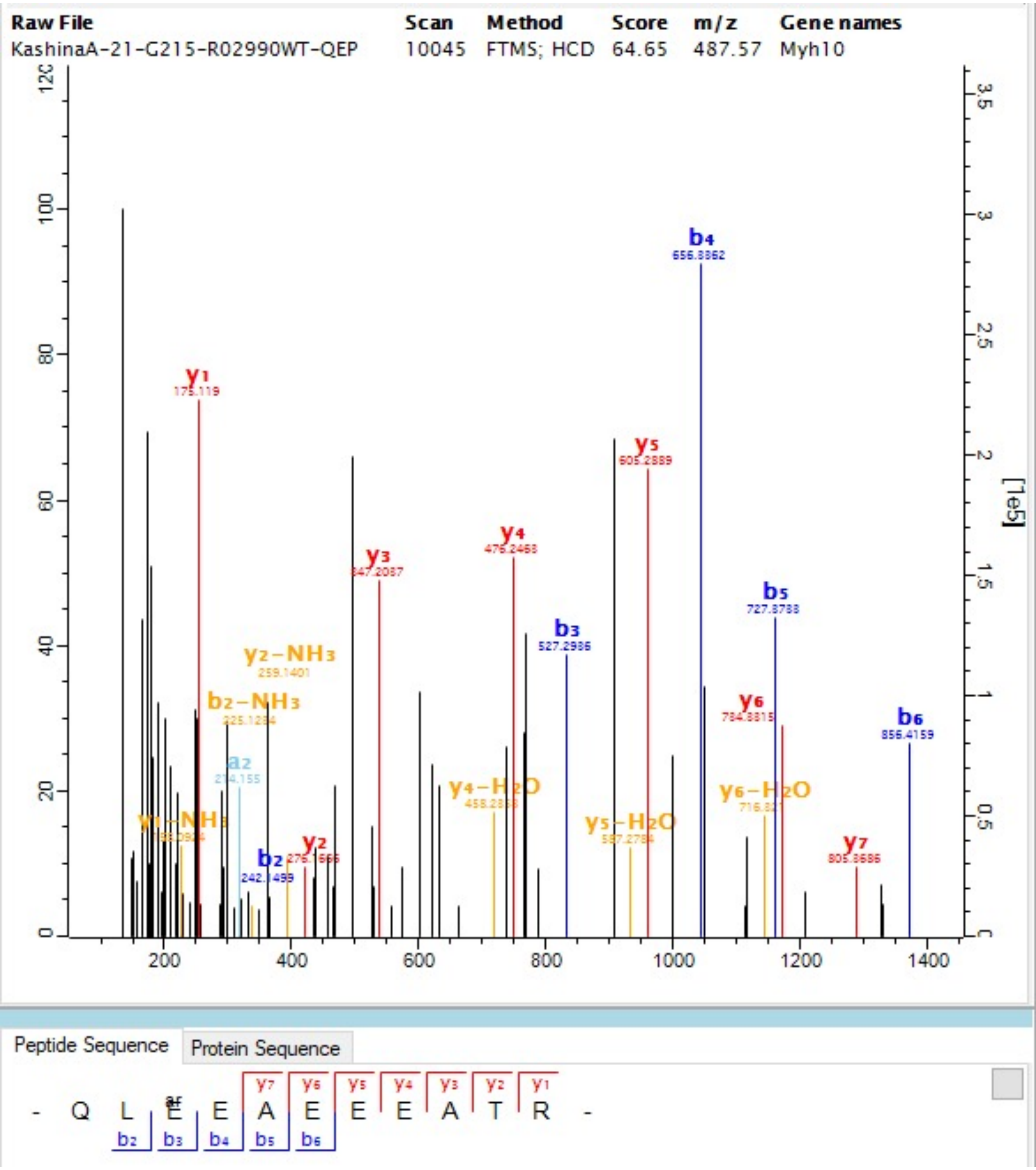

Raw File
KashinaA-21-G215-R02990WT-QEP
Scan
10045
Method
FTMS; HCD
Score
64.65
m/z
487.57
Gene names
Myh10
[1e5]
y1 175.119
y2-NH3 259.1401
b2-NH3 225.1284
a2 214.155
b2 242.1499
y2 276.1666
y3 347.2067
y4 476.2468
y4-H2O 458.2358
b3 527.2986
y5 605.2889
y5-H2O 587.2784
b4 656.3362
b5 727.3788
y6 734.3315
y6-H2O 716.321
y7 805.3686
b6 856.4159
Peptide Sequence
Protein Sequence
- Q L E E A E E E A T R -

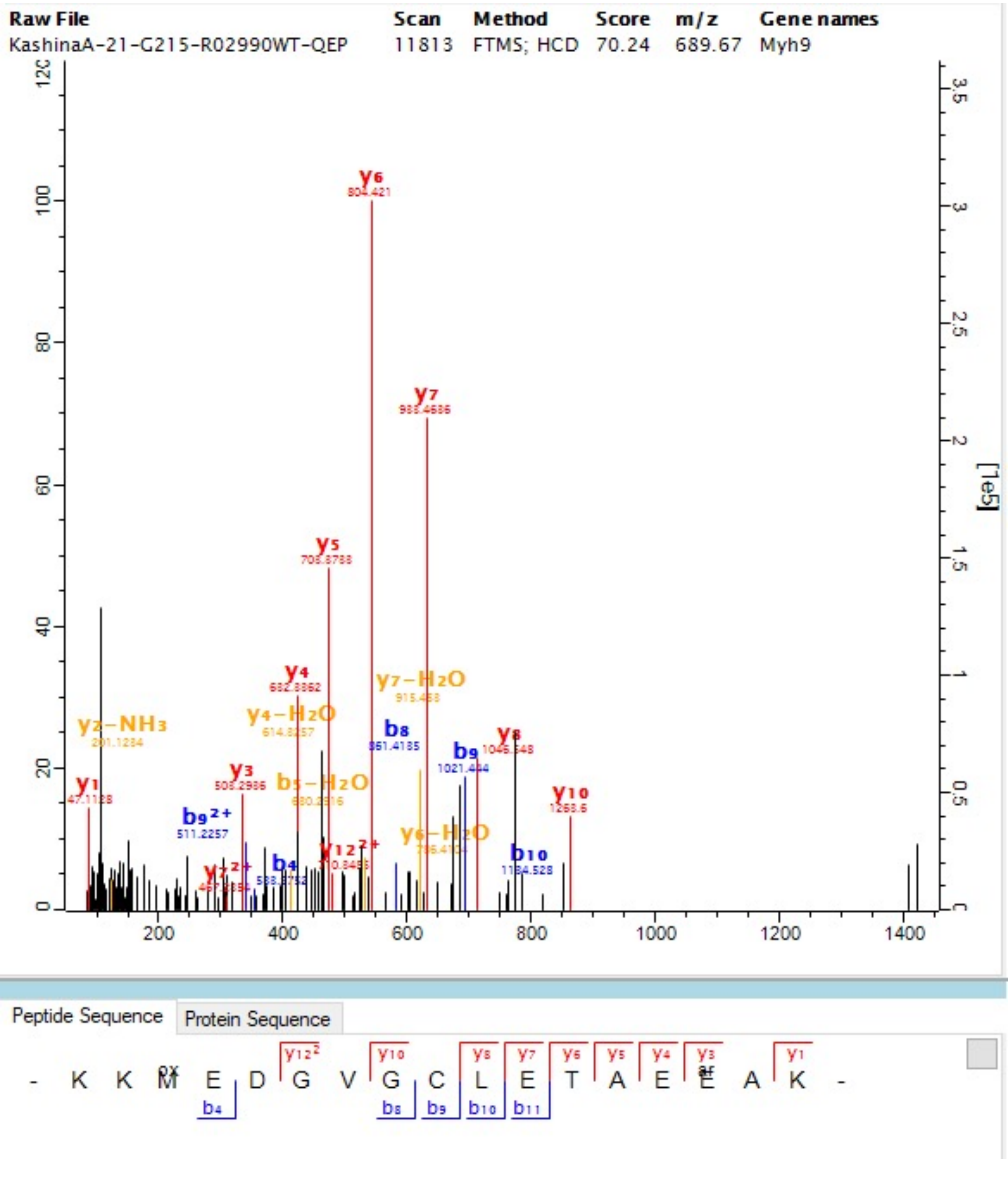

Raw File
KashinaA-21-G215-R02990WT-QEP
Scan
11813
Method
FTMS; HCD
Score
70.24
m/z
689.67
Gene names
Myh9
y6
804.421
y7
y5
y4
y7−H2O
915.458
y2−NH3
201.1234
y4−H2O
b8
b9
1021.444
y8
y3
y10
y1
b5−H2O
b9 2+
511.2257
y6−H2O
b10
y12 2+
y7 2+
b4
0
20
40
60
80
100
200
400
600
800
1000
1200
1400
[1e5]
Peptide Sequence
Protein Sequence
- K K M E D G V G C L E T A E E A K -

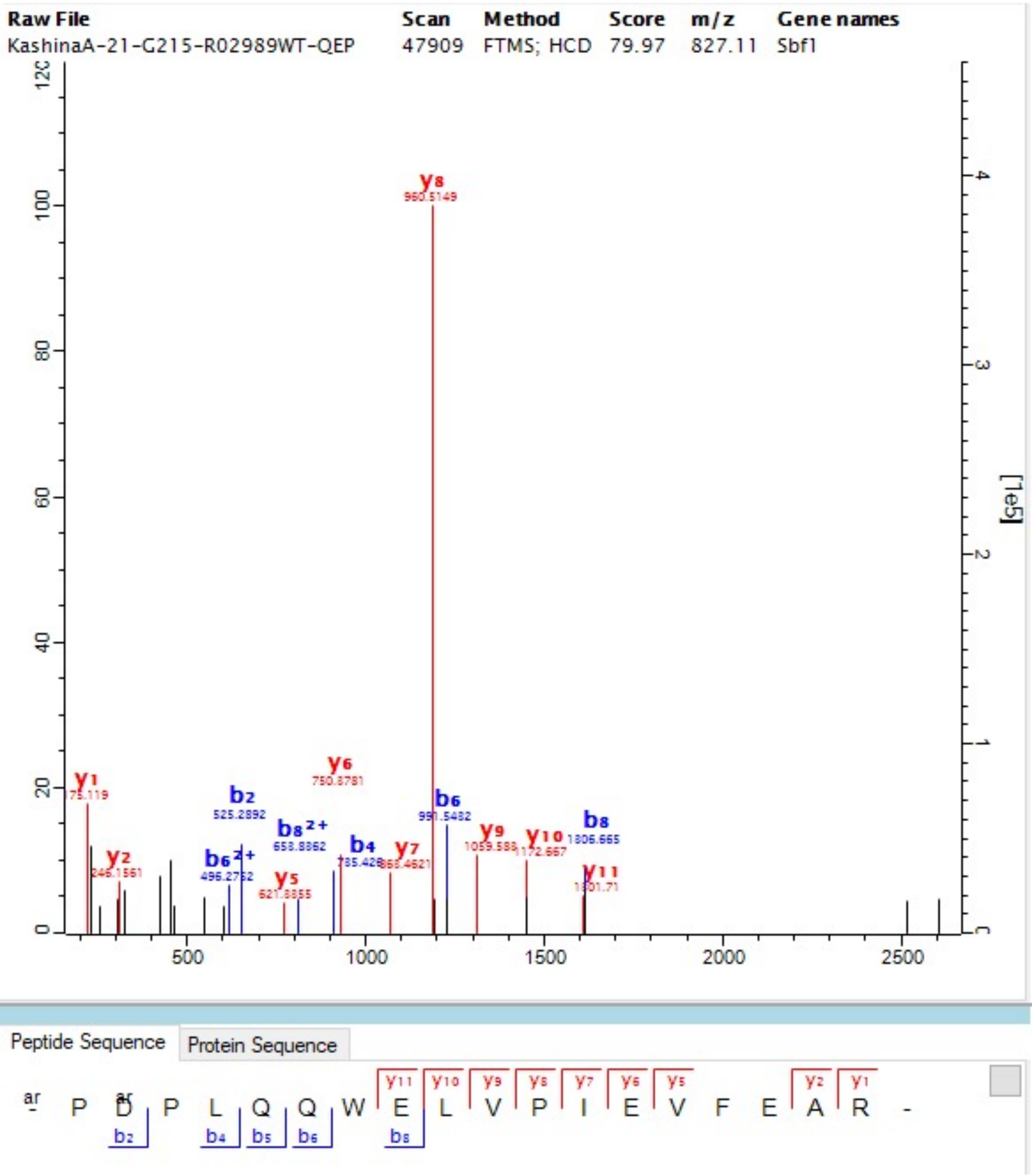
Raw File
KashinaA-21-G215-R02989WT-QEP
Scan
47909
Method
FTMS; HCD
Score
79.97
m/z
827.11
Gene names
Sbf1
Peptide Sequence
Protein Sequence
P D P L Q Q W E L V P I E V F E A R

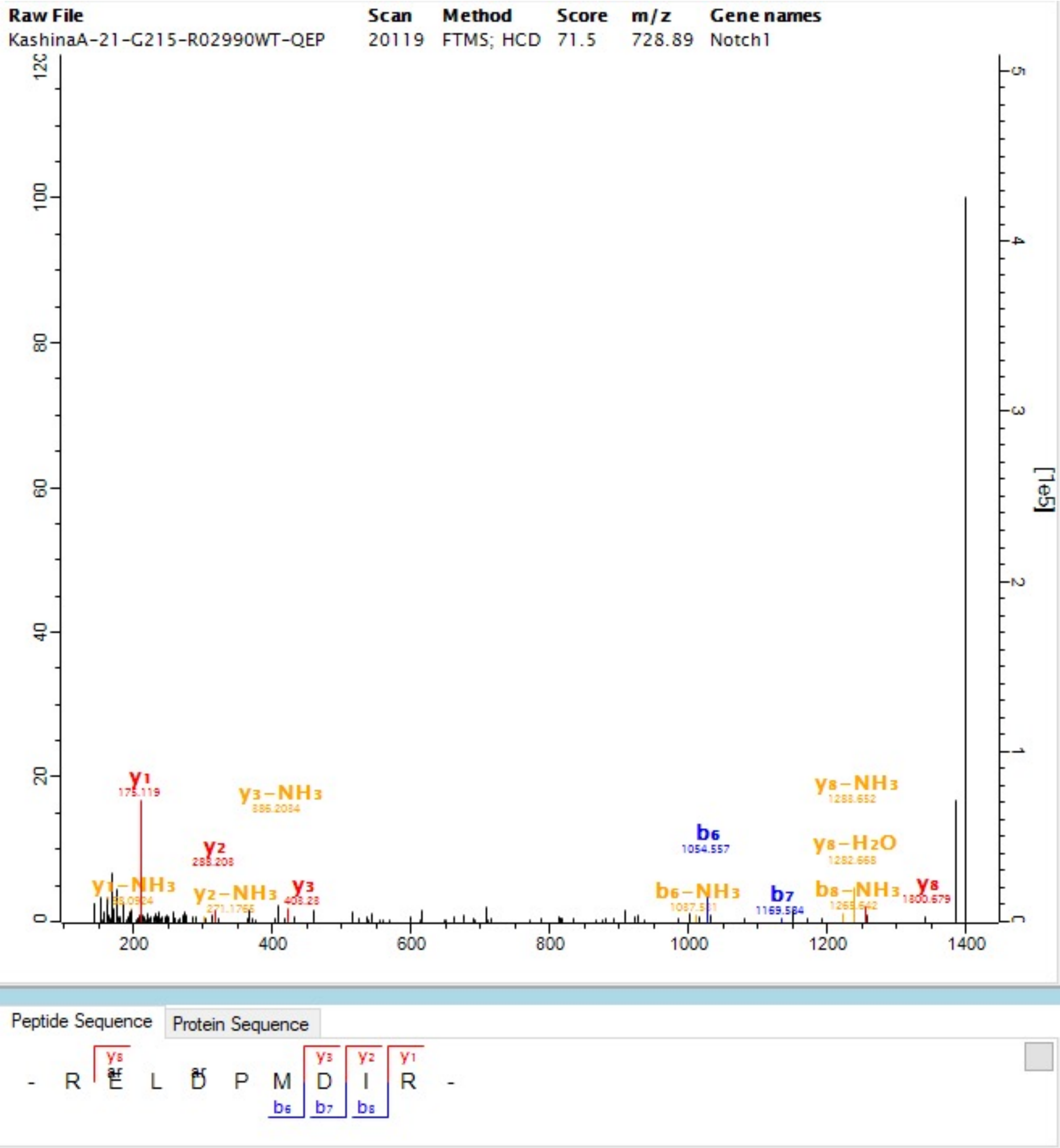

Raw File
KashinaA-21-G215-R02990WT-QEP
Scan
20119
Method
FTMS; HCD
Score
71.5
m/z
728.89
Gene names
Notch1
[1e5]
y1
y3-NH3
y2
y1-NH3
y2-NH3
y3
b6
1054.557
b6-NH3
b7
y8-NH3
1283.652
y8-H2O
1282.668
b8-NH3
y8
1300.679
Peptide Sequence
Protein Sequence
- R E L D P M D I R -
y8
y3
y2
y1
b6
b7
b8

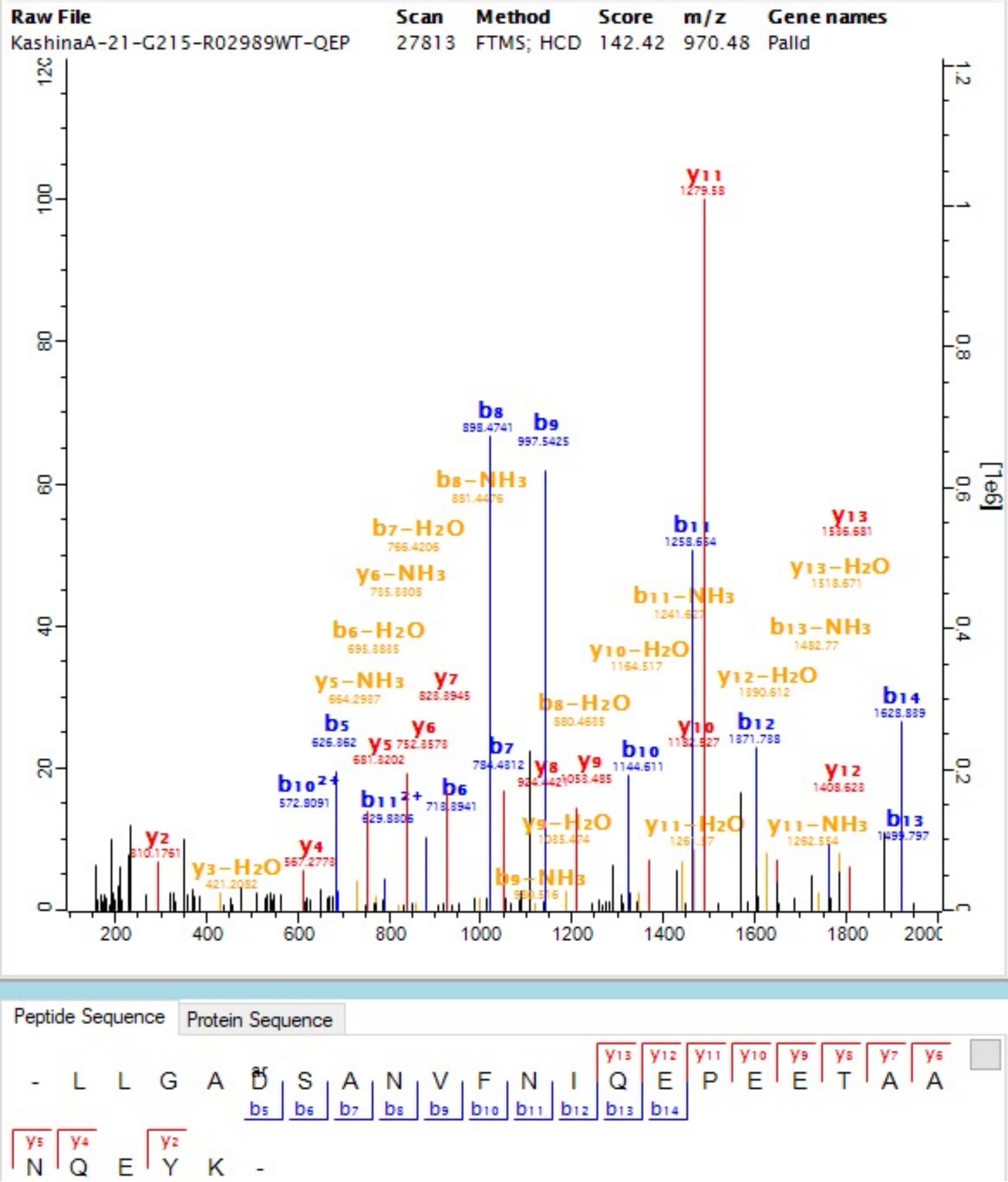

Raw File
KashinaA-21-G215-R02989WT-QEP
Scan
27813
Method
FTMS; HCD
Score
142.42
m/z
970.48
Gene names
Palld
[1e6]
y11
1279.58
b8
898.4741
b9
997.5425
b8-NH3
881.4476
b7-H2O
766.4206
y6-NH3
735.8808
b6-H2O
695.8835
y5-NH3
664.2937
y7
828.8945
b11
1258.664
y13
1536.681
y13-H2O
1518.671
b11-NH3
1241.627
b13-NH3
1482.77
y10-H2O
1164.517
y12-H2O
1390.612
b8-H2O
880.4635
b14
1628.889
b5
626.862
y6
752.8578
y5
681.8202
b7
784.4812
y8
924.4421
y9
1053.485
b10
1144.611
y10
1182.527
b12
1371.788
y12
1408.623
b10 2+
572.8091
b11 2+
629.8806
b6
718.8941
y9-H2O
1035.474
y11-H2O
1261.57
y11-NH3
1262.554
b13
1499.797
y2
310.1761
y3-H2O
421.2082
y4
567.2778
b9-NH3
980.516
Peptide Sequence
Protein Sequence
- L L G A D S A N V F N I Q E P E E T A A
N Q E Y K -

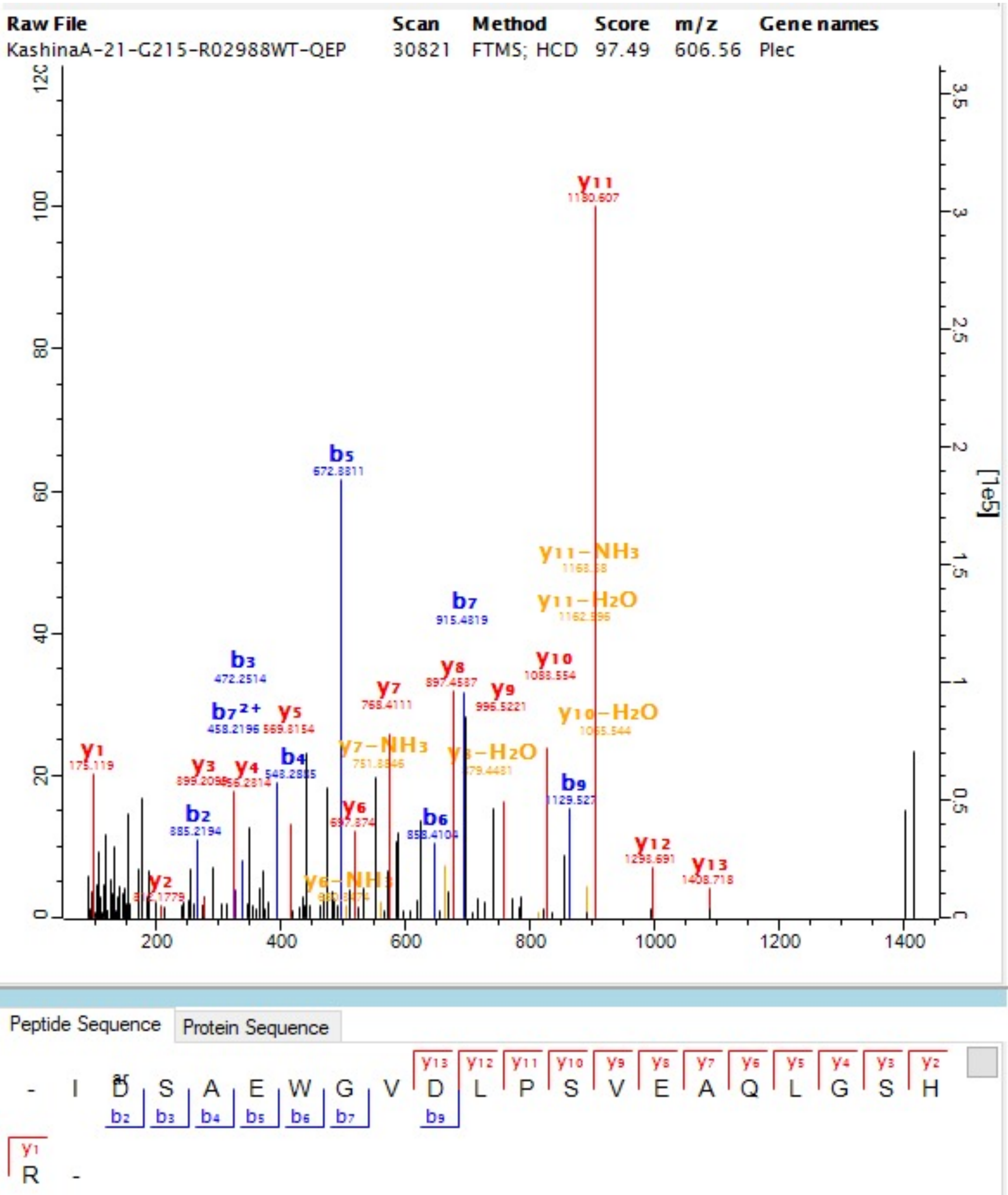

Raw File
KashinaA-21-G215-R02988WT-QEP
Scan
30821
Method
FTMS; HCD
Score
97.49
m/z
606.56
Gene names
Plec
[1e5]
y11
y11-NH3
y11-H2O
b5
672.8811
b7
915.4819
b3
472.2514
y10
y8
y7
y9
996.5221
y10-H2O
b7 2+
458.2196
y5
569.8154
y1
y3
y4
b4
y7-NH3
y8-H2O
b9
b2
885.2194
y6
b6
y12
y13
y2
y6-NH3
Peptide Sequence
Protein Sequence
- I D S A E W G V D L P S V E A Q L G S H
R -

Raw File	Scan	Method	Score	m/z	Gene names
KashinaA-21-G215-R02989WT-QEP	16884	FTMS; HCD	90.91	681.36	Plec

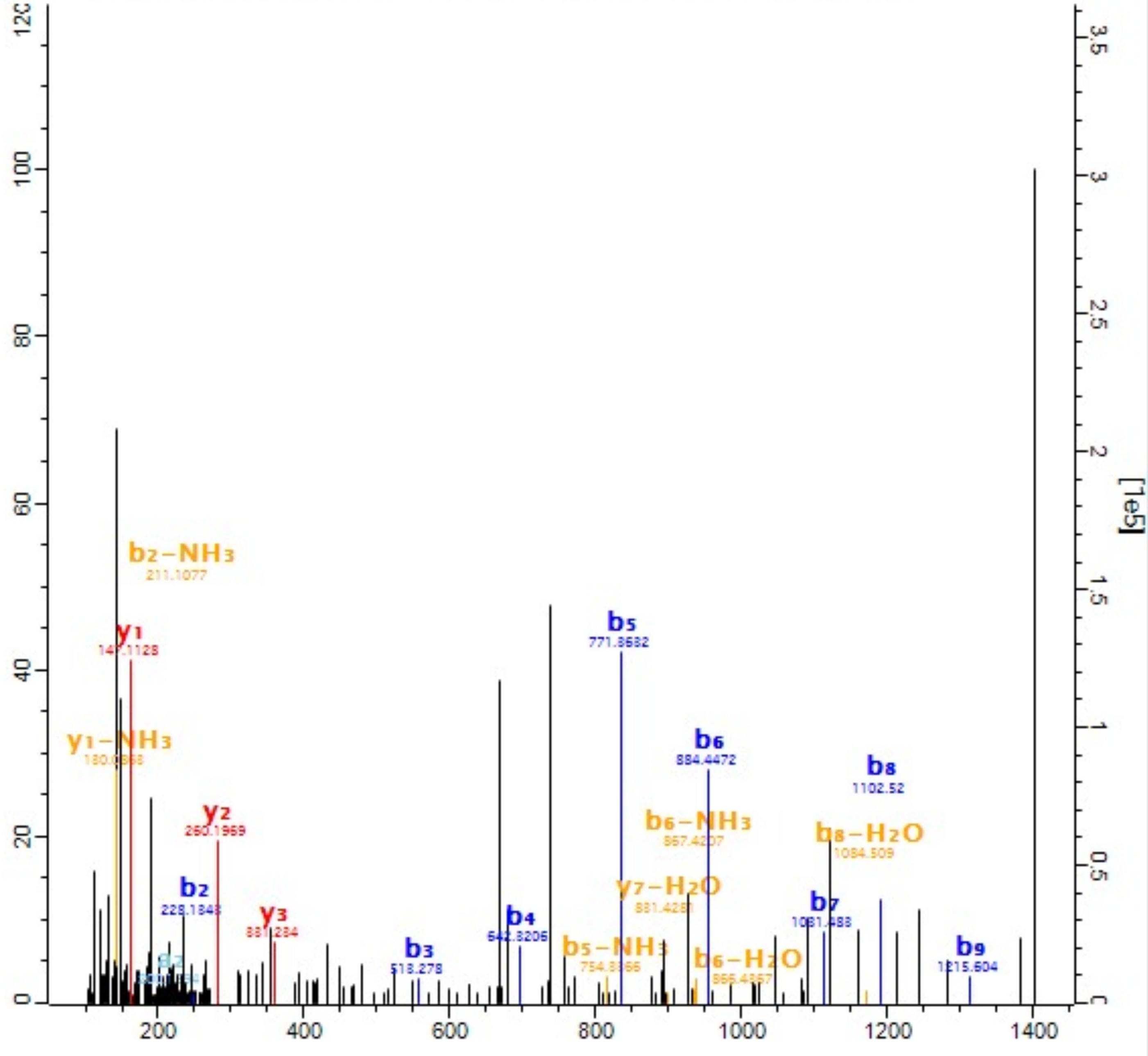

Peptide Sequence | Protein Sequence

- Q V E E E I M A L K -

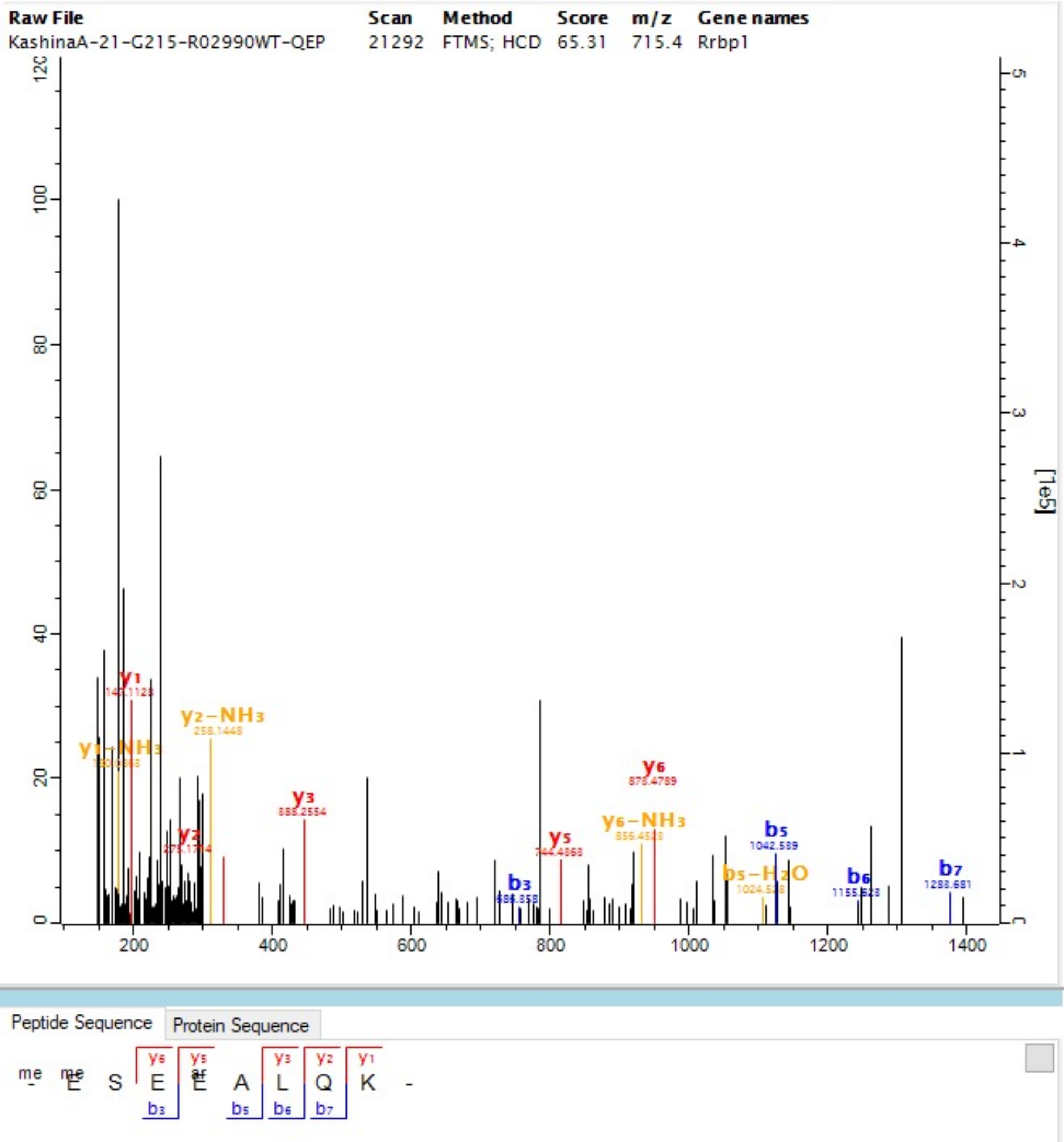

Raw File
KashinaA-21-G215-R02990WT-QEP
Scan
21292
Method
FTMS; HCD
Score
65.31
m/z
715.4
Gene names
Rrbp1
[1e5]
y1
y2-NH3
258.1448
y2
y3
y5
y6
878.4789
y6-NH3
856.4528
b3
b5
1042.589
b5-H2O
b6
b7
1283.681
Peptide Sequence
Protein Sequence

Raw File	Scan	Method	Score	m/z	Gene names
KashinaA-21-G215-R02989WT-QEP	49113	FTMS; HCD	114.2	923.49	Myo1c

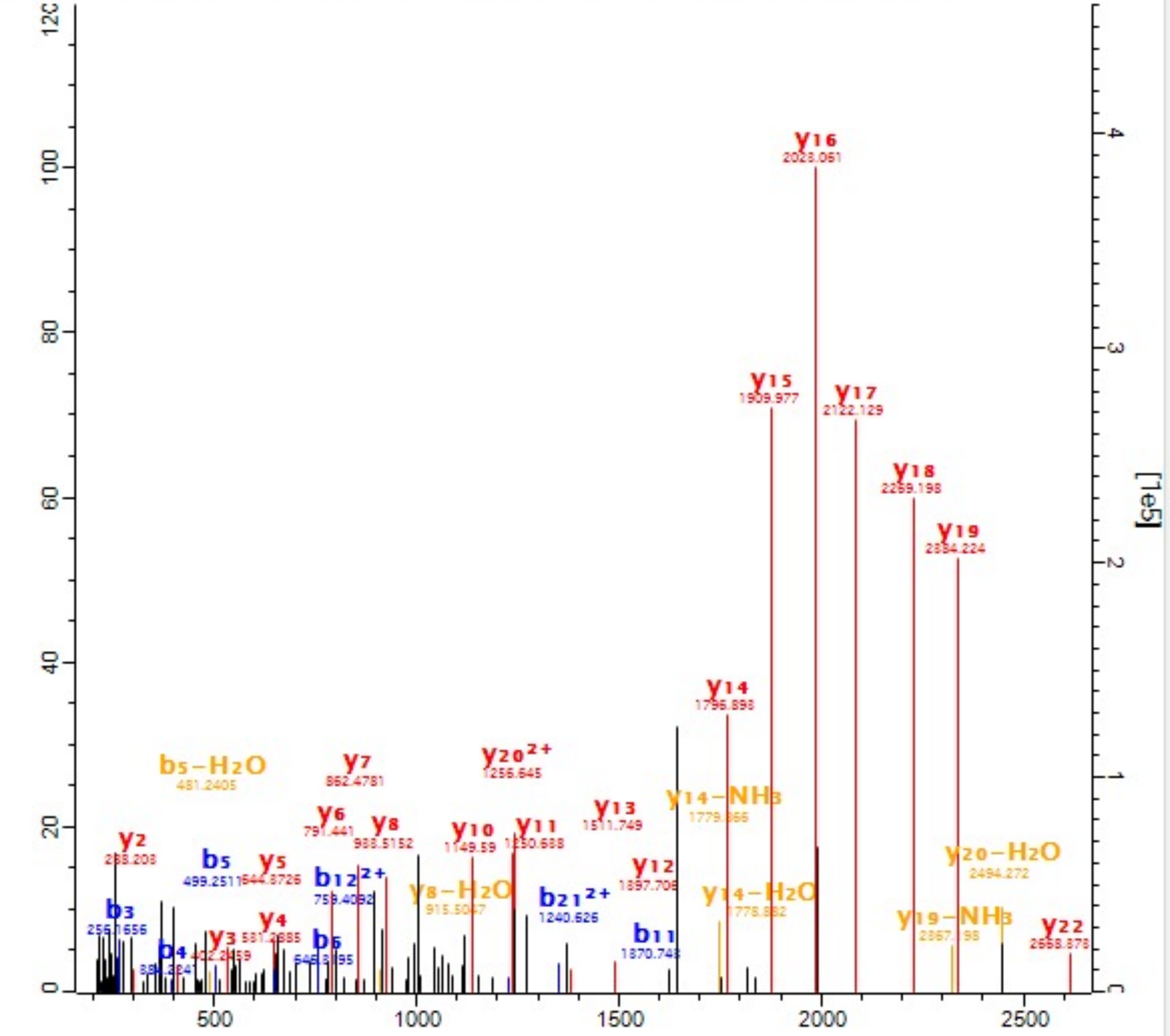

Peptide Sequence | Protein Sequence

- V G V Q D F V L L E N F T S E A A F I E N L R -

y22, y20 2+, y19, y18, y17, y16, y15, y14, y13, y12, y11, y10, y8, y7, y6, y5, y4, y3, y2

b3, b4, b5, b6, b11, b12 2+, b21 2+
