# Supplementary material for: Global Analysis of Post-Translational Side-Chain Arginylation Using Pan-Arginylation Antibodies
Source: Mol Cell Proteomics. 2023 Oct 12;22(11):100664. doi: 10.1016/j.mcpro.2023.100664 (PMC10656225; doi:10.1016/j.mcpro.2023.100664)
Supplement: Supplemental File 2 [file mmc2.pdf]

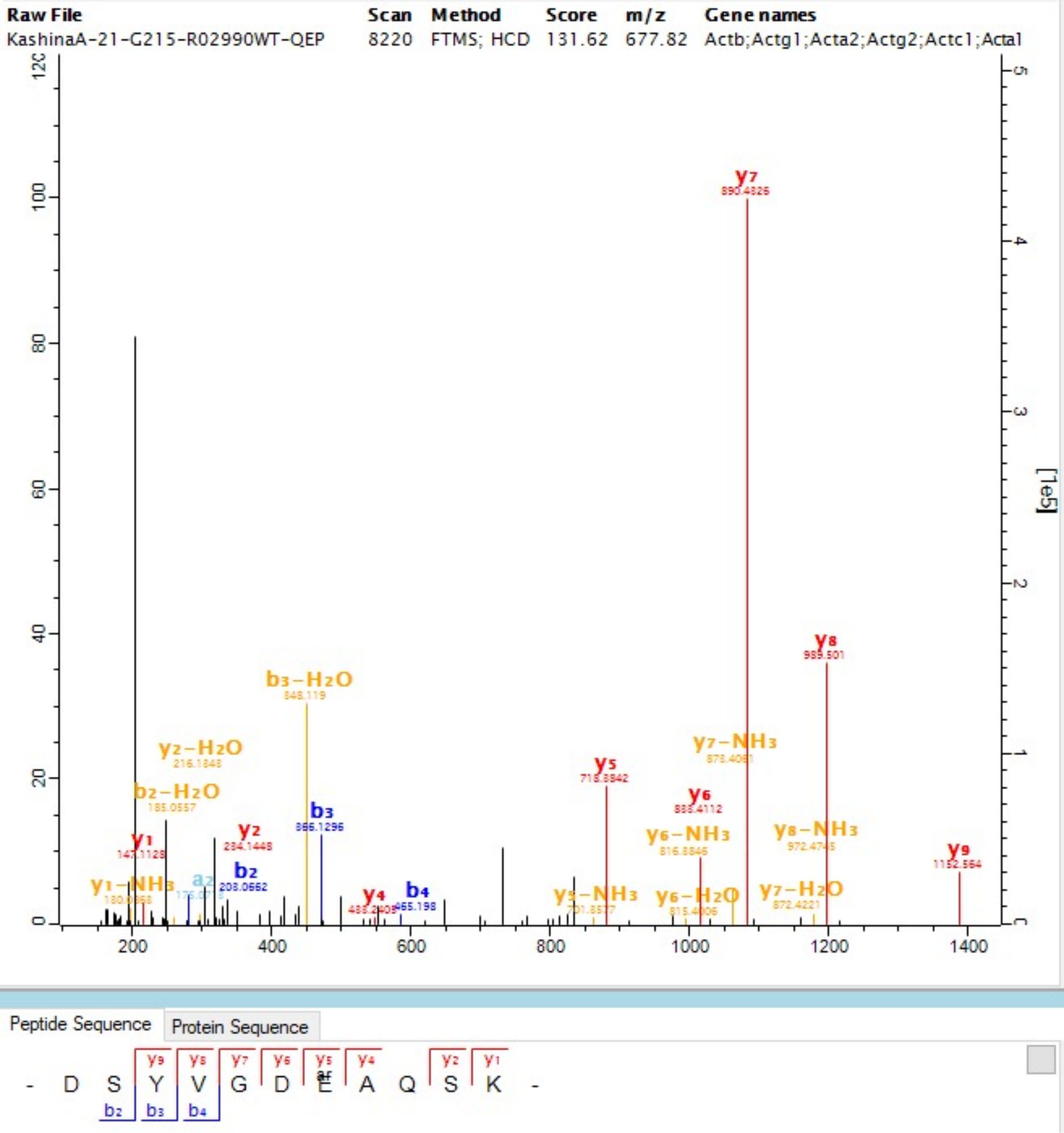
Raw File
KashinaA-21-G215-R02990WT-QEP
Scan
8220
Method
FTMS; HCD
Score
131.62
m/z
677.82
Gene names
Actb;Actg1;Acta2;Actg2;Actc1;Acta1
y7
y8
b3-H2O
y2-H2O
y7-NH3
y5
b2-H2O
y6
b3
y6-NH3
y8-NH3
y1
y2
y9
b2
a2
y1-NH3
y4
b4
y5-NH3
y6-H2O
y7-H2O
[1e5]
Peptide Sequence
Protein Sequence
- D S Y V G D E A Q S K -

Raw File	Scan	Method	Score	m/z	Gene names
KashinaA-21-G215-R02990WT-QEP	18480	FTMS; HCD	136.17	636.34	Actn1

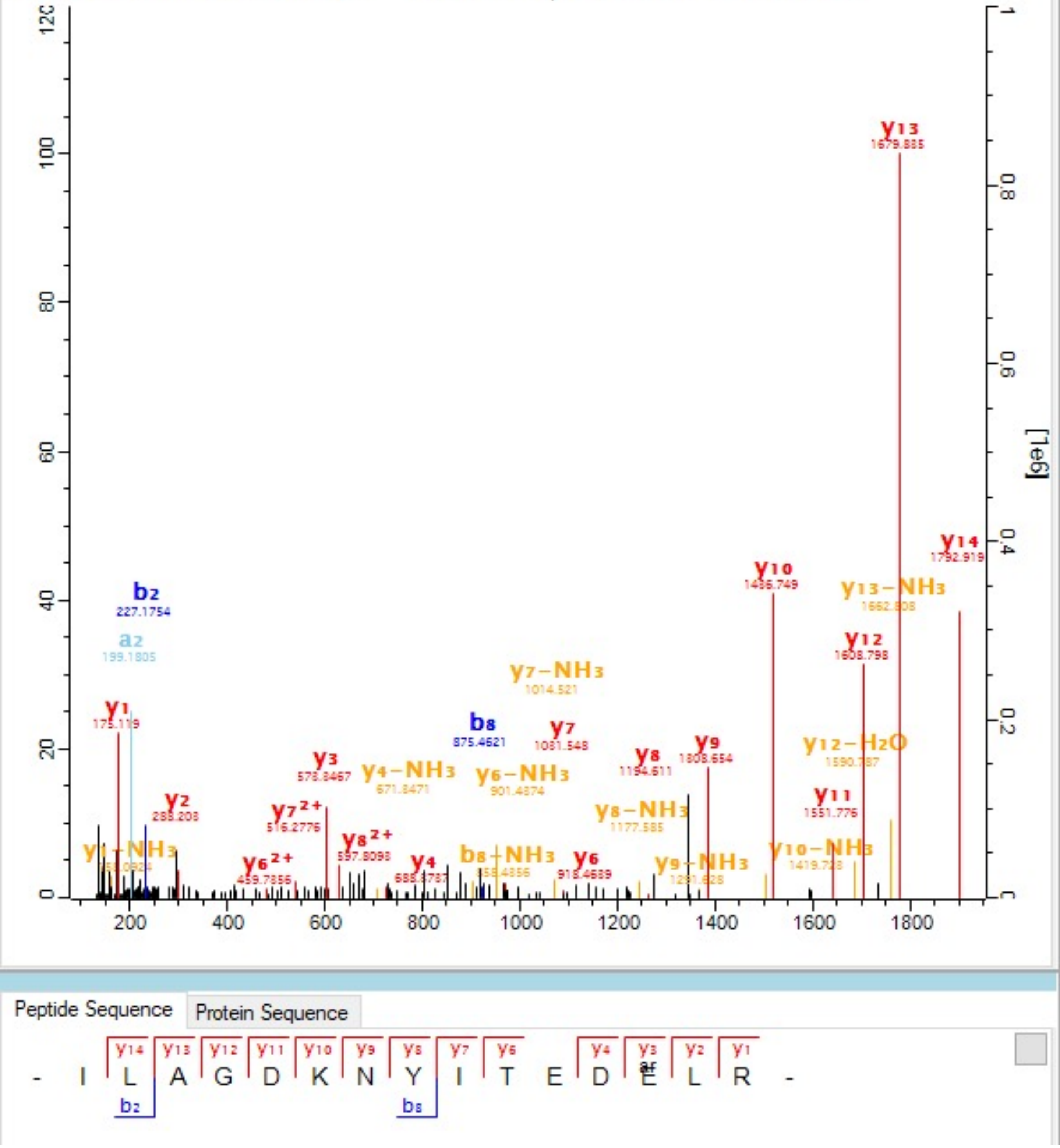

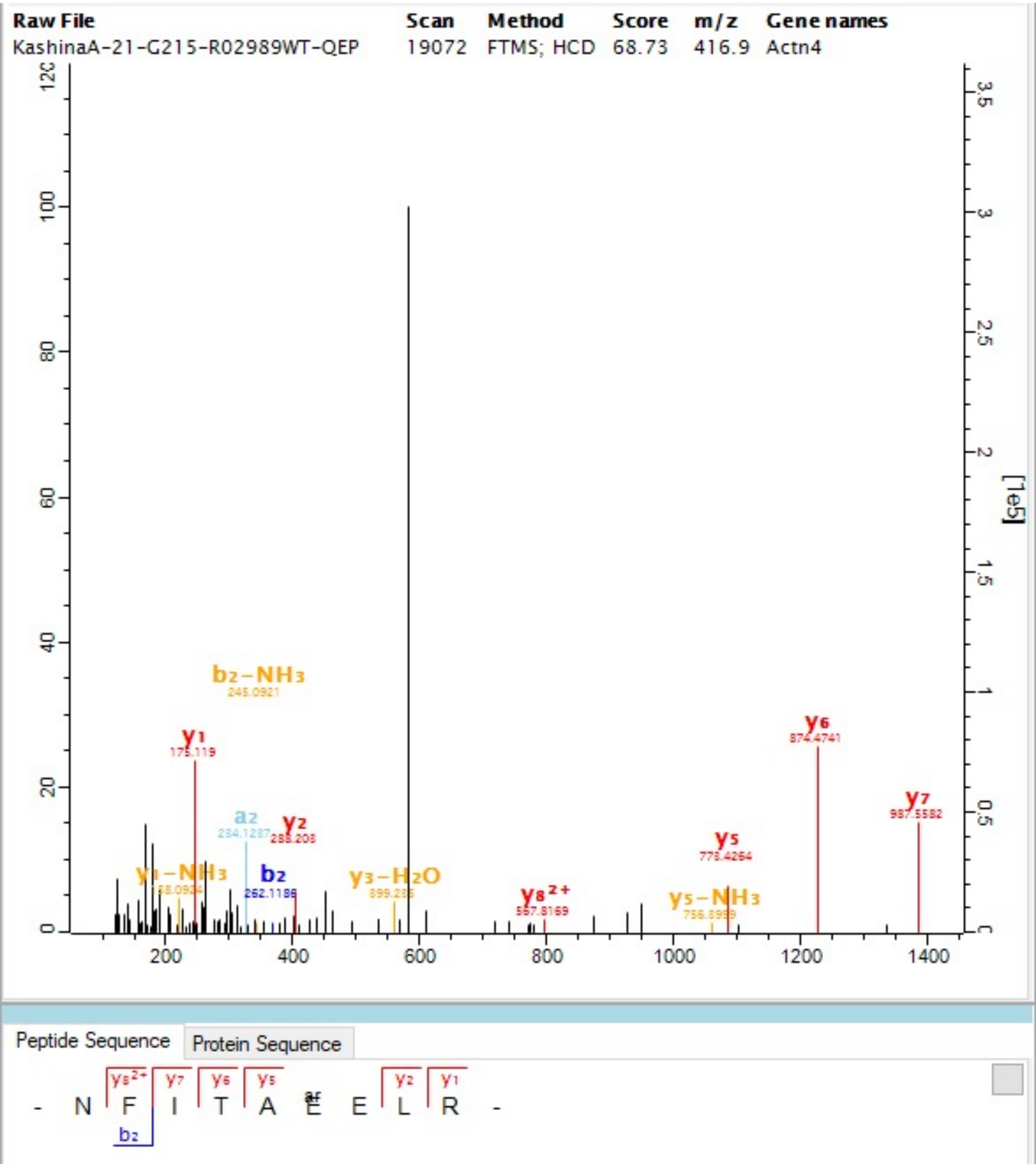
Raw File
KashinaA-21-G215-R02989WT-QEP
Scan
19072
Method
FTMS; HCD
Score
68.73
m/z
416.9
Gene names
Actn4
[1e5]
b2-NH3
245.0921
y1
a2
y2
y1-NH3
b2
y3-H2O
y8 2+
y5
y5-NH3
y6
874.4741
y7
Peptide Sequence
Protein Sequence
- N F I T A E E L R -

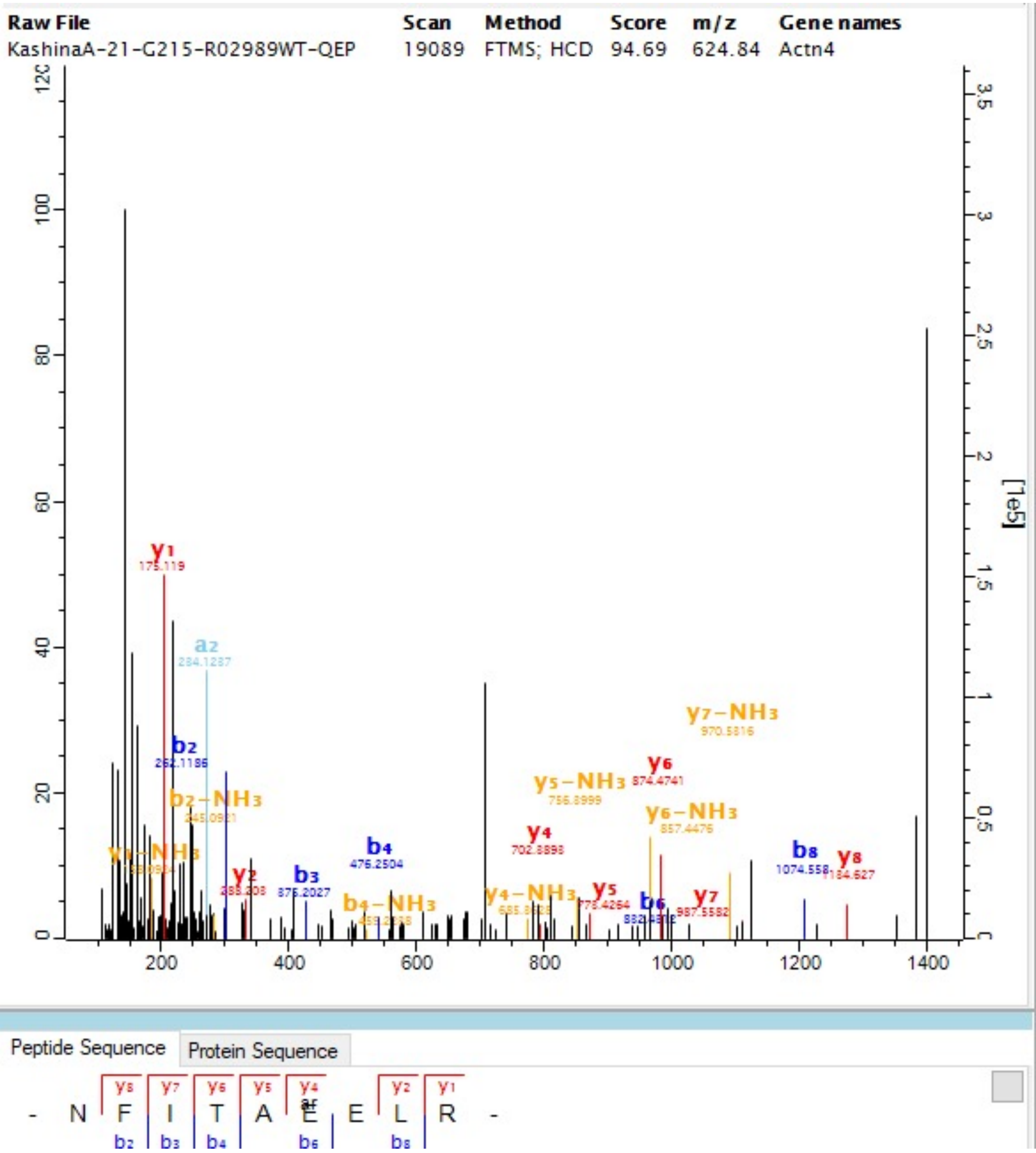

Raw File
Scan
Method
Score
m/z
Gene names
KashinaA-21-G215-R02989WT-QEP
19089
FTMS; HCD
94.69
624.84
Actn4
y1
175.119
a2
b2
262.1186
b2-NH3
245.0921
y2
b3
b4
476.2504
b4-NH3
y4
y4-NH3
y5
y5-NH3
y6
874.4741
y6-NH3
857.4476
b6
y7
y7-NH3
970.5316
b8
1074.558
y8
[1e5]
Peptide Sequence
Protein Sequence
- N F I T A E E L R -

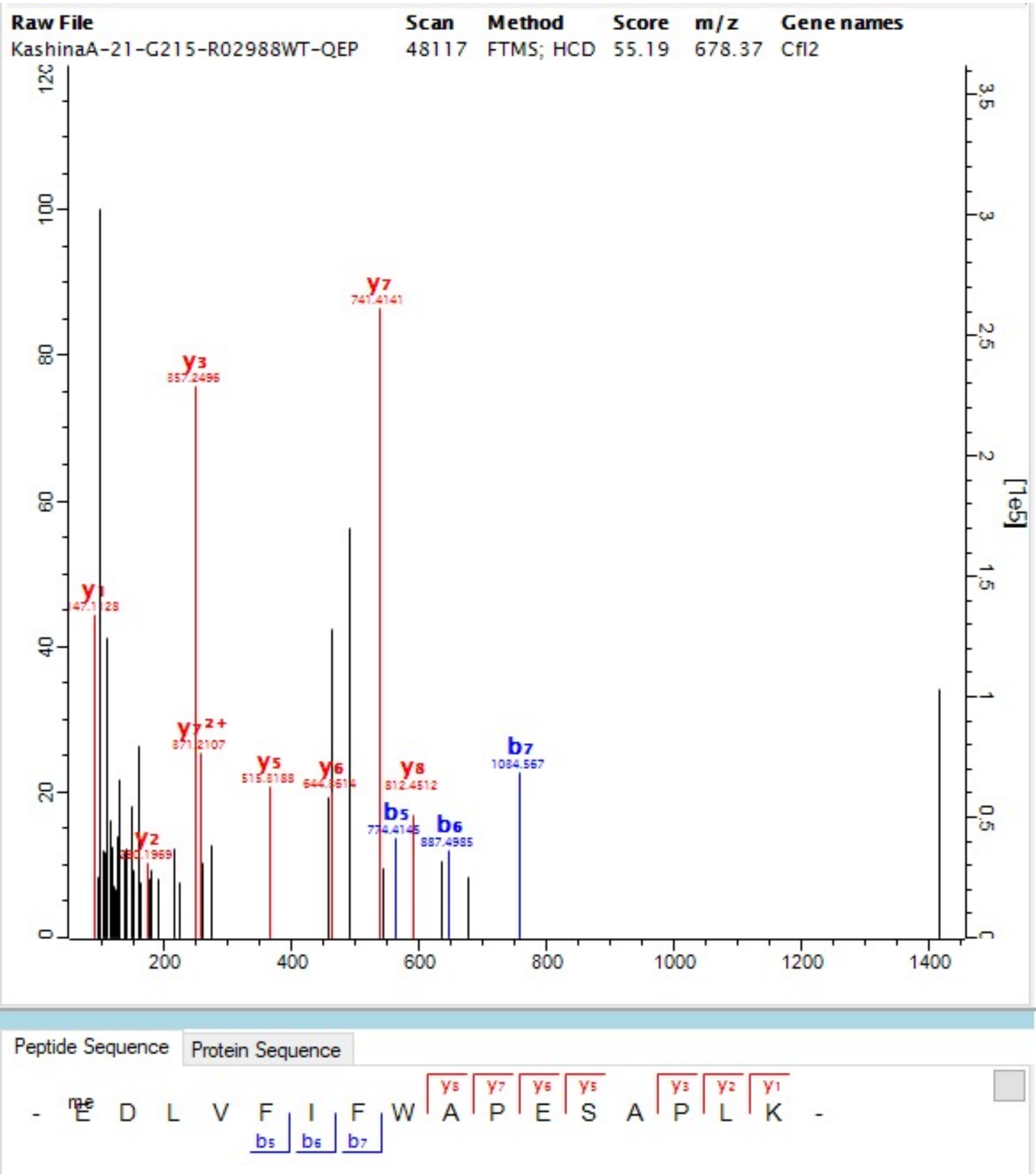
Raw File
KashinaA-21-G215-R02988WT-QEP
Scan
48117
Method
FTMS; HCD
Score
55.19
m/z
678.37
Gene names
Cfl2
y7
741.4141
y3
y1
y7 2+
y5
515.8188
y6
y8
812.4512
b7
1084.567
b5
b6
887.4985
y2
[1e5]
Peptide Sequence
Protein Sequence
- E D L V F I F W A P E S A P L K -
me
b5 b6 b7
y8 y7 y6 y5 y3 y2 y1

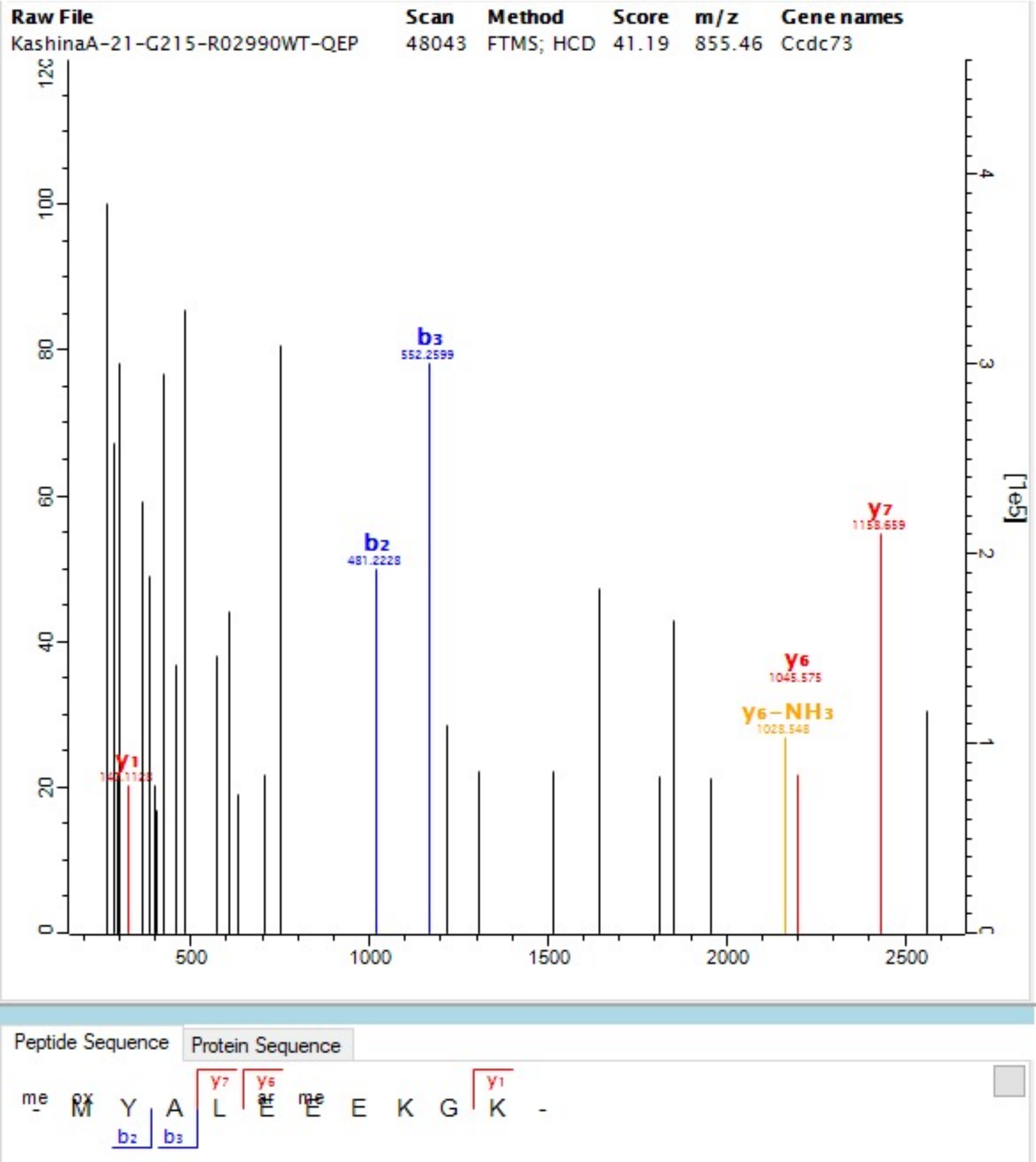

Raw File
KashinaA-21-G215-R02990WT-QEP
Scan
48043
Method
FTMS; HCD
Score
41.19
m/z
855.46
Gene names
Ccdc73
b3
552.2599
b2
481.2228
y7
y6
1045.575
y6-NH3
1028.548
y1
[1e5]
Peptide Sequence
Protein Sequence
- M Y A L E E E K G K -

Raw File	Scan	Method	Score	m/z	Gene names
KashinaA-21-G215-R02988WT-QEP	48530	FTMS; HCD	54.16	855.96	Ccdc73

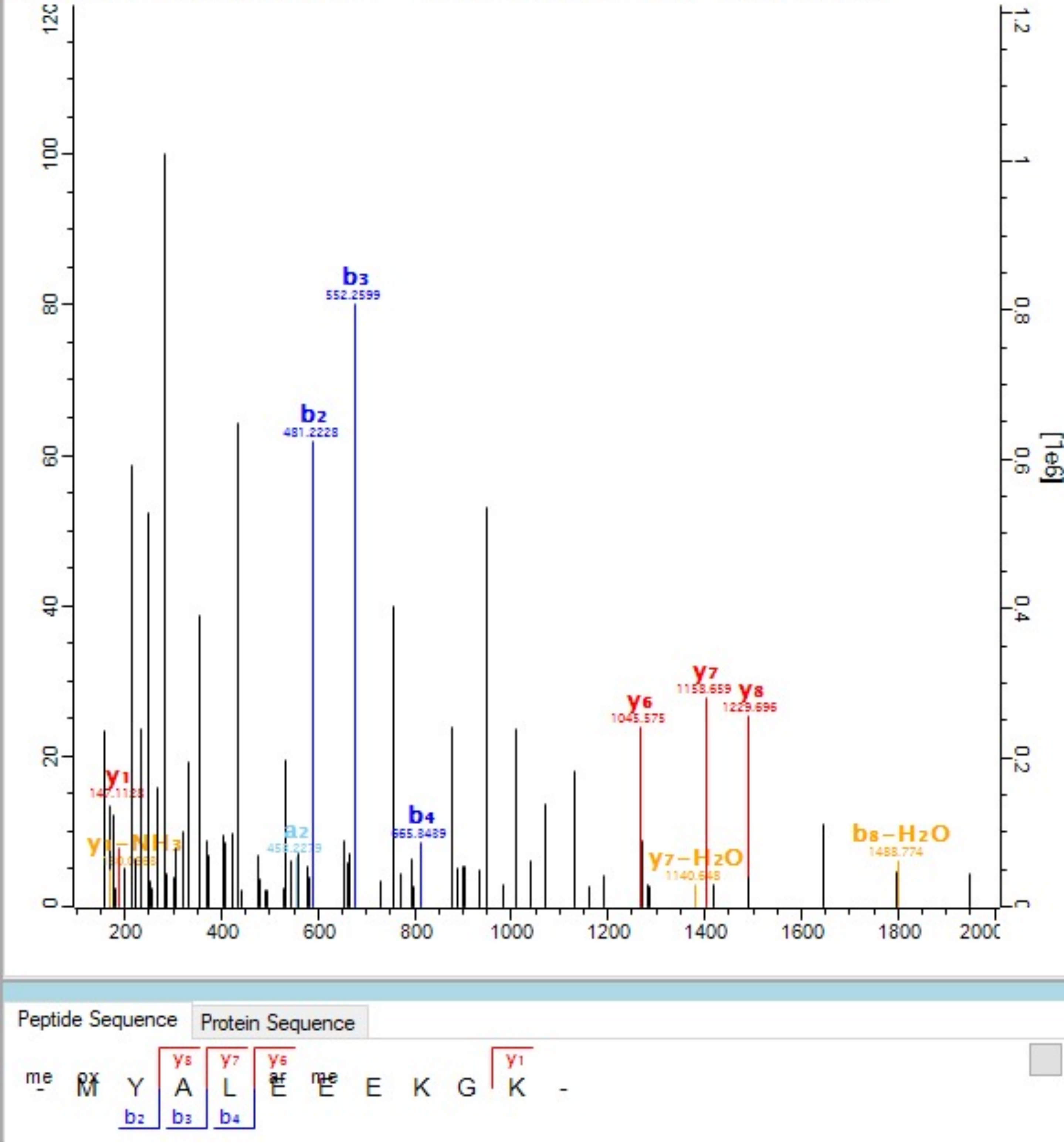

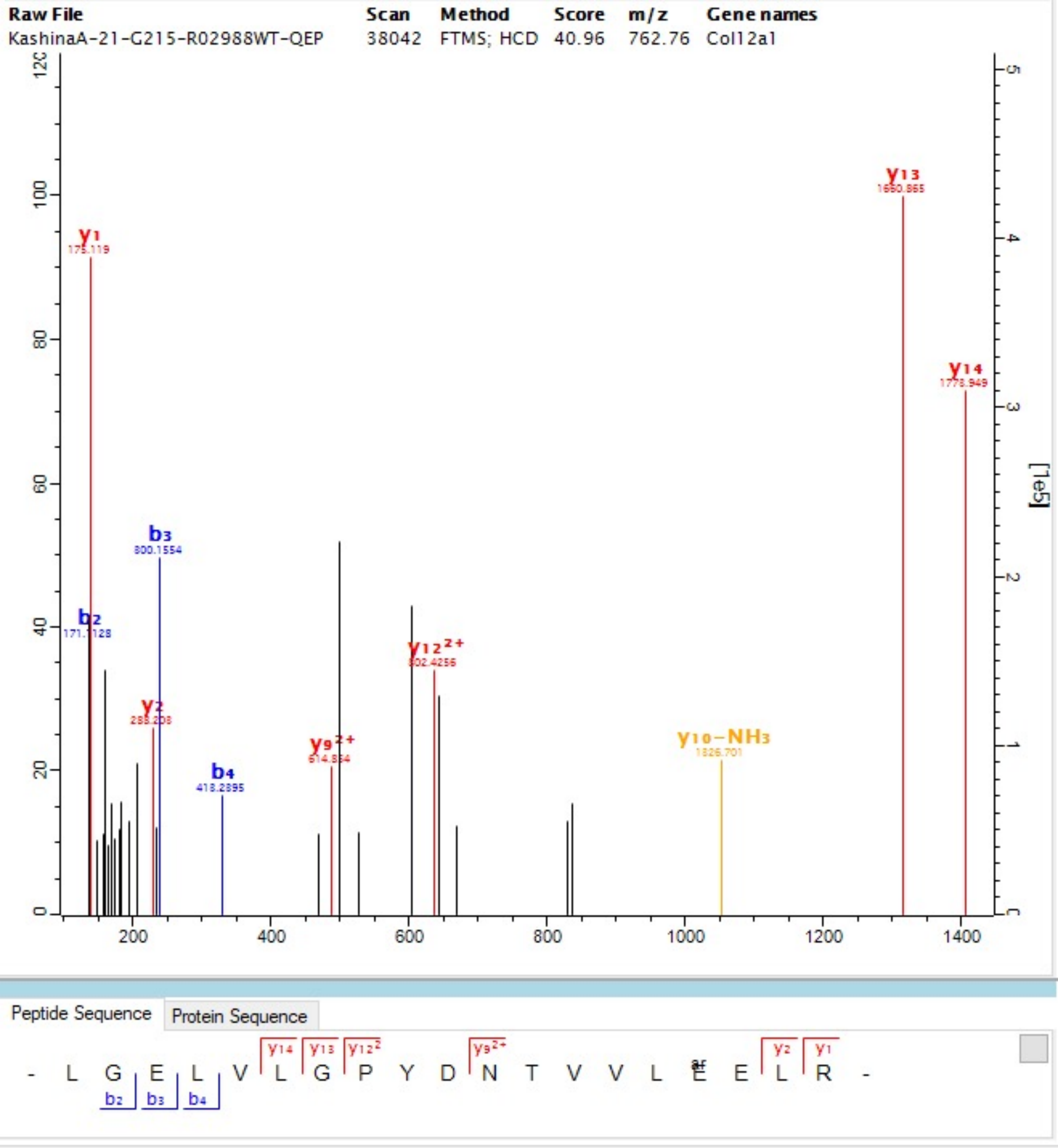
Raw File
KashinaA-21-G215-R02988WT-QEP
Scan
38042
Method
FTMS; HCD
Score
40.96
m/z
762.76
Gene names
Col12a1
y1
y13
y14
b3
b2
y12 2+
y2
y9 2+
b4
y10-NH3
[1e5]
Peptide Sequence
Protein Sequence
- L G E L V L G P Y D N T V V L E E L R -

Raw File	Scan	Method	Score	m/z	Gene names
KashinaA-21-G215-R02989WT-QEP	21861	FTMS; HCD	54.61	595.64	Col12a1

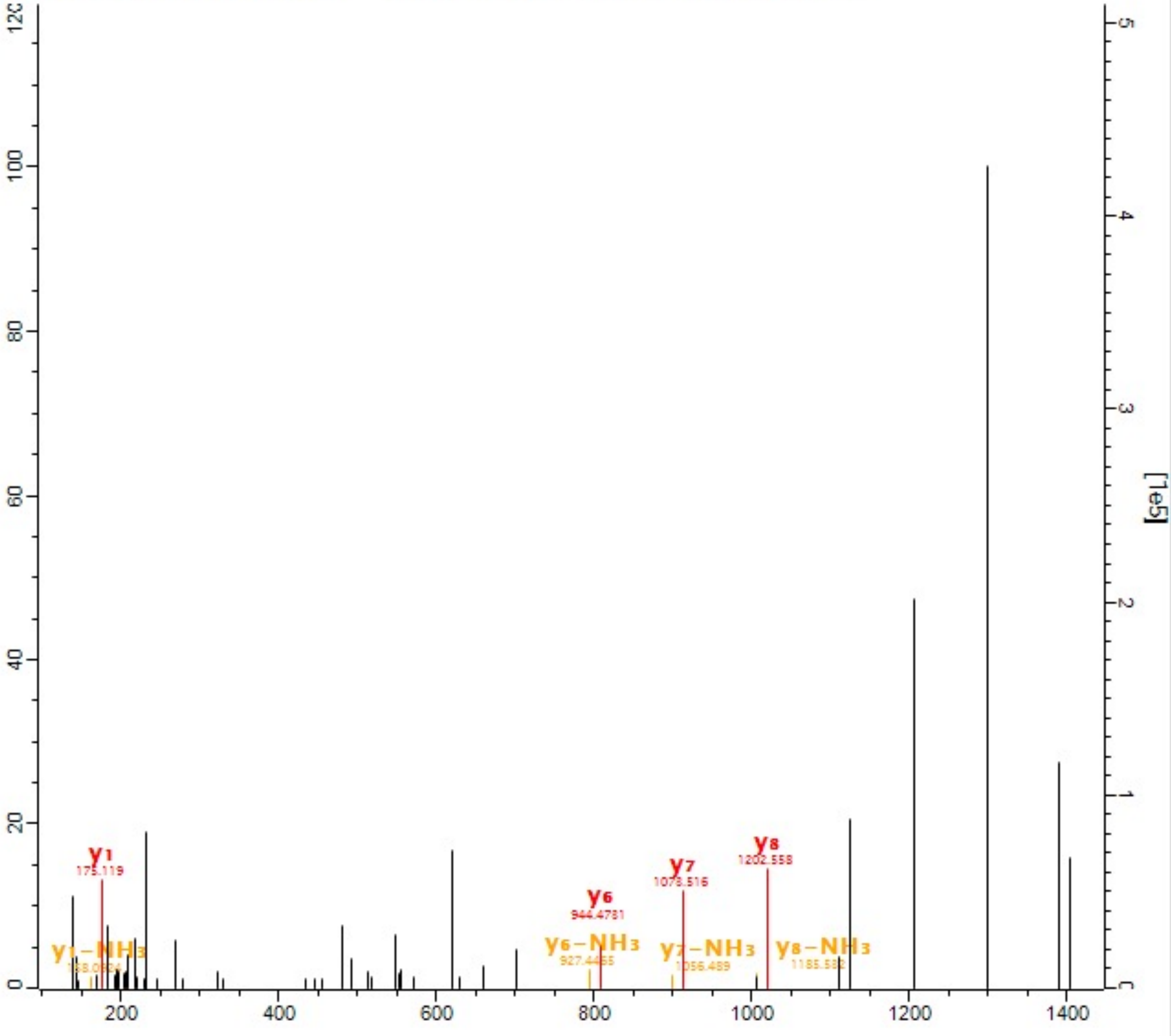

Peptide Sequence | Protein Sequence

- V D E E T E H T M R -

(di on N-terminus and D; me on E; y8, y7, y6, y1 fragment markers)

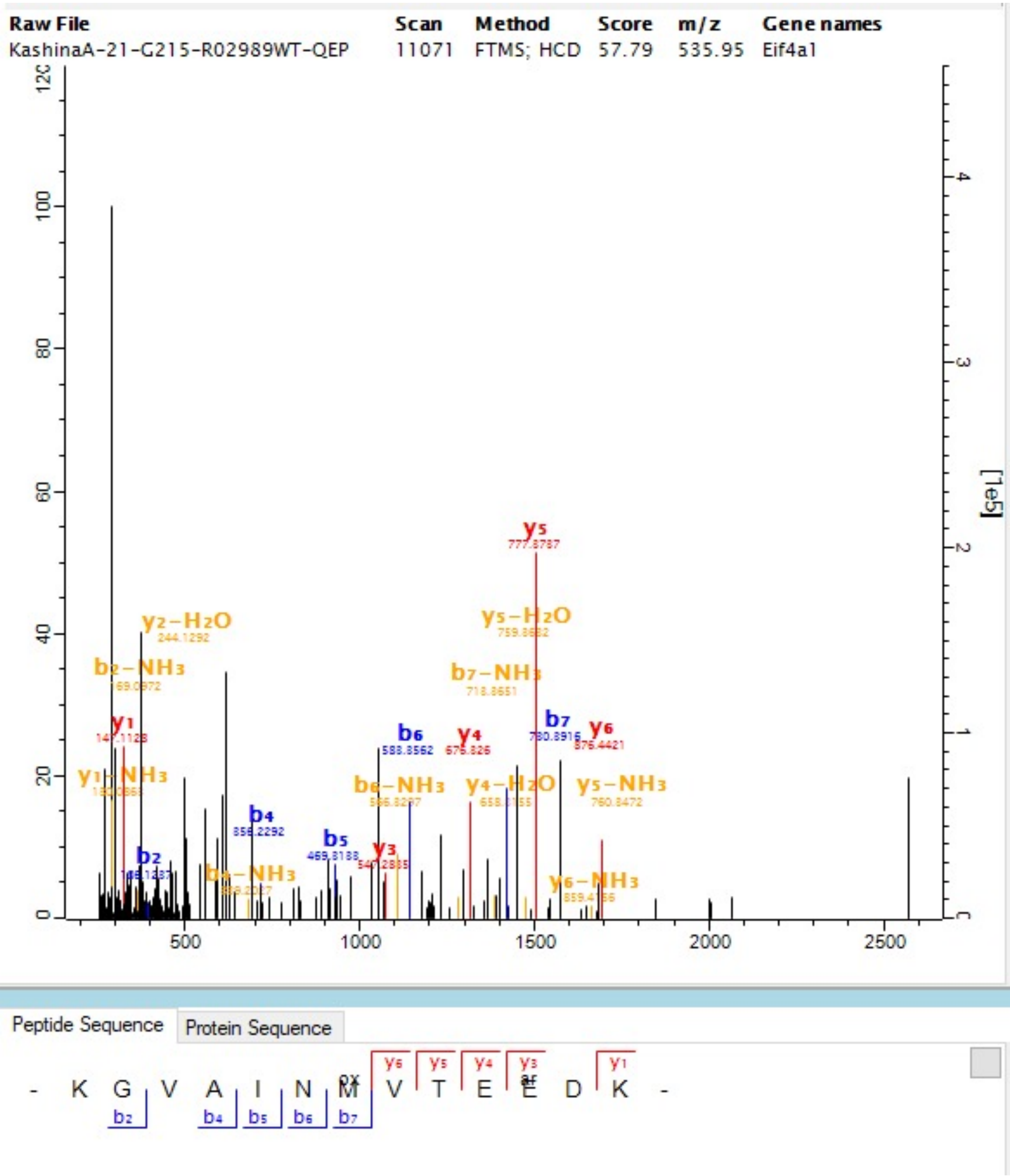
Raw File
KashinaA-21-G215-R02989WT-QEP
Scan
11071
Method
FTMS; HCD
Score
57.79
m/z
535.95
Gene names
Eif4a1
y5
777.8787
y5-H2O
759.8682
b7-NH3
718.8651
y2-H2O
244.1292
b2-NH3
b7
780.8916
y6
876.4421
b6
588.8562
y4
y1
y1-NH3
b6-NH3
y4-H2O
y5-NH3
760.8472
b4
858.2292
b5
469.8188
y3
b2
b4-NH3
y6-NH3
[1e5]
Peptide Sequence
Protein Sequence
- K G V A I N M V T E E D K -
ox
ar
y6 y5 y4 y3 y1
b2 b4 b5 b6 b7

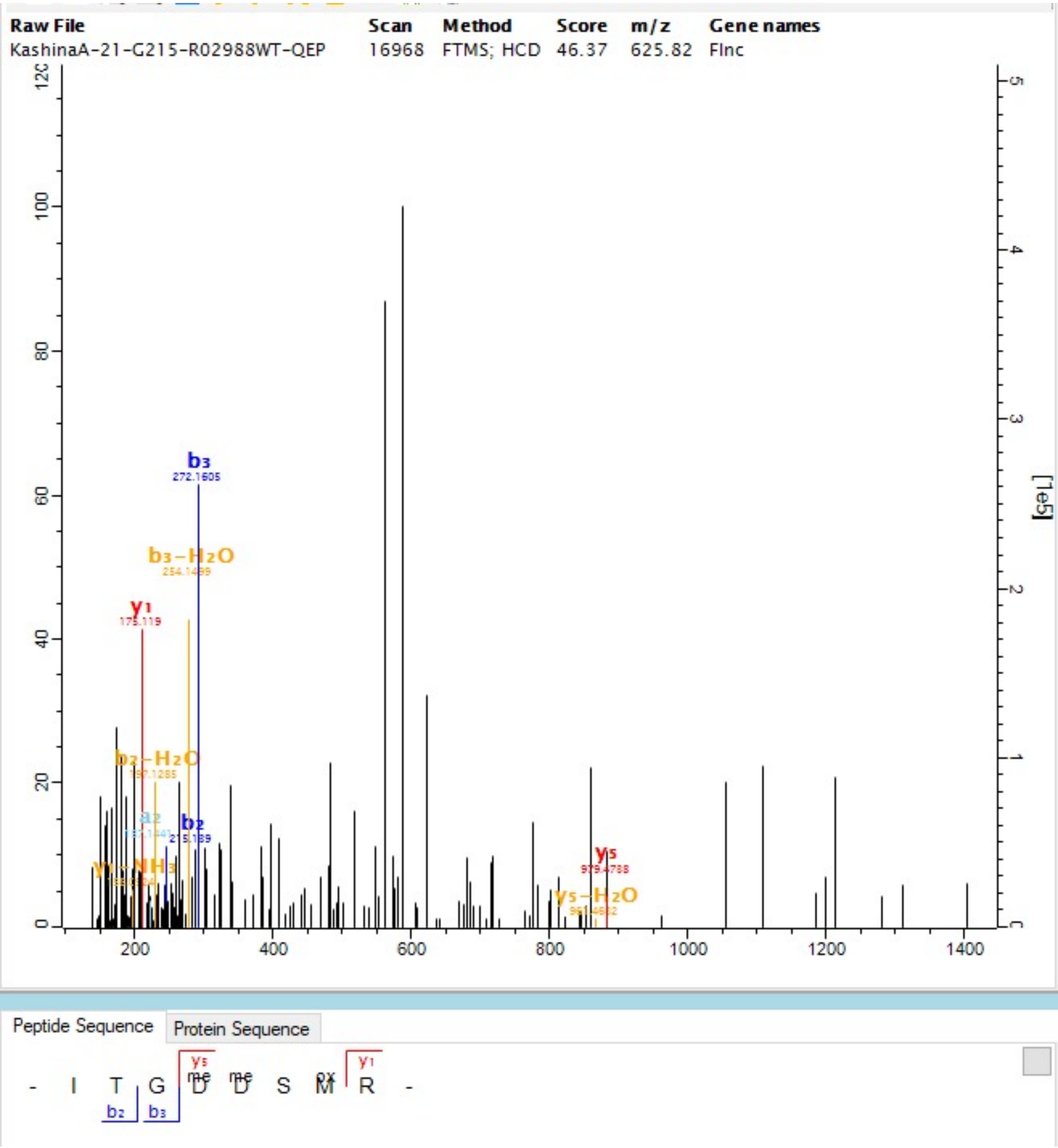
Raw File
KashinaA-21-G215-R02988WT-QEP
Scan
16968
Method
FTMS; HCD
Score
46.37
m/z
625.82
Gene names
Flnc
b3
272.1605
b3-H2O
254.1499
y1
175.119
b2-H2O
197.1285
b2
215.139
y5
979.4788
y5-H2O
961.4682
[1e5]
Peptide Sequence
Protein Sequence
- I T G D D S M R -
me me ox
b2 b3 y5 y1

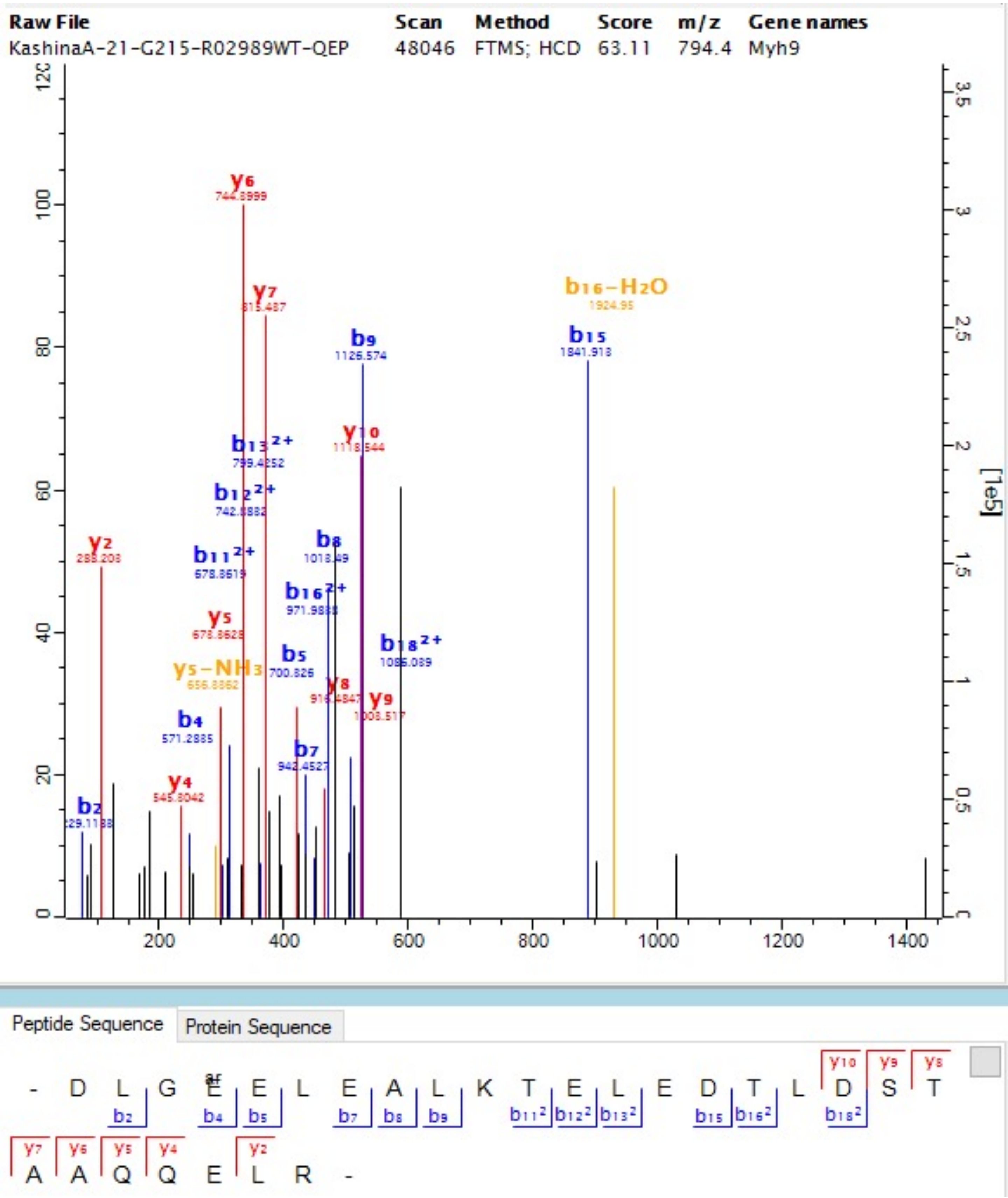

Raw File
KashinaA-21-G215-R02989WT-QEP
Scan
48046
Method
FTMS; HCD
Score
63.11
m/z
794.4
Gene names
Myh9
y6 744.8999
y7 815.487
b9 1126.574
b16-H2O 1924.95
b15 1841.918
y10 1118.544
b13 2+ 799.4252
b12 2+ 742.8882
y2 288.208
b11 2+ 678.8619
b8 1013.49
b16 2+ 971.9888
y5 678.8628
b18 2+ 1085.089
y5-NH3 656.8862
b5 700.826
y8 916.4847
y9 1003.517
b4 571.2885
b7 942.4527
y4 545.8042
b2 229.1188
0
20
40
60
80
100
120
200
400
600
800
1000
1200
1400
0
0.5
1
1.5
2
2.5
3
3.5
[1e5]
Peptide Sequence
Protein Sequence
- D L G E E L E A L K T E L E D T L D S T
A A Q Q E L R -
b2 b4 b5 b7 b8 b9 b11 2 b12 2 b13 2 b15 b16 2 b18 2
y10 y9 y8 y7 y6 y5 y4 y2

Raw File	Scan	Method	Score	m/z	Gene names
KashinaA-21-G215-R02988WT-QEP	48768	FTMS; HCD	92.83	794.65	Myh9

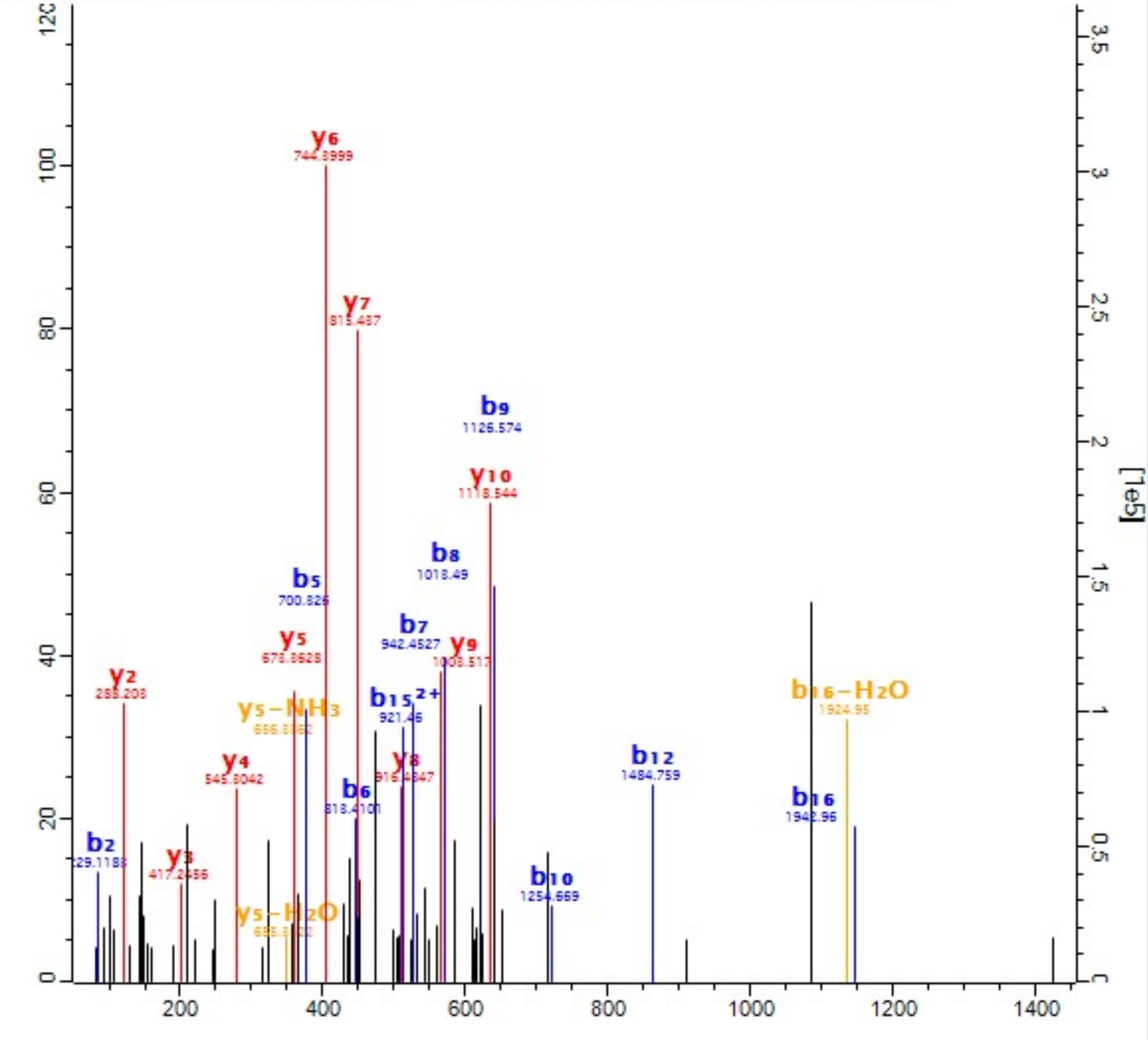

Peptide Sequence | Protein Sequence

- D L G E E L E A L K T E L E D T L D S T A A Q Q E L R -

b2, b5, b6, b7, b8, b9, b10, b12, b15², b16

y10, y9, y8, y7, y6, y5, y4, y3, y2

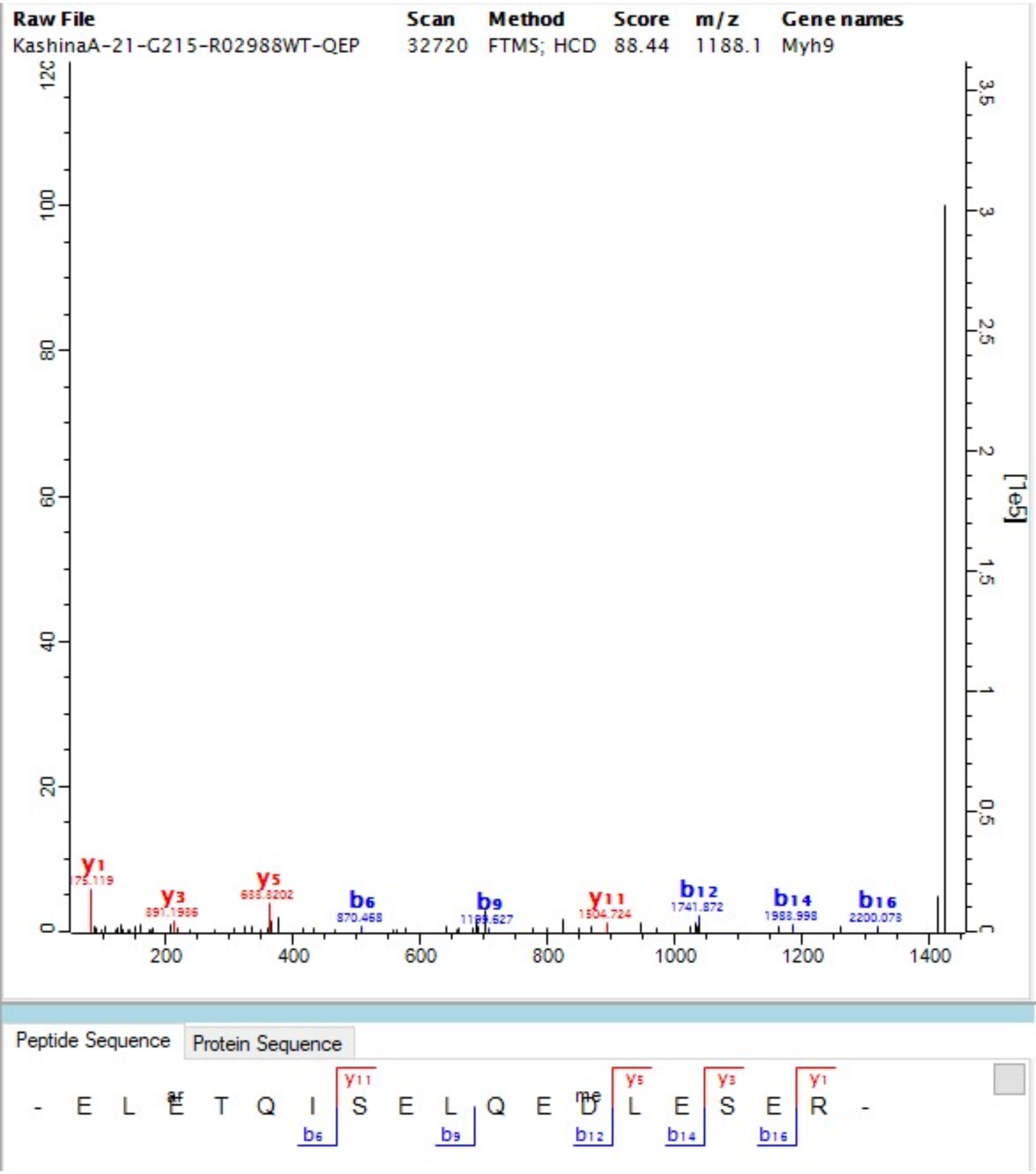

Raw File
KashinaA-21-G215-R02988WT-QEP
Scan
32720
Method
FTMS; HCD
Score
88.44
m/z
1188.1
Gene names
Myh9
[1e5]
y1
y3
y5
b6
870.468
b9
y11
1504.724
b12
1741.872
b14
b16
2200.078
Peptide Sequence
Protein Sequence
- E L E T Q I S E L Q E D L E S E R -

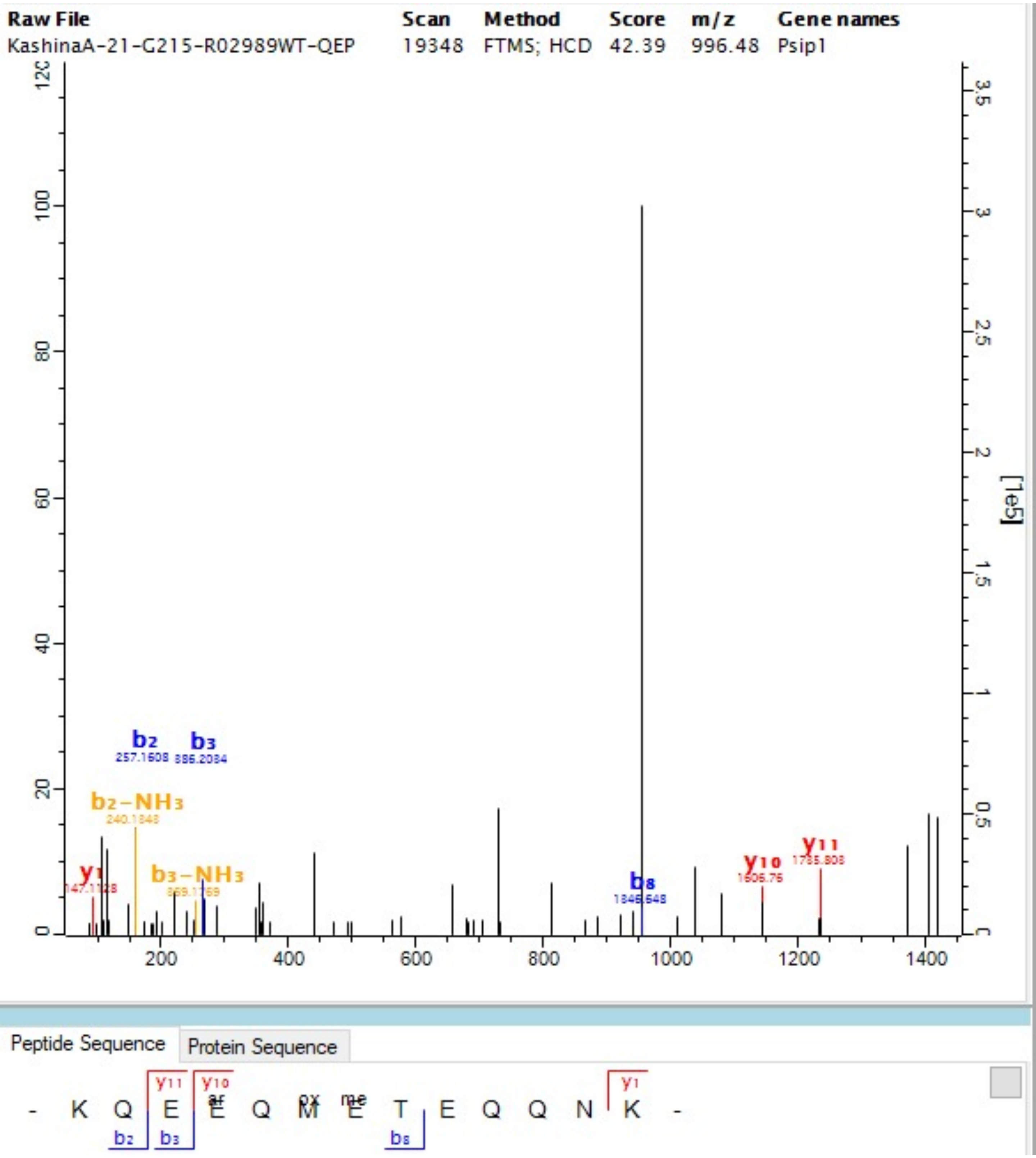

Raw File
KashinaA-21-G215-R02989WT-QEP
Scan
19348
Method
FTMS; HCD
Score
42.39
m/z
996.48
Gene names
Psip1
[1e5]
b2
257.1608
b3
b2-NH3
240.1848
y1
147.1128
b3-NH3
b8
y10
y11
Peptide Sequence
Protein Sequence
- K Q E E Q M E T E Q Q N K -

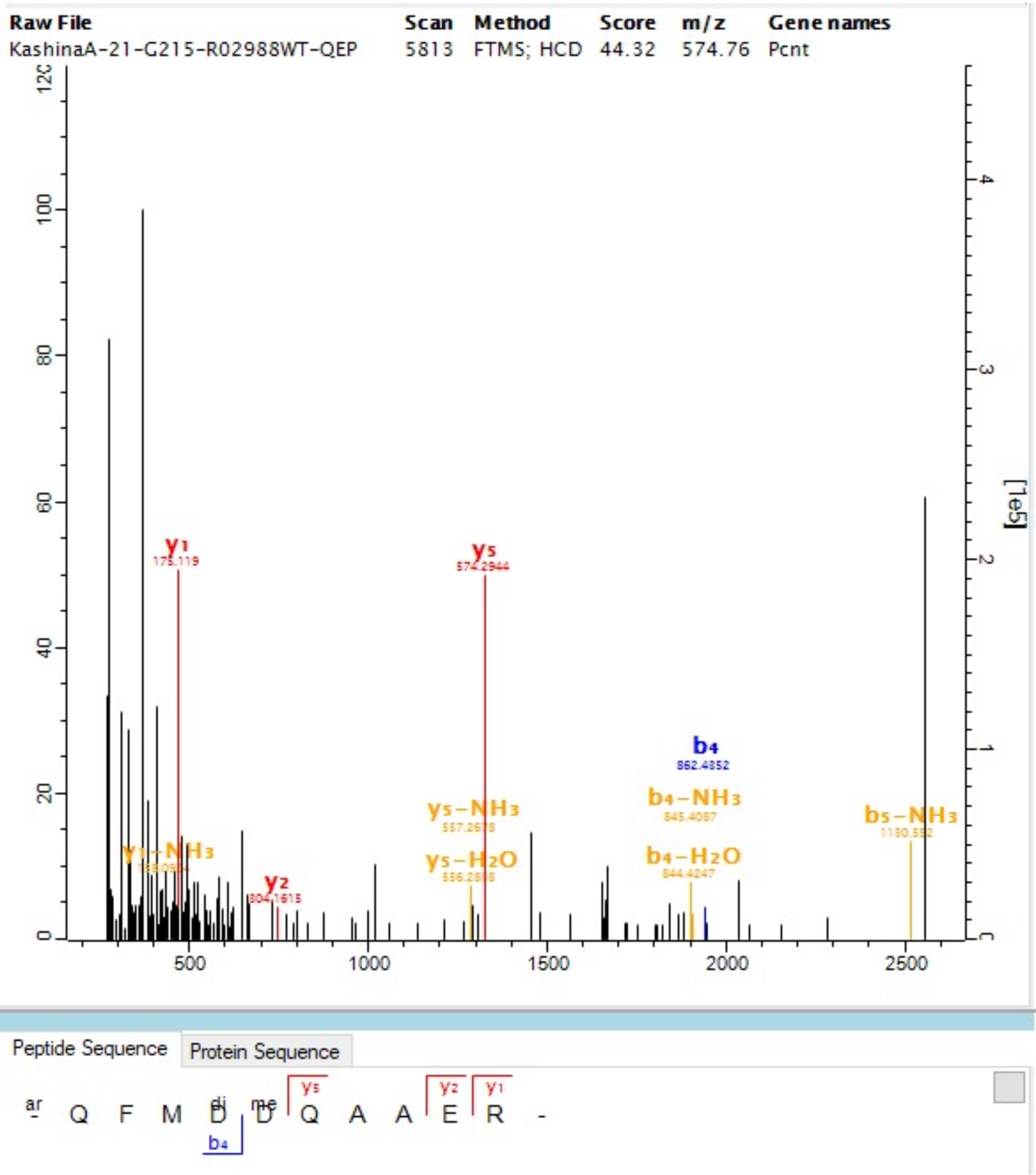
Raw File
KashinaA-21-G215-R02988WT-QEP
Scan
5813
Method
FTMS; HCD
Score
44.32
m/z
574.76
Gene names
Pcnt
y1
175.119
y5
574.2944
y2
304.1615
y1−NH3
y5−NH3
557.2678
y5−H2O
556.2838
b4
862.4852
b4−NH3
845.4087
b4−H2O
844.4247
b5−NH3
1180.552
[1e5]
Peptide Sequence
Protein Sequence
ar - Q F M D D Q A A E R -
di
me
y5
y2
y1
b4

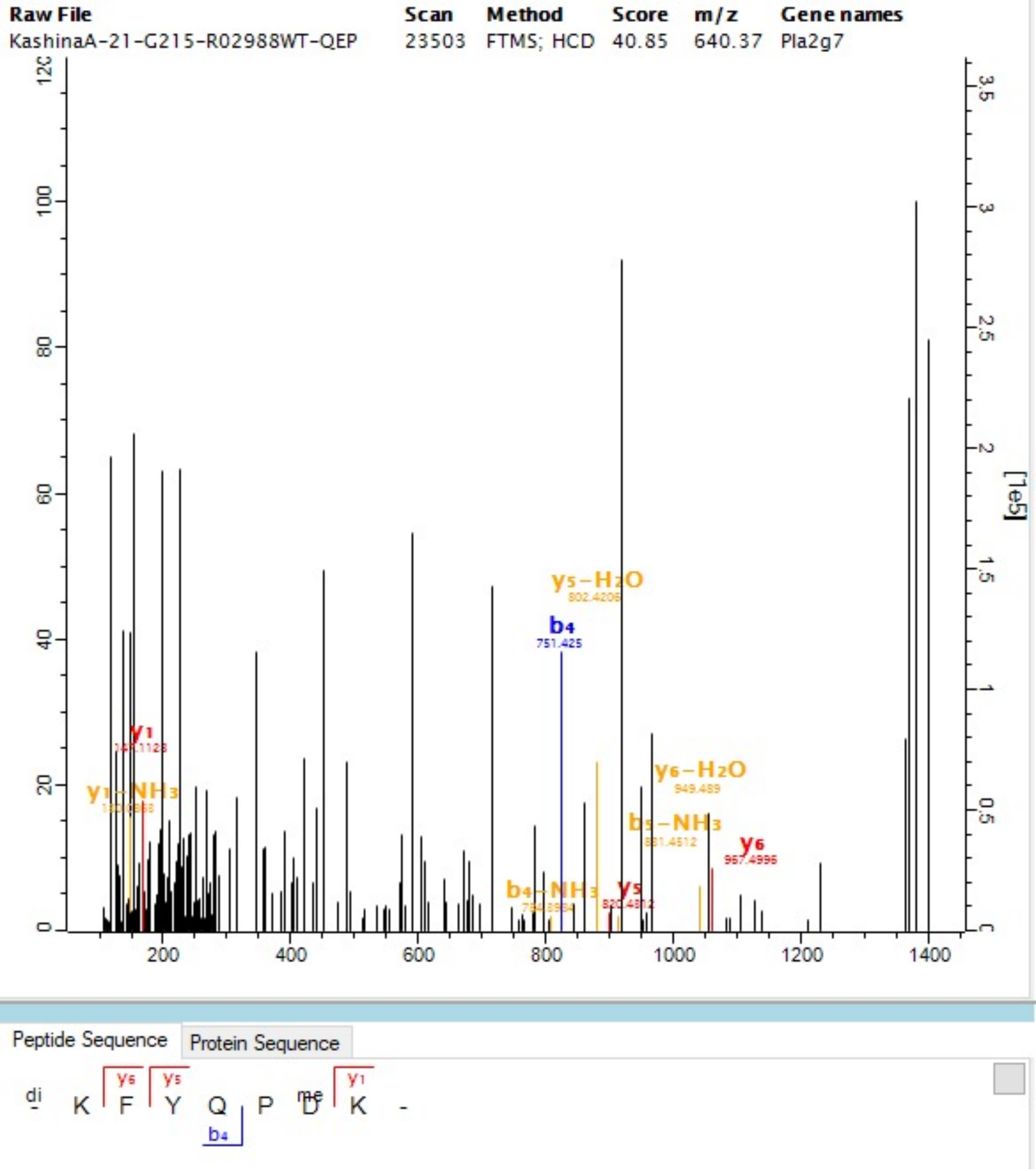
Raw File
KashinaA-21-G215-R02988WT-QEP
Scan
23503
Method
FTMS; HCD
Score
40.85
m/z
640.37
Gene names
Pla2g7
y5-H2O
802.4206
b4
751.425
y1
y1-NH3
y6-H2O
949.489
b5-NH3
y6
b4-NH3
y5
[1e5]
Peptide Sequence
Protein Sequence
di - K F Y Q P D K -
me
y6
y5
y1
b4

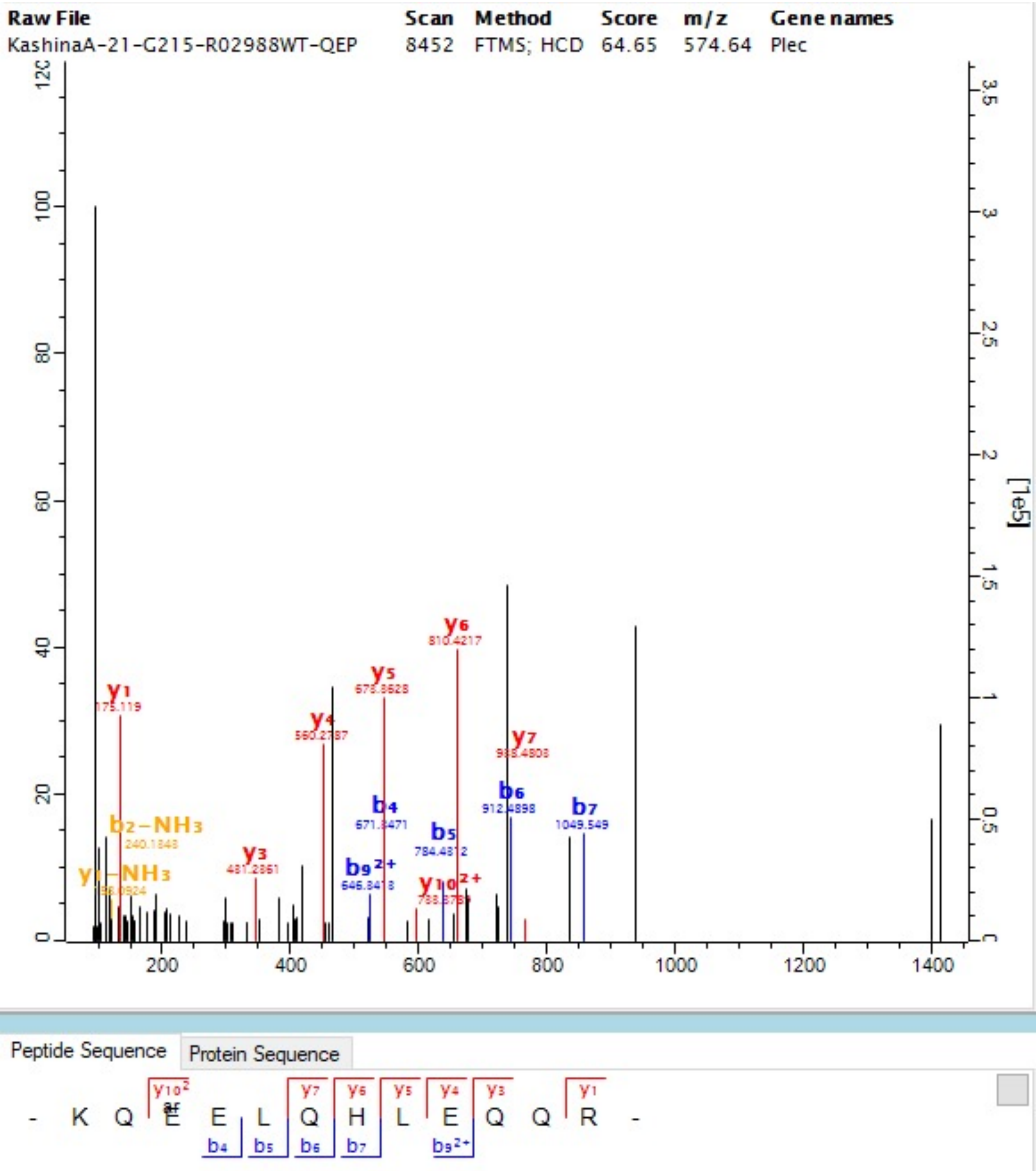

Raw File
KashinaA-21-G215-R02988WT-QEP
Scan
8452
Method
FTMS; HCD
Score
64.65
m/z
574.64
Gene names
Plec
y1
y4
560.2787
y5
y6
810.4217
y7
b2-NH3
240.1343
y3
481.2861
b4
671.3471
b5
784.4312
b6
912.4898
b7
1049.549
b9 2+
646.8418
y10 2+
[1e5]
Peptide Sequence
Protein Sequence
- K Q E E L Q H L E Q Q R -

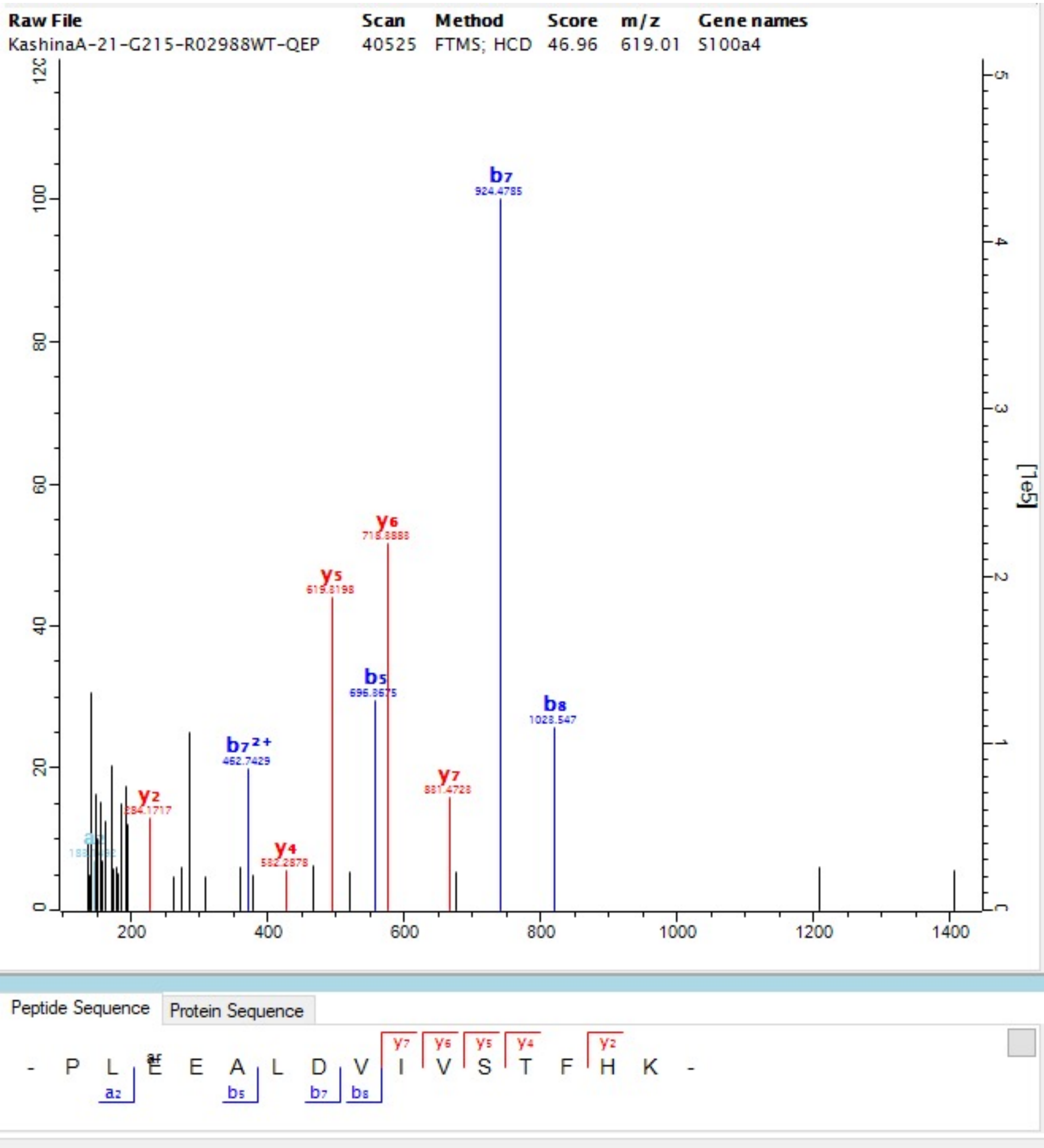
Raw File
KashinaA-21-G215-R02988WT-QEP
Scan
40525
Method
FTMS; HCD
Score
46.96
m/z
619.01
Gene names
S100a4
[1e5]
b7
924.4785
y6
y5
b5
b8
1028.547
b7 2+
462.7429
y7
881.4728
y2
284.1717
y4
582.2878
Peptide Sequence
Protein Sequence
- P L E E A L D V I V S T F H K -

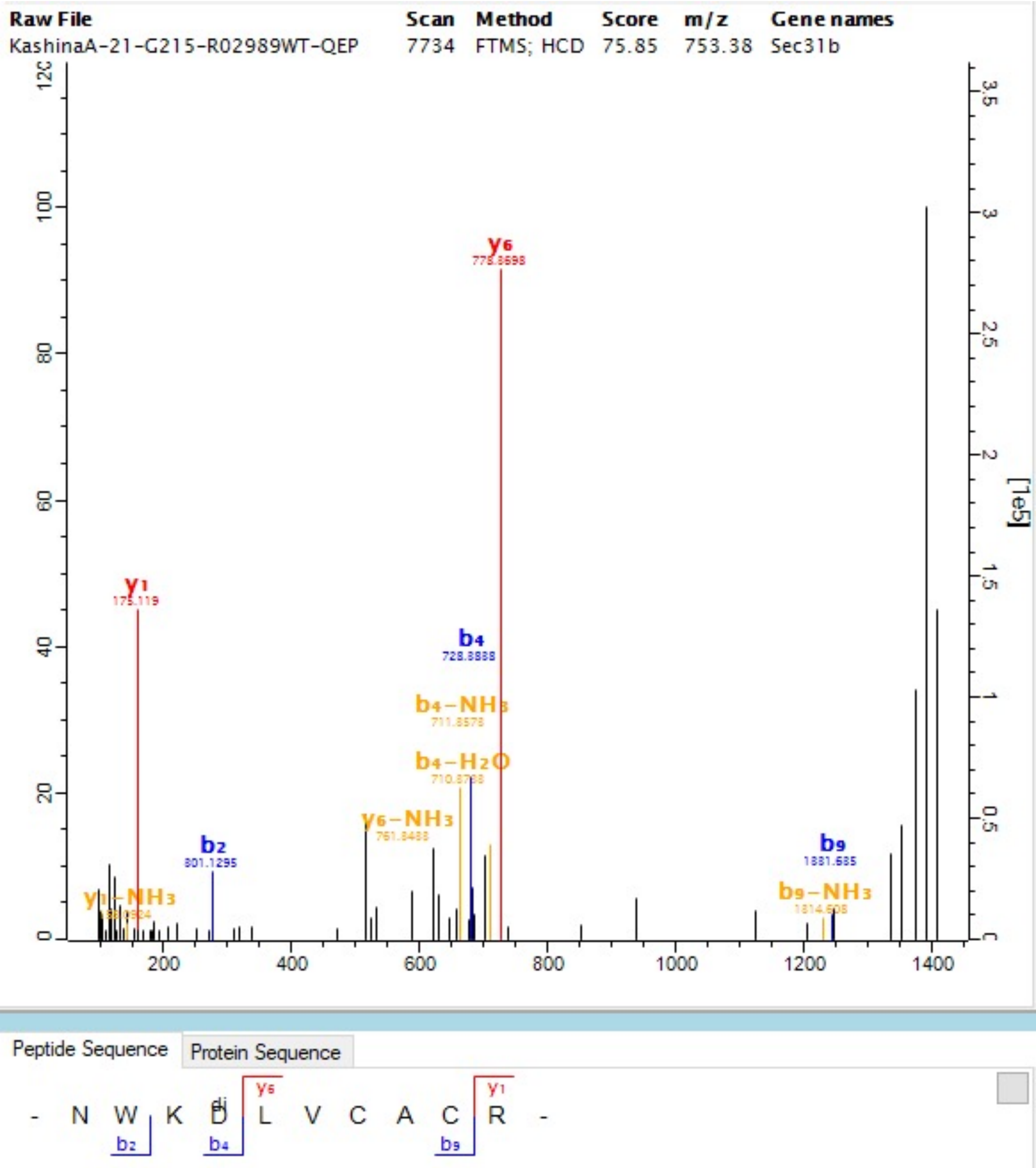
Raw File
KashinaA-21-G215-R02989WT-QEP
Scan
7734
Method
FTMS; HCD
Score
75.85
m/z
753.38
Gene names
Sec31b
y6
y1
175.119
b4
728.8888
b4-NH3
711.8578
b4-H2O
y6-NH3
761.8488
b2
801.1295
b9
1881.685
b9-NH3
y1-NH3
[1e5]
Peptide Sequence
Protein Sequence
- N W K D L V C A C R -
b2
b4
b9
y6
y1

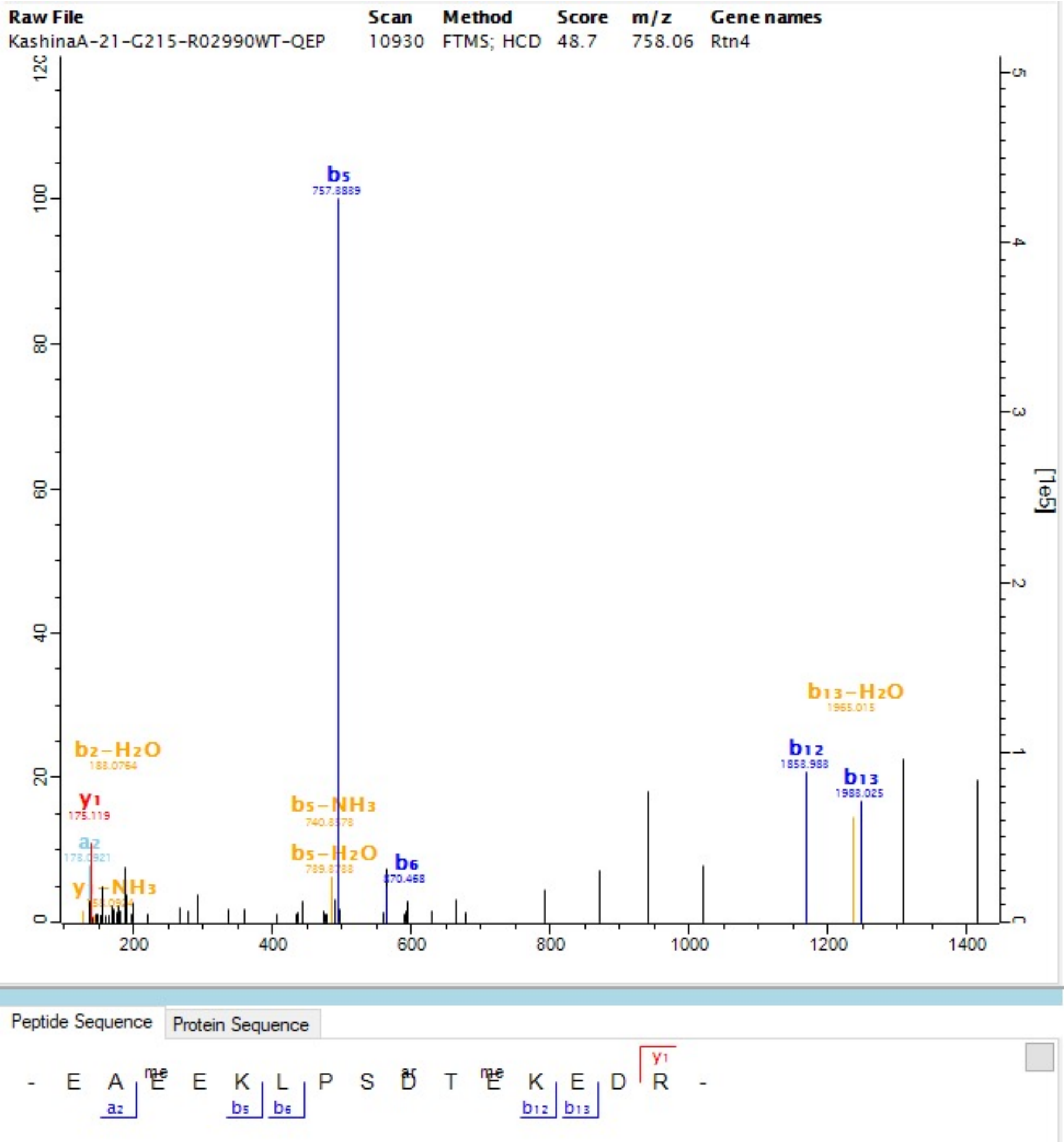
Raw File
KashinaA-21-G215-R02990WT-QEP
Scan
10930
Method
FTMS; HCD
Score
48.7
m/z
758.06
Gene names
Rtn4
b5
757.8889
b13-H2O
1965.015
b2-H2O
183.0764
b12
1858.988
b13
1988.025
y1
175.119
b5-NH3
740.8378
a2
178.0921
b5-H2O
739.8788
b6
870.468
[1e5]
Peptide Sequence
Protein Sequence
- E A E E K L P S D T E K E D R -
me
ar
me
a2
b5
b6
b12
b13
y1

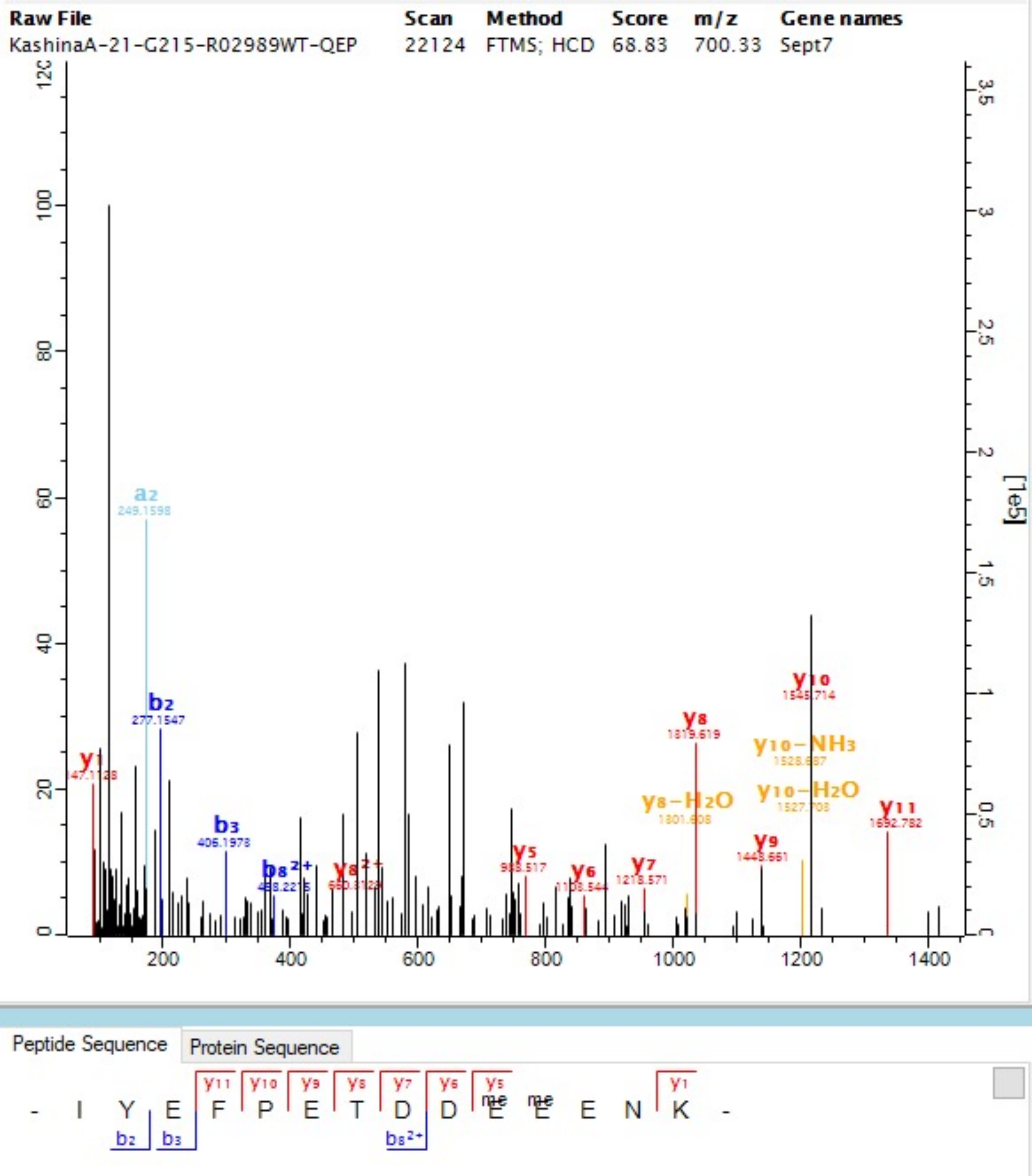

Raw File
KashinaA-21-G215-R02989WT-QEP
Scan
22124
Method
FTMS; HCD
Score
68.83
m/z
700.33
Gene names
Sept7
[1e5]
a2
249.1598
b2
277.1547
b3
406.1978
y1
147.1128
b8 2+
y8 2+
y5
y6
y7
y8
y8-H2O
y9
y10
y10-NH3
y10-H2O
y11
Peptide Sequence
Protein Sequence
- I Y E F P E T D D E E E N K -
me me

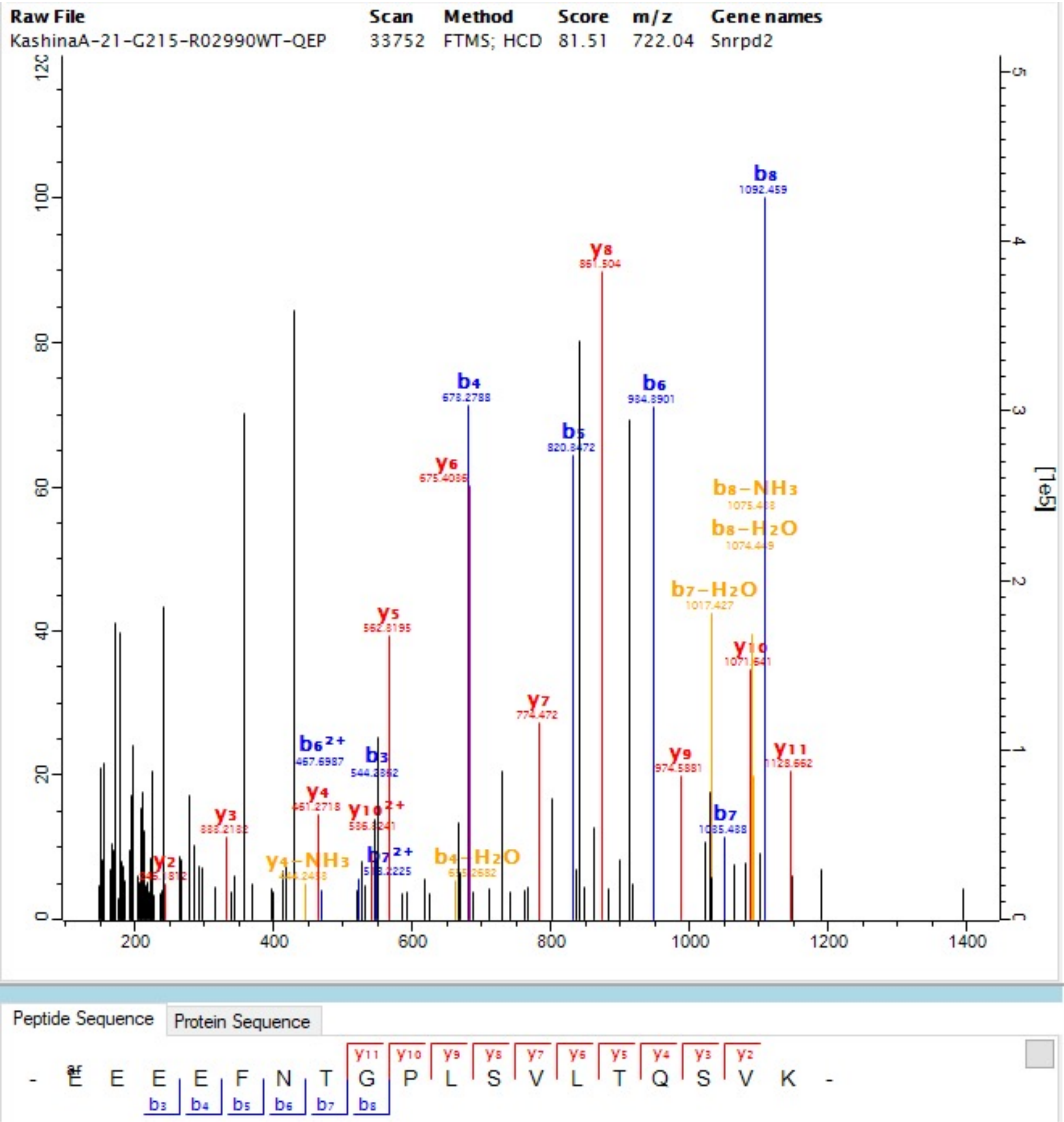
Raw File
KashinaA-21-G215-R02990WT-QEP
Scan
33752
Method
FTMS; HCD
Score
81.51
m/z
722.04
Gene names
Snrpd2
b8
1092.459
y8
861.504
b4
678.2788
b6
984.3901
b5
820.3472
y6
675.4086
b8-NH3
1075.438
b8-H2O
1074.449
b7-H2O
1017.427
y5
562.3195
y10
1071.641
y7
774.472
b6 2+
467.6987
b3
544.2362
y9
974.5881
y11
1128.662
y4
461.2718
y3
333.2182
y10 2+
536.3241
b7
1035.488
b7 2+
518.2225
y2
b4-H2O
y4-NH3
[1e5]
Peptide Sequence
Protein Sequence
- E E E E F N T G P L S V L T Q S V K -
y11 y10 y9 y8 y7 y6 y5 y4 y3 y2
b3 b4 b5 b6 b7 b8

Raw File	Scan	Method	Score	m/z	Gene names
KashinaA-21-G215-R02988WT-QEP	46872	FTMS; HCD	71.43	794.4	Sptbn1

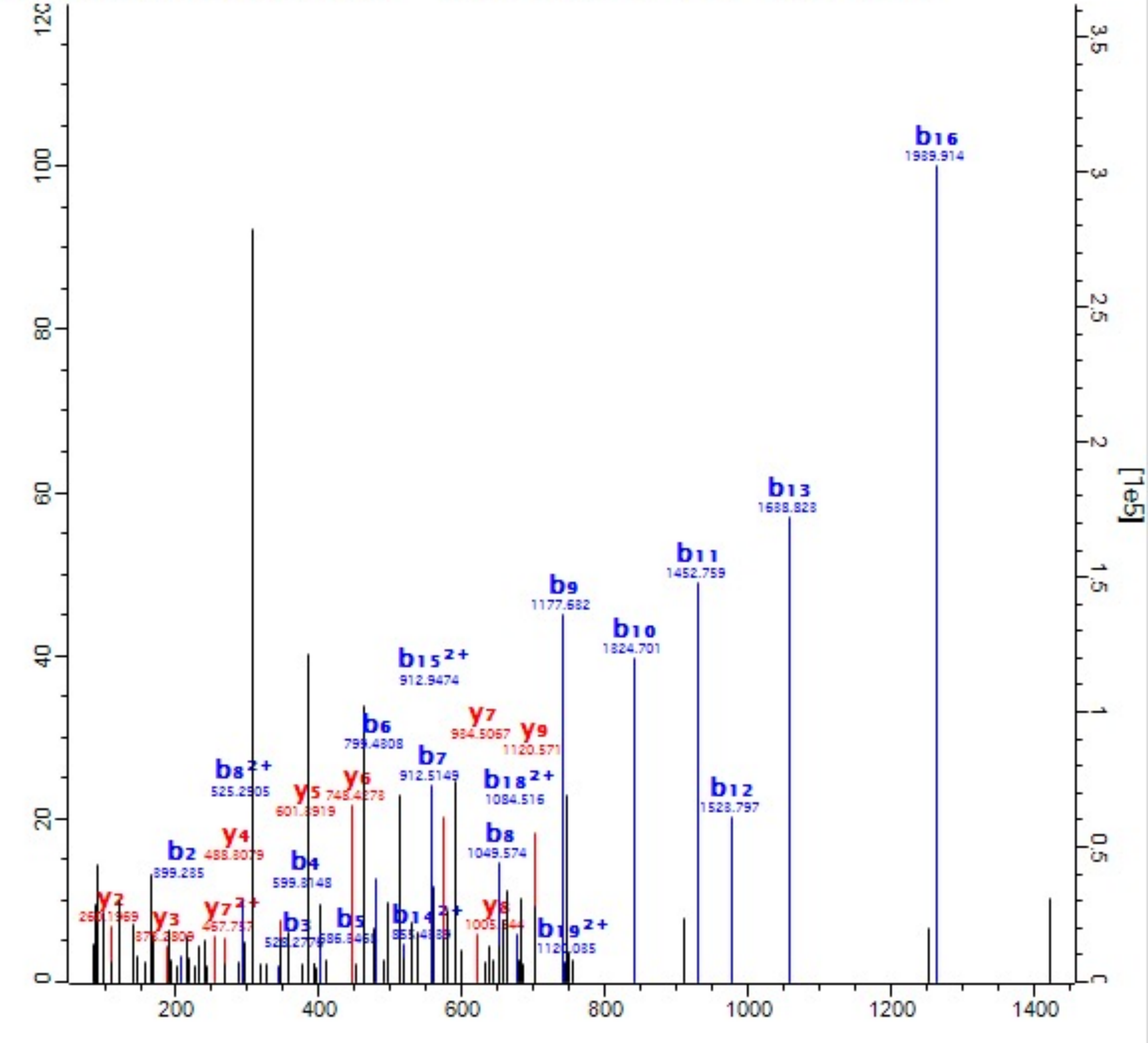

Peptide Sequence | Protein Sequence

- L E(ac) E A S L L H Q F Q A D A D D I D A W M(ox) L D I L K -

b2 b3 b4 b5 b6 b7 b8 b9 b10 b11 b12 b13 b14² b15² b16 b18² b19²

y9 y8 y7 y6 y5 y4 y3 y2

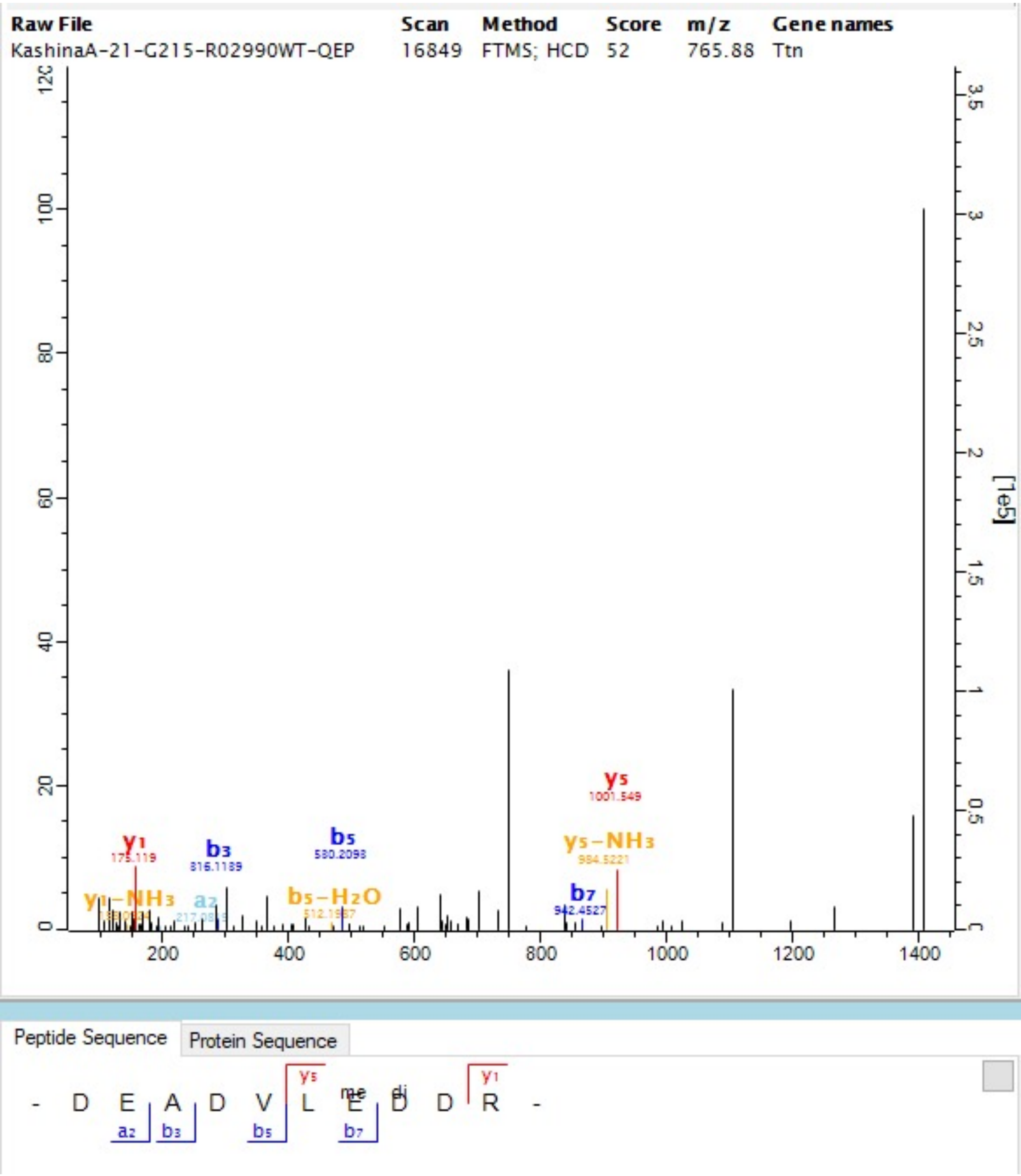

Raw File
KashinaA-21-G215-R02990WT-QEP
Scan
16849
Method
FTMS; HCD
Score
52
m/z
765.88
Gene names
Ttn
[1e5]
y1
175.119
b3
316.1189
b5
580.2098
y5
1001.549
y5-NH3
984.5221
b7
942.4527
y1-NH3
a2
b5-H2O
Peptide Sequence
Protein Sequence
- D E A D V L E D D R -
me
di
a2
b3
b5
b7
y5
y1

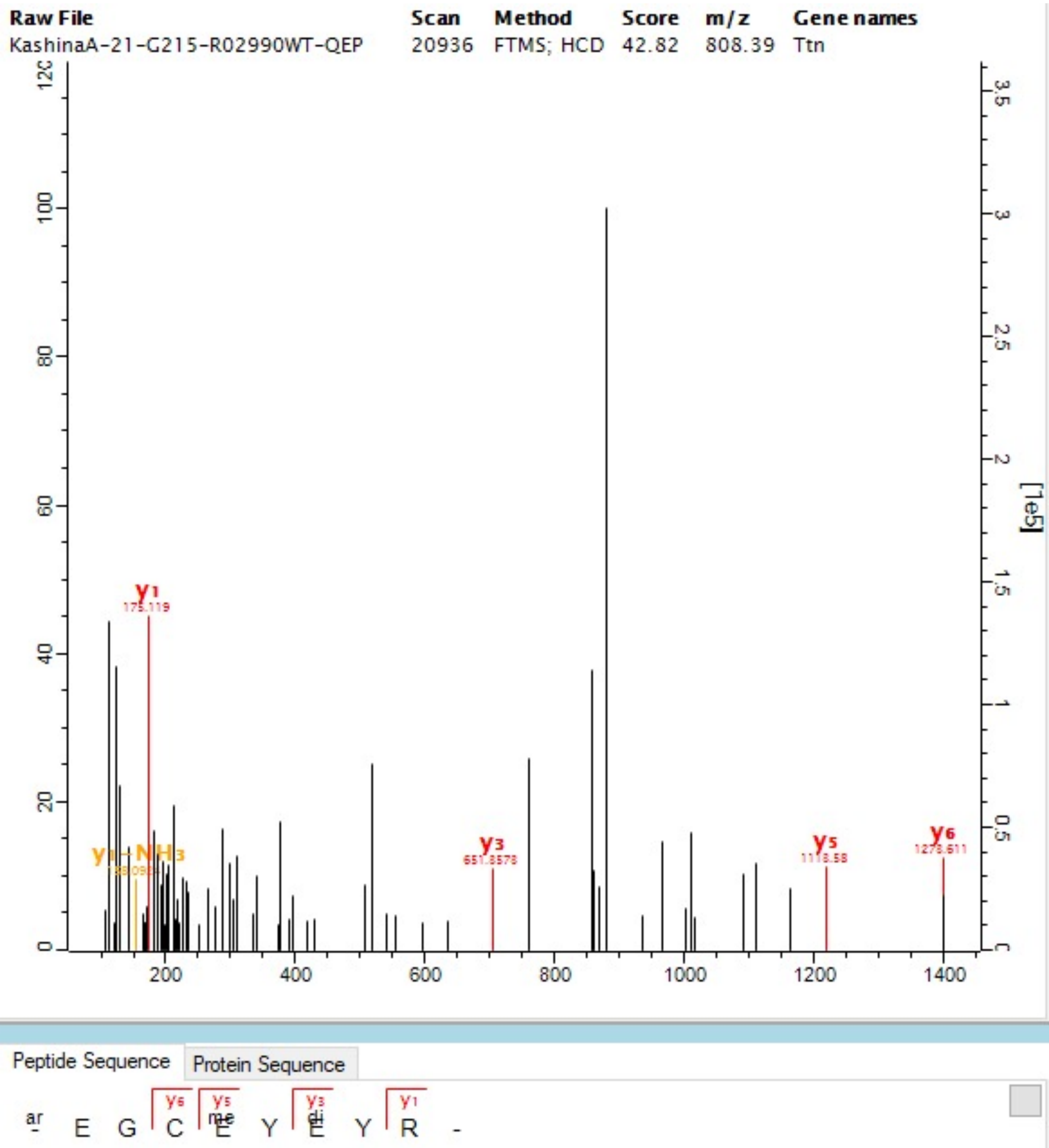

Raw File
KashinaA-21-G215-R02990WT-QEP
Scan
20936
Method
FTMS; HCD
Score
42.82
m/z
808.39
Gene names
Ttn
y1
175.119
y1-NH3
y3
y5
y6
[1e5]
Peptide Sequence
Protein Sequence
E G C E Y E Y R

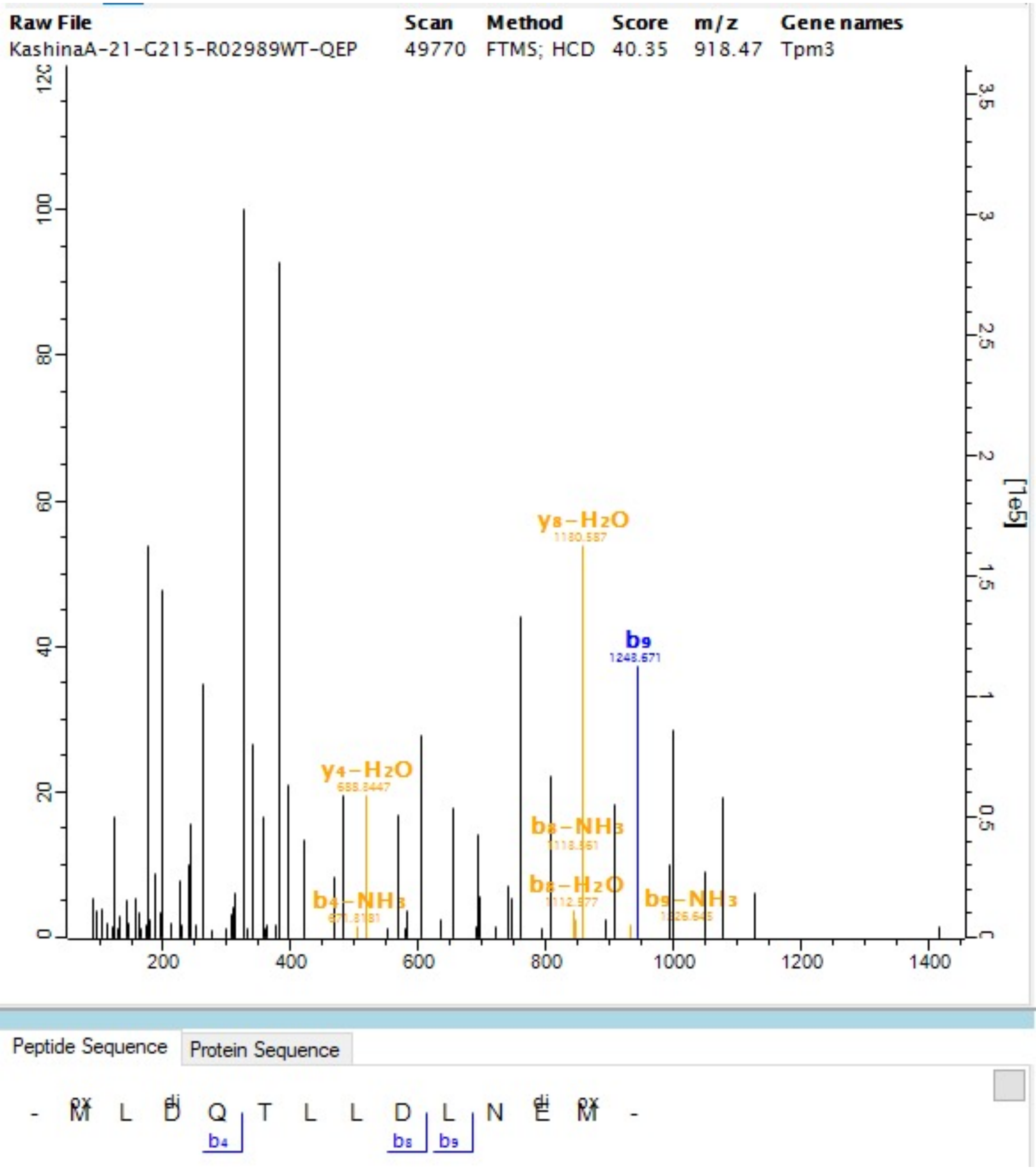
Raw File
KashinaA-21-G215-R02989WT-QEP
Scan
49770
Method
FTMS; HCD
Score
40.35
m/z
918.47
Gene names
Tpm3
y_8-H_2O
1180.587
b_9
1248.671
y_4-H_2O
b_8-NH_3
b_8-H_2O
b_4-NH_3
b_9-NH_3
[1e5]
Peptide Sequence
Protein Sequence
- M L D Q T L L D L N E M -
ox
di
di
ox
b_4
b_8
b_9

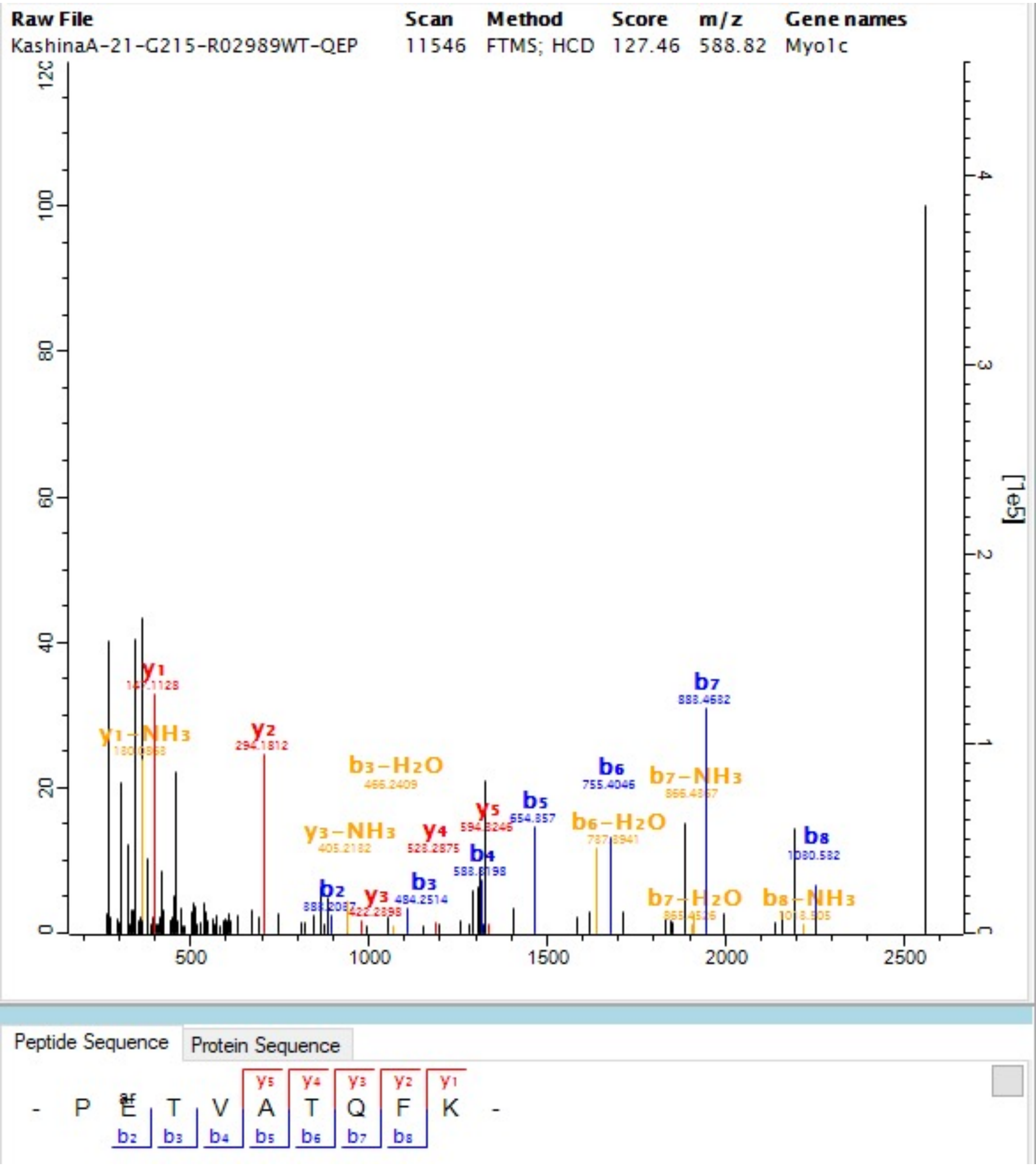

Raw File
KashinaA-21-G215-R02989WT-QEP
Scan
11546
Method
FTMS; HCD
Score
127.46
m/z
588.82
Gene names
Myo1c
[1e5]
y1
y1-NH3
y2
294.1812
b3-H2O
466.2409
y3-NH3
405.2182
y4
528.2875
y5
b5
654.857
b6
755.4046
b7-NH3
b6-H2O
b7
888.4682
b8
1080.582
b4
b3
484.2514
b2
y3
b7-H2O
b8-NH3
Peptide Sequence
Protein Sequence
- P E T V A T Q F K -
