# Supplementary material for: Global Analysis of Post-Translational Side-Chain Arginylation Using Pan-Arginylation Antibodies
Source: Mol Cell Proteomics. 2023 Oct 12;22(11):100664. doi: 10.1016/j.mcpro.2023.100664 (PMC10656225; doi:10.1016/j.mcpro.2023.100664)
Supplement: Supplemental File 3 [file mmc3.pdf]

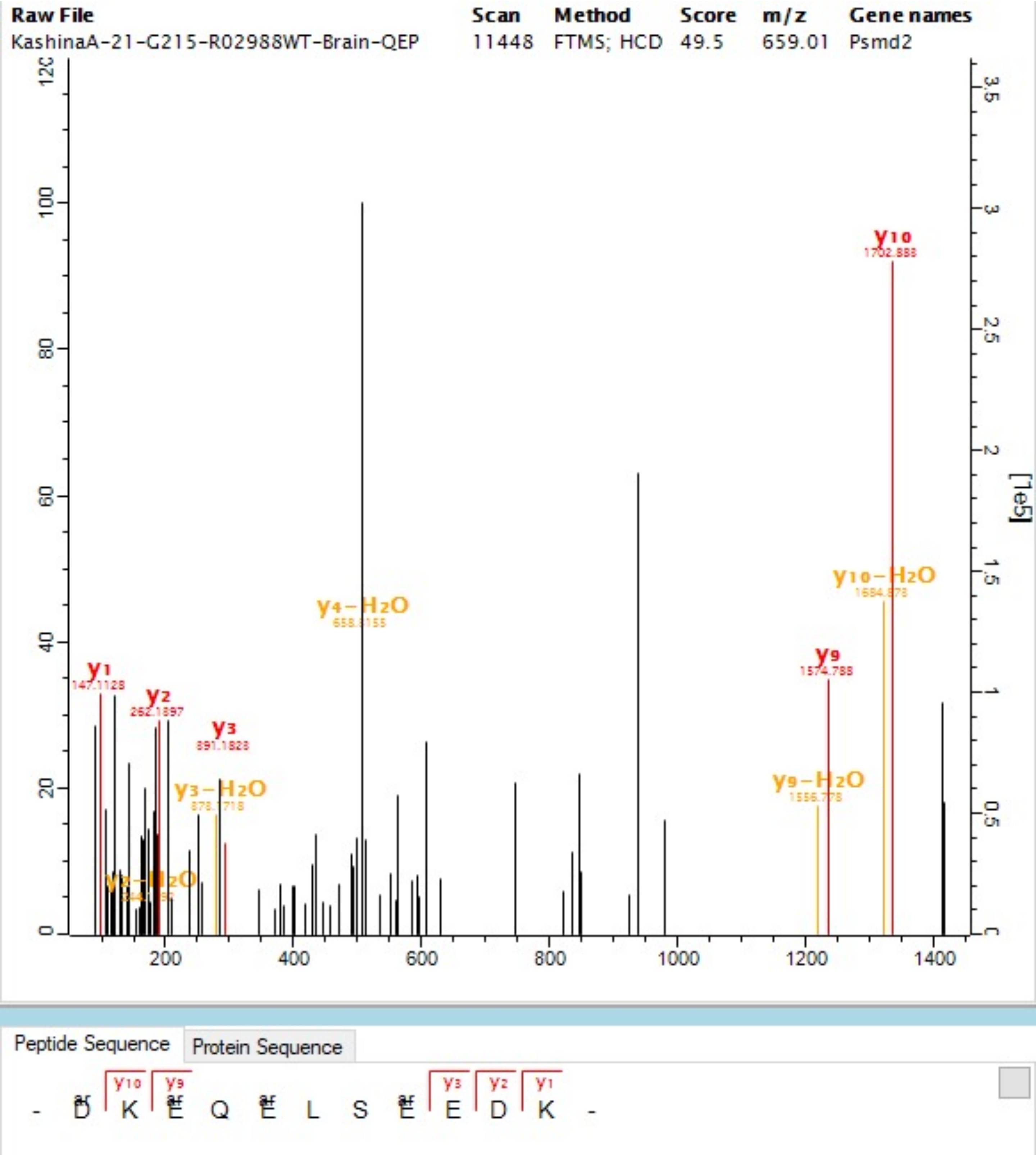
Raw File
KashinaA-21-G215-R02988WT-Brain-QEP
Scan
11448
Method
FTMS; HCD
Score
49.5
m/z
659.01
Gene names
Psmd2
y10
1702.888
y10−H2O
y9
1574.788
y9−H2O
y4−H2O
y1
147.1128
y2
262.1397
y3
391.1828
y3−H2O
y2−H2O
[1e5]
Peptide Sequence
Protein Sequence
- D K E Q E L S E E D K -

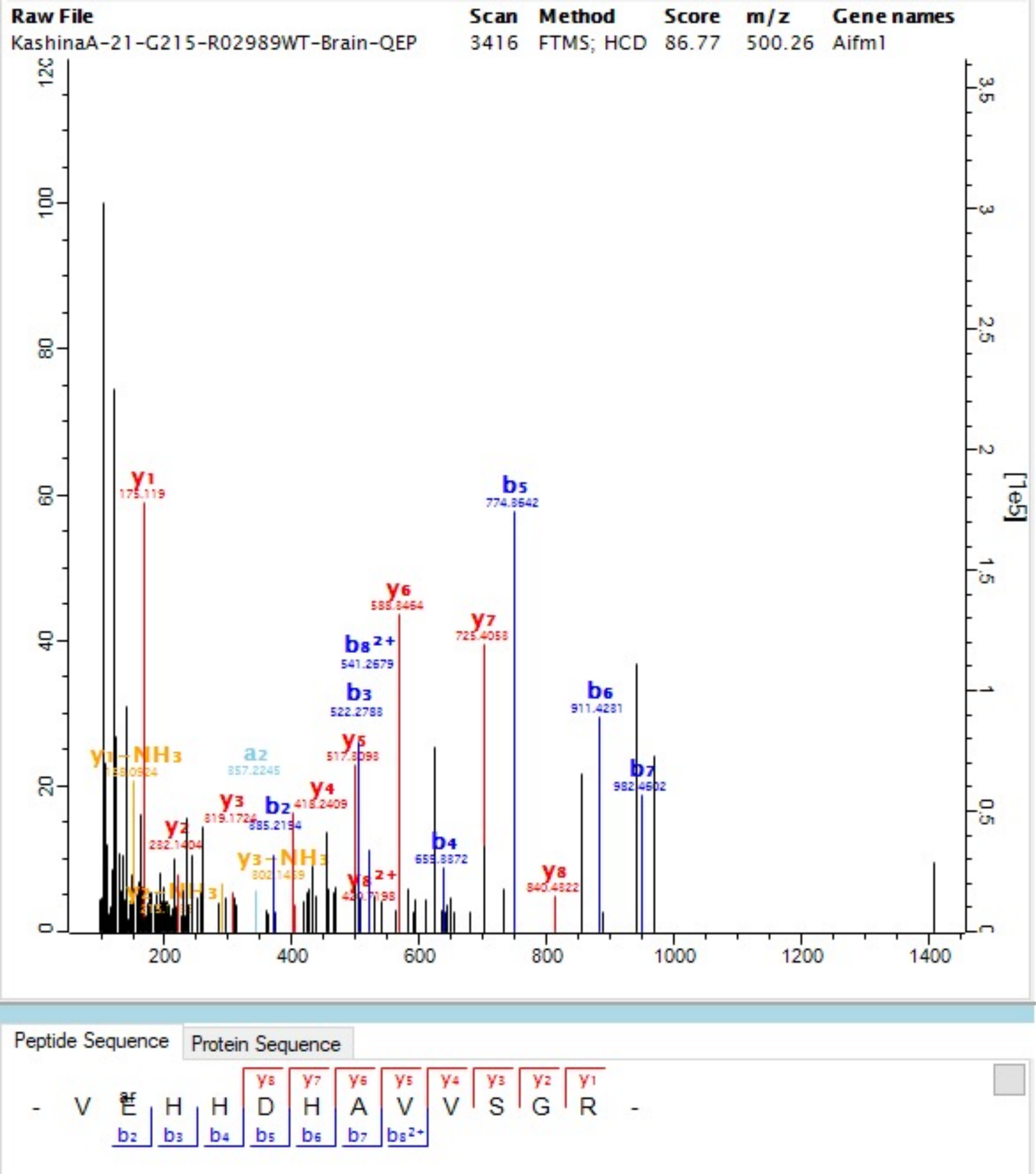
Raw File
KashinaA-21-G215-R02989WT-Brain-QEP
Scan
3416
Method
FTMS; HCD
Score
86.77
m/z
500.26
Gene names
Aifm1
[1e5]
y1
y2
y3
y4
y5
y6
y7
y8
y8 2+
b2
b3
b4
b5
b6
b7
b8 2+
a2
y1-NH3
y3-NH3
200
400
600
800
1000
1200
1400
Peptide Sequence
Protein Sequence
- V E H H D H A V V S G R -

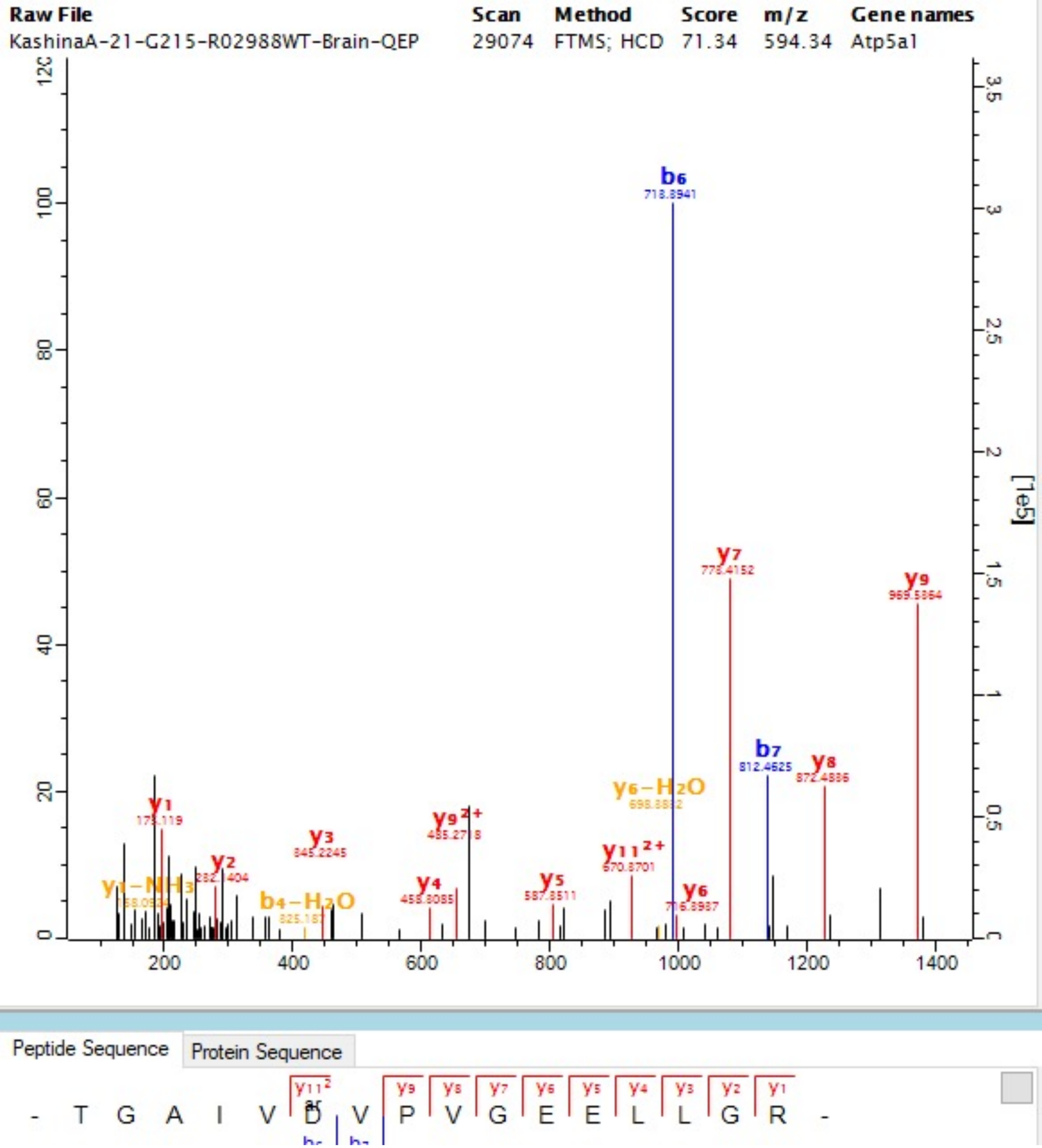

Raw File
KashinaA-21-G215-R02988WT-Brain-QEP
Scan
29074
Method
FTMS; HCD
Score
71.34
m/z
594.34
Gene names
Atp5a1
[1e5]
b6
718.3941
y7
778.4152
y9
969.5864
b7
812.4625
y8
872.4886
y6-H2O
698.8832
y1
175.119
y9 2+
485.2718
y3
345.2245
y2
232.1404
y11 2+
570.8701
y4
458.3085
y5
587.3511
y6
716.3937
y1-NH3
b4-H2O
325.187
Peptide Sequence
Protein Sequence
- T G A I V D V P V G E E L L G R -

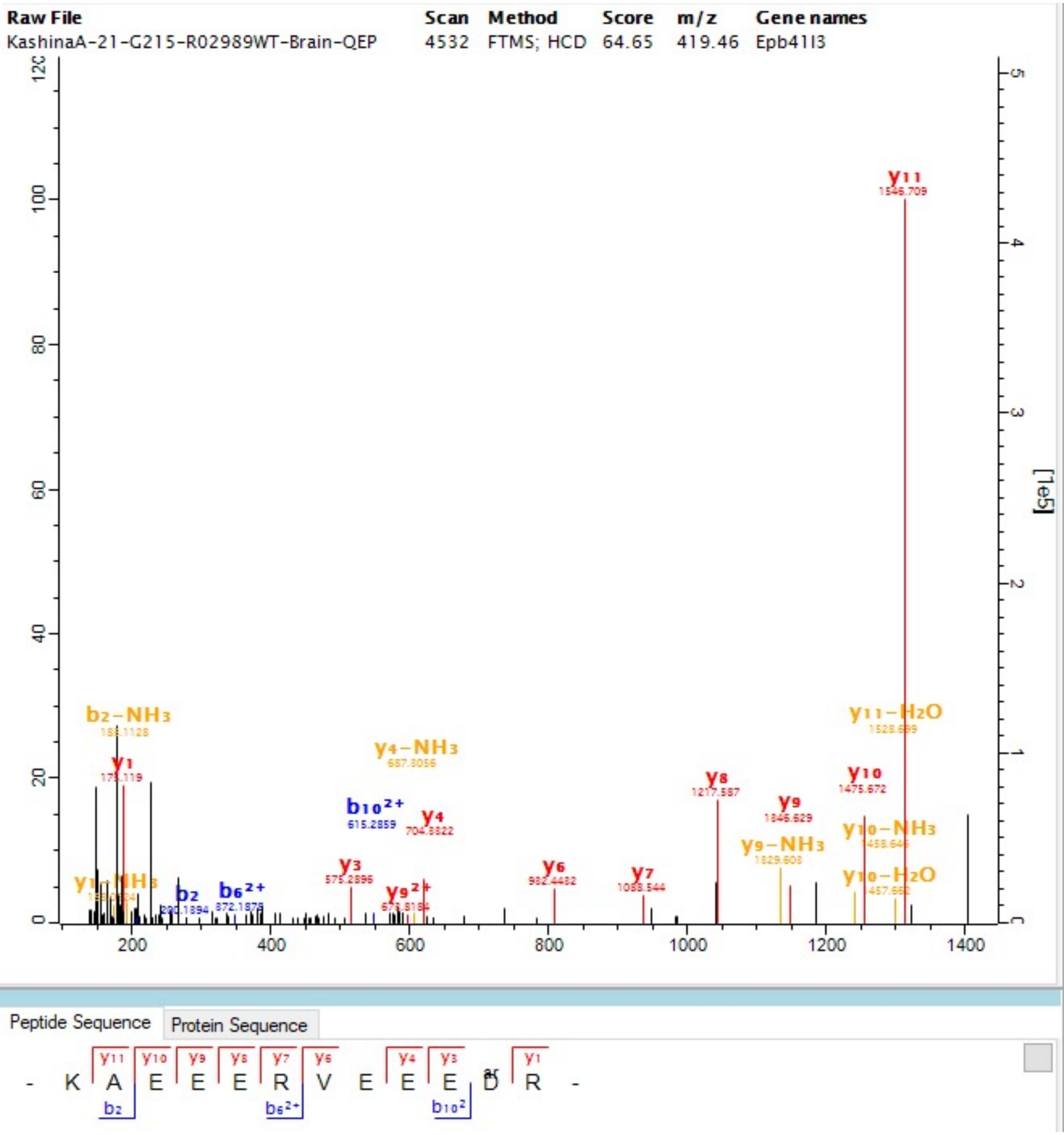
Raw File
KashinaA-21-G215-R02989WT-Brain-QEP
Scan
4532
Method
FTMS; HCD
Score
64.65
m/z
419.46
Gene names
Epb41l3
[1e5]
y11
b2-NH3
y11-H2O
y4-NH3
y1
y8
y10
b10²⁺
615.2859
y4
y9
y10-NH3
y9-NH3
y3
y6
y7
1217.587
1088.544
575.2896
y10-H2O
b6²⁺
872.1878
b2
y9²⁺
200
400
600
800
1000
1200
1400
Peptide Sequence
Protein Sequence
- K A E E E R V E E E D R -

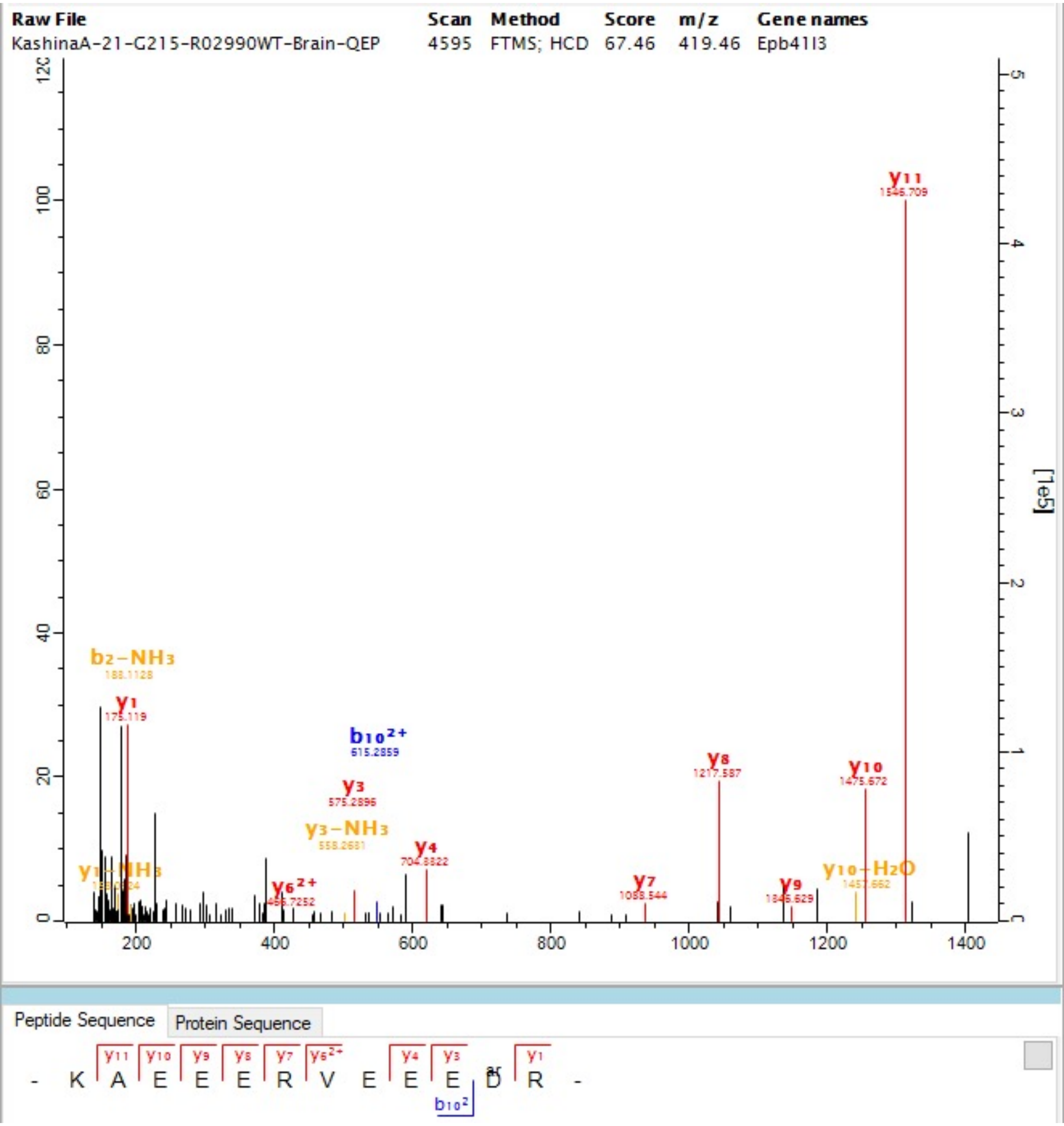
Raw File
KashinaA-21-G215-R02990WT-Brain-QEP
Scan
4595
Method
FTMS; HCD
Score
67.46
m/z
419.46
Gene names
Epb41l3
y11
1546.709
b2-NH3
188.1128
y1
175.119
b10 2+
615.2859
y8
1217.587
y10
1475.672
y3
575.2896
y3-NH3
558.2631
y4
704.3322
y6 2+
[1e5]
y7
1088.544
y9
y10-H2O
1457.662
Peptide Sequence
Protein Sequence
- K A E E E R V E E E D R -

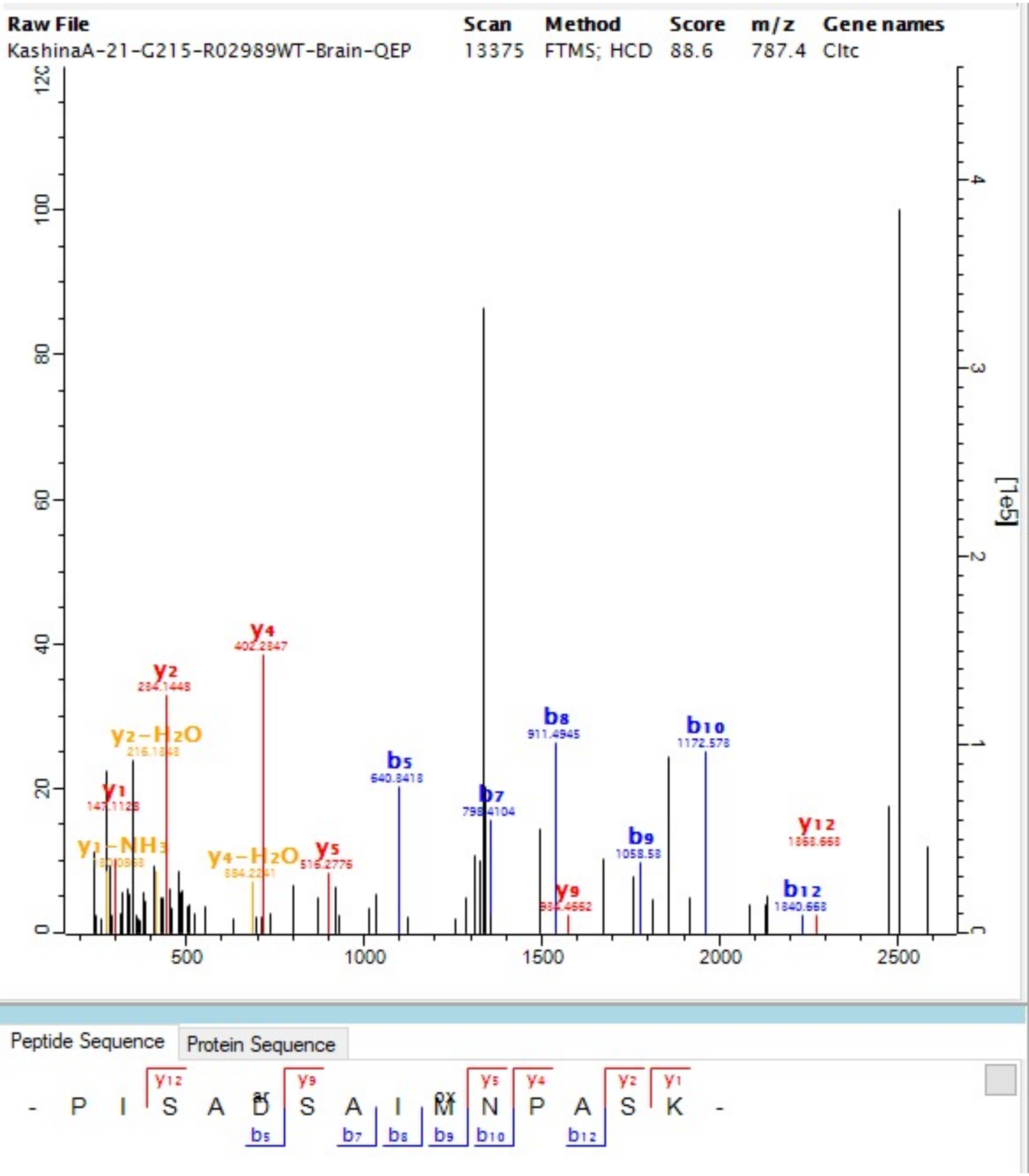

Raw File
KashinaA-21-G215-R02989WT-Brain-QEP
Scan
13375
Method
FTMS; HCD
Score
88.6
m/z
787.4
Gene names
Cltc
[1e5]
y4
402.2847
y2
y2-H2O
y1
y1-NH3
y4-H2O
y5
b5
b7
b8
911.4945
b9
1058.58
b10
1172.578
y9
y12
b12
Peptide Sequence
Protein Sequence
- P I S A D S A I M N P A S K -

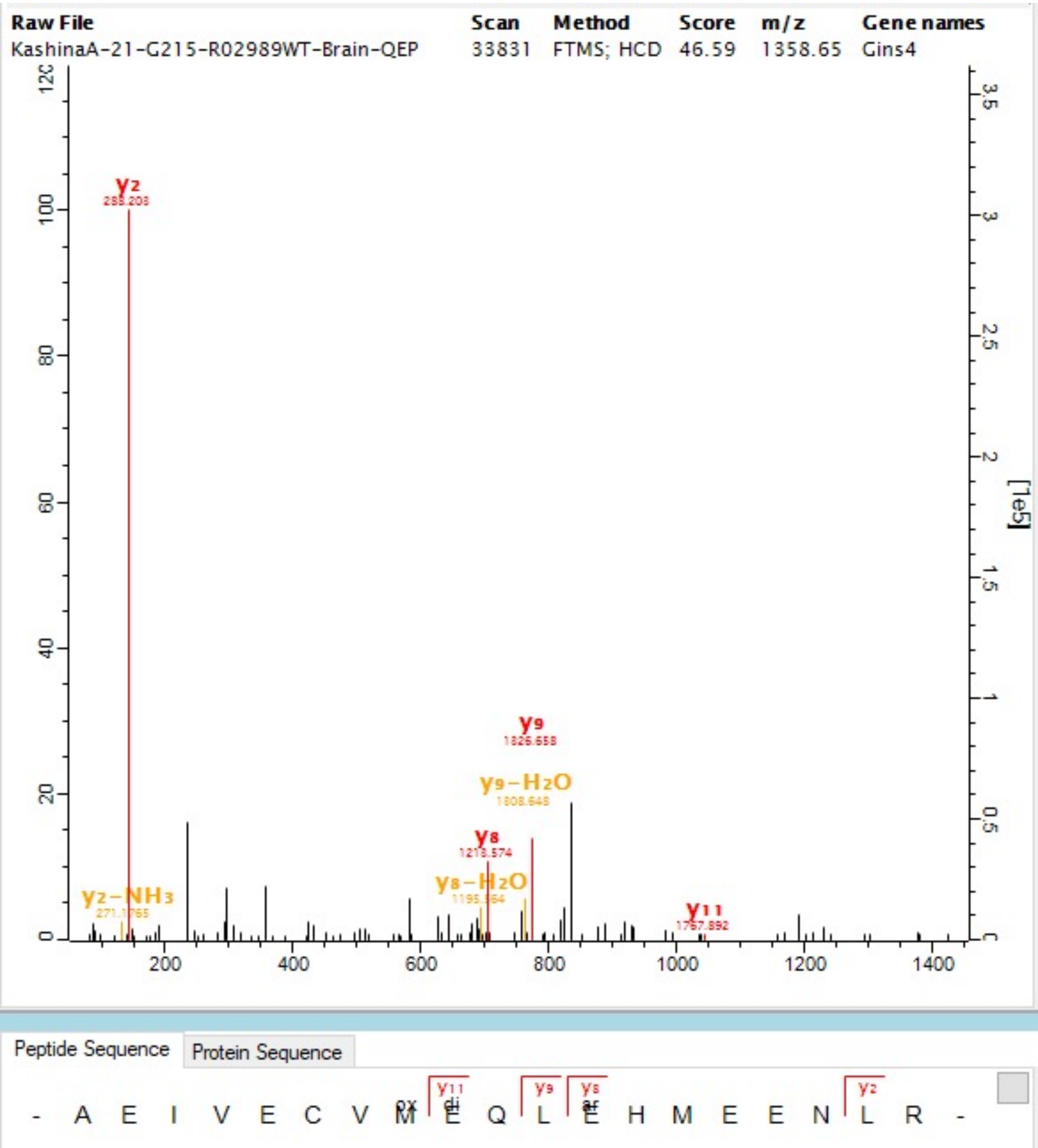

Raw File
KashinaA-21-G215-R02989WT-Brain-QEP
Scan
33831
Method
FTMS; HCD
Score
46.59
m/z
1358.65
Gene names
Gins4
y2
y2-NH3
y8
y8-H2O
y9
y9-H2O
y11
[1e5]
Peptide Sequence
Protein Sequence
- A E I V E C V M E Q L E H M E E N L R -

Raw File	Scan	Method	Score	m/z	Gene names
KashinaA-21-G215-R02990WT-Brain-QEP	3560	FTMS; HCD	87.62	408.87	Dbn1

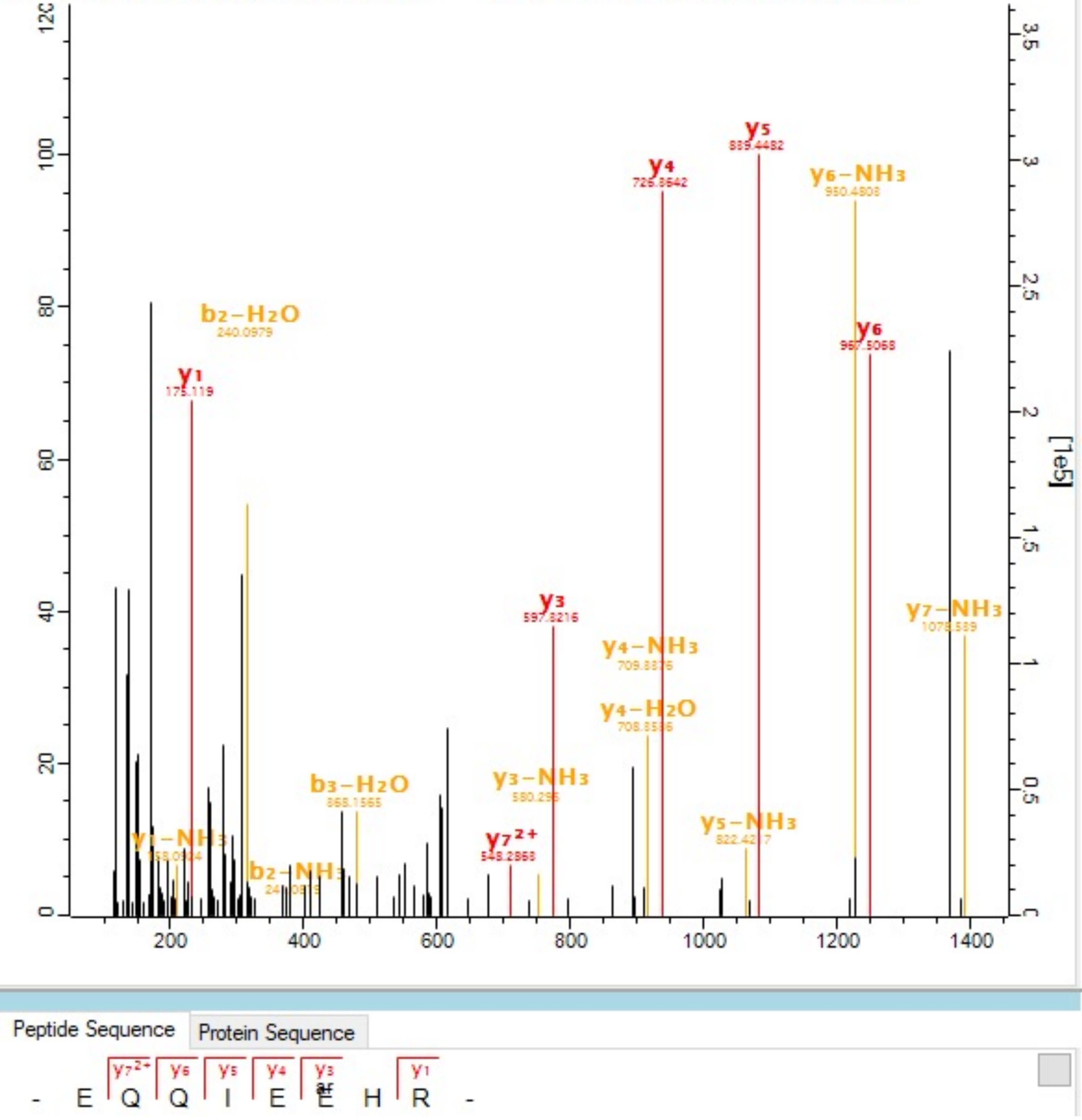

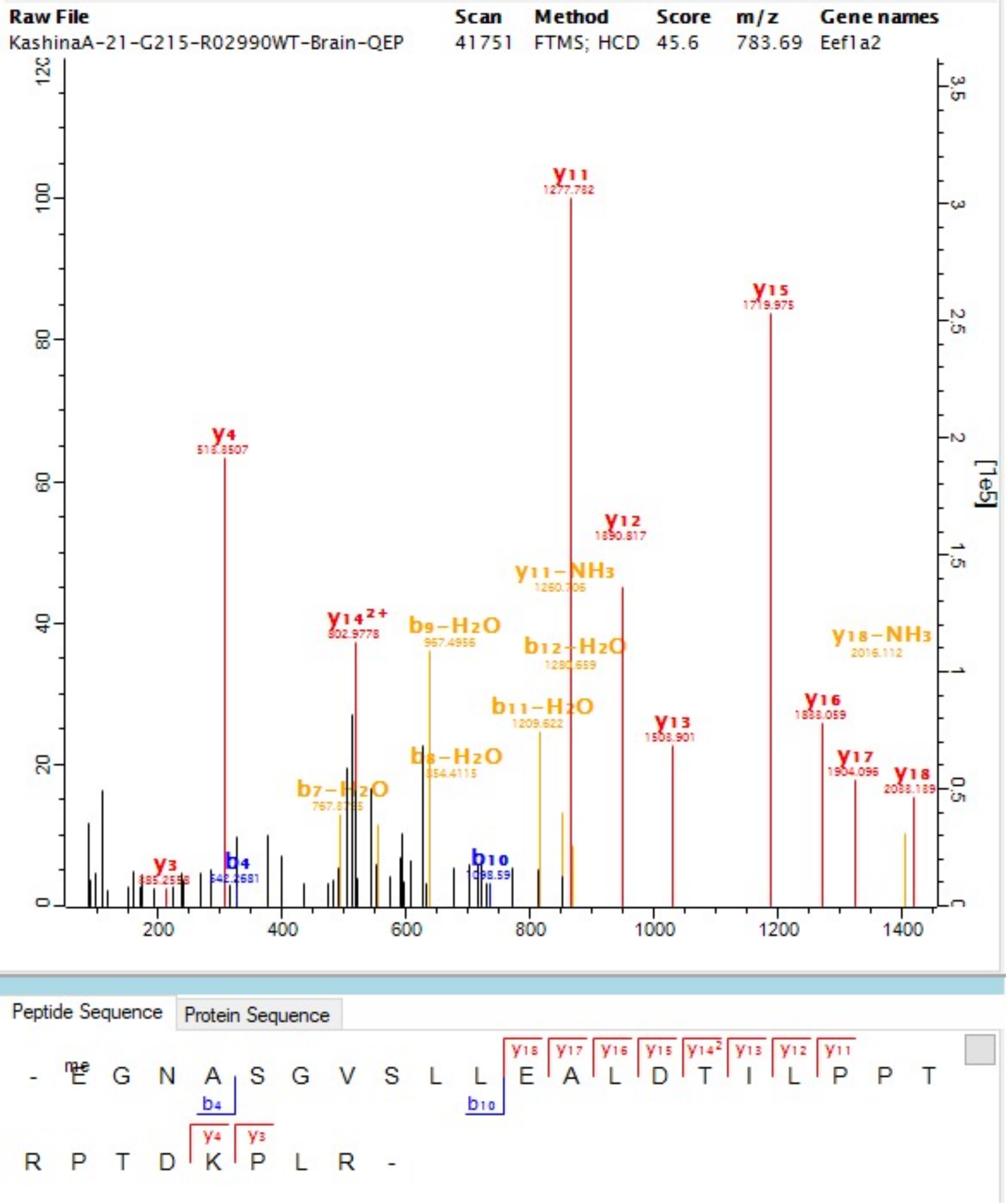

Raw File
KashinaA-21-G215-R02990WT-Brain-QEP
Scan
41751
Method
FTMS; HCD
Score
45.6
m/z
783.69
Gene names
Eef1a2
y11
1277.782
y15
y4
y12
y11-NH3
y14 2+
b9-H2O
b12-H2O
y18-NH3
2016.112
b11-H2O
1209.622
y13
y16
b8-H2O
y17
y18
b7-H2O
y3
b4
b10
Peptide Sequence
Protein Sequence
me
- E G N A S G V S L L E A L D T I L P P T
R P T D K P L R -

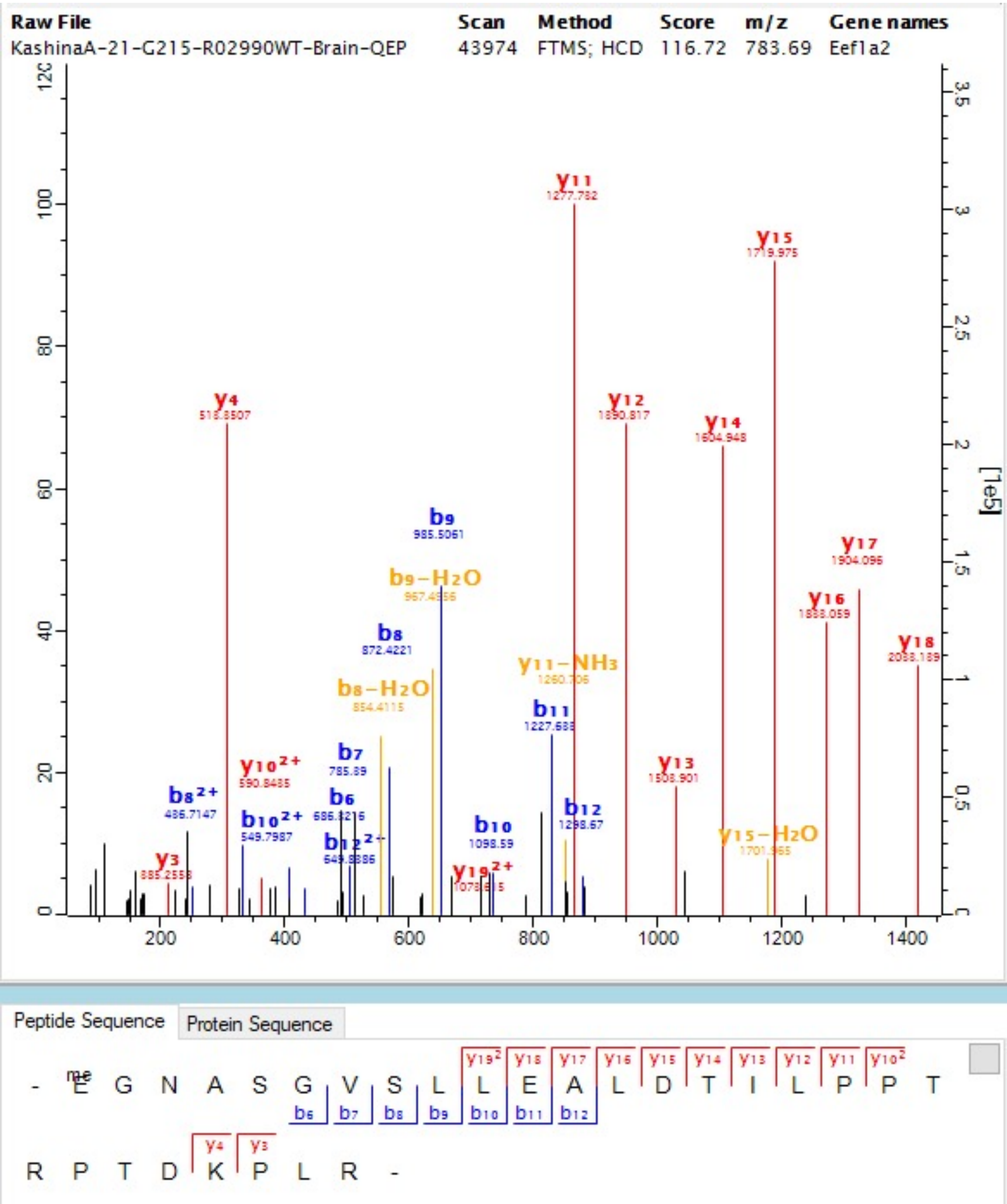

Raw File
KashinaA-21-G215-R02990WT-Brain-QEP
Scan
43974
Method
FTMS; HCD
Score
116.72
m/z
783.69
Gene names
Eef1a2
[1e5]
y11 1277.782
y15 1719.975
y4 518.3507
y12 1390.817
y14 1604.948
b9 985.5061
b9-H2O 967.4956
y17 1904.096
y16 1833.059
b8 872.4221
y18 2033.139
y11-NH3 1260.756
b8-H2O 854.4115
b11 1227.688
y10²⁺ 590.8485
b7 785.39
y13 1503.901
b8²⁺ 436.7147
b6 686.3216
b12 1298.67
b10²⁺ 549.7987
b10 1098.59
y15-H2O 1701.965
b12²⁺ 649.8386
y3 385.2558
y19²⁺ 1078.615
Peptide Sequence
Protein Sequence
- E G N A S G V S L L E A L D T I L P P T
me
R P T D K P L R -

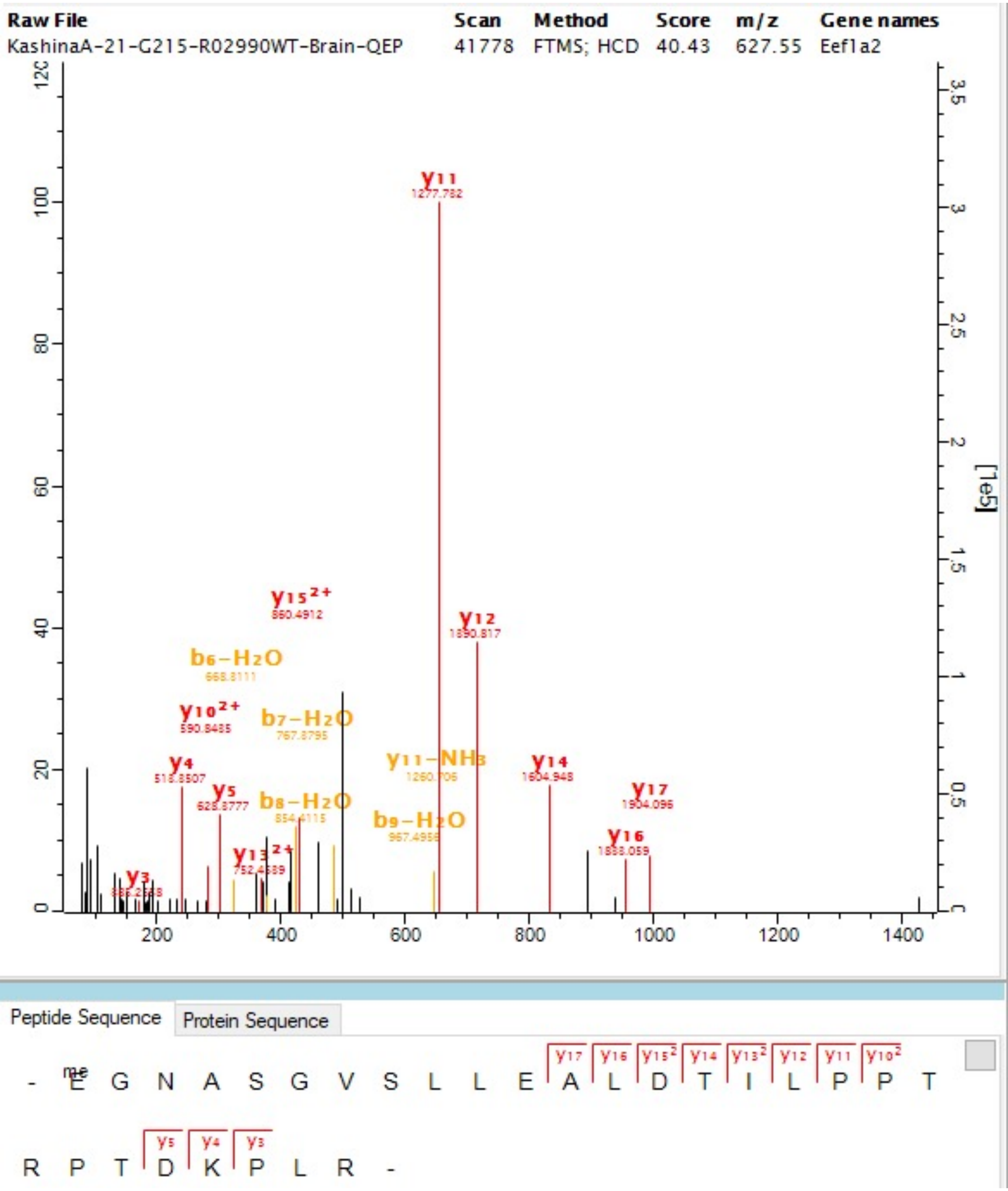
Raw File
KashinaA-21-G215-R02990WT-Brain-QEP
Scan
41778
Method
FTMS; HCD
Score
40.43
m/z
627.55
Gene names
Eef1a2
y11
1277.782
y12
1390.817
y15 2+
860.4912
b6-H2O
668.8111
y10 2+
590.8485
b7-H2O
767.8795
y11-NH3
1260.706
y14
1604.948
y4
518.8507
y5
628.8777
b8-H2O
854.4115
b9-H2O
967.4956
y17
1904.096
y16
1833.059
y13 2+
752.4389
y3
[1e5]
Peptide Sequence
Protein Sequence
- E G N A S G V S L L E A L D T I L P P T
R P T D K P L R -

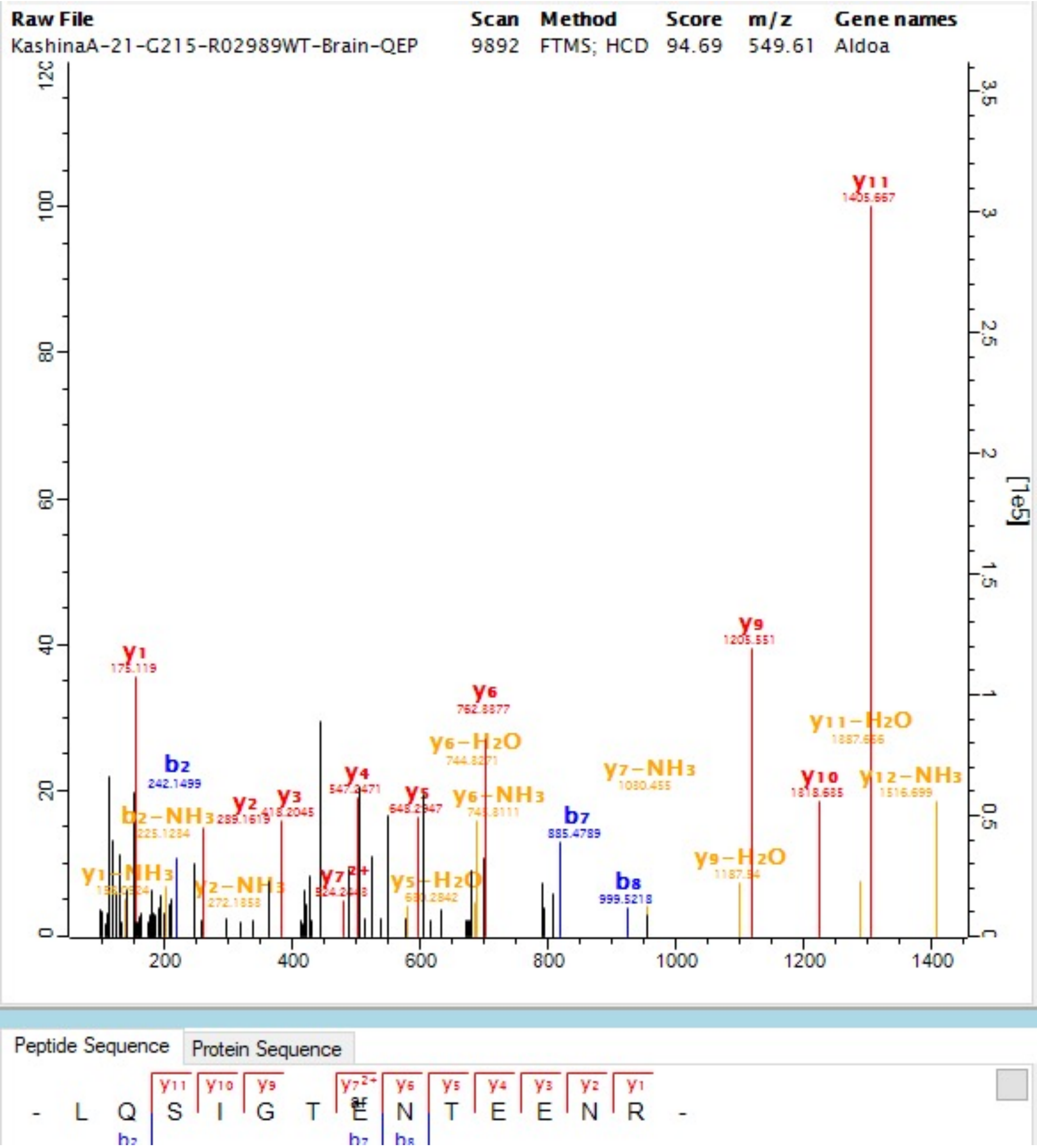

Raw File
KashinaA-21-G215-R02989WT-Brain-QEP
Scan
9892
Method
FTMS; HCD
Score
94.69
m/z
549.61
Gene names
Aldoa
[1e5]
y11
1405.667
y9
1205.551
y1
175.119
y6
762.8877
y11−H2O
y6−H2O
744.8271
y7−NH3
1030.455
b2
242.1499
y4
547.2471
y10
y12−NH3
1516.699
y2
y3
y5
y6−NH3
b2−NH3
225.1234
b7
885.4789
y9−H2O
1187.54
y1−NH3
y2−NH3
272.1858
y7 2+
y5−H2O
b8
999.5218
200
400
600
800
1000
1200
1400
Peptide Sequence
Protein Sequence
y11
y10
y9
y7 2+
y6
y5
y4
y3
y2
y1
- L Q S I G T E N T E E N R -
b2
b7
b8

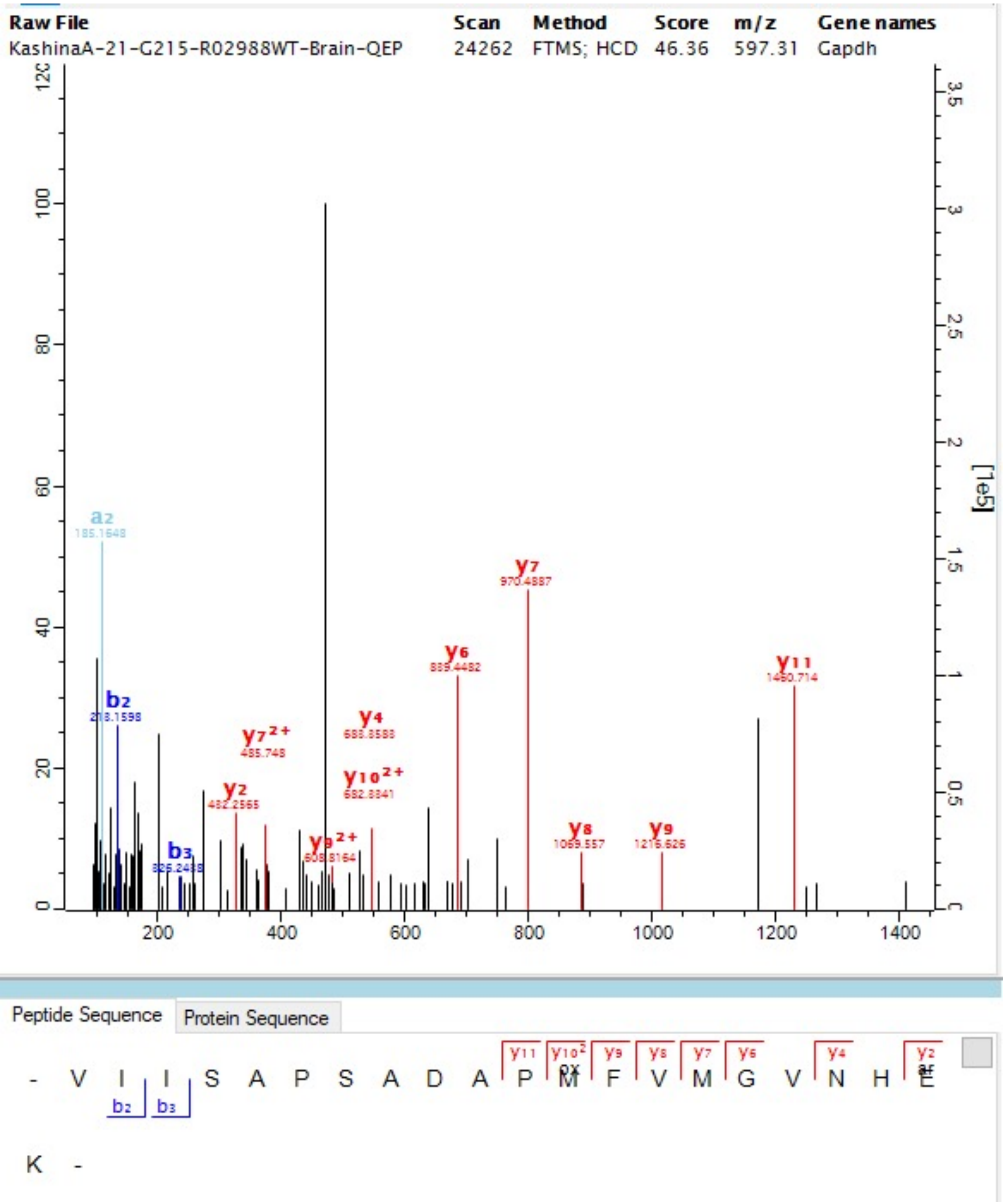
Raw File
KashinaA-21-G215-R02988WT-Brain-QEP
Scan
24262
Method
FTMS; HCD
Score
46.36
m/z
597.31
Gene names
Gapdh
[1e5]
a2
b2
b3
y2
y7 2+
y9 2+
y4
y10 2+
y6
y7
970.4887
y8
y9
y11
Peptide Sequence
Protein Sequence
- V I I S A P S A D A P M F V M G V N H E
K -
ox
y11 y10 2 y9 y8 y7 y6 y4 y2
b2 b3

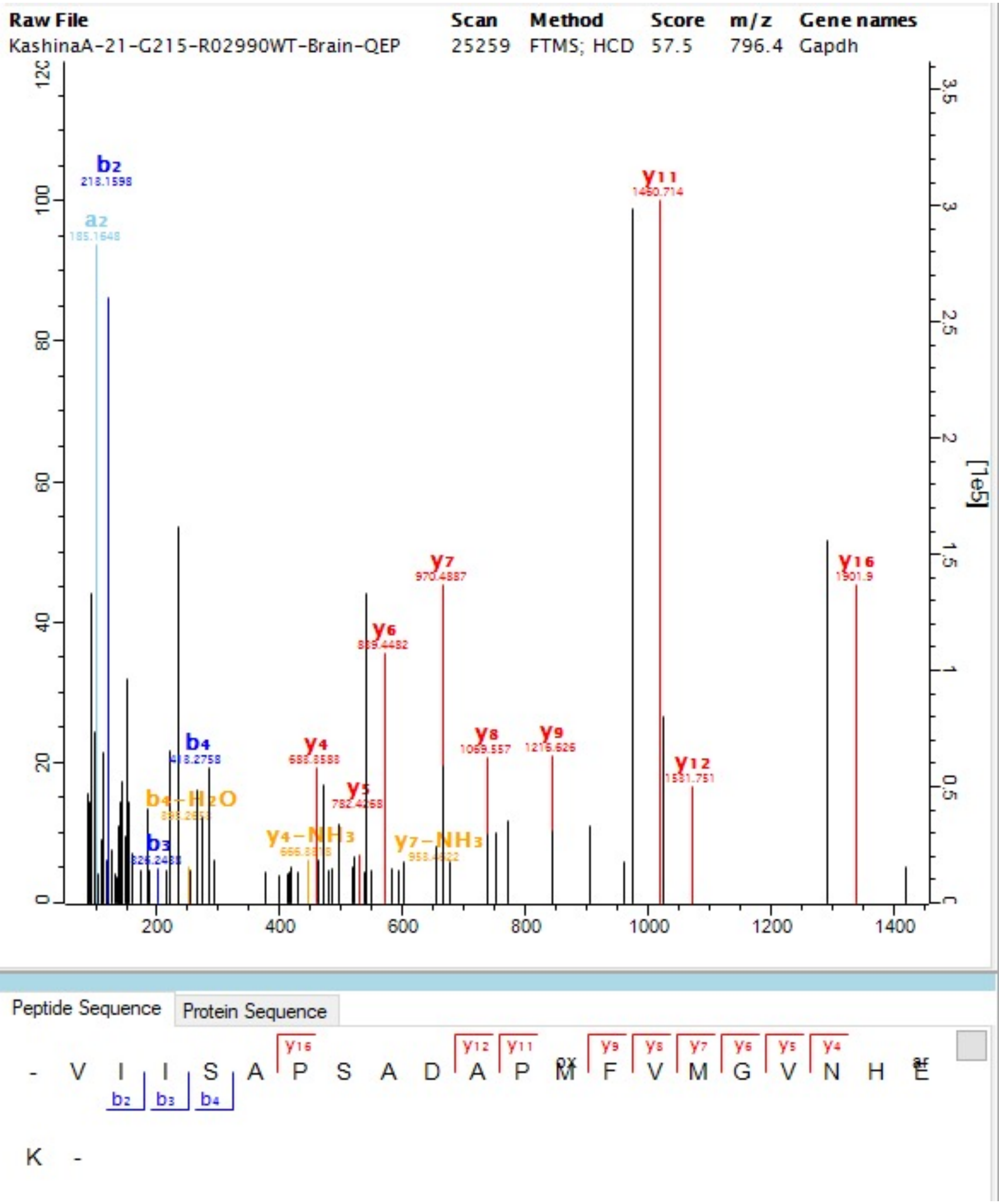

Raw File
KashinaA-21-G215-R02990WT-Brain-QEP
Scan
25259
Method
FTMS; HCD
Score
57.5
m/z
796.4
Gene names
Gapdh
b2
218.1598
a2
185.1648
y11
y7
970.4887
y16
y6
b4
418.2758
y4
y5
y8
y9
y12
b4-H2O
b3
y4-NH3
y7-NH3
[1e5]
Peptide Sequence
Protein Sequence
- V I I S A P S A D A P M F V M G V N H E
K -

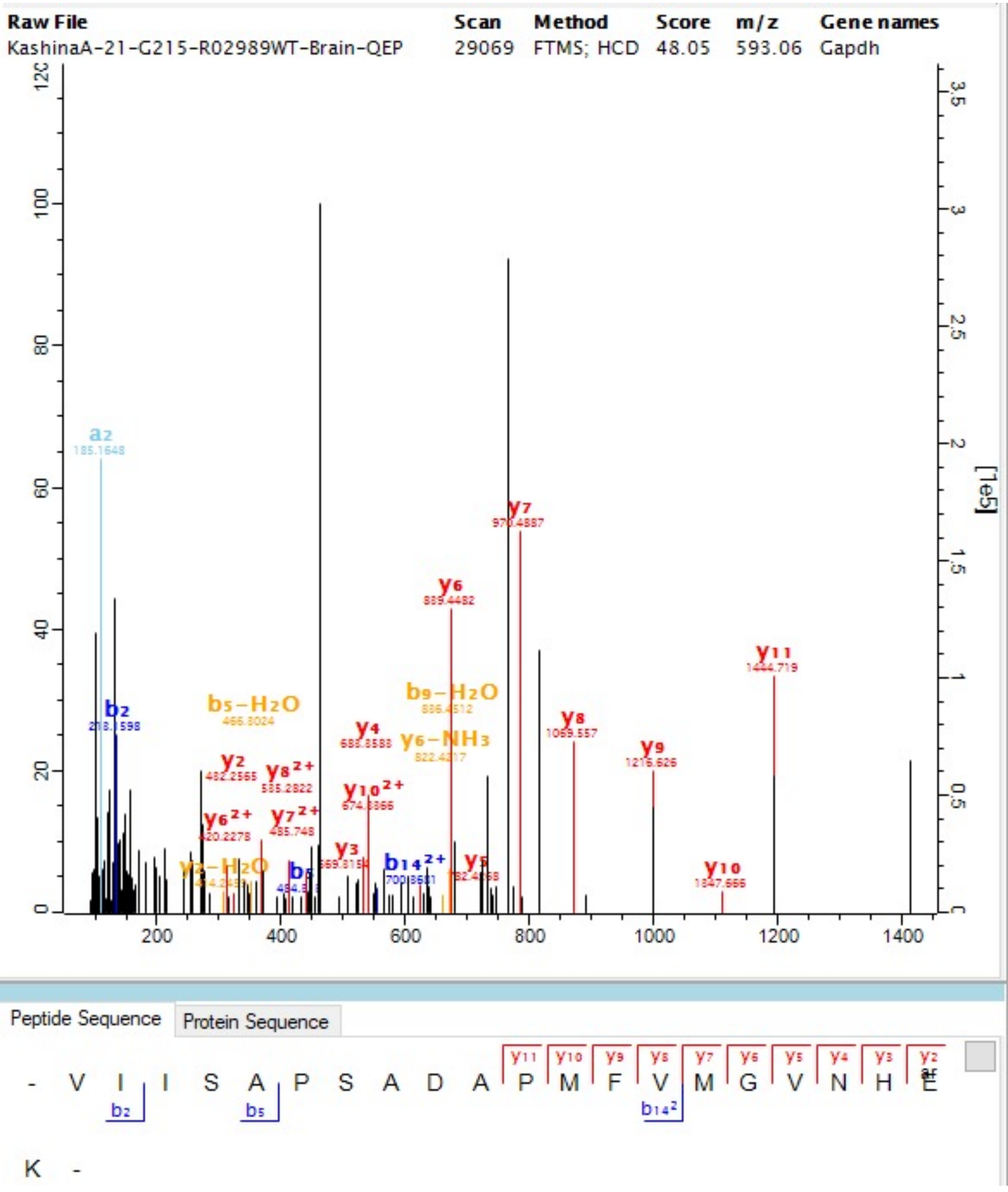
Raw File
KashinaA-21-G215-R02989WT-Brain-QEP
Scan
29069
Method
FTMS; HCD
Score
48.05
m/z
593.06
Gene names
Gapdh
[1e5]
a2
185.1648
b2
y7
970.4887
y6
839.4482
y11
1444.719
b5-H2O
466.3024
b9-H2O
836.4512
y4
683.8583
y6-NH3
822.4217
y8
1069.557
y9
1216.626
y2
482.2565
y8 2+
535.2822
y10 2+
674.3366
y6 2+
420.2278
y7 2+
485.748
y3
b14 2+
y10
1347.666
Peptide Sequence
Protein Sequence
- V I I S A P S A D A P M F V M G V N H E
K -
y11 y10 y9 y8 y7 y6 y5 y4 y3 y2
b2 b5 b14 2

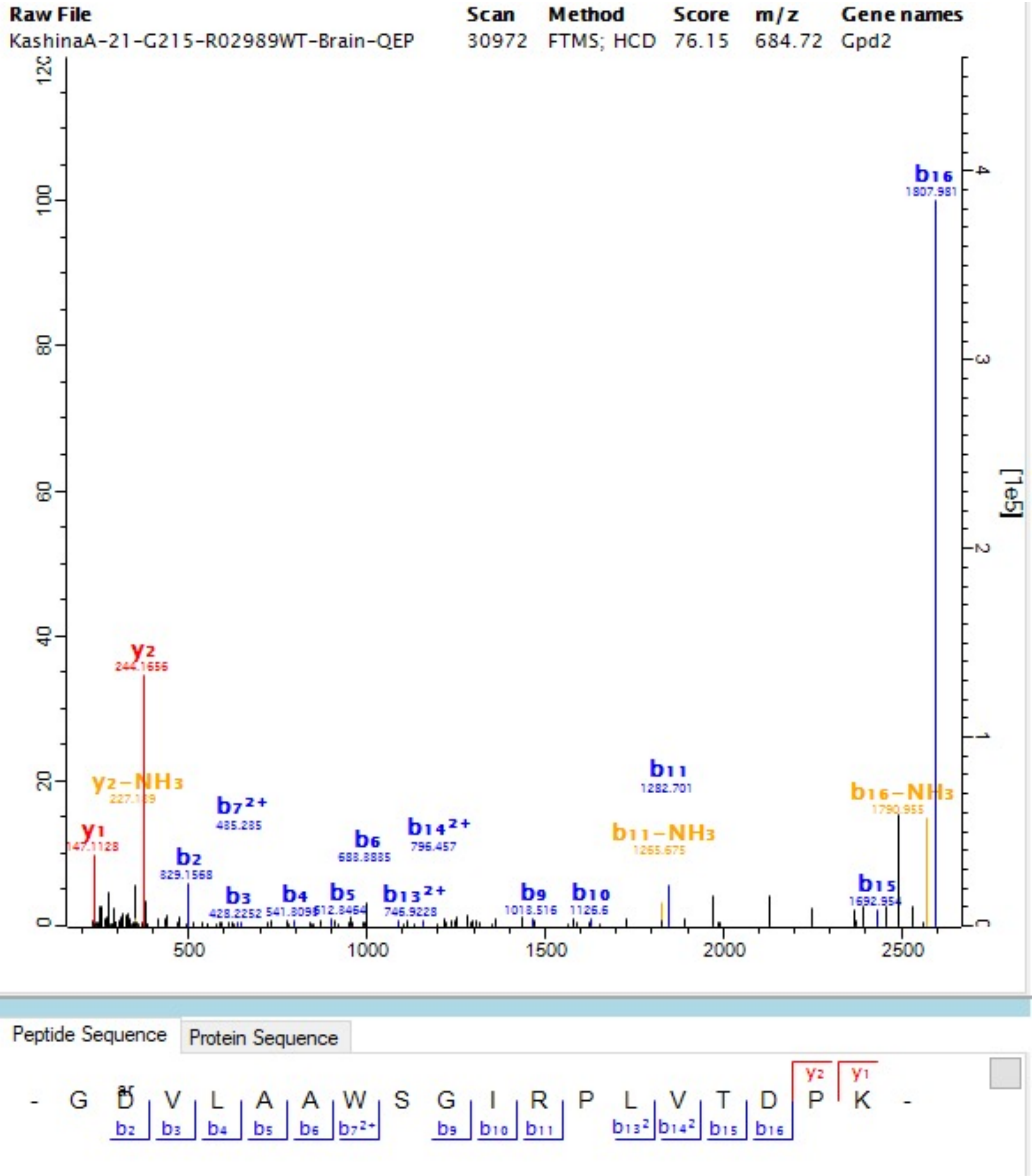

Raw File
KashinaA-21-G215-R02989WT-Brain-QEP
Scan
30972
Method
FTMS; HCD
Score
76.15
m/z
684.72
Gene names
Gpd2
y2
244.1656
y2-NH3
y1
147.1128
b2
b3
b4
b5
b6
b7 2+
485.285
b9
1018.516
b10
1126.6
b11
1282.701
b11-NH3
1265.675
b13 2+
746.9228
b14 2+
796.457
b15
1692.954
b16
1807.981
b16-NH3
1790.955
[1e5]
Peptide Sequence
Protein Sequence
- G D V L A A W S G I R P L V T D P K -

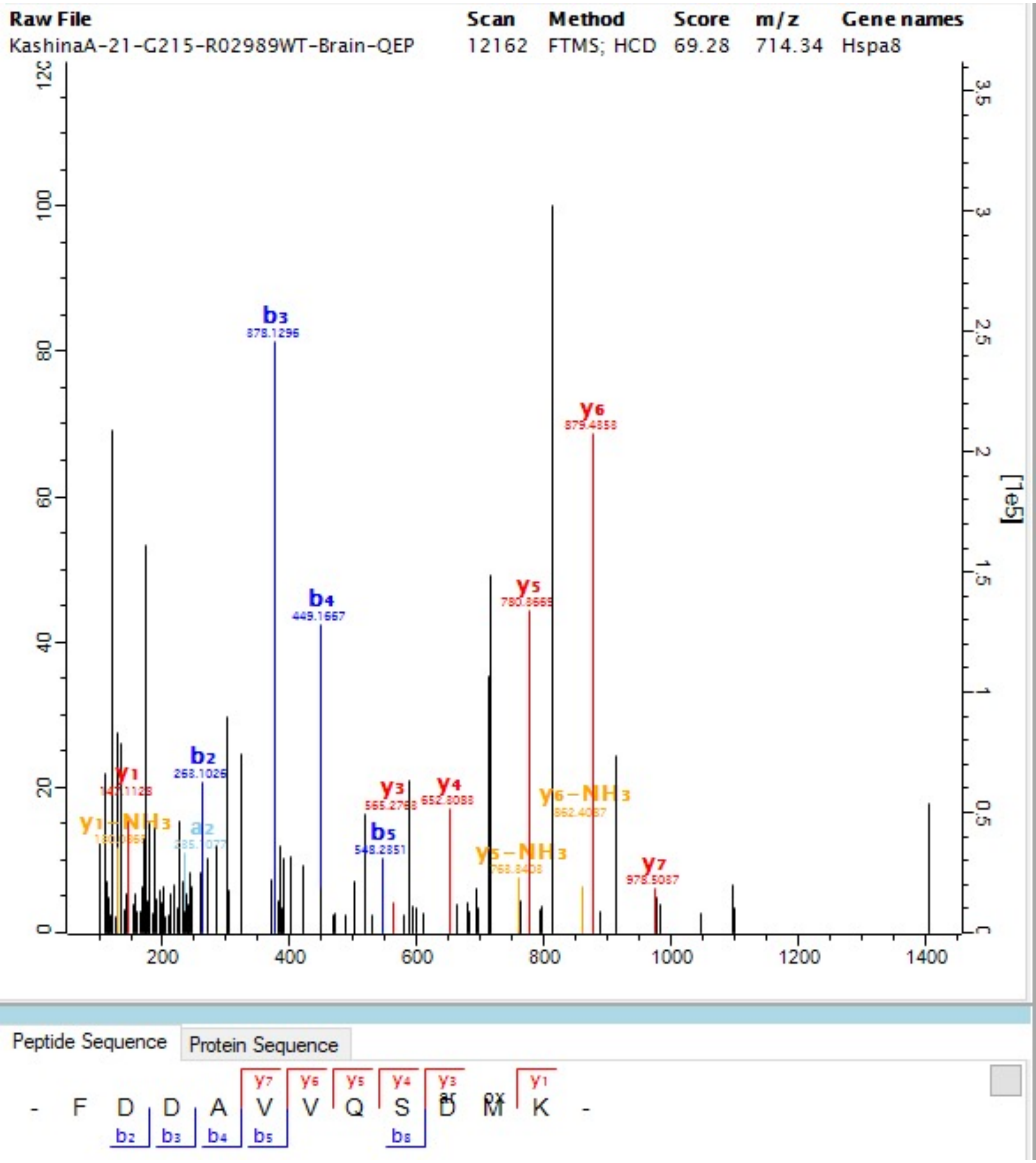

Raw File
KashinaA-21-G215-R02989WT-Brain-QEP
Scan
12162
Method
FTMS; HCD
Score
69.28
m/z
714.34
Gene names
Hspa8
[1e5]
b3
b4
b2
b5
a2
y1
y1-NH3
y3
y4
y5
y6
y7
y5-NH3
y6-NH3
Peptide Sequence
Protein Sequence
- F D D A V V Q S D M K -

Raw File	Scan	Method	Score	m/z	Gene names
KashinaA-21-G215-R02988WT-Brain-QEP	5556	FTMS; HCD	59.81	648.05	Hdgfrp2

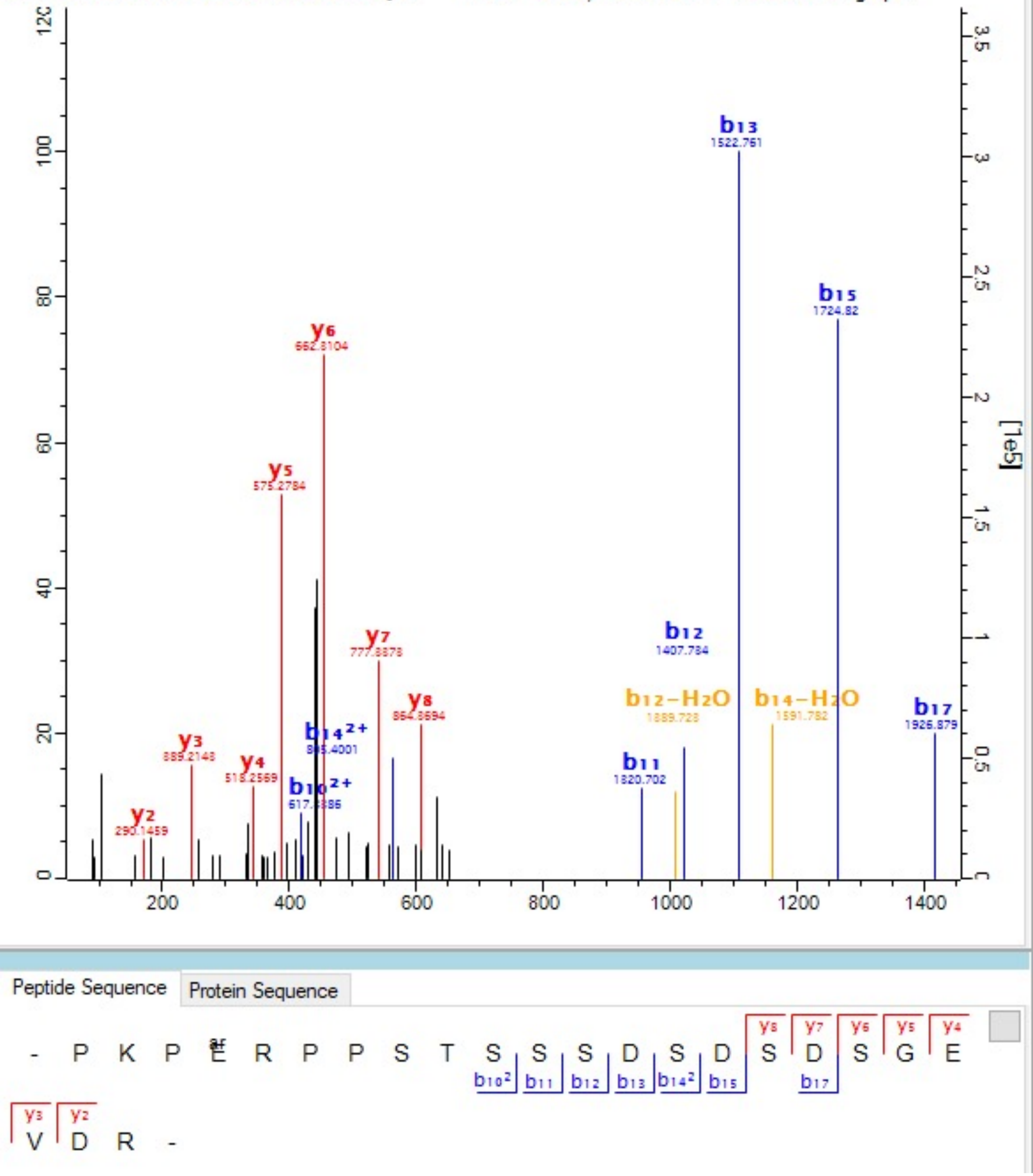

Raw File	Scan	Method	Score	m/z	Gene names
KashinaA-21-G215-R02990WT-Brain-QEP	5820	FTMS; HCD	52.48	648.05	Hdgfrp2

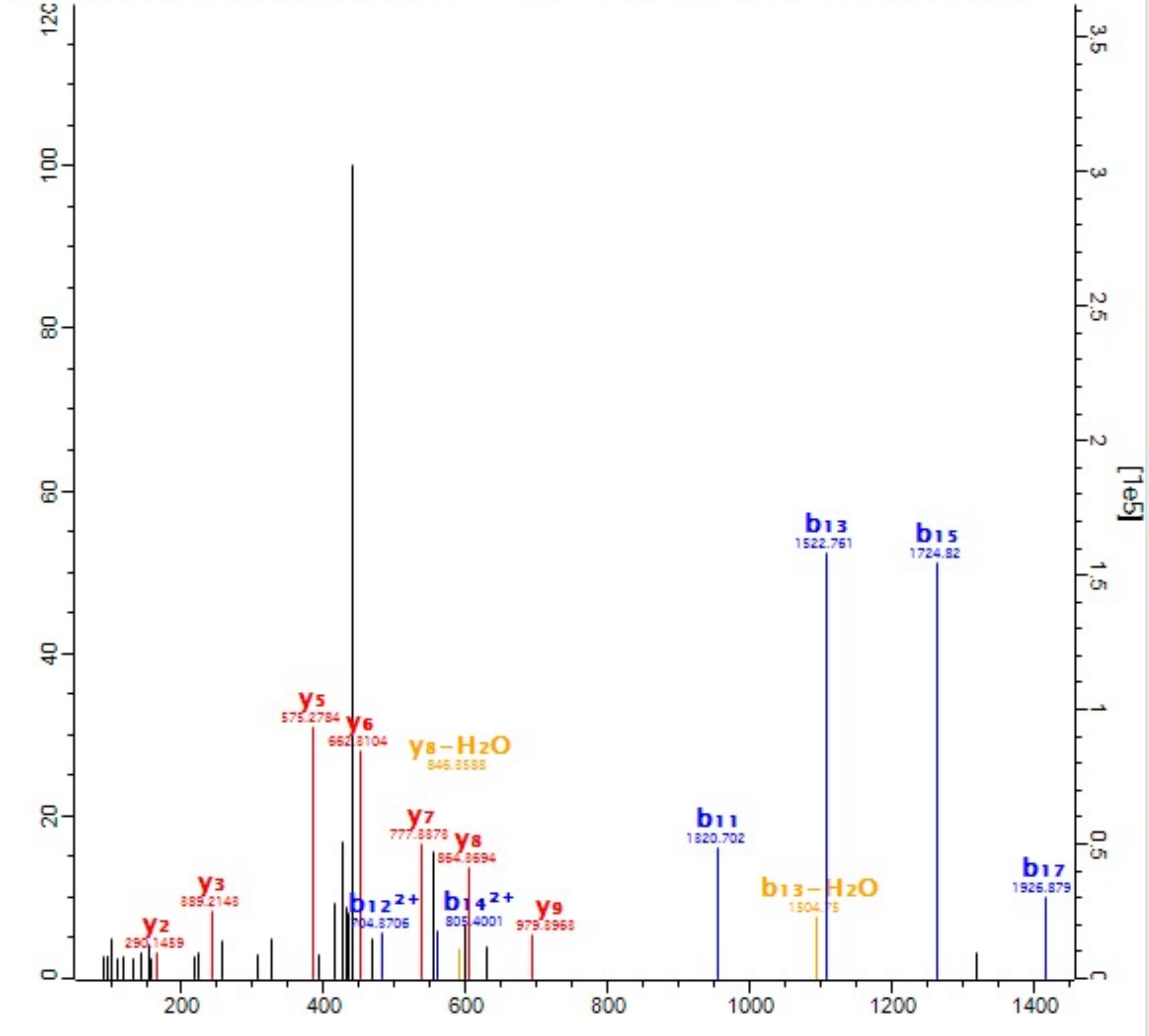

Peptide Sequence | Protein Sequence

- P K P E R P P S T S S S D S D S D S G E

V D R -

b11 b12² b13 b14² b15 b17

y9 y8 y7 y6 y5 y3 y2

Raw File	Scan	Method	Score	m/z	Gene names
KashinaA-21-G215-R02989WT-Brain-QEP	5816	FTMS; HCD	42.04	648.05	Hdgfrp2

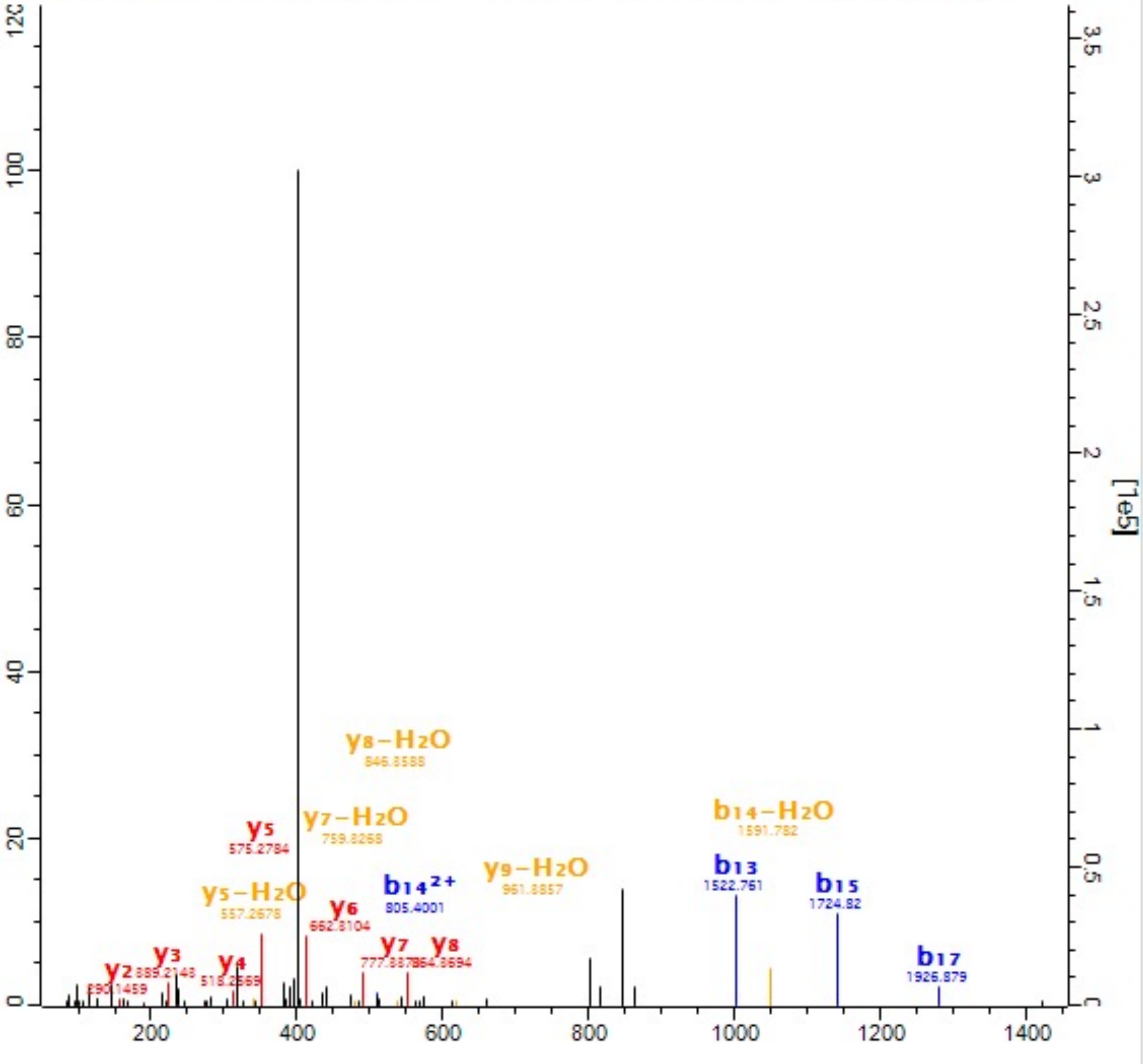

Peptide Sequence | Protein Sequence

- P K P E R P P S T S S S D S D S D S G E V D R -

b13 b14² b15 b17

y8 y7 y6 y5 y4 y3 y2

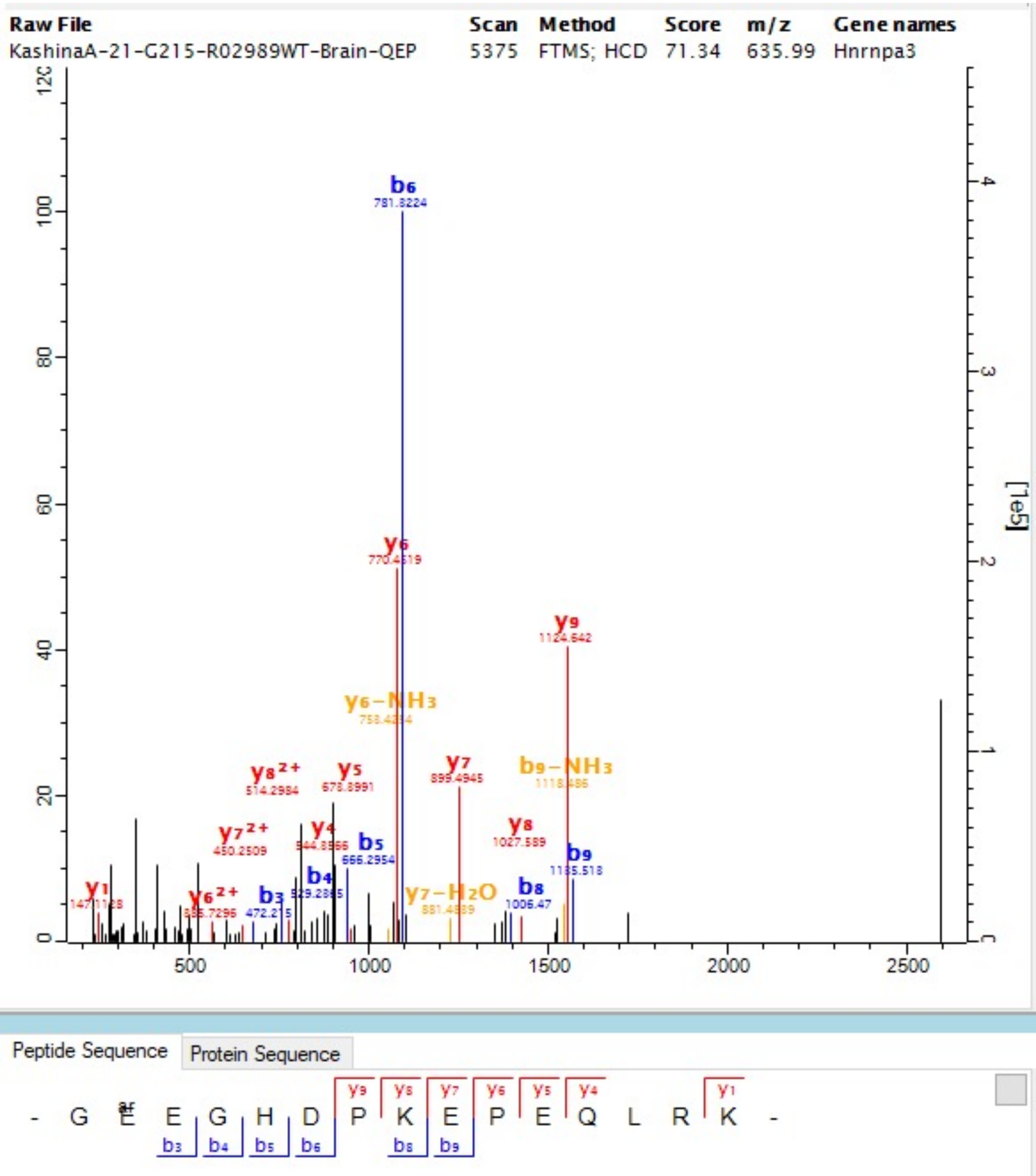

Raw File
KashinaA-21-G215-R02989WT-Brain-QEP
Scan
5375
Method
FTMS; HCD
Score
71.34
m/z
635.99
Gene names
Hnrnpa3
b6
781.3224
y6
y6-NH3
y9
1124.642
y8 2+
514.2984
y5
678.3991
y7
899.4945
b9-NH3
1118.486
y7 2+
450.2509
y4
b5
666.2954
y8
1027.589
b9
y1
y6 2+
b3
472.215
b4
y7-H2O
881.4889
b8
1006.47
[1e5]
500
1000
1500
2000
2500
Peptide Sequence
Protein Sequence
- G E E G H D P K E P E Q L R K -
y9 y8 y7 y6 y5 y4 y1
b3 b4 b5 b6 b8 b9

Raw File	Scan	Method	Score	m/z	Gene names
KashinaA-21-G215-R02989WT-Brain-QEP	5384	FTMS; HCD	84.76	477	Hnrnpa3

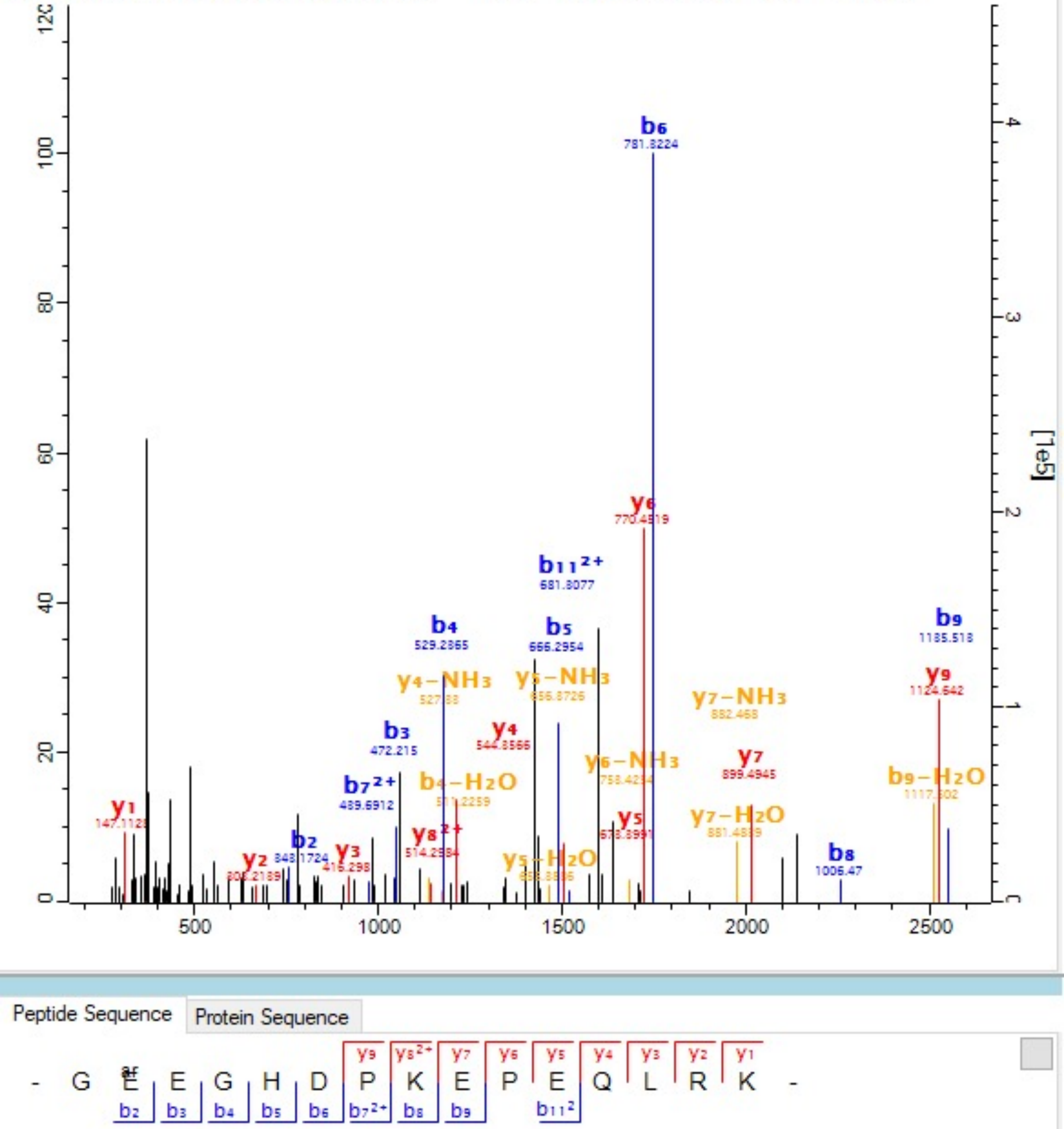

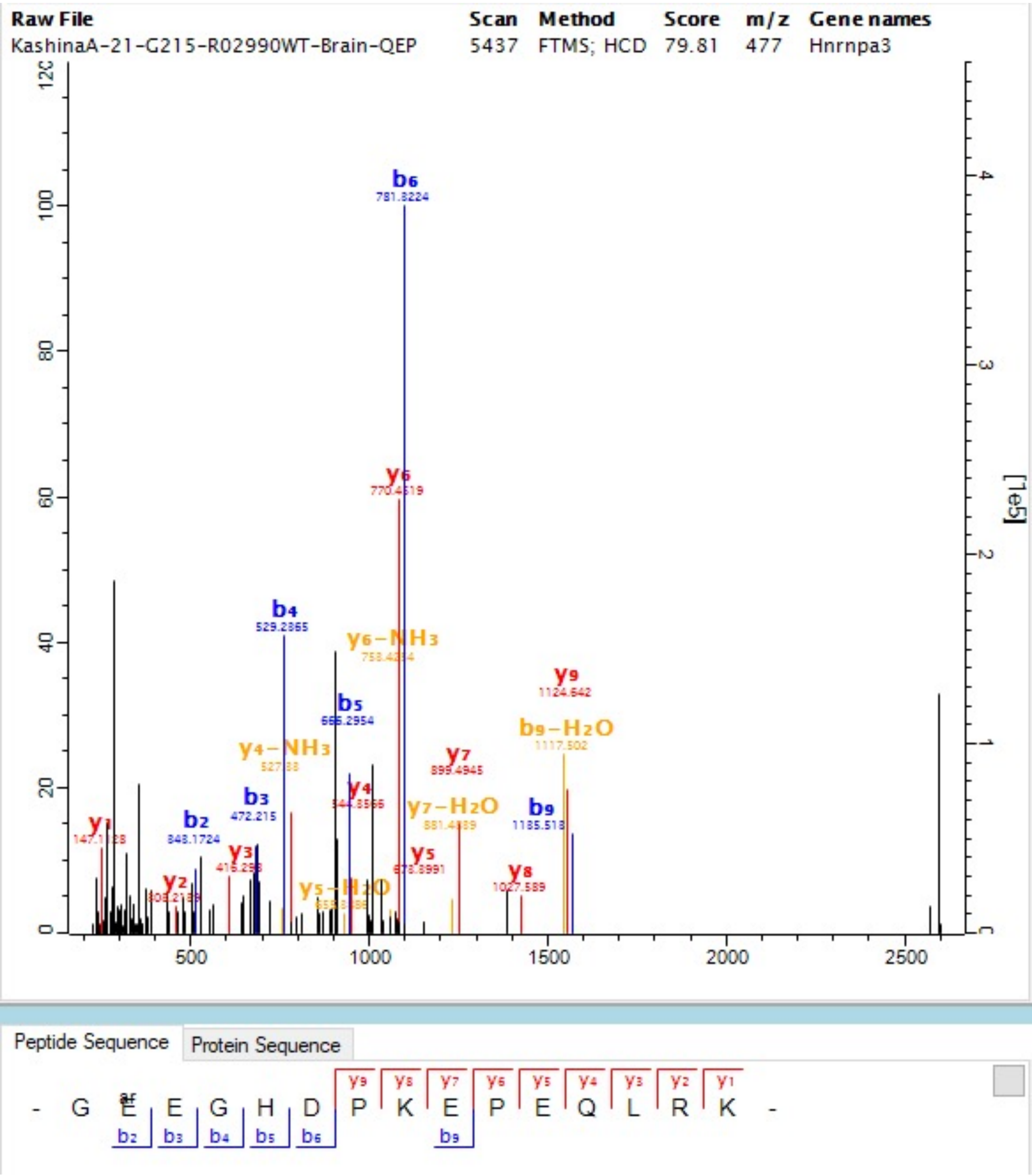

Raw File
KashinaA-21-G215-R02990WT-Brain-QEP
Scan
5437
Method
FTMS; HCD
Score
79.81
m/z
477
Gene names
Hnrnpa3
b6
781.8224
y6
770.4319
b4
529.2865
y6-NH3
753.4254
y9
1124.642
b5
666.2954
b9-H2O
1117.502
y4-NH3
527.38
y7
899.4945
b3
472.215
y4
544.3566
y7-H2O
881.4839
b9
1135.518
b2
343.1724
y1
147.1128
y3
416.298
y5
673.3991
y2
303.2189
y8
1027.589
y5-H2O
[1e5]
Peptide Sequence
Protein Sequence
y9 y8 y7 y6 y5 y4 y3 y2 y1
- G E E G H D P K E P E Q L R K -
af
b2 b3 b4 b5 b6 b9

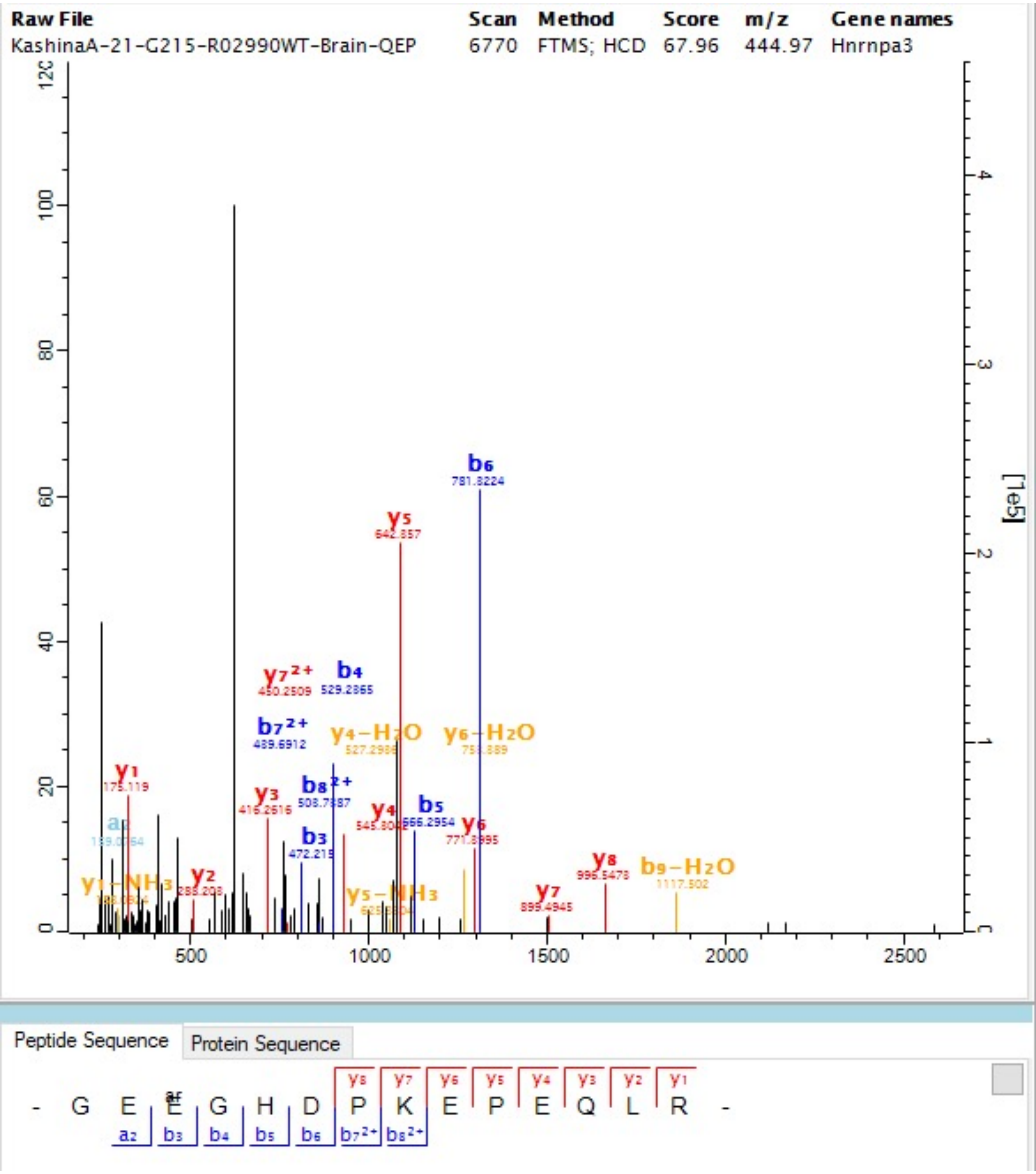
Raw File
KashinaA-21-G215-R02990WT-Brain-QEP
Scan
6770
Method
FTMS; HCD
Score
67.96
m/z
444.97
Gene names
Hnrnpa3
b6
781.8224
y5
642.857
y7 2+
450.2509
b4
529.2865
b7 2+
489.6912
y4-H2O
527.2986
y6-H2O
y1
175.119
y3
416.2616
b8 2+
508.7887
y4
b5
666.2954
y6
b3
472.215
y8
995.5478
b9-H2O
1117.502
y2
y7
899.4945
[1e5]
Peptide Sequence
Protein Sequence
- G E E G H D P K E P E Q L R -
y8 y7 y6 y5 y4 y3 y2 y1
a2 b3 b4 b5 b6 b7 2+ b8 2+

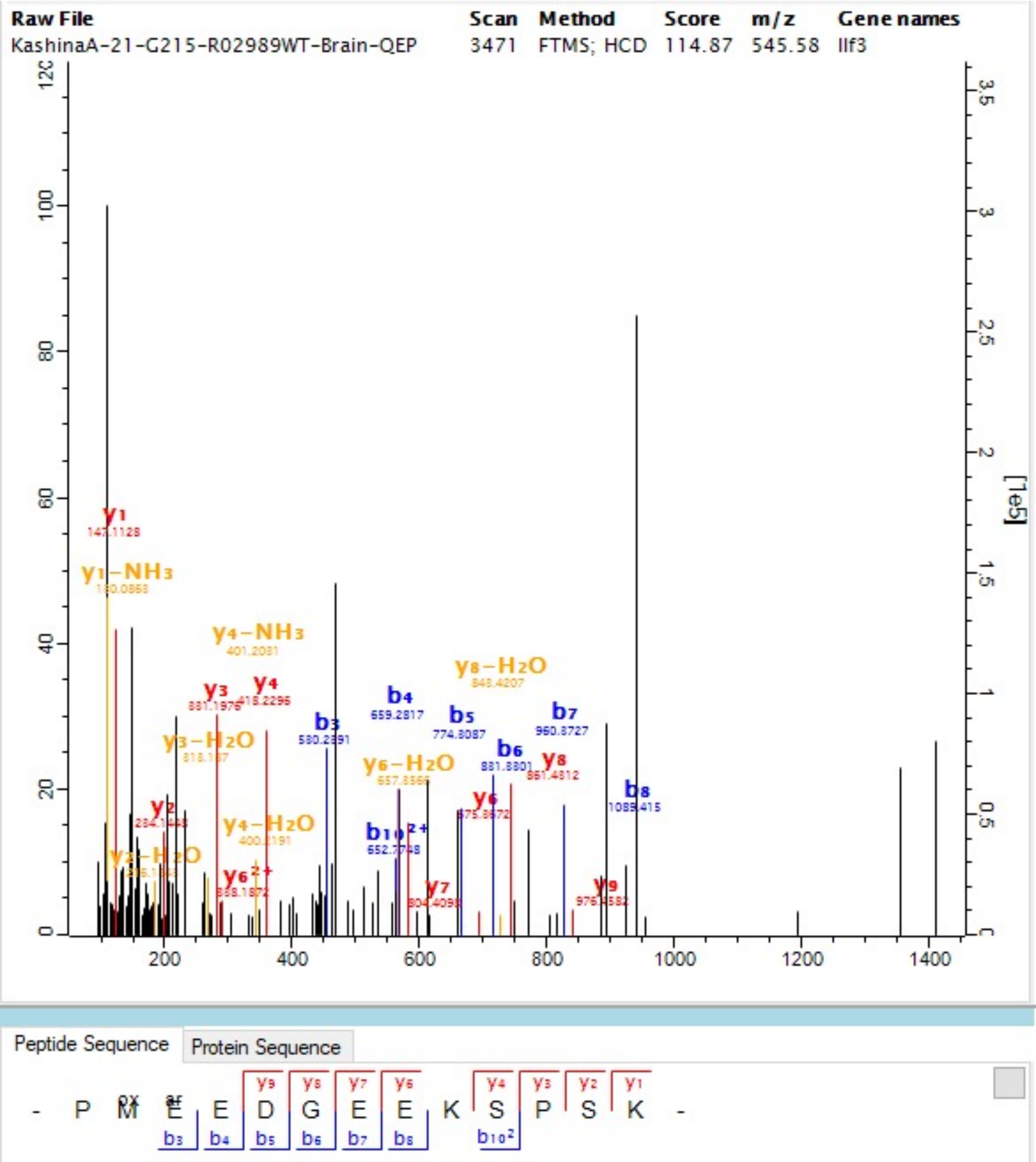
Raw File
KashinaA-21-G215-R02989WT-Brain-QEP
Scan
3471
Method
FTMS; HCD
Score
114.87
m/z
545.58
Gene names
Ilf3
y1
y1−NH3
y4−NH3
401.2081
y8−H2O
848.4207
y3
y4
418.2296
b4
659.2817
b5
774.3087
b7
960.3727
b3
580.2391
y3−H2O
b6
881.3801
y8
861.4812
y6−H2O
657.3566
y2
284.1448
y6
b8
1089.415
y4−H2O
400.2191
b10 2+
652.7748
y2−H2O
y6 2+
y7
y9
[1e5]
Peptide Sequence
Protein Sequence
- P M E E D G E E K S P S K -
y9 y8 y7 y6 y4 y3 y2 y1
b3 b4 b5 b6 b7 b8 b10 2

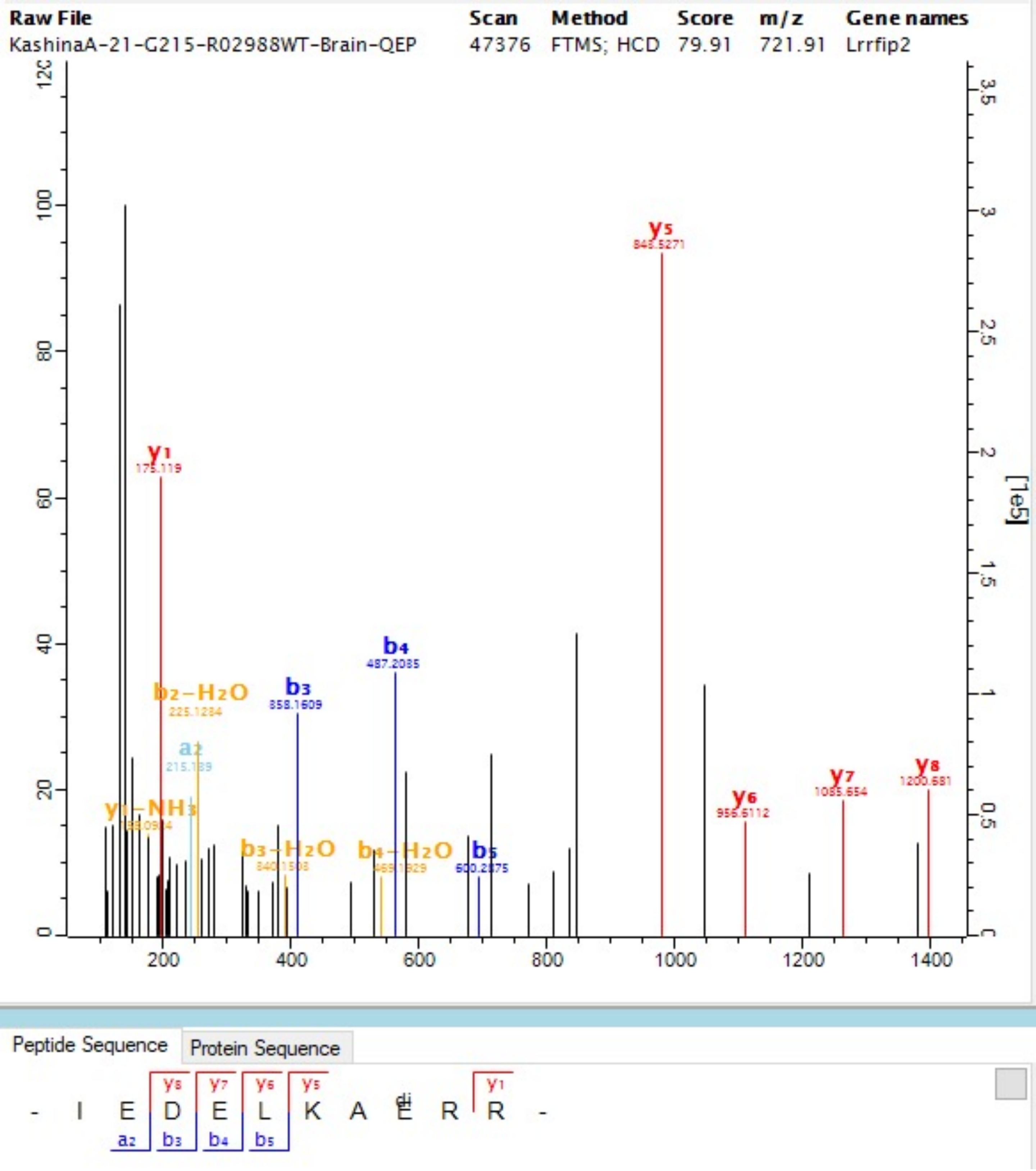

Raw File
KashinaA-21-G215-R02988WT-Brain-QEP
Scan
47376
Method
FTMS; HCD
Score
79.91
m/z
721.91
Gene names
Lrrfip2
y1
175.119
y5
b4
487.2085
b3
b2-H2O
225.1284
a2
y1-NH3
b3-H2O
b4-H2O
b5
y6
y7
y8
[1e5]
Peptide Sequence
Protein Sequence
- I E D E L K A E R R -

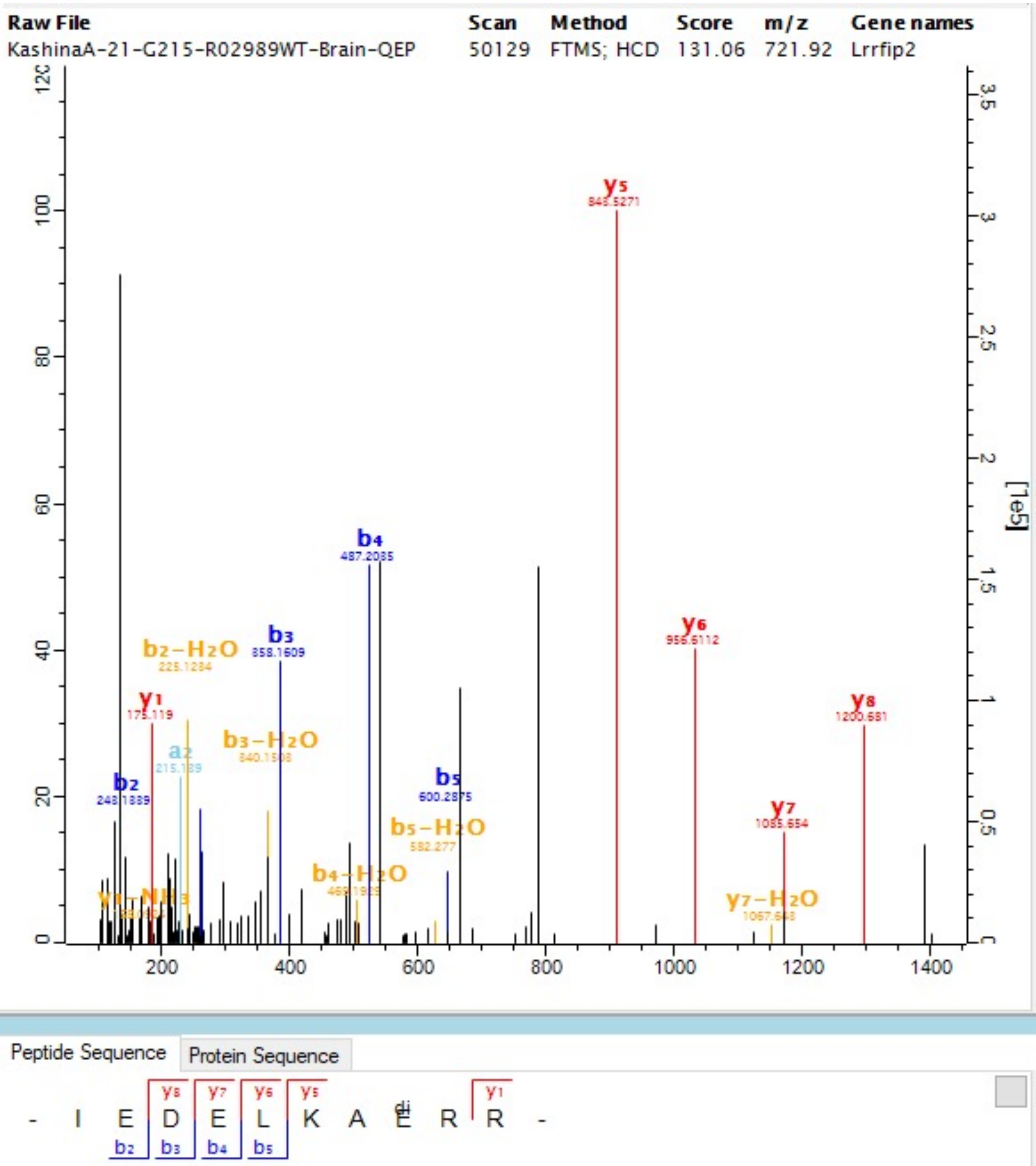

Raw File
KashinaA-21-G215-R02989WT-Brain-QEP
Scan
50129
Method
FTMS; HCD
Score
131.06
m/z
721.92
Gene names
Lrrfip2
[1e5]
y5
y6
y7
y8
y1
b2
b3
b4
b5
a2
b2-H2O
b3-H2O
b4-H2O
b5-H2O
y7-H2O
Peptide Sequence
Protein Sequence
- I E D E L K A E R R -

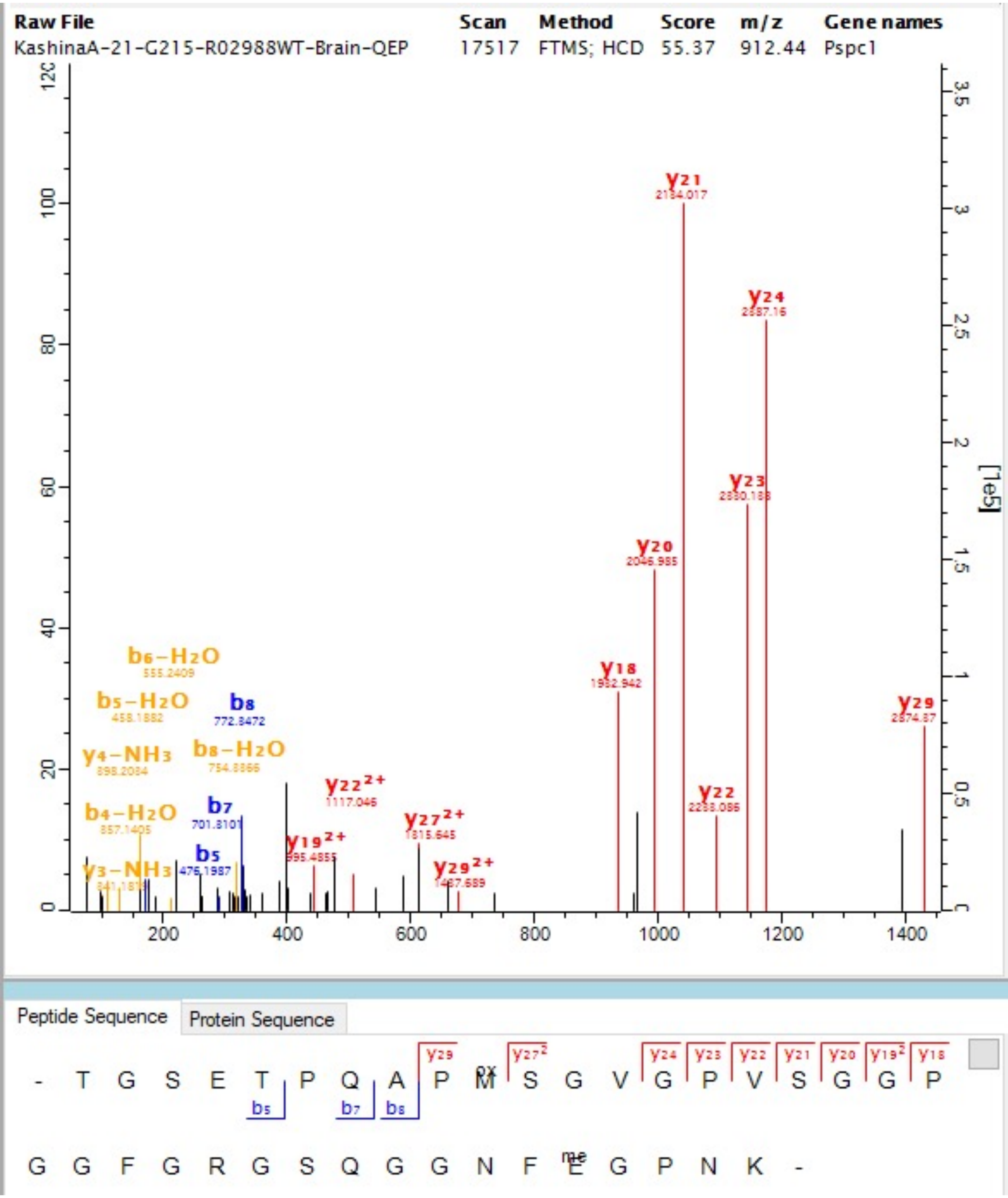

Raw File
KashinaA-21-G215-R02988WT-Brain-QEP
Scan
17517
Method
FTMS; HCD
Score
55.37
m/z
912.44
Gene names
Pspc1
[1e5]
y21
y24
y23
y20
y18
y29
y22
y22 2+
y27 2+
y19 2+
y29 2+
b6-H2O
555.2409
b5-H2O
b8
772.3472
y4-NH3
b8-H2O
b4-H2O
b7
701.8101
b5
476.1987
y3-NH3
Peptide Sequence
Protein Sequence
- T G S E T P Q A P M S G V G P V S G G P
ox
G G F G R G S Q G G N F E G P N K -
me

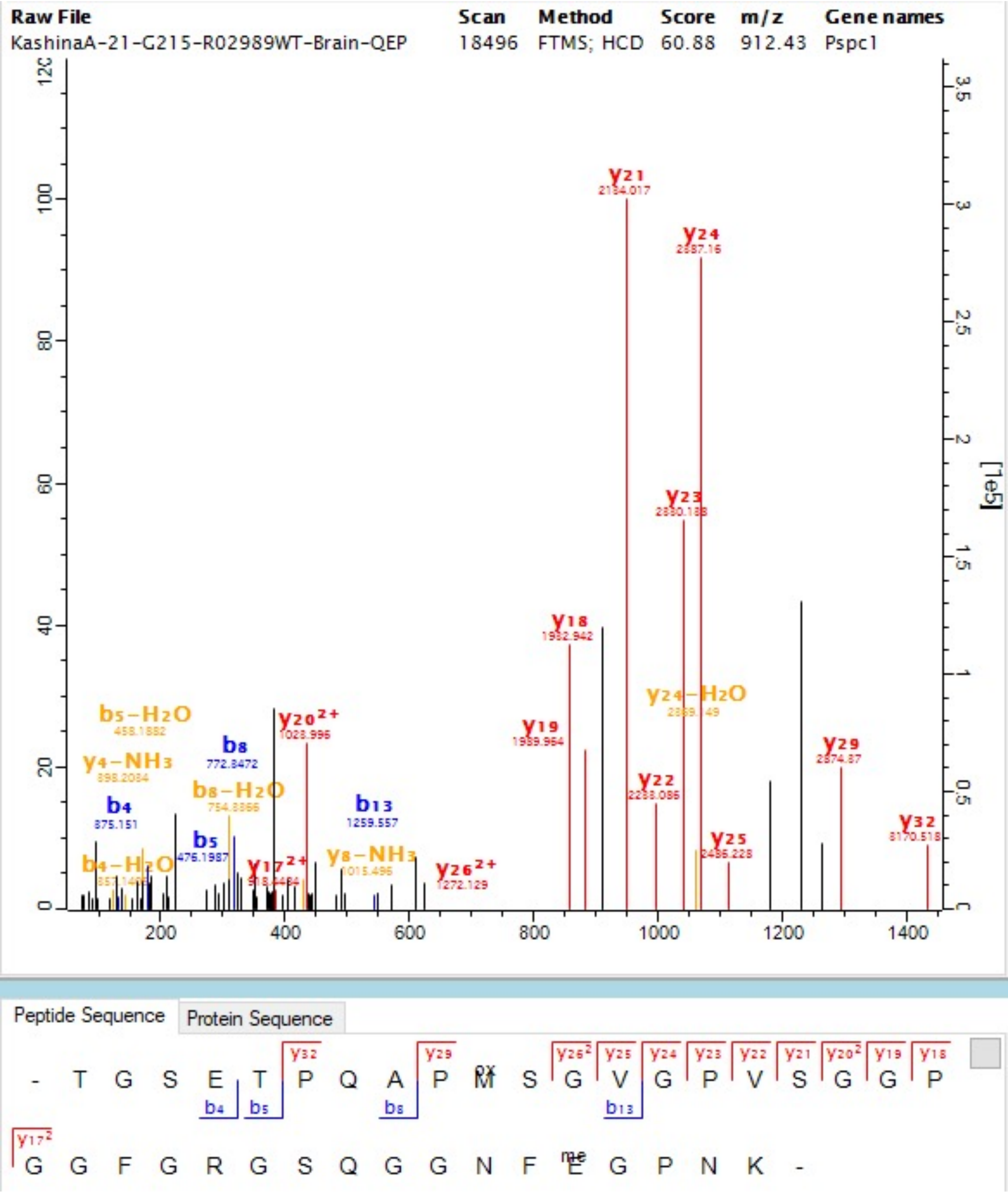
Raw File
KashinaA-21-G215-R02989WT-Brain-QEP
Scan
18496
Method
FTMS; HCD
Score
60.88
m/z
912.43
Gene names
Pspc1
y21
y24
y23
y18
y19
y22
y25
y29
y32
y20 2+
y17 2+
y26 2+
y24−H2O
y4−NH3
y8−NH3
b4
b5
b8
b13
b4−H2O
b5−H2O
b8−H2O
[1e5]
Peptide Sequence
Protein Sequence
- T G S E T P Q A P M S G V G P V S G G P
G G F G R G S Q G G N F E G P N K -

Raw File	Scan	Method	Score	m/z	Gene names
KashinaA-21-G215-R02990WT-Brain-QEP	18365	FTMS; HCD	82.55	912.69	Pspc1

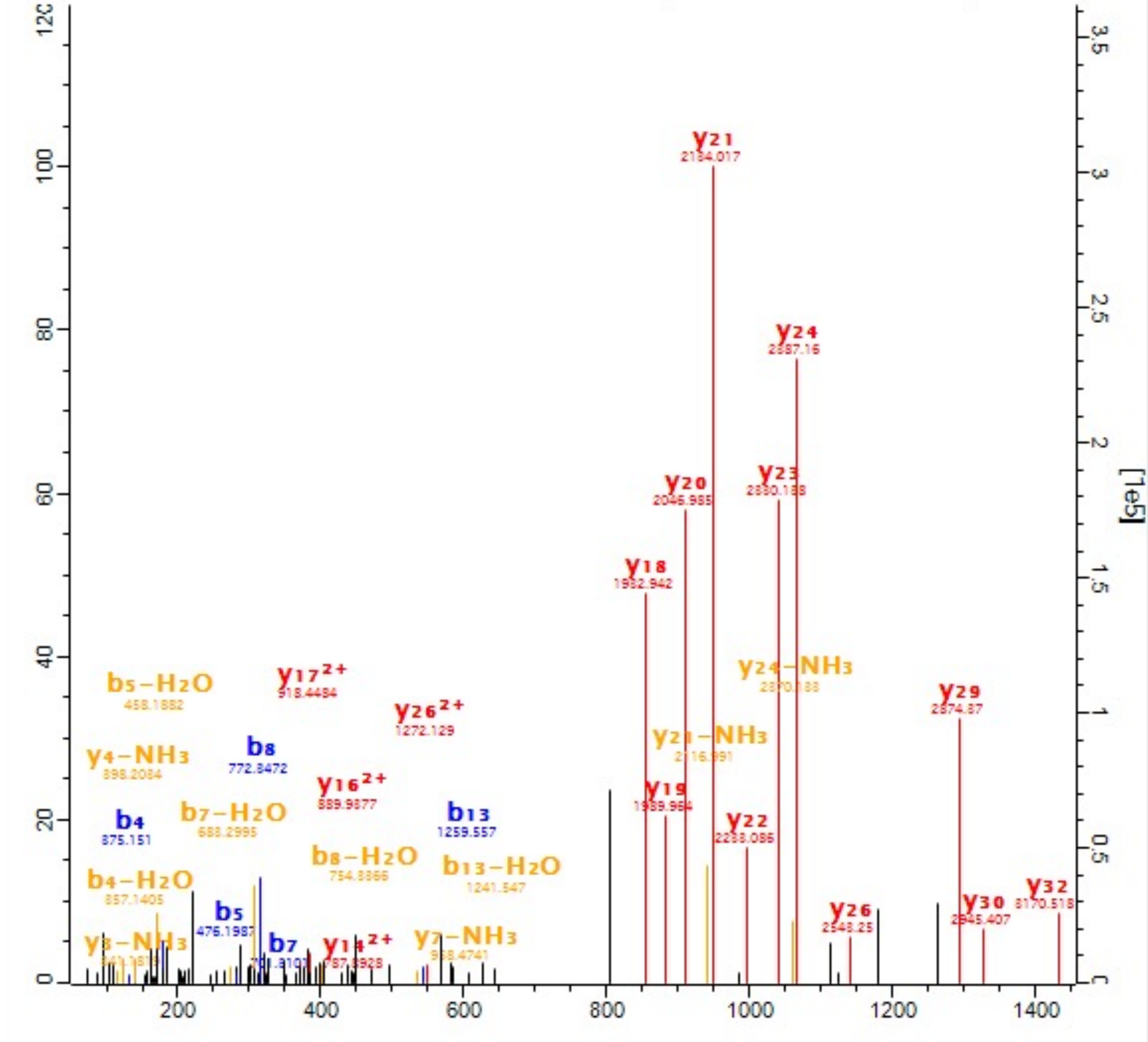

Peptide Sequence | Protein Sequence

- T G S E T P Q A P M(ox) S G V G P V S G G P

G G F G R G S Q G G N F E(me) G P N K -

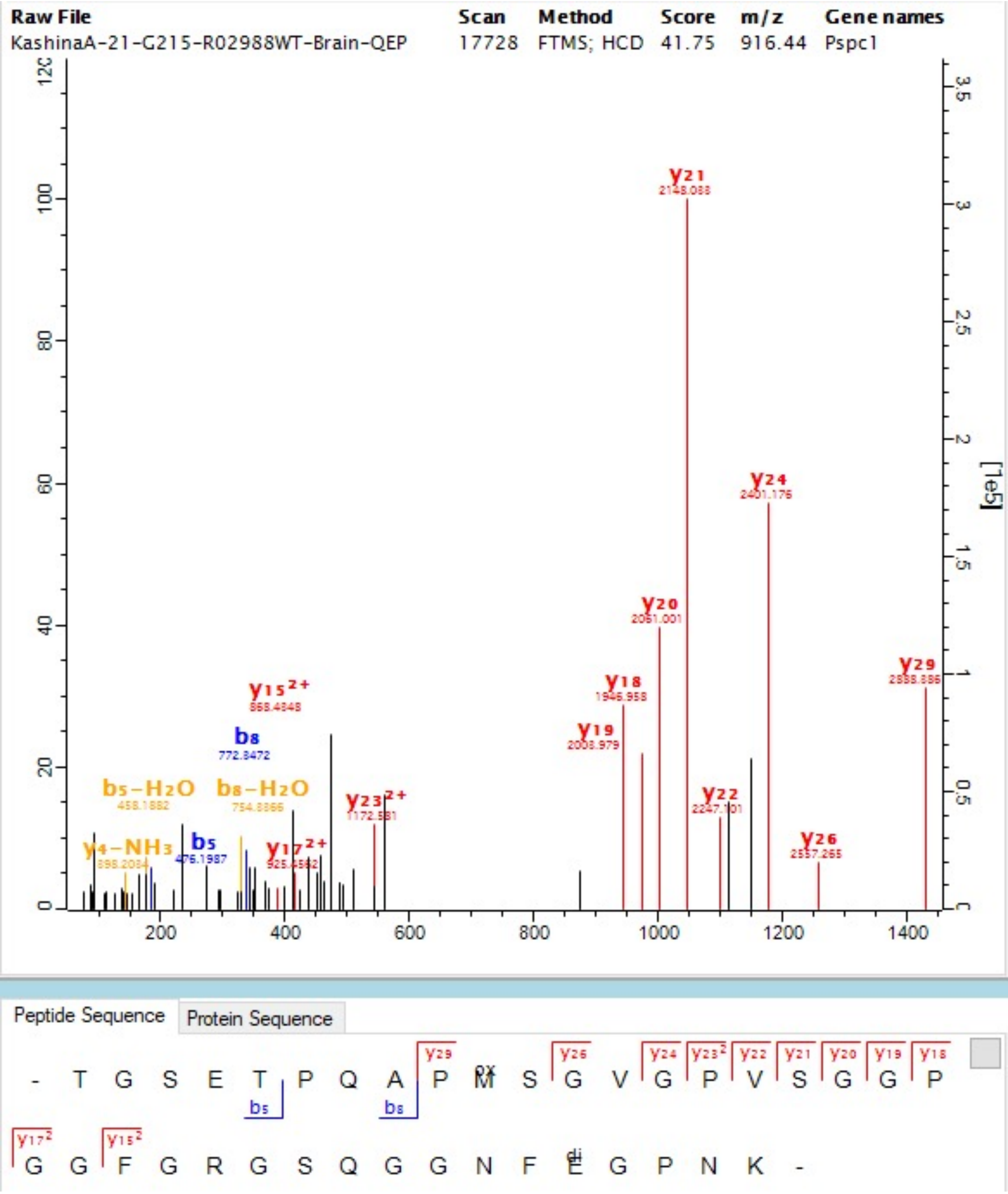
Raw File
KashinaA-21-G215-R02988WT-Brain-QEP
Scan
17728
Method
FTMS; HCD
Score
41.75
m/z
916.44
Gene names
Pspc1
y21
2148.088
y24
2401.176
y20
2061.001
y29
2888.886
y18
1946.958
y19
2003.979
y22
2247.101
y26
2557.265
y15 2+
868.4846
b8
772.8472
b5-H2O
458.1882
b8-H2O
754.8866
y23 2+
1172.581
y4-NH3
b5
476.1987
y17 2+
925.4562
[1e5]
Peptide Sequence
Protein Sequence
- T G S E T P Q A P M S G V G P V S G G P
ox
G G F G R G S Q G G N F E G P N K -
di

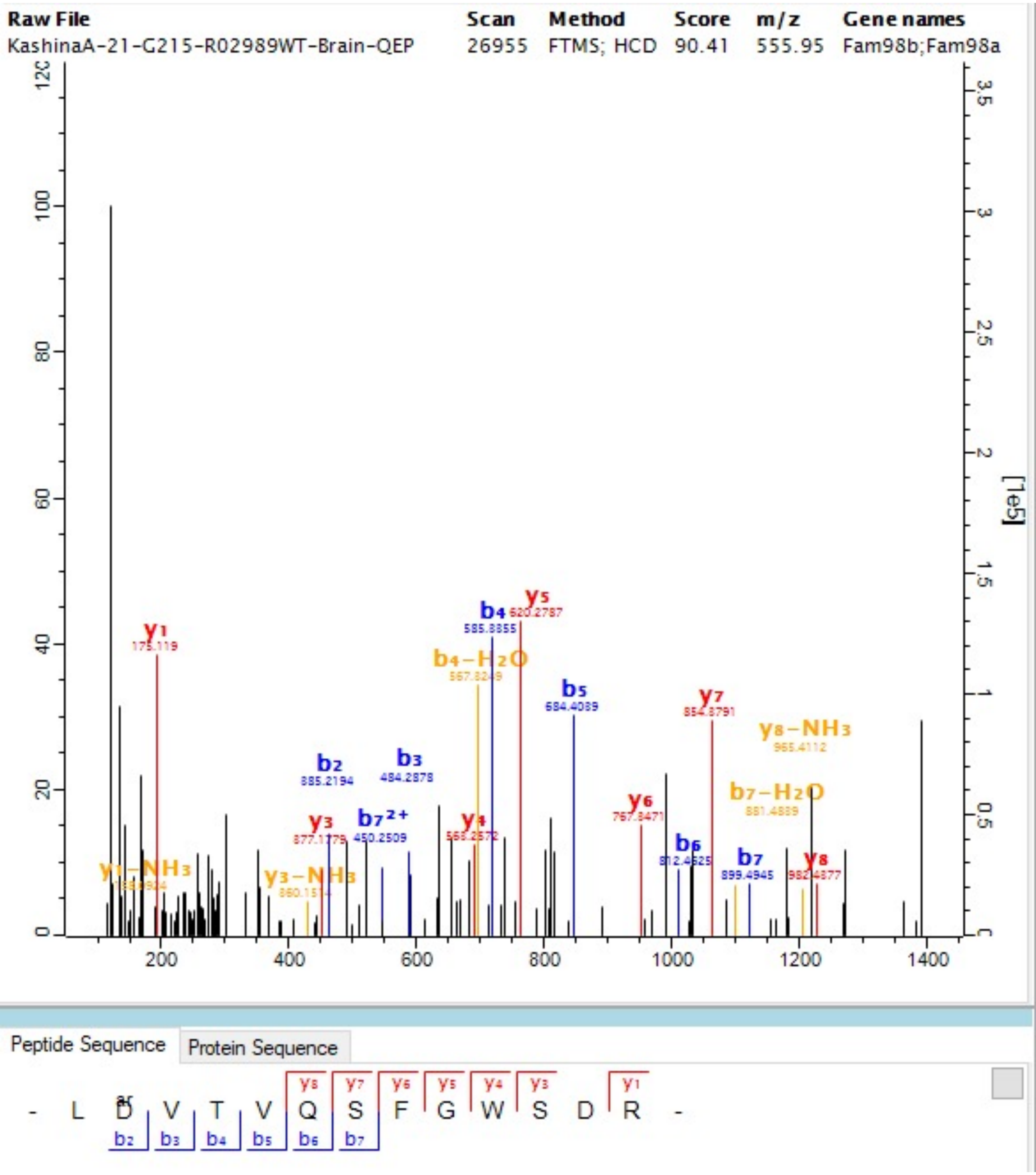
Raw File
KashinaA-21-G215-R02989WT-Brain-QEP
Scan
26955
Method
FTMS; HCD
Score
90.41
m/z
555.95
Gene names
Fam98b;Fam98a
[1e5]
y1
b4
585.8855
y5
620.2787
b4-H2O
567.8249
b5
684.4089
y7
854.8791
y8-NH3
965.4112
b2
385.2194
b3
484.2878
b7-H2O
881.4889
y3
b7 2+
450.2509
y4
y6
b6
b7
899.4945
y8
y1-NH3
y3-NH3
Peptide Sequence
Protein Sequence
- L D V T V Q S F G W S D R -

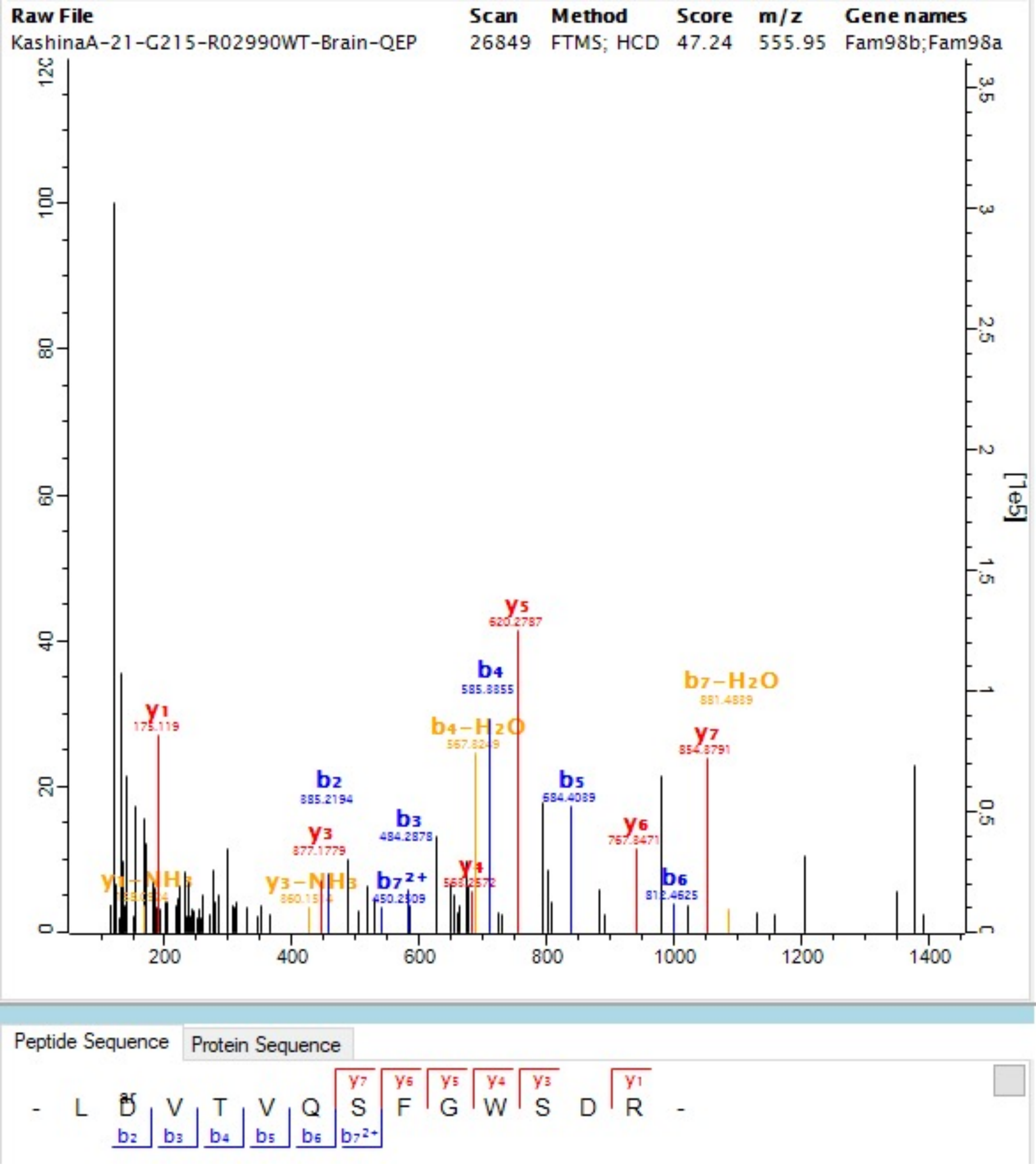
Raw File
KashinaA-21-G215-R02990WT-Brain-QEP
Scan
26849
Method
FTMS; HCD
Score
47.24
m/z
555.95
Gene names
Fam98b;Fam98a
y1
175.119
b2
385.2194
y3
377.1779
b3
484.2878
b7 2+
450.2309
b4
585.3355
b4-H2O
567.3249
y5
620.2787
b5
684.4039
y6
767.3471
b6
812.4625
y7
854.3791
b7-H2O
881.4839
[1e5]
Peptide Sequence
Protein Sequence
- L D V T V Q S F G W S D R -

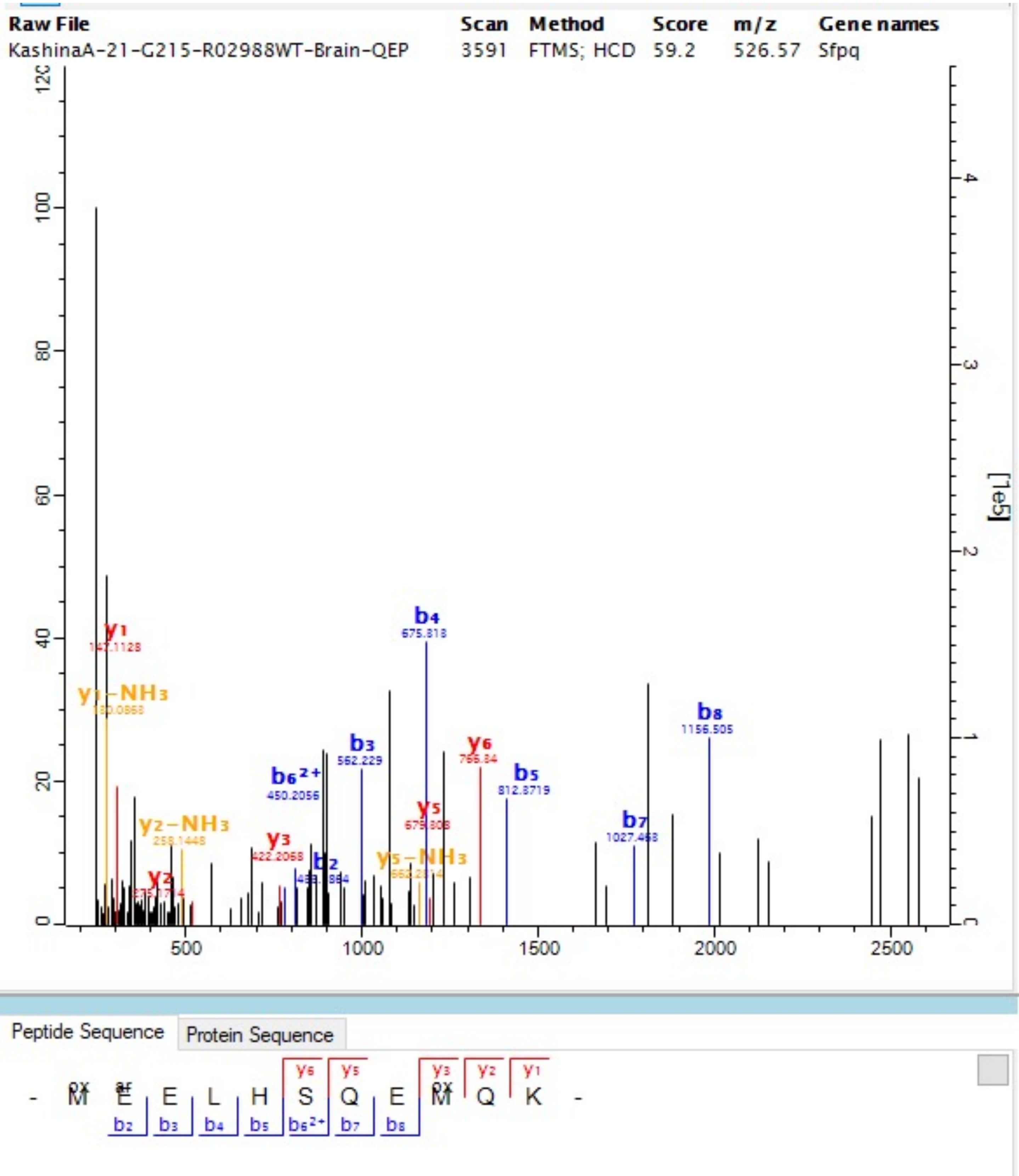

Raw File
KashinaA-21-G215-R02988WT-Brain-QEP
Scan
3591
Method
FTMS; HCD
Score
59.2
m/z
526.57
Gene names
Sfpq
y1
147.1128
y1-NH3
y2-NH3
y2
y3
422.2068
b2
b6 2+
450.2056
b3
562.229
b4
675.818
y5-NH3
y5
y6
b5
812.8719
b7
1027.468
b8
1156.505
[1e5]
Peptide Sequence
Protein Sequence
- M E E L H S Q E M Q K -
ox
ac
ox
y6 y5 y3 y2 y1
b2 b3 b4 b5 b6 2+ b7 b8

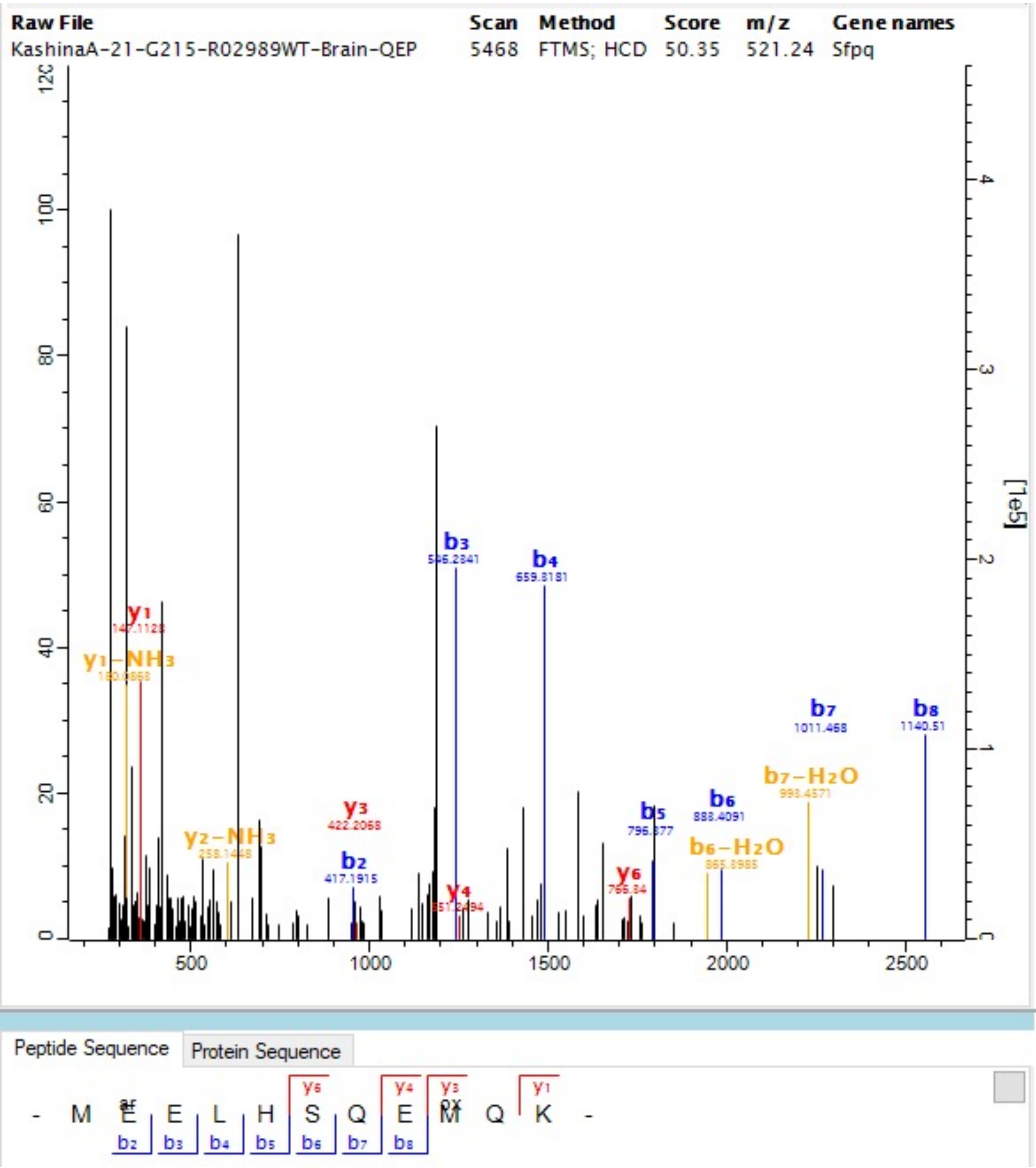
Raw File
KashinaA-21-G215-R02989WT-Brain-QEP
Scan
5468
Method
FTMS; HCD
Score
50.35
m/z
521.24
Gene names
Sfpq
[1e5]
y1
y1-NH3
y2-NH3
y3
422.2068
b2
417.1915
b3
b4
659.8181
y4
y6
b5
b6
888.4091
b6-H2O
b7-H2O
b7
1011.468
b8
1140.51
Peptide Sequence
Protein Sequence
- M E E L H S Q E M Q K -
y6 y4 y3 y1
b2 b3 b4 b5 b6 b7 b8

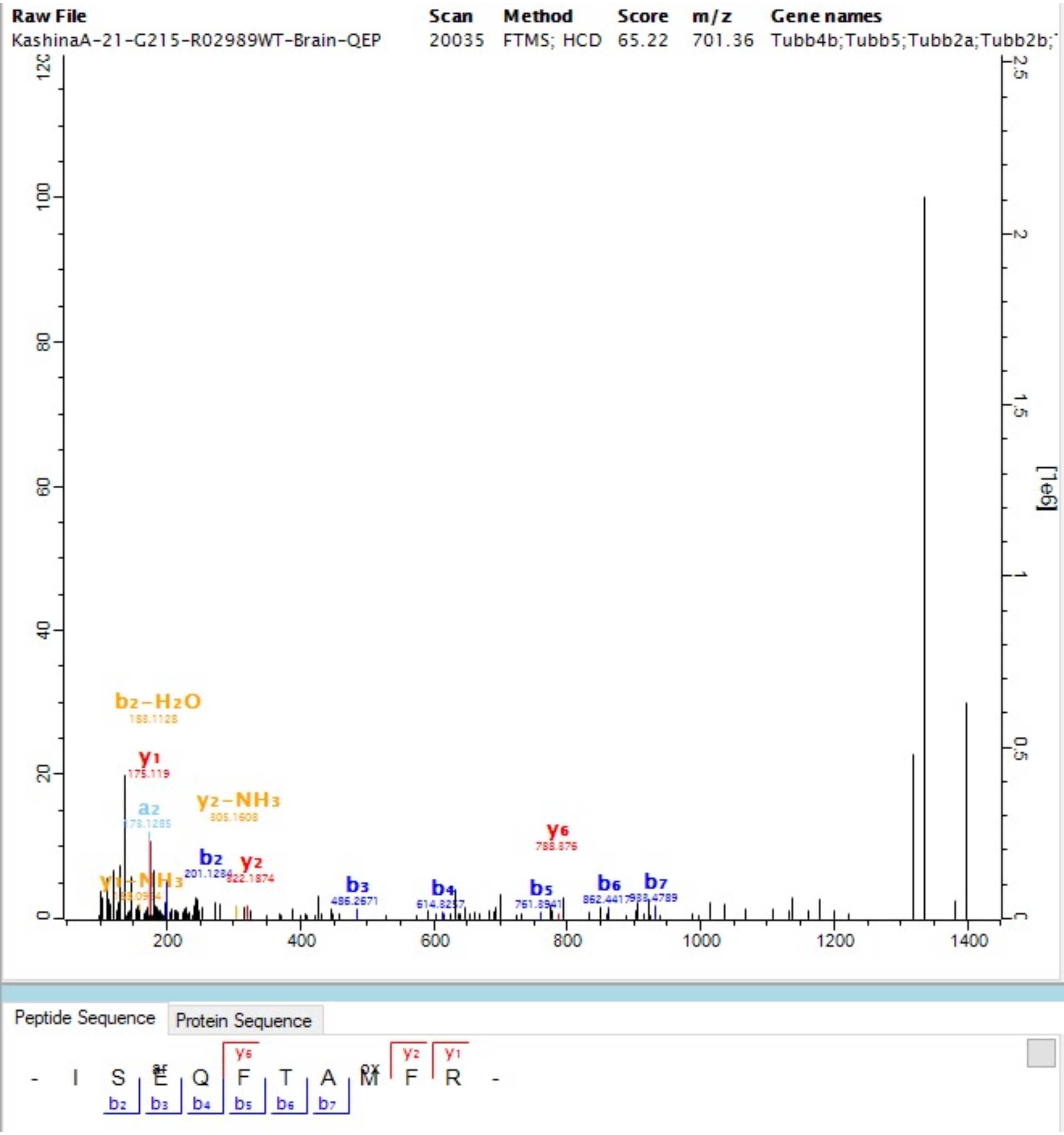
Raw File
KashinaA-21-G215-R02989WT-Brain-QEP
Scan
20035
Method
FTMS; HCD
Score
65.22
m/z
701.36
Gene names
Tubb4b;Tubb5;Tubb2a;Tubb2b;
0
20
40
60
80
100
200
400
600
800
1000
1200
1400
0.5
1
1.5
2
2.5
[1e6]
b2-H2O
y1
175.119
a2
y2-NH3
b2
201.1234
y2
b3
b4
b5
b6
b7
y6
Peptide Sequence
Protein Sequence
- I S E Q F T A M F R -
y6
y2
y1
b2
b3
b4
b5
b6
b7
