# Supplementary material for: Global Analysis of Post-Translational Side-Chain Arginylation Using Pan-Arginylation Antibodies
Source: Mol Cell Proteomics. 2023 Oct 12;22(11):100664. doi: 10.1016/j.mcpro.2023.100664 (PMC10656225; doi:10.1016/j.mcpro.2023.100664)
Supplement: Supplemental File 4 [file mmc4.pdf]

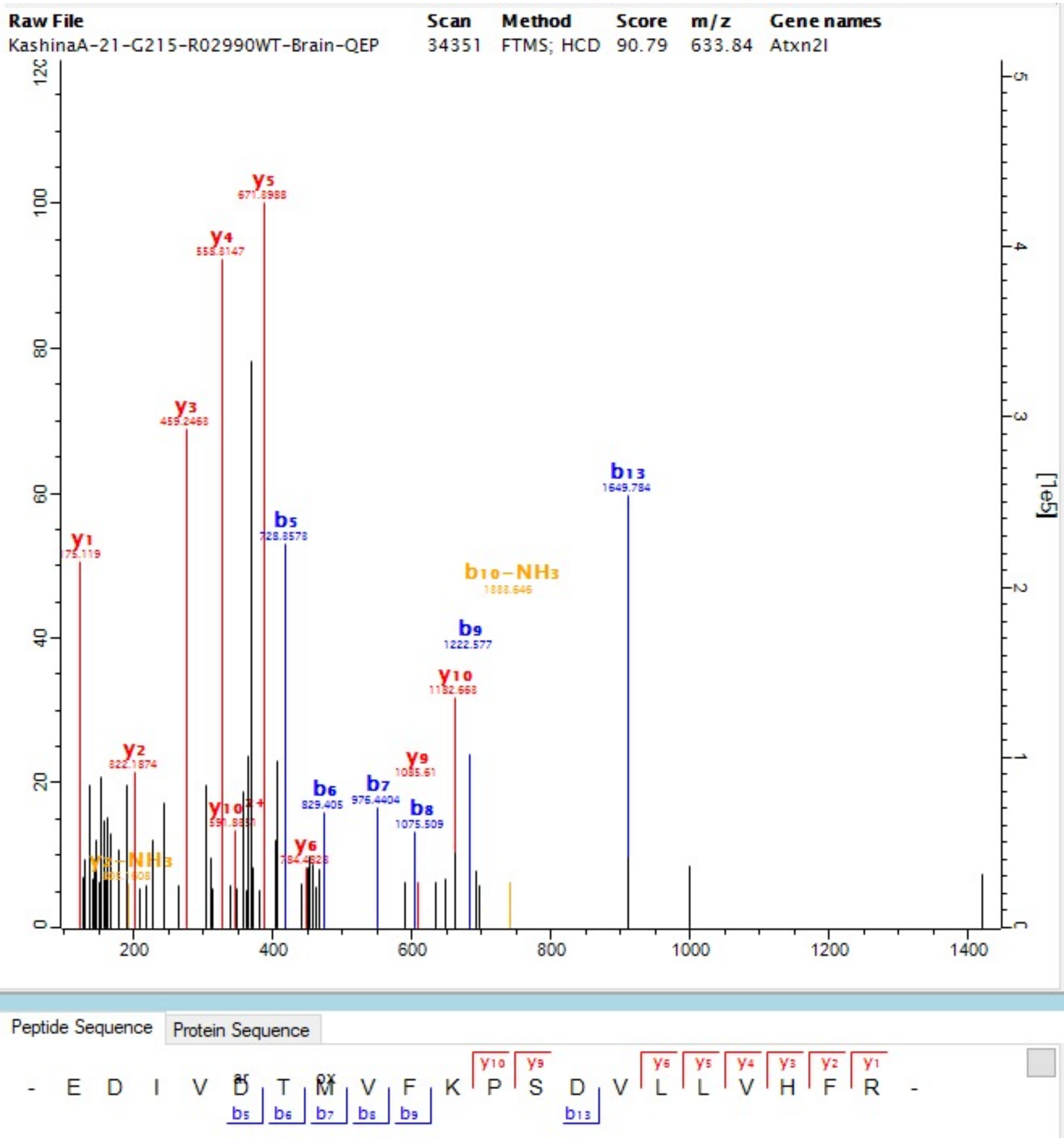
Raw File
KashinaA-21-G215-R02990WT-Brain-QEP
Scan
34351
Method
FTMS; HCD
Score
90.79
m/z
633.84
Gene names
Atxn2l
y5
671.8988
y4
558.8147
y3
459.2468
b13
1649.784
b5
728.8578
y1
175.119
b10-NH3
b9
1222.577
y10
y2
322.1874
y9
1085.61
b6
829.405
b7
976.4404
b8
1075.509
y6
[1e5]
Peptide Sequence
Protein Sequence
- E D I V D T M V F K P S D V L L V H F R -
ac
ox

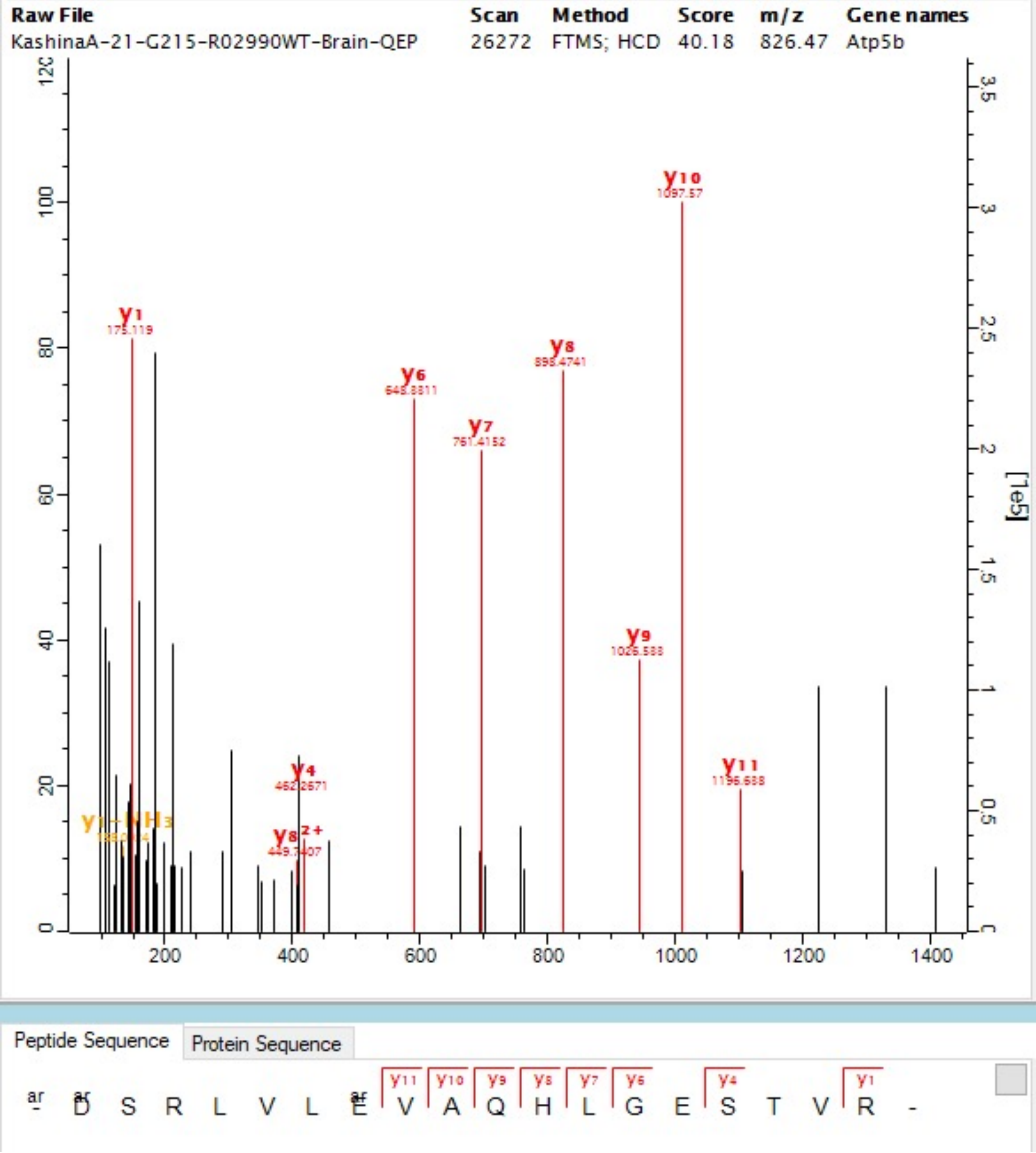

Raw File
KashinaA-21-G215-R02990WT-Brain-QEP
Scan
26272
Method
FTMS; HCD
Score
40.18
m/z
826.47
Gene names
Atp5b
y10
1097.57
y1
175.119
y6
y8
y7
761.4152
y9
y4
462.2671
y11
[1e5]
Peptide Sequence
Protein Sequence
D S R L V L E V A Q H L G E S T V R

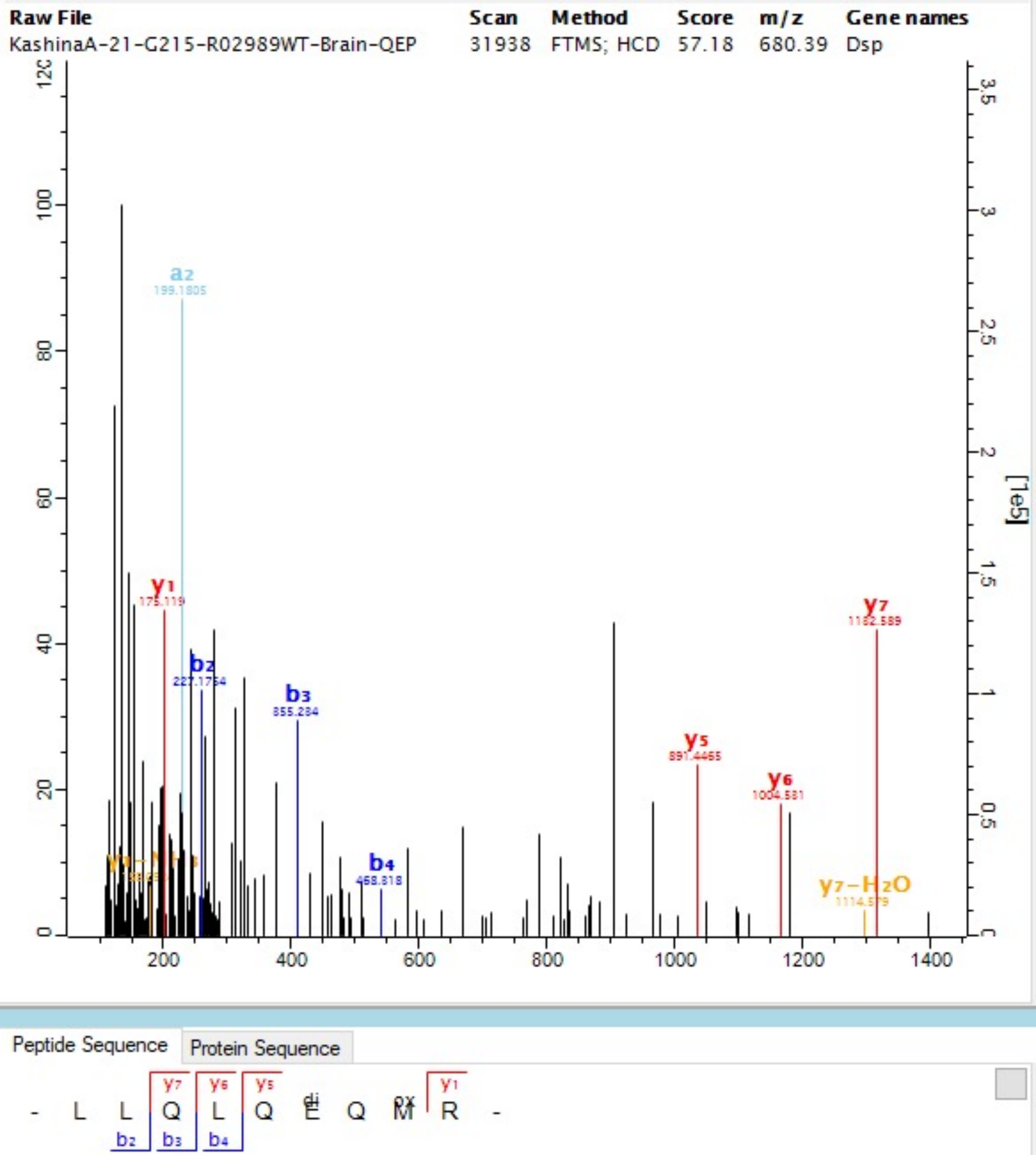
Raw File
KashinaA-21-G215-R02989WT-Brain-QEP
Scan
31938
Method
FTMS; HCD
Score
57.18
m/z
680.39
Gene names
Dsp
[1e5]
a2
199.1805
y1
175.119
b2
b3
b4
y5
891.4465
y6
y7
y7-H2O
Peptide Sequence
Protein Sequence
- L L Q L Q E Q M R -

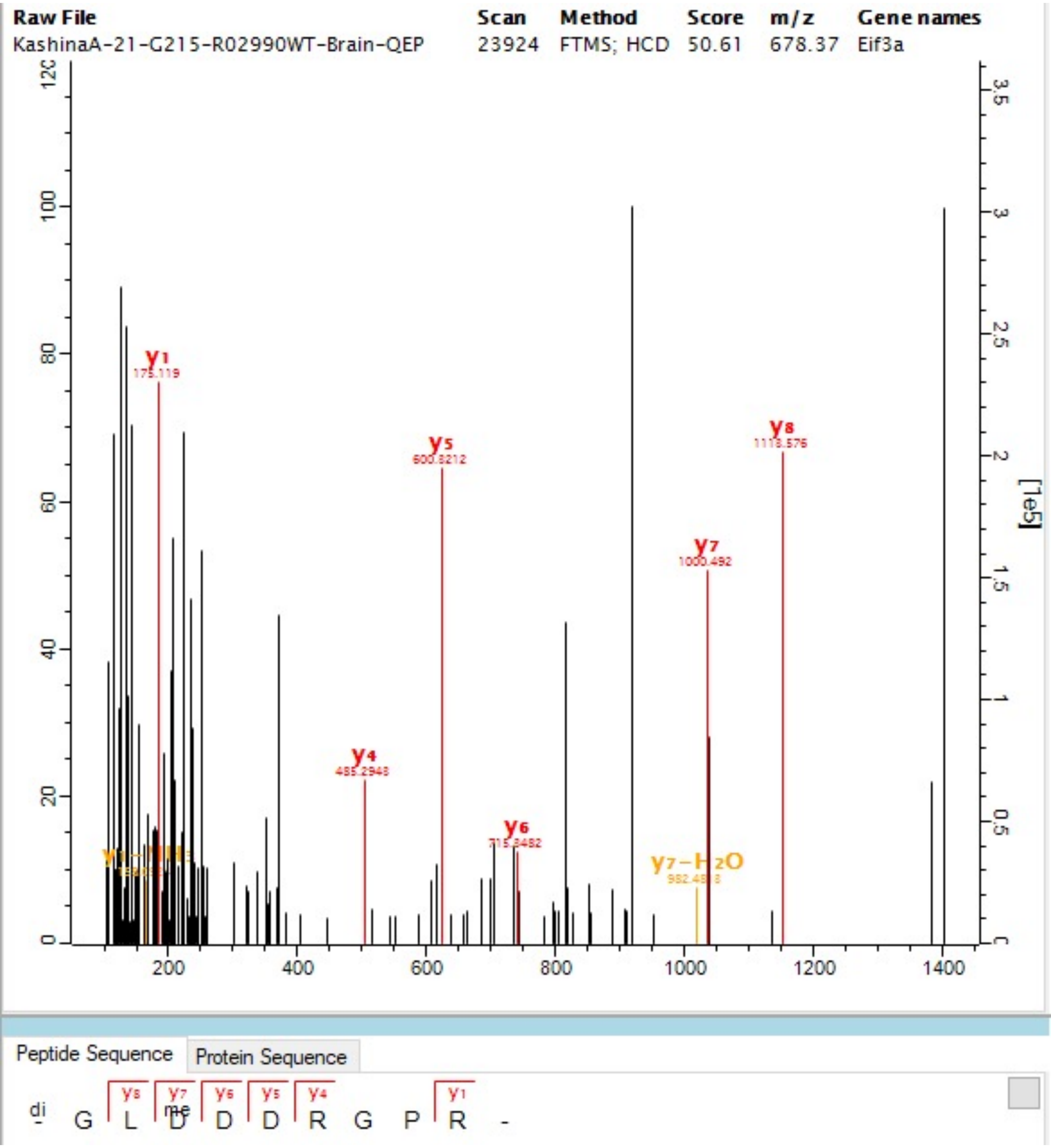

Raw File
KashinaA-21-G215-R02990WT-Brain-QEP
Scan
23924
Method
FTMS; HCD
Score
50.61
m/z
678.37
Gene names
Eif3a
y1
y4
y5
y6
y7
y8
y7-H2O
[1e5]
Peptide Sequence
Protein Sequence
di
me
G L D D D R G P R

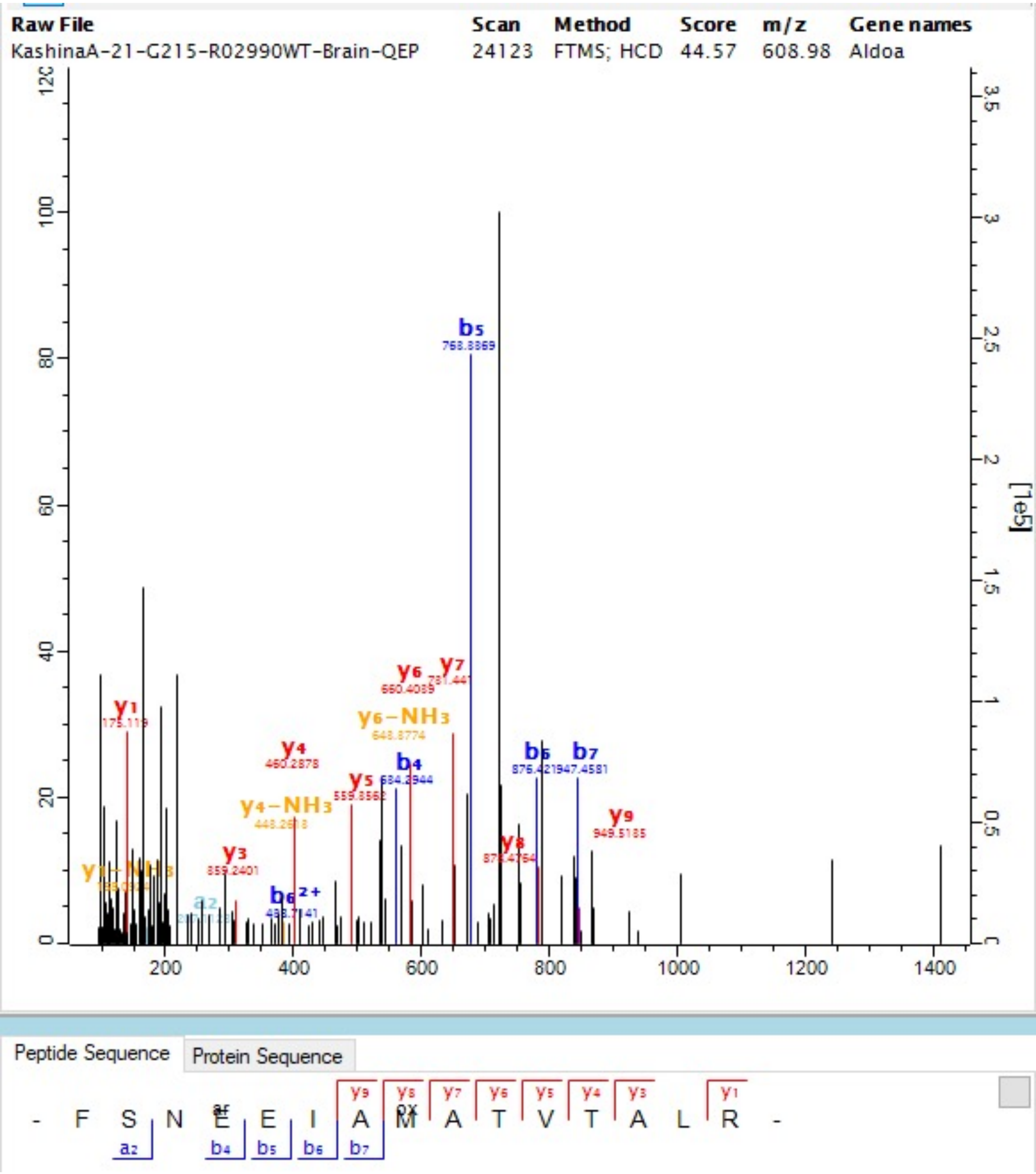
Raw File
KashinaA-21-G215-R02990WT-Brain-QEP
Scan
24123
Method
FTMS; HCD
Score
44.57
m/z
608.98
Gene names
Aldoa
b5
b4
b6
b7
b6 2+
a2
y1
y3
y4
y5
y6
y7
y8
y9
y4−NH3
y6−NH3
[1e5]
Peptide Sequence
Protein Sequence
- F S N E E I A M A T V T A L R -

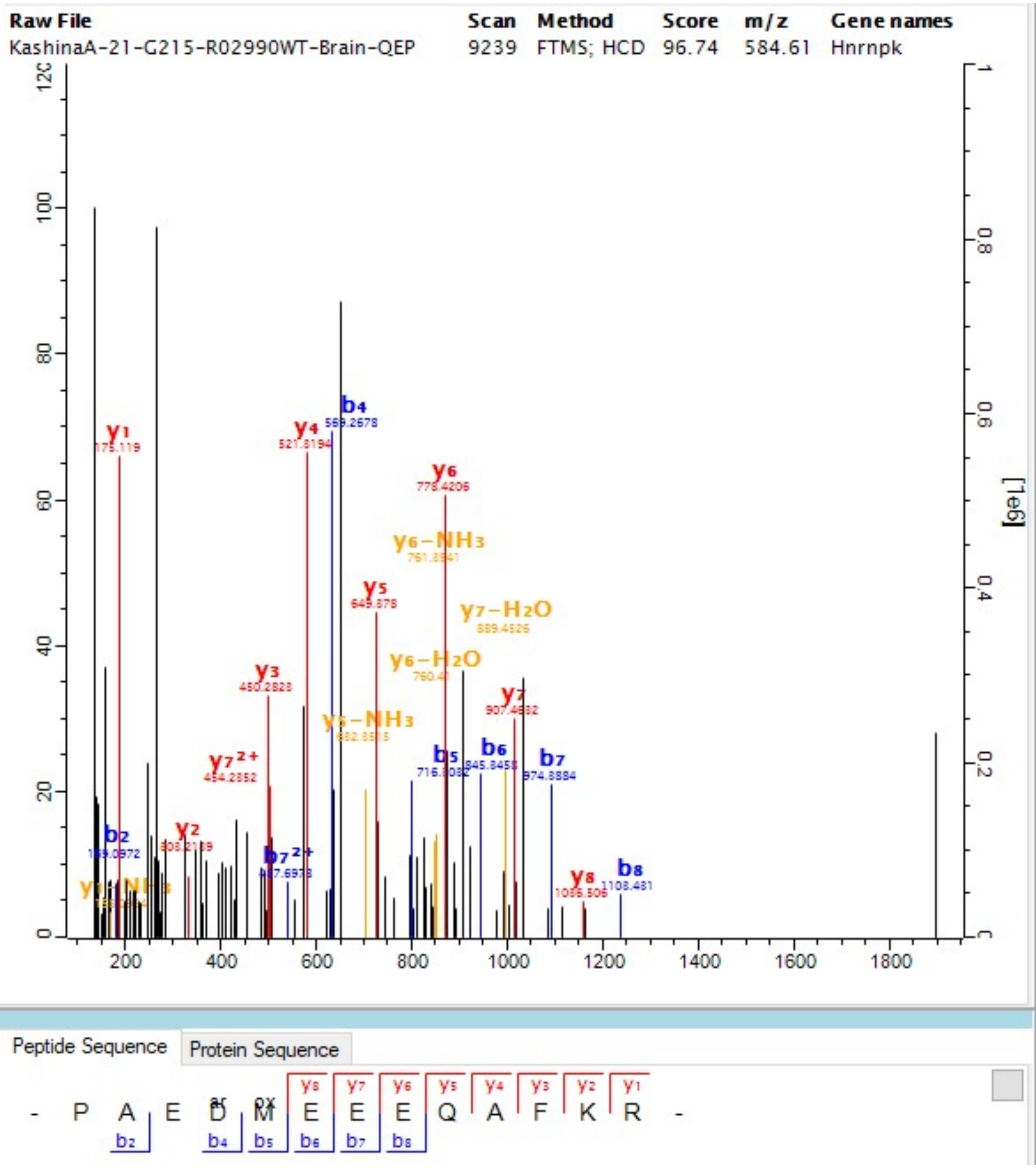
Raw File
KashinaA-21-G215-R02990WT-Brain-QEP
Scan
9239
Method
FTMS; HCD
Score
96.74
m/z
584.61
Gene names
Hnrnpk
[1e6]
y1
175.119
y4
521.8194
b4
569.2678
y6
778.4206
y6−NH3
761.3941
y5
649.378
y7−H2O
889.4526
y6−H2O
760.41
y3
450.2823
y7
907.4632
y5−NH3
632.3515
b5
716.3082
b6
845.3458
b7
974.3884
y7 2+
454.2352
b2
169.0972
y2
303.2139
b7 2+
487.6978
y8
1036.506
b8
1103.481
Peptide Sequence
Protein Sequence
- P A E D M E E E Q A F K R -
ox
y8 y7 y6 y5 y4 y3 y2 y1
b2 b4 b5 b6 b7 b8

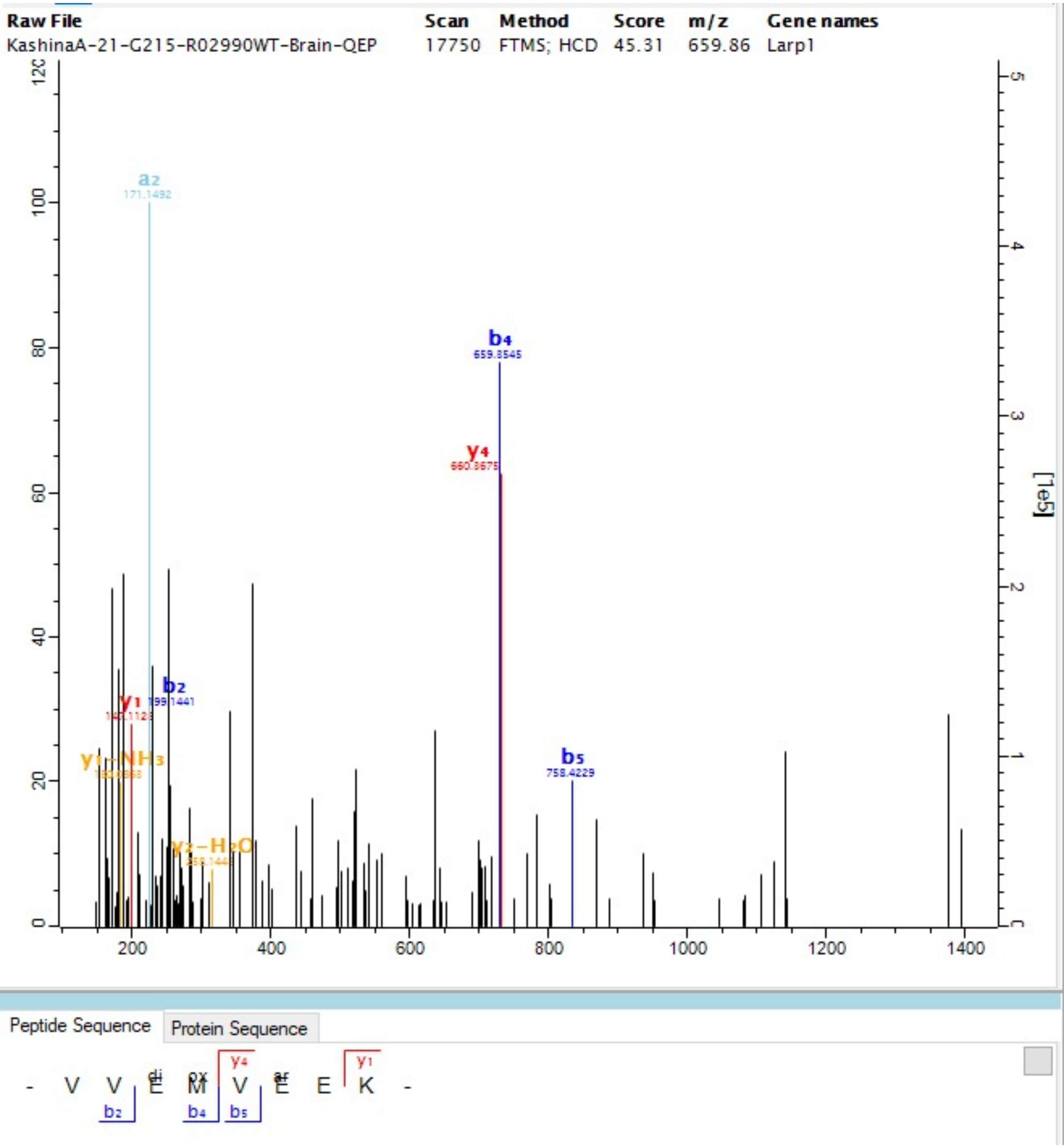

Raw File
KashinaA-21-G215-R02990WT-Brain-QEP
Scan
17750
Method
FTMS; HCD
Score
45.31
m/z
659.86
Gene names
Larp1
a2
171.1492
b4
659.8545
y4
660.3675
b2
199.1441
y1
b5
758.4229
y1-NH3
y2-H2O
[1e5]
Peptide Sequence
Protein Sequence
- V V E M V E E K -

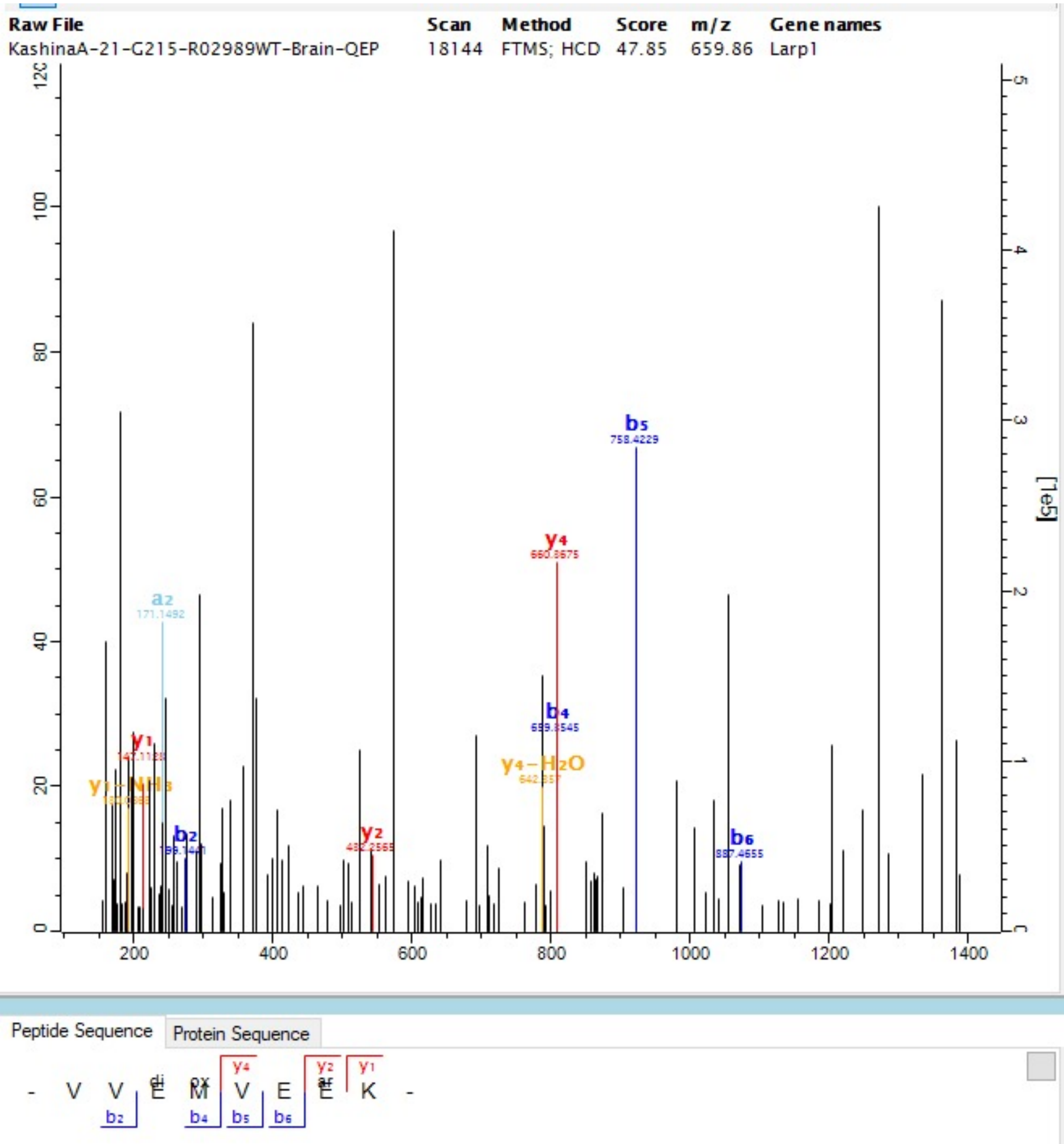
Raw File
KashinaA-21-G215-R02989WT-Brain-QEP
Scan
18144
Method
FTMS; HCD
Score
47.85
m/z
659.86
Gene names
Larp1
b5
758.4229
y4
a2
171.1492
b4
y4-H2O
y1
y2
b2
b6
887.4655
[1e5]
Peptide Sequence
Protein Sequence
- V V E M V E E K -
di
ox
ar
y4
y2
y1
b2
b4
b5
b6

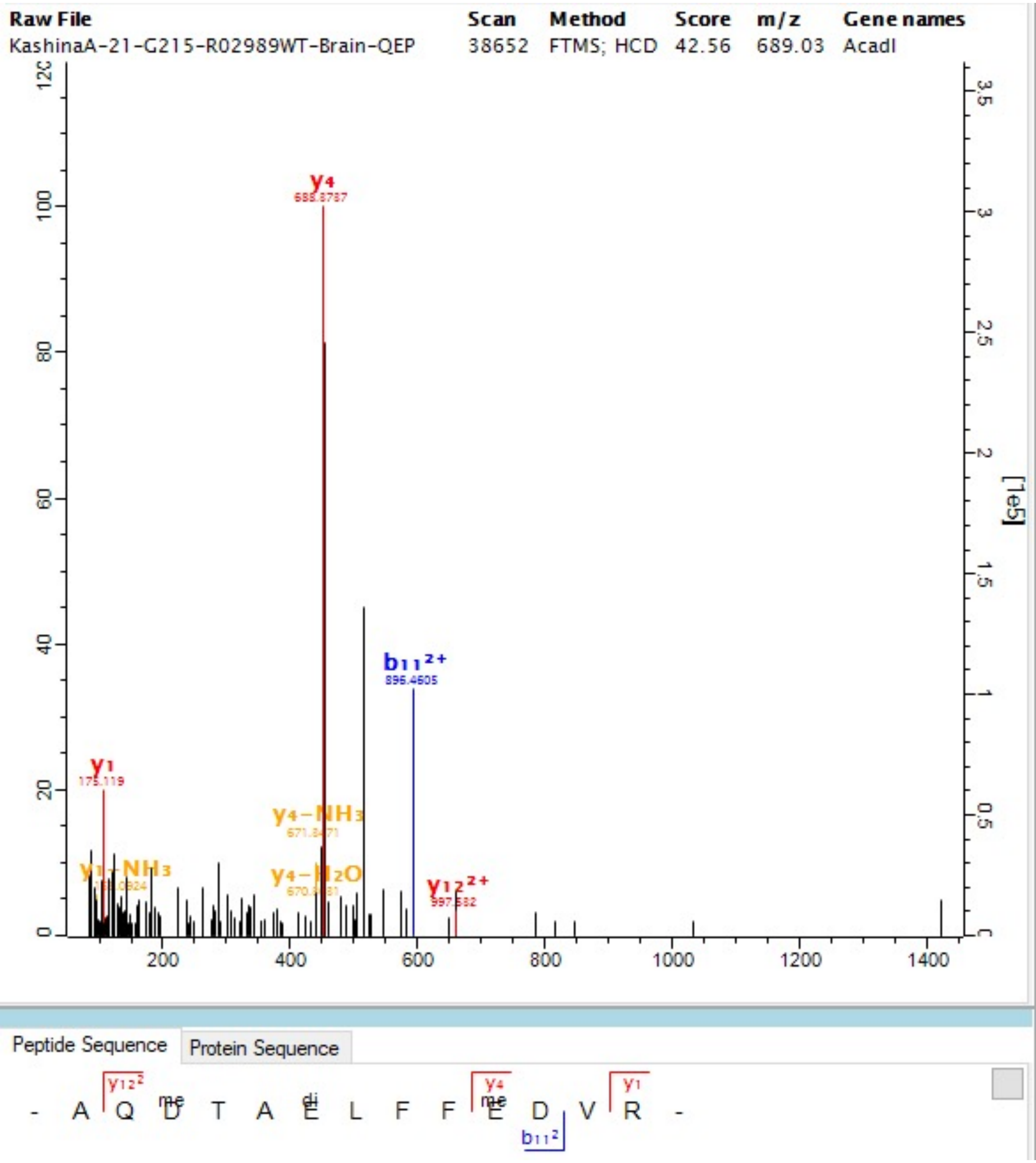

Raw File
KashinaA-21-G215-R02989WT-Brain-QEP
Scan
38652
Method
FTMS; HCD
Score
42.56
m/z
689.03
Gene names
Acadl
y4
b11 2+
896.4605
y1
175.119
y4-NH3
y1-NH3
y4-H2O
y12 2+
[1e5]
Peptide Sequence
Protein Sequence
- A Q D T A E L F F E D V R -

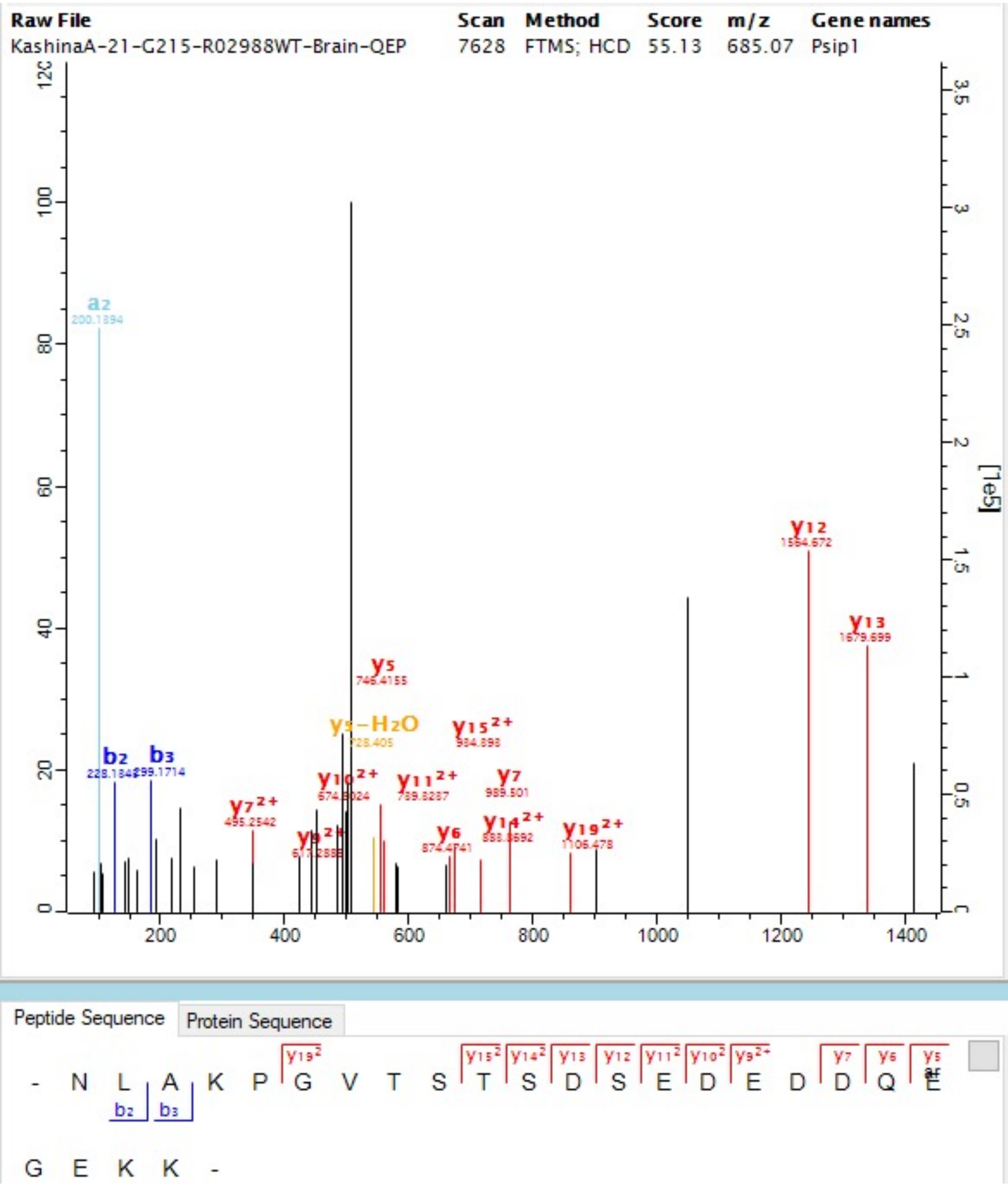
Raw File
KashinaA-21-G215-R02988WT-Brain-QEP
Scan
7628
Method
FTMS; HCD
Score
55.13
m/z
685.07
Gene names
Psip1
[1e5]
a2
200.1894
b2
228.1848
b3
299.1714
y7 2+
495.2542
y5-H2O
728.405
y5
746.4155
y10 2+
674.3024
y11 2+
789.8287
y15 2+
984.898
y7
989.501
y6
874.4741
y14 2+
y19 2+
1106.478
y12
y13
1679.699
Peptide Sequence
Protein Sequence
- N L A K P G V T S T S D S E D E D D Q E
G E K K -

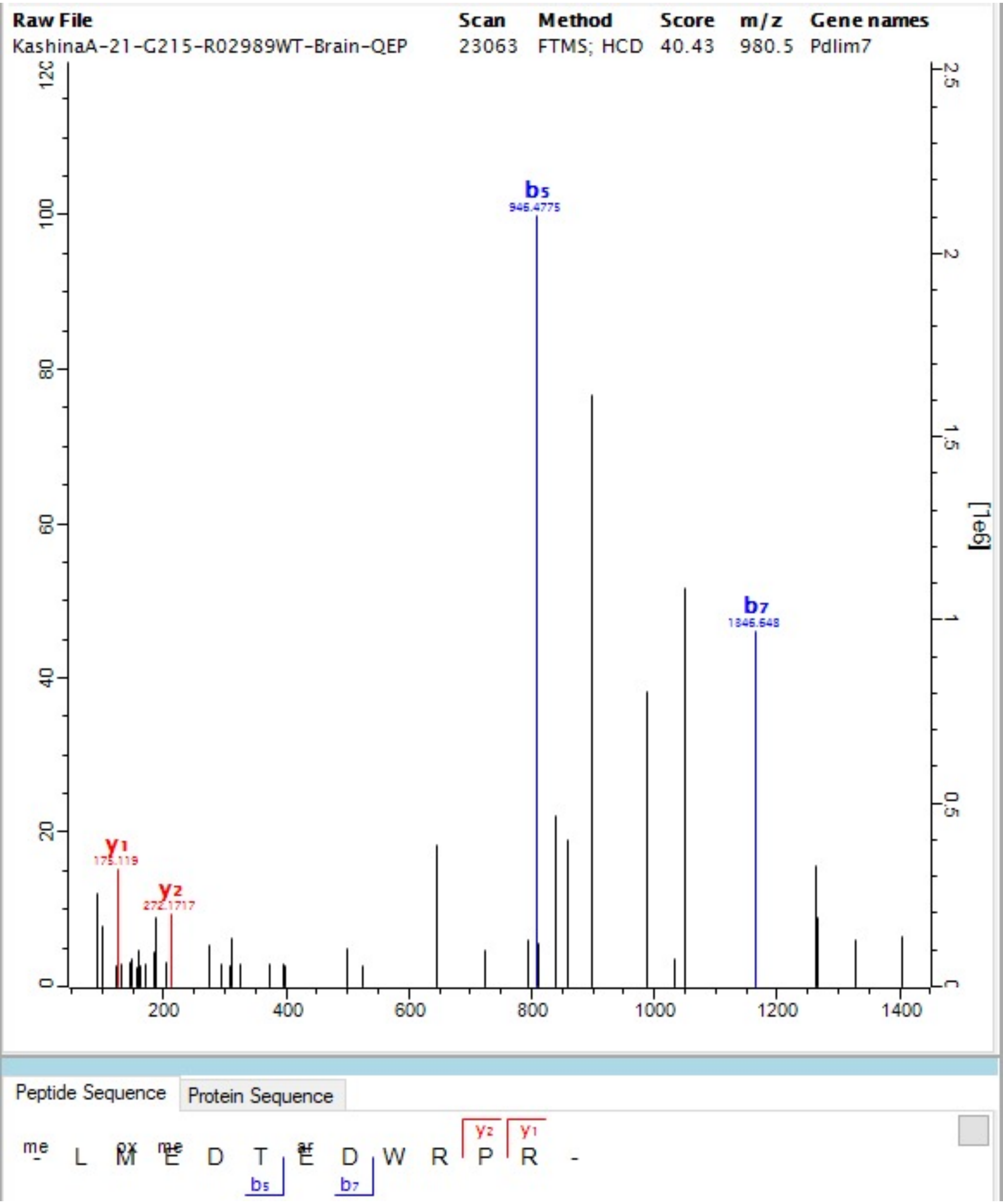

Raw File
KashinaA-21-G215-R02989WT-Brain-QEP
Scan
23063
Method
FTMS; HCD
Score
40.43
m/z
980.5
Gene names
Pdlim7
b5
946.4775
b7
1345.648
y1
175.119
y2
272.1717
0
20
40
60
80
100
200
400
600
800
1000
1200
1400
0.5
1
1.5
2
2.5
[1e6]
Peptide Sequence
Protein Sequence
me
ox
me
ac
- L M E D T E D W R P R -
b5
b7
y2
y1

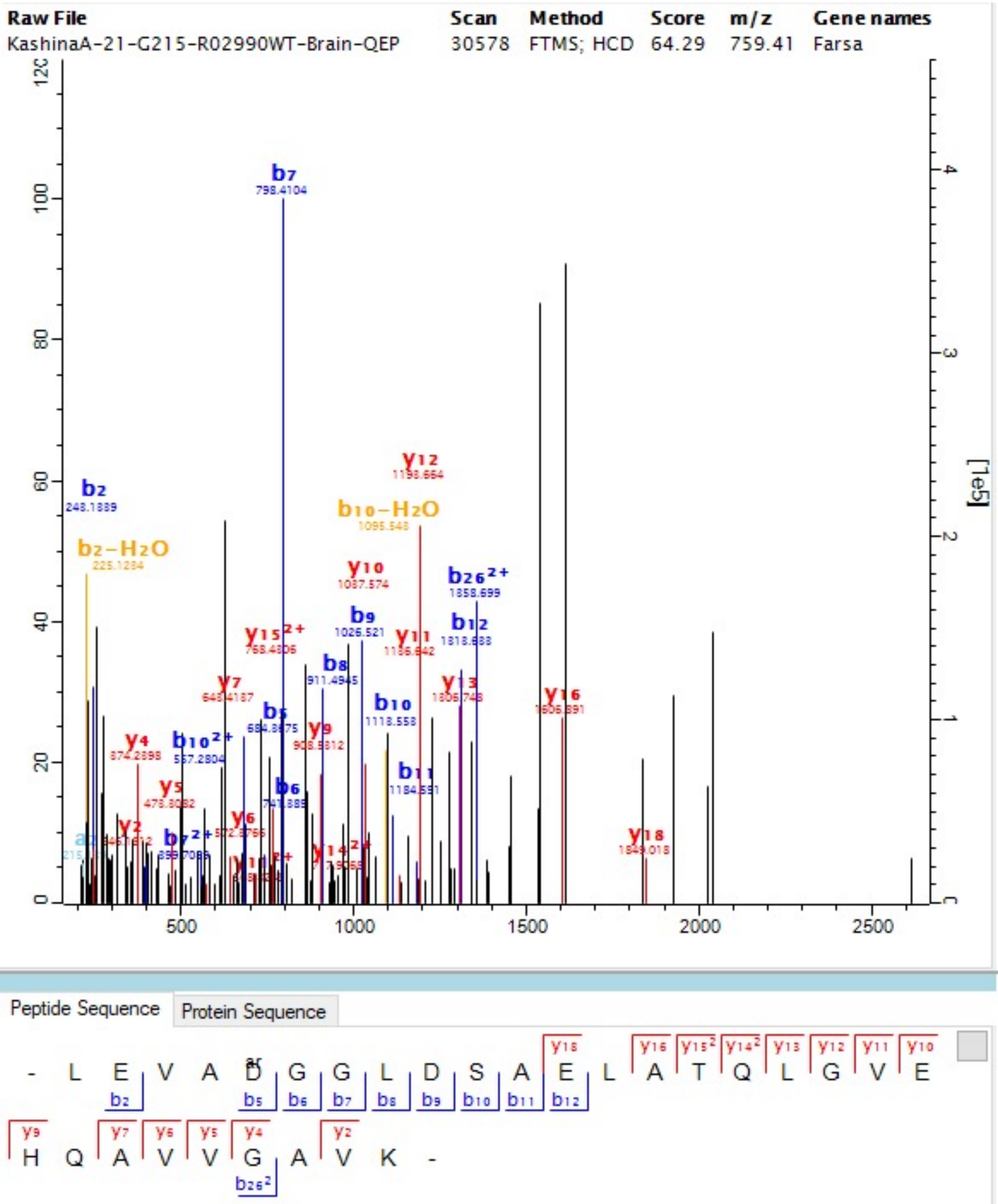

Raw File
KashinaA-21-G215-R02990WT-Brain-QEP
Scan
30578
Method
FTMS; HCD
Score
64.29
m/z
759.41
Gene names
Farsa
b7
798.4104
b2
248.1889
b2-H2O
225.1284
y12
1198.664
b10-H2O
1095.548
y10
1087.574
b26 2+
1858.699
b9
1026.521
b12
1318.688
y15 2+
768.4306
y11
1186.642
b8
911.4945
y7
648.4187
y13
1306.748
y16
1606.891
b10
1118.558
b5
684.3675
y9
906.5312
y4
374.2898
b10 2+
557.2804
b11
1184.591
b6
741.885
y5
473.3082
y6
572.3766
y2
y18
1849.018
Peptide Sequence
Protein Sequence
- L E V A D G G L D S A E L A T Q L G V E
H Q A V V G A V K -

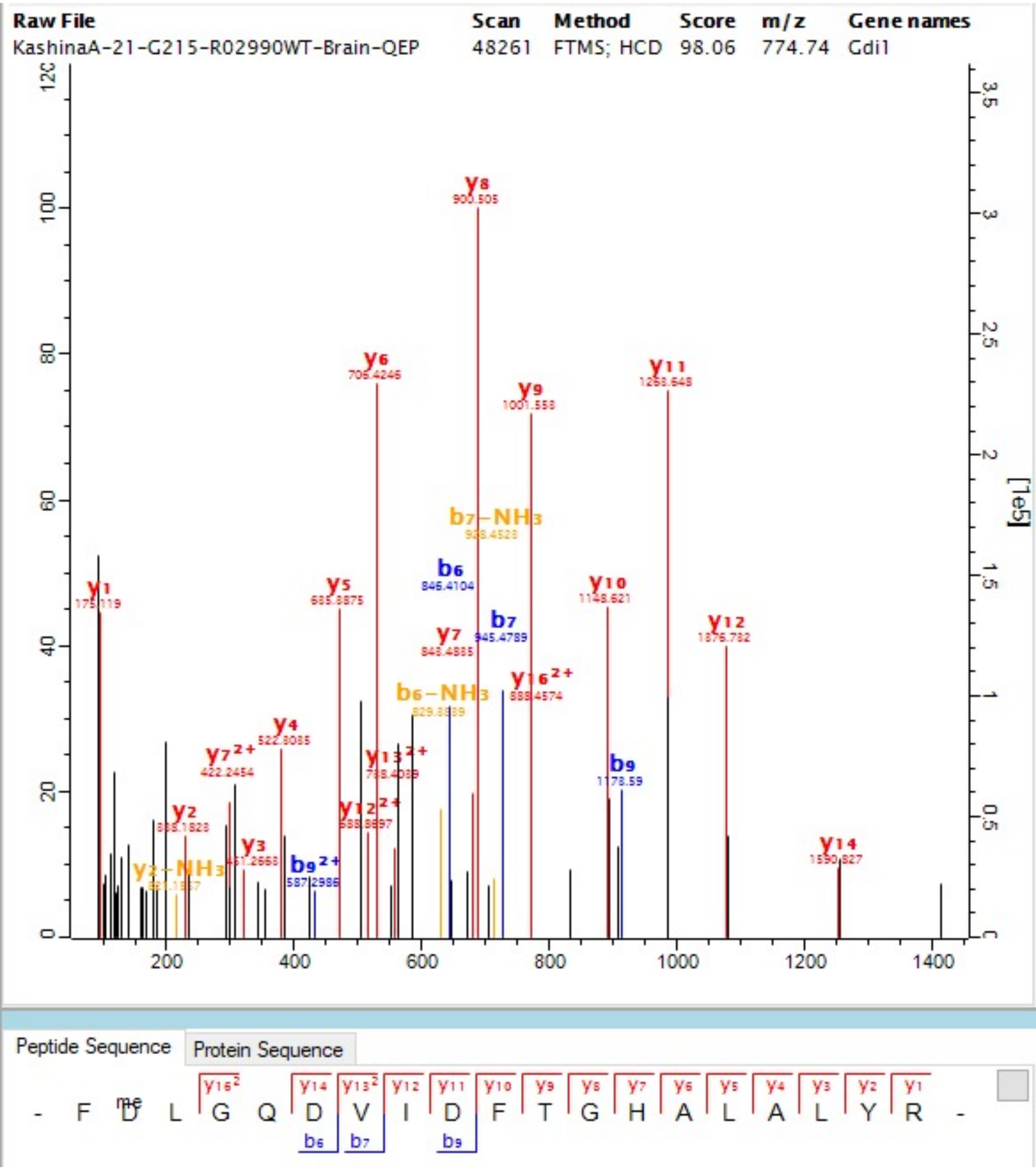

Raw File
KashinaA-21-G215-R02990WT-Brain-QEP
Scan
48261
Method
FTMS; HCD
Score
98.06
m/z
774.74
Gene names
Gdi1
y8
900.505
y6
706.4246
y9
1001.553
y11
b7-NH3
b6
846.4104
y1
175.119
y5
y10
y7
b7
945.4789
y12
y16 2+
b6-NH3
y4
522.3085
y7 2+
422.2454
y13 2+
b9
1178.59
y2
y12 2+
y3
b9 2+
y2-NH3
y14
Peptide Sequence
Protein Sequence
- F D L G Q D V I D F T G H A L A L Y R -
me
y16 2
y14
y13 2
y12
y11
y10
y9
y8
y7
y6
y5
y4
y3
y2
y1
b6
b7
b9
[1e5]

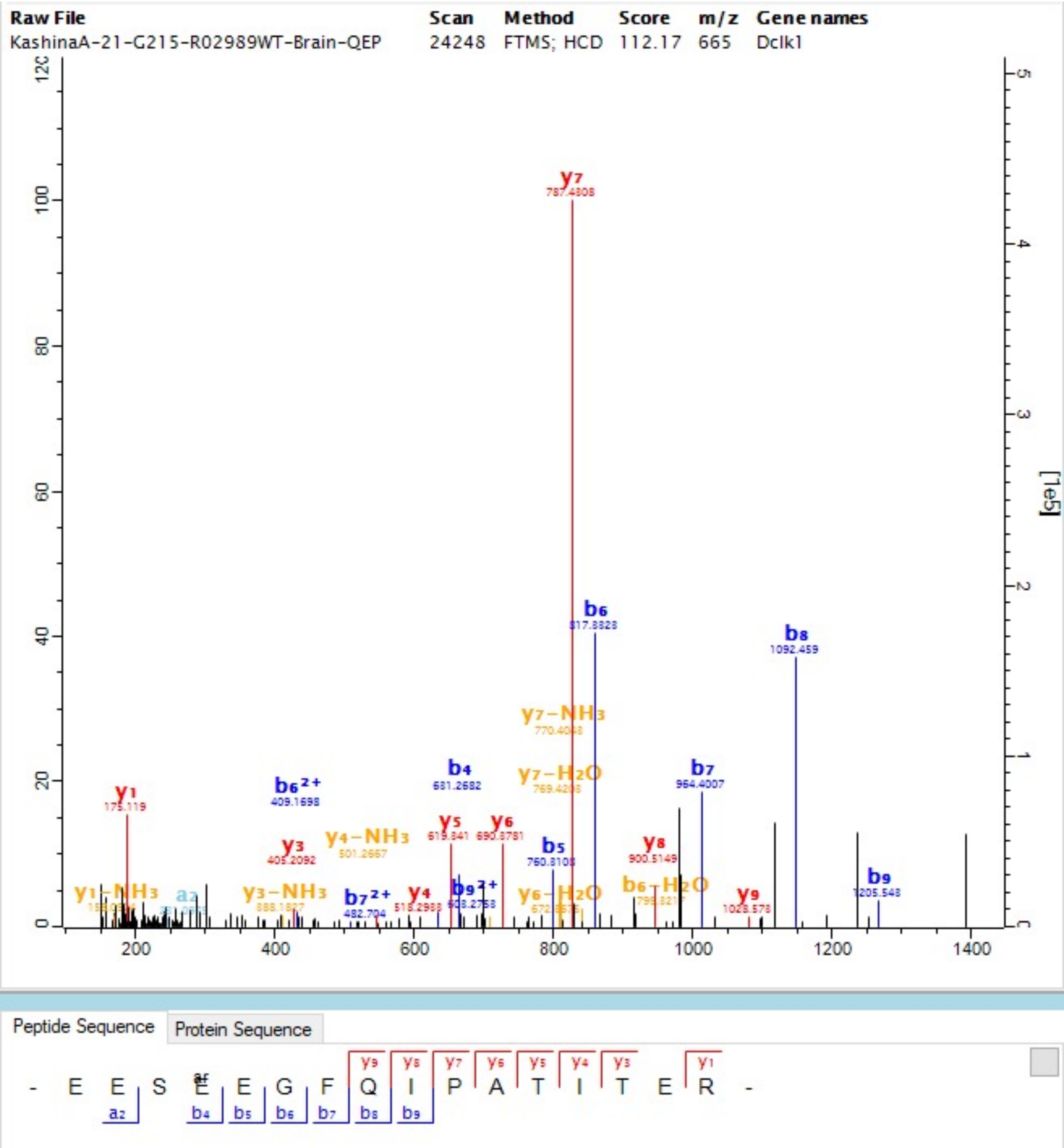

Raw File
KashinaA-21-G215-R02989WT-Brain-QEP
Scan
24248
Method
FTMS; HCD
Score
112.17
m/z
665
Gene names
Dclk1
[1e5]
y7
b6
b8
y7-NH3
y7-H2O
b4
b7
b6 2+
409.1698
y1
175.119
y5
y6
y3
y4-NH3
b5
y8
y1-NH3
a2
y3-NH3
b7 2+
y4
b9 2+
y6-H2O
b6-H2O
y9
b9
Peptide Sequence
Protein Sequence
- E E S E E G F Q I P A T I T E R -

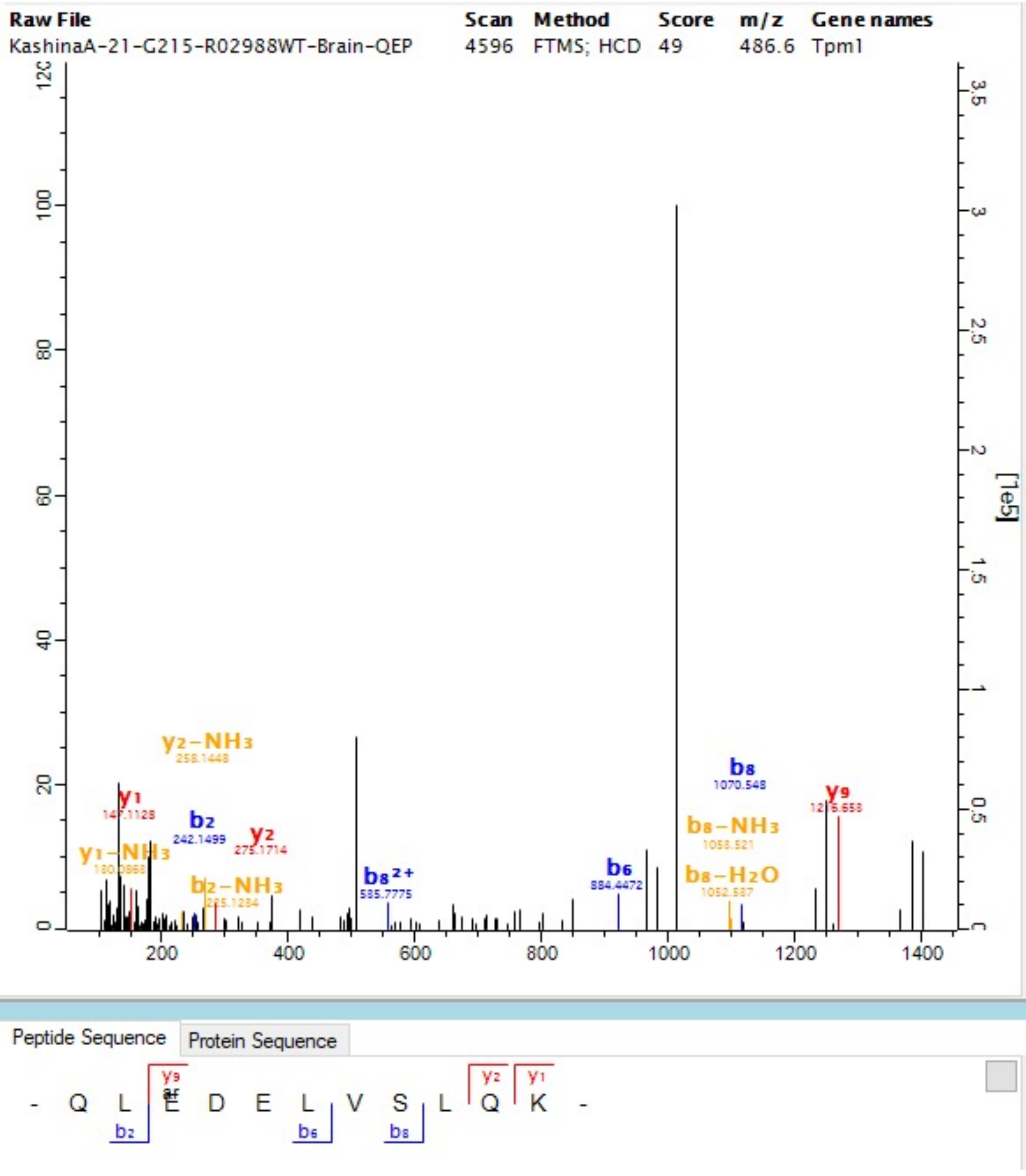
Raw File
KashinaA-21-G215-R02988WT-Brain-QEP
Scan
4596
Method
FTMS; HCD
Score
49
m/z
486.6
Gene names
Tpm1
[1e5]
y2−NH3
y1
y1−NH3
b2
242.1499
y2
275.1714
b2−NH3
b8 2+
585.7775
b6
884.4472
b8
1070.548
b8−NH3
1053.521
b8−H2O
1052.537
y9
Peptide Sequence
Protein Sequence
- Q L E D E L V S L Q K -
y9
y2
y1
b2
b6
b8

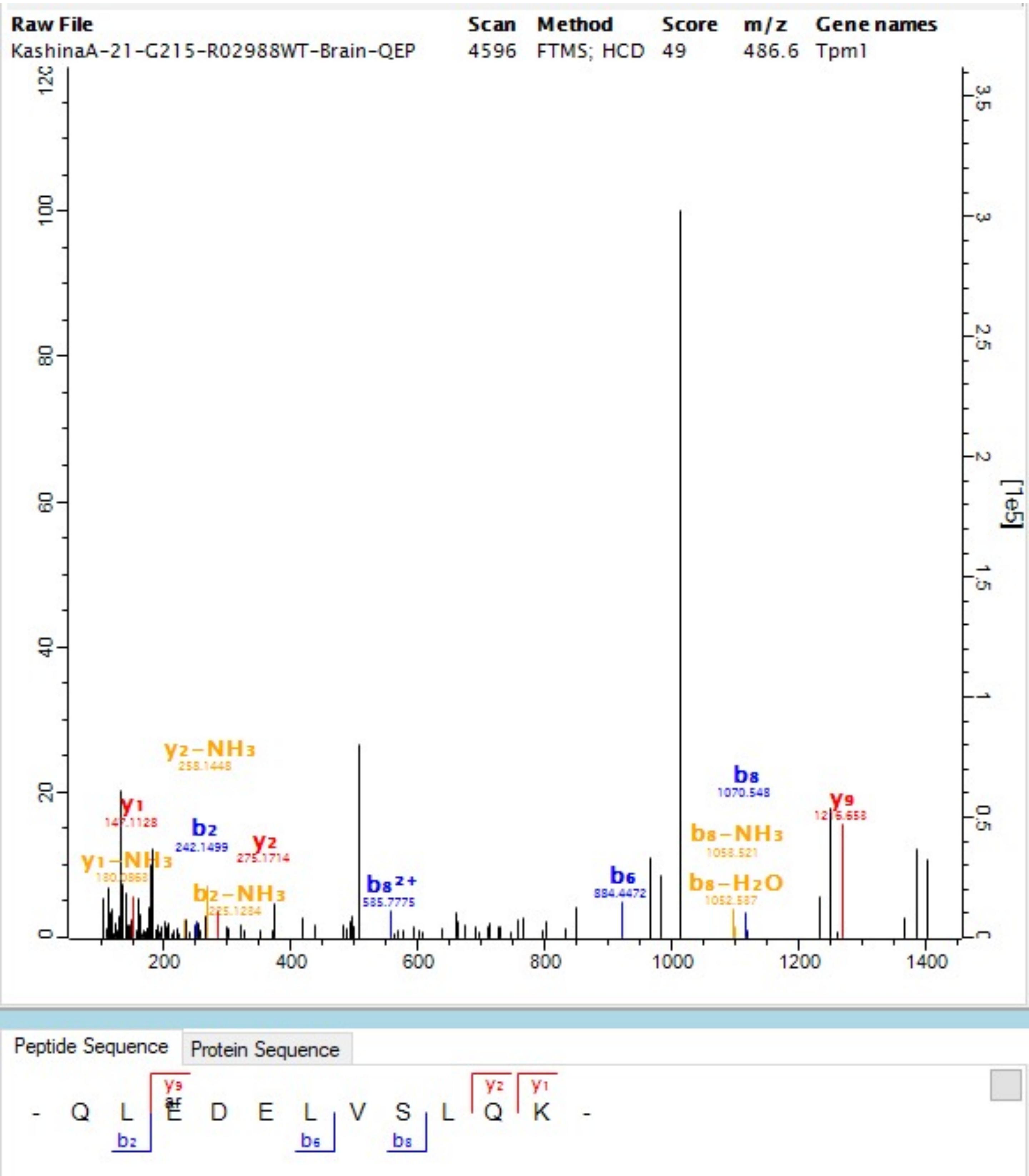

Raw File
KashinaA-21-G215-R02988WT-Brain-QEP
Scan
4596
Method
FTMS; HCD
Score
49
m/z
486.6
Gene names
Tpm1
y2-NH3
y1
b2
242.1499
y2
y1-NH3
b2-NH3
b8 2+
585.7775
b6
884.4472
b8
1070.548
b8-NH3
b8-H2O
y9
[1e5]
Peptide Sequence
Protein Sequence
- Q L E D E L V S L Q K -

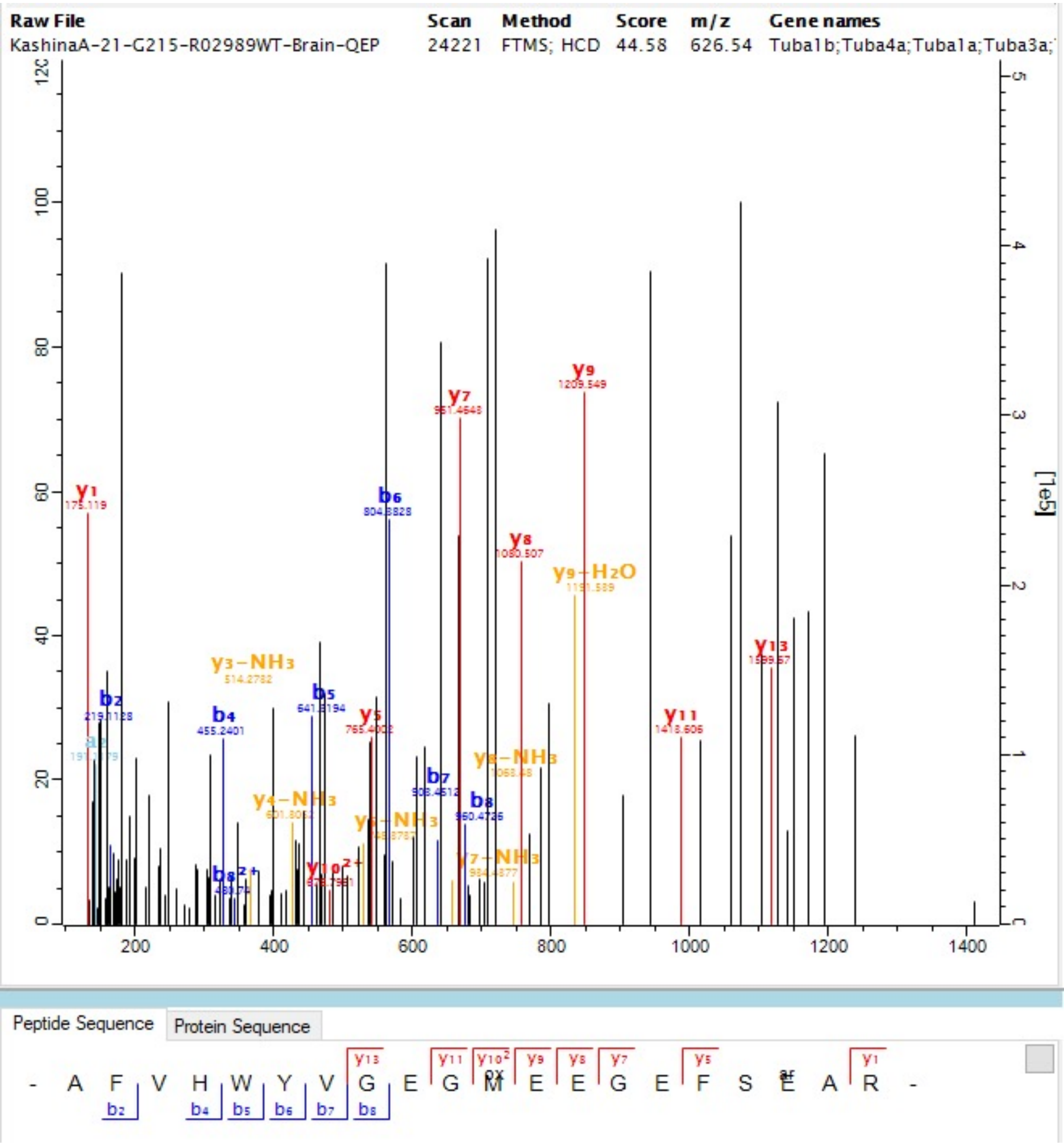
Raw File
KashinaA-21-G215-R02989WT-Brain-QEP
Scan
24221
Method
FTMS; HCD
Score
44.58
m/z
626.54
Gene names
Tuba1b;Tuba4a;Tuba1a;Tuba3a;
y1
175.119
b2
219.1128
y3-NH3
514.2782
b4
455.2401
b5
641.3194
y5
765.4002
b6
804.3828
y7
951.4648
y8
1080.507
y9
1209.549
y9-H2O
1191.539
y8-NH3
1063.48
b7
903.4512
b8
960.4726
y4-NH3
601.3052
y7-NH3
934.4377
y11
1418.606
y13
1599.67
b8 2+
480.74
y10 2+
678.7981
[1e5]
Peptide Sequence
Protein Sequence
- A F V H W Y V G E G M E E G E F S E A R -
ox
y13 y11 y10 2 y9 y8 y7 y5 y1
b2 b4 b5 b6 b7 b8

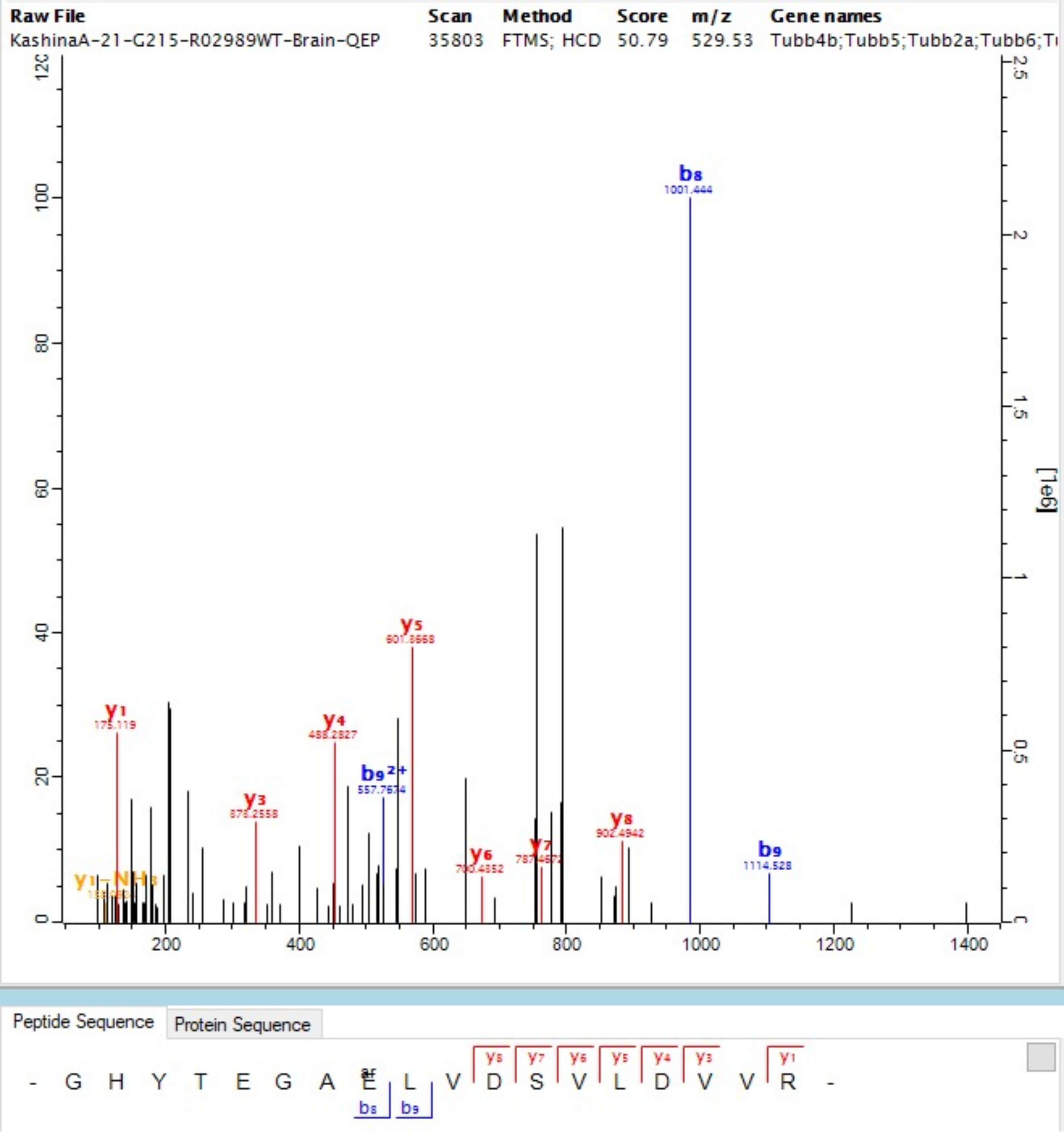
Raw File
KashinaA-21-G215-R02989WT-Brain-QEP
Scan
35803
Method
FTMS; HCD
Score
50.79
m/z
529.53
Gene names
Tubb4b;Tubb5;Tubb2a;Tubb6;Tu
[1e6]
b8
1001.444
y1
175.119
y3
y4
b9 2+
557.7674
y5
y6
y7
y8
902.4942
b9
1114.528
y1-NH3
Peptide Sequence
Protein Sequence
- G H Y T E G A E L V D S V L D V V R -

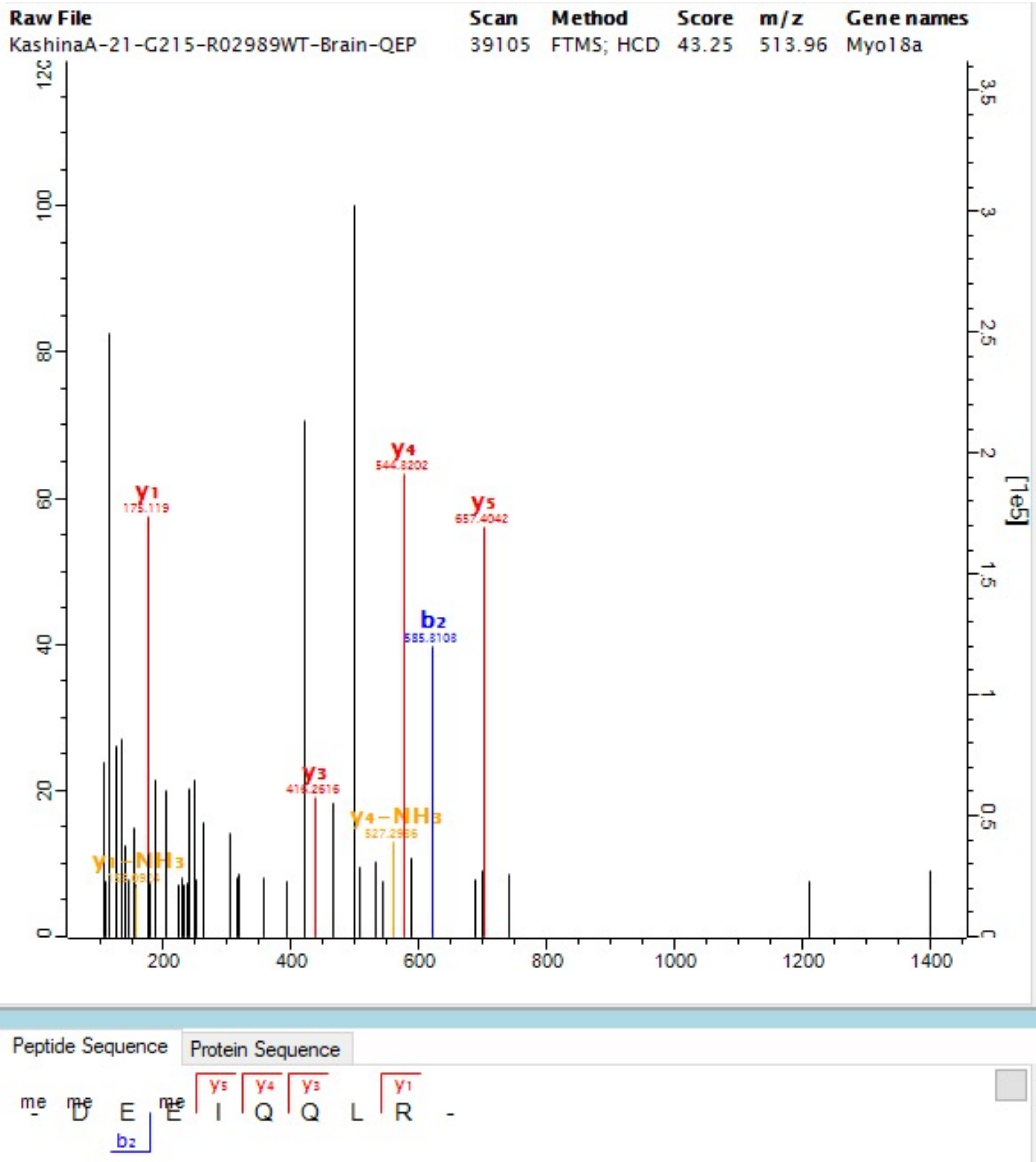
Raw File
KashinaA-21-G215-R02989WT-Brain-QEP
Scan
39105
Method
FTMS; HCD
Score
43.25
m/z
513.96
Gene names
Myo18a
0
20
40
60
80
100
0.5
1
1.5
2
2.5
3
3.5
[1e5]
200
400
600
800
1000
1200
1400
y1
175.119
y1-NH3
y3
416.2616
y4-NH3
527.2956
y4
544.3202
b2
585.3108
y5
657.4042
Peptide Sequence
Protein Sequence
me
me
me
-
D
E
E
I
Q
Q
L
R
-
y5
y4
y3
y1
b2
