# Supplementary material for: Global Analysis of Post-Translational Side-Chain Arginylation Using Pan-Arginylation Antibodies
Source: Mol Cell Proteomics. 2023 Oct 12;22(11):100664. doi: 10.1016/j.mcpro.2023.100664 (PMC10656225; doi:10.1016/j.mcpro.2023.100664)
Supplement: Supplemental Data [file mmc5.pdf]

Global analysis of post-translational sidechain arginylation using pan-arginylation antibodies

Brittany MacTaggart[1], Marie Shimogawa[2], Marshall Lougee[2], Hsin-Yao Tang[3], E. J. Petersson[2], and Anna Kashina[1]*

[1]University of Pennsylvania, School of Veterinary Medicine, Philadelphia, PA 19104; [2]Department of Chemistry, University of Pennsylvania School of Arts and Sciences, Philadelphia, PA 19104; [3]Proteomics and Metabolomics Facility, Wistar Institute, Philadelphia, PA 19104

SUPPLEMENTAL ONLINE INFORMATION

LIST OF SUPPLEMENTARY FILES:

Supplementary Table 1. Quantification data for Table 1.
Supplementary Table 2. Non-high confidence arginylation sites identified exclusively in wildtype samples.
Supplementary Table 3. High confidence arginylation sites identified in brain samples.
Supplementary Table 4. Non-high confidence arginylation sites identified in brain samples.
Supplementary File 1. Annotated spectra from Table 2.
Supplementary File 2. Annotated spectra from Supplementary Table 2.
Supplementary File 3. Annotated spectra from Supplementary Table 3.
Supplementary File 4. Annotated spectra from Supplementary Table 4.

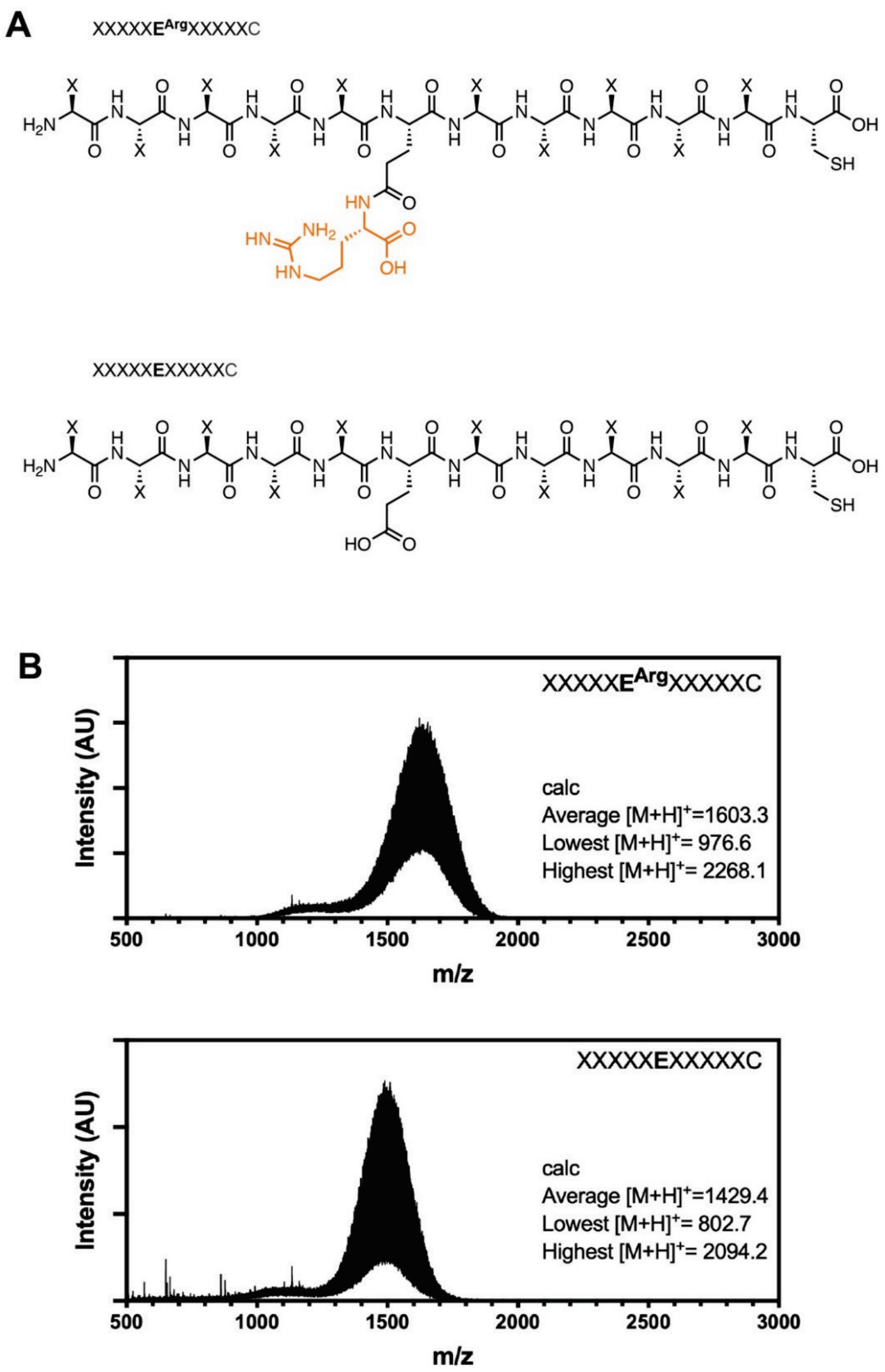

Figure S1. Related to Figure 1. Structure and characterization of arginylated peptides: (A) General structure of the product peptide libraries XXXXXE^{Arg}XXXXXC (upper) and XXXXXEXXXXXC (lower); (B) MALDI-MS of product peptide libraries XXXXXE^{Arg}XXXXXC (upper) and XXXXXEXXXXXC (lower).

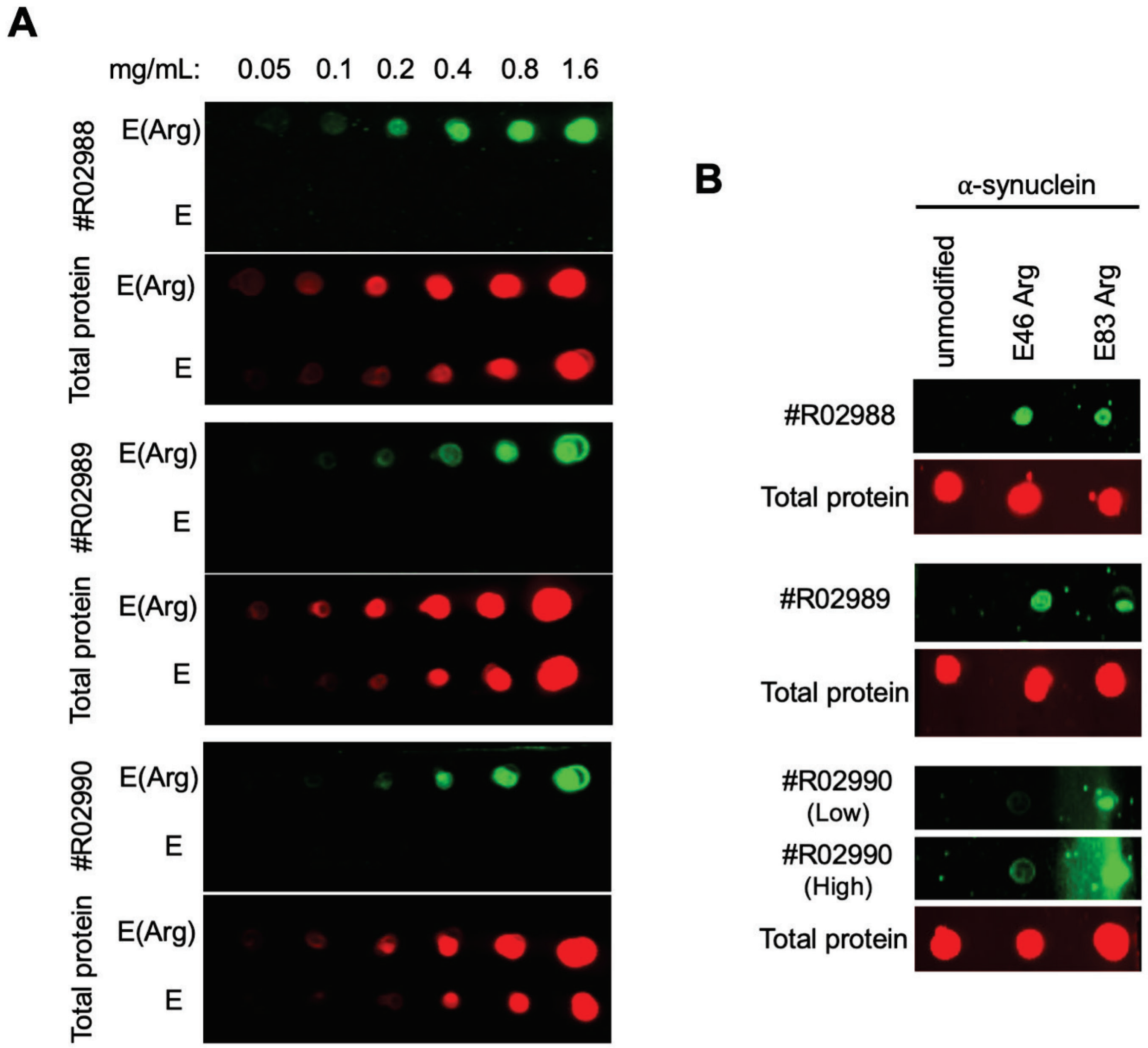

Figure S2. Related to Figure 1. Validation of all three pan-arginylation antibodies. (A) Dot blots of arginylated and unmodified peptide mixtures using all three pan-arginylation antibodies (green) and total protein stain (red). **(B)** Dot blots of arginylated (E46, E83) and unmodified α-synuclein using all three pan-arginylation antibodies (green) and total protein stain (red).

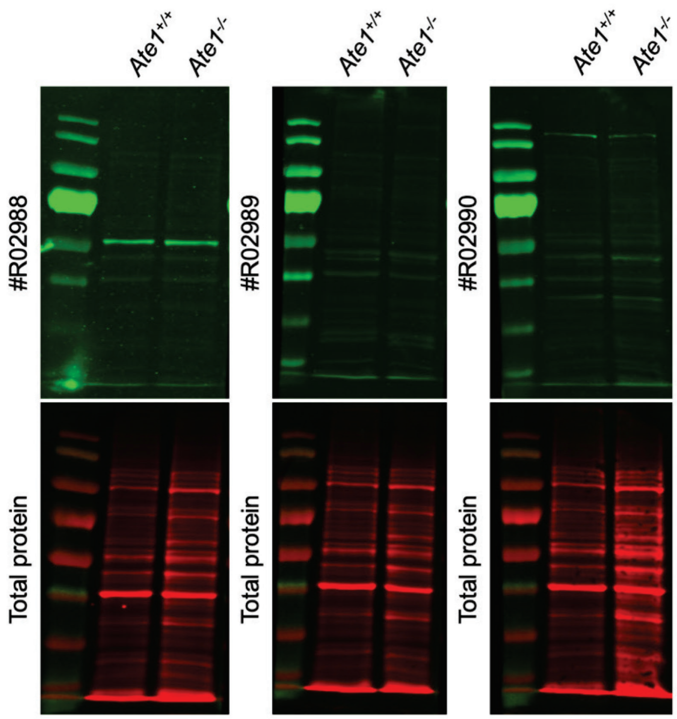

Figure S3. Related to Figure 1. Pan-arginylation antibodies are not suitable for Western blots on cell lysates. Western blots on *Ate1*$^{+/+}$ and *Ate1*$^{-/-}$ cell lysates using the three pan-arginylation antibodies (green) and total protein stain (red).

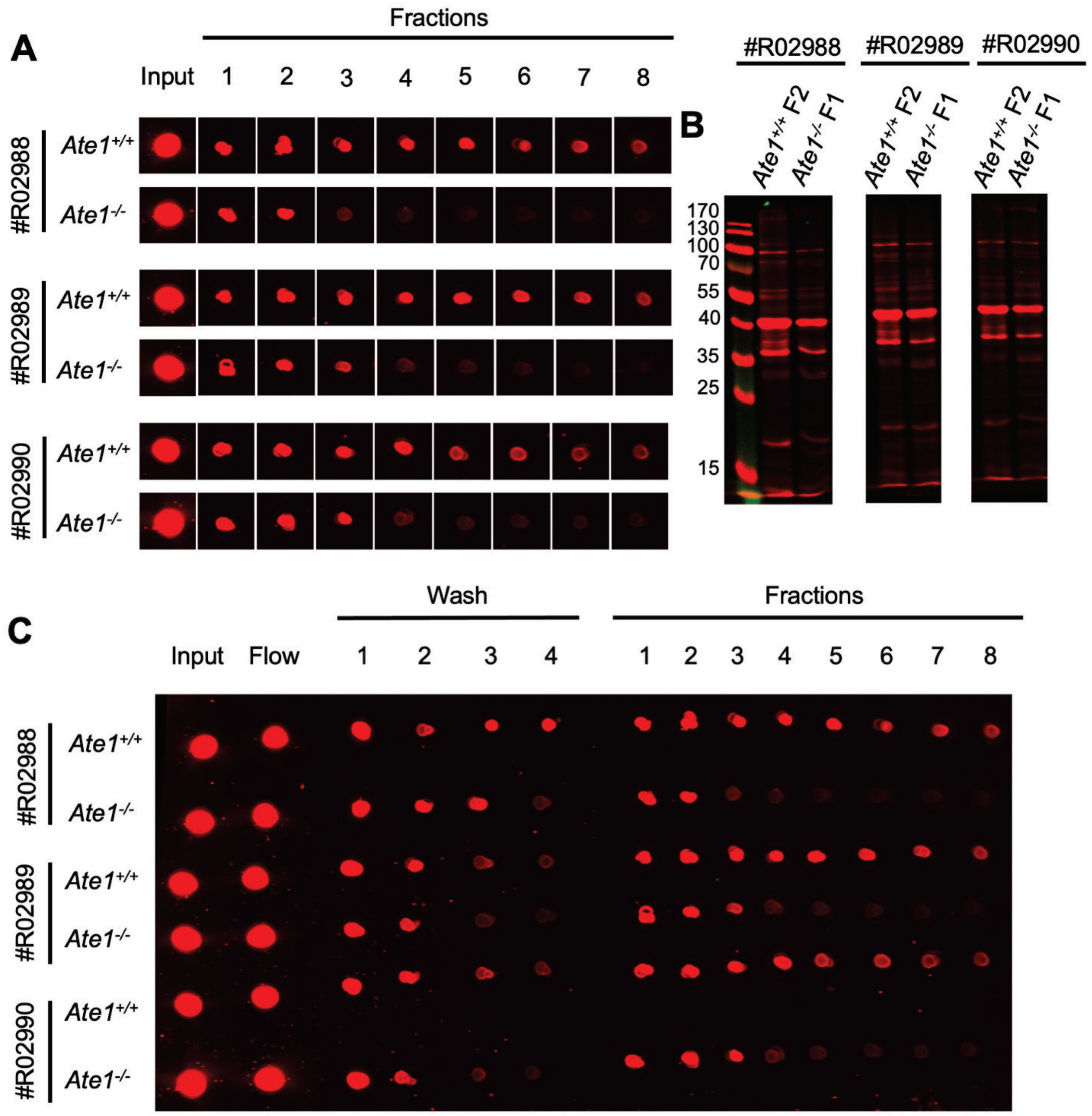

Figure S4. Related to Figure 2. Pan-arginylation pull downs from all three pan-arginylation antibodies. (A) Dot blot showing total protein stain of the input and eluted fractions for *Ate1*$^{+/+}$ and *Ate1*$^{-/-}$ cell lysates pulled down with all three pan-arginylation antibodies. Images were cropped to align the dots, but all scaling and quantification were done on the full image. For uncropped membrane, see Fig. S5. **(B)** Western blot of wildtype fraction 2 (*Ate1*$^{+/+}$ F2) and *Ate1* knockout fraction 1 (*Ate1*$^{-/-}$ F1) using total protein stain (red). **(C)** Uncropped dot blot showing total protein stain of the input, flow-through, washes, and eluted fractions for *Ate1*$^{+/+}$ and *Ate1*$^{-/-}$ cell lysates pulled down with all three pan-arginylation antibodies.

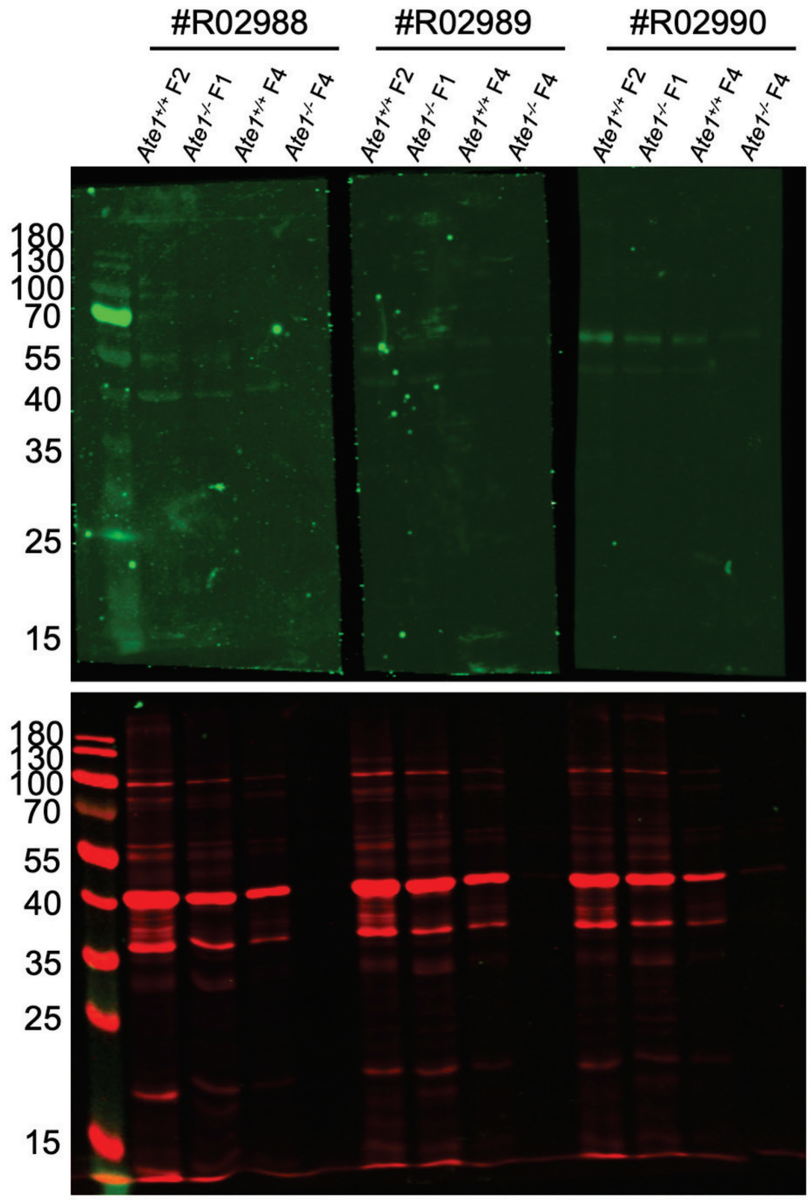

Figure S5. Related to Figure 2. Pan-arginylation antibodies are not suitable for Western blots on fractions from pan-arginylation pull down. Western blots on wildtype fractions 2 and 4 (*Ate1*$^{+/+}$ F2 and F4) and *Ate1* knockout fractions 1 and 4 (*Ate1*$^{-/-}$ F1 and F4) using the three pan-arginylation antibodies (green) and total protein stain (red).

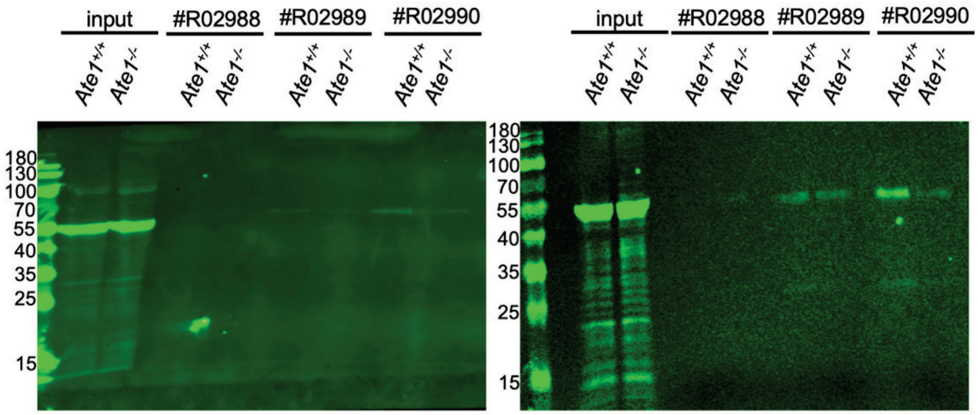

Figure S6. Related to Figure 3. Full membranes for confirmation Western blots. Uncropped Western blots of α-tubulin (left) and β-tubulin (right) enrichment in Ate1$^{+/+}$ samples.

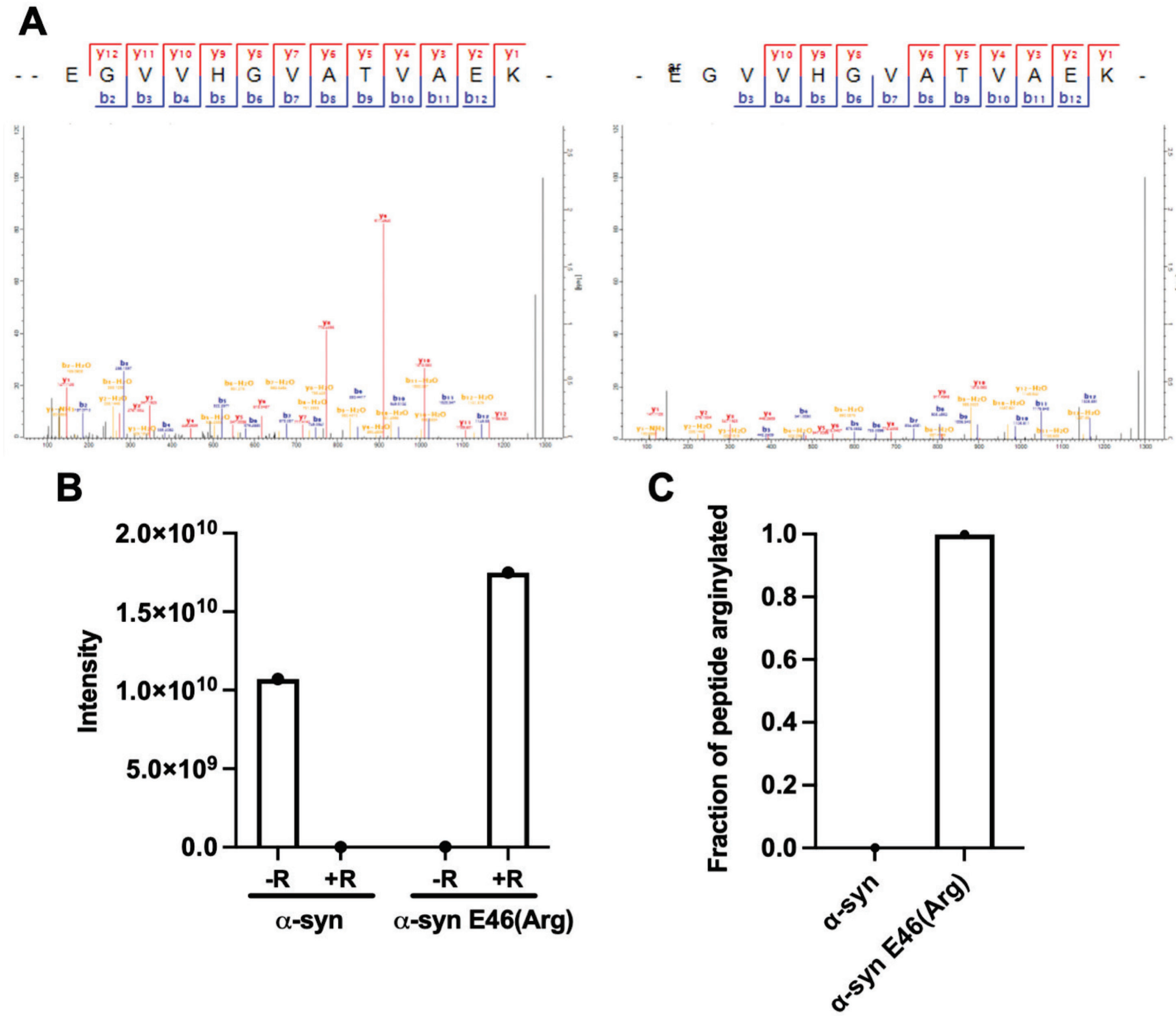

Figure S7. Related to Table 2, Supplementary Table 2, Supplementary Table 3, and Supplementary Table 4. Trypsin digestion during mass spectrometry analysis does not remove side chain arginylation. (A) Annotated spectra of unmodified (left) or arginylated (right) α-synuclein peptides. **(B)** Intensity of unmodified or arginylated (E46 Arg) α-synuclein peptides arginylated or unarginylated following trypsin digestion. **(C)** Fraction of unmodified or arginylated (E46 Arg) α-synuclein peptides arginylated following trypsin digestion.
